# *Corylus avellana*: A Source of Diarylheptanoids With α-Glucosidase Inhibitory Activity Evaluated by *in vitro* and *in silico* Studies

**DOI:** 10.3389/fpls.2022.805660

**Published:** 2022-02-14

**Authors:** Milena Masullo, Gianluigi Lauro, Antonietta Cerulli, Giuseppe Bifulco, Sonia Piacente

**Affiliations:** Dipartimento di Farmacia, Università degli Studi di Salerno, Fisciano, Italy

**Keywords:** *Corylus avellana*, diarylheptanoid, α-glucosidase, molecular docking, eco-friendly extraction

## Abstract

*Corylus avellana* hard shells, green leafy involucres, leaves, and male flowers have shown to be a source of diarylheptanoids, a class of natural products with promising biological activities. Cyclic diarylheptanoids, named giffonins, were isolated from the Italian cultivar “Tonda di Giffoni.” Even if many efforts have been made to establish the chemistry of these compounds, little is known about their biological properties. Herein, the inhibitory effects of diarylheptanoids isolated from *C. avellana* byproducts against α-glucosidase enzyme were evaluated. Molecular docking experiments disclosed the establishment of several key interactions between all the screened diarylheptanoids and the protein counterpart, whose model was built through homology modeling procedure, thus rationalizing the detected inhibitory activities. Specifically, the most active compounds giffonin J (**10**), K (**11**), and P (**16**) were able to make both H-bonds and π–π stacking contacts with different residues belonging to the binding site responsible for the catalytic activity of the investigated enzyme. To highlight the occurrence of the bioactive diarylheptanoids in the extracts of *C. avellana* byproducts obtained by eco-friendly extractions, their LC-MS profiles were analyzed. LC-MS analysis showed how giffonin J (**10**), K (**11**), and P (**16**) occurred in the ethanol extract of the leaves, while in the extracts of shells and green leafy involucres only giffonin P (**16**) was evident. Moreover, the quantitative analysis of giffonin J (**10**), K (**11**), and P (**16**) in *C. avellana* byproducts was carried out by an analytical approach based on LC–ESI/QTrap/MS, using the Multiple Reaction Monitoring (MRM) experiment. These results prompt to evaluate *C. avellana* byproducts, especially the leaves, as a prospective source of bioactive diarylheptanoids for the development of functional ingredients for the treatment of diabetes.

## Introduction

*Corylus avellana* L. (Betulaceae), commonly known as hazelnut, represents one of the world’s most famous tree nuts. The nutritional properties of the kernel are mainly due to lipids showing beneficial biological activity. The kernels are usually eaten roasted and are widely employed in the confectionery industry to prepare chocolate, cream, cakes, and other bakery products. The Protected Geographical Indication (PGI) is a European mark attributed to *C. avellana*, cultivar “Tonda di Giffoni,” which guarantees the quality and reputation to safeguard this agricultural product. The hazelnut “Tonda di Giffoni” is characterized by particular features, physicochemical quality, and organoleptic properties, which can be brought back to its origin (Cerulli et al., 2018b; Bottone et al., 2019).

Our previous investigations focused the attention on the leaves, flowers, shells, green leafy involucres, and kernel of *C. avellana* cultivar “Tonda di Giffoni,” leading to the isolation of natural compounds belonging to the class of flavonoids, caffeic acids, and diarylheptanoids (Masullo et al., 2015a,b, 2016, 2017; Cerulli et al., 2017, 2018a).

Diarylheptanoids are molecules made up of two phenolic rings linked by a seven carbons chain. A first classification gathers them in linear or cyclic diarylheptanoids. For the latter group, the two phenolic rings of the linear diarylheptanoids can couple by C-C linkage or by C-O linkage, generating the meta, meta-bridged biaryls, or bridged diaryl ethers, respectively, also known as cyclic diarylheptanoids and cyclic diaryletherheptanoids. In our previous investigation, cyclic diarylheptanoids and cyclic diaryletherheptanoids isolated from the Italian cultivar “Tonda di Giffoni” were named giffonins.

The antioxidant activity of some giffonins was evaluated. When tested for their antioxidant ability by TBARS assay, giffonins A–I and K–U caused a lipid peroxidation inhibition at least comparable to curcumin, used as a reference compound (Masullo et al., 2015a,b, 2016, 2021). This assay revealed how giffonins D and H, tested at a concentration of 10 μM, were able to reduce H_2_O_2_–and H_2_O_2_/Fe^2+^-induced lipid peroxidation by more than 60 and 50%, respectively (Masullo et al., 2015b). Based on these results, giffonins showing the highest inhibitory activity were further investigated: giffonins K and G at 10 μM resulted in the most active compounds to reduce the oxidation of thiol groups and carbonylation in plasma proteins (Cerulli et al., 2021; Masullo et al., 2021). Some diarylheptanoids from *C. avellana* were evaluated for the antimicrobial activity against the Gram-positive strains *Bacillus cereus* and *Staphylococcus aureus* and the Gram-negative strains *Escherichia coli* and *Pseudomonas aeruginosa*. Giffonin U and carpinontriol B at a 40 μg/disk concentration caused zones of inhibition completely comparable to those obtained with tetracycline, used as the positive control (Cerulli et al., 2017).

Considering the previously cited works, even if many efforts were made to establish the chemistry of these compounds, deeper insight into the biological properties of these molecules should be provided. Diarylheptanoids gain an emerging interest due to their considerable biological activities, including anti-inflammatory, pro-apoptotic, anti-influenza, anti-emetic, and estrogenic actions (Jahng and Park, 2018). Along with these activities, they also showed to possess α-glucosidase inhibitory activity; this is the case of the linear diarylheptanoids amomutsaokols A–K, isolated from the fruits of *Amomum tsao-ko* (He et al., 2020) and the cyclic diarylheptanoids from *Alnus sieboldiana* (Chiba et al., 2013).

Type 2 diabetes mellitus is a metabolic disorder in which the body does not produce enough insulin or does not respond to it correctly. In this case, individuals with type 2 diabetes mellitus develop hyperglycemia (Brown et al., 2017). One of the strategies for treating this disorder consists in the inhibition of the enzymes that hydrolyze the carbohydrate; in this way, starch molecules are not metabolized to glucose molecules. Enzymes involved in this process are represented by salivary and pancreatic α-amylases, responsible for breaking dietary carbohydrates in oligosaccharides and α-glucosidases which, in the small intestine, metabolize oligosaccharides in single glucose molecules. Therefore, the inhibition of the α-glucosidase enzyme represents an interesting strategy in the treatment of diabetes, since it can retard the uptake of dietary carbohydrates and reduces post-prandial hyperglycemia (Brown et al., 2017).

Herein, the inhibitory effects of diarylheptanoids isolated from *C. avellana* byproducts against α-glucosidase enzyme were evaluated. Molecular docking experiments were performed for a small library of giffonins to shed light on the molecular basis behind their inhibitory activity against the enzyme.

To explore the potential use of *C. avellana* byproducts as a source of these biologically active compounds, leaves, male flowers, shells, and green leafy involucres were submitted to extraction by “eco-friendly” procedure using ethanol as extraction solvent. To compare the ethanol extracts of different parts of *C. avellana* for their content in diarylheptanoids, HPLC–electrospray ionization (ESI)–Orbitrap MS analysis was performed. This is the first report on the analysis of ethanol extracts of *C. avellana* byproducts. The quantitative analysis of the molecules showing the highest activity was performed by LC–ESI/QTrap/MS, using the Multiple Reaction Monitoring (MRM) mode.

This study provides the first evidence for the valorization and utilization of *C. avellana* byproducts as a source of bioactive compounds with potential anti-diabetic properties.

## Materials and Methods

### General Experimental Procedures

NMR spectroscopic data were acquired in MeOH-*d*_4_ (99.95%, Sigma–Aldrich, Milan, Italy) on a Bruker DRX-600 spectrometer (Bruker BioSpin GmBH, Rheinstetten, Germany) equipped with a Bruker 5 mm TCI CryoProbe at 300 K. The frequency for ^1^H and ^13^C were 600 and 150 MHz, respectively. Spectra were referenced using the solvent signal at 3.34 ppm as the chemical shift standard, obtaining good peak alignment. Identification of metabolites was achieved using chemical shifts (ppm). The ^1^H NMR spectra were collected with a relaxation delay of 1 s, 64 K data points, and a spectral width of 8 ppm. 2D-NMR experiments, including Double Quantum Filter Homonuclear Correlation Spectroscopy (DQF-COSY), Heteronuclear Single-Quantum Correlation Spectroscopy (HSQC), and Heteronuclear Multiple Bond Correlation (HMBC) were implemented permitting the assignment of the existing metabolites. HSQC was obtained with a spectral width of 10 and 160 ppm in the proton and carbon dimensions, respectively, 2 K data points, and a recycle delay of 1 s. HMBC was obtained with a spectral width of 10 and 240 ppm in the proton and carbon dimensions, respectively, 4 K data points, and a recycle delay of 1 s. The COSY was acquired with a spectral width of 10 ppm in both dimensions and 1 K data points. Data processing was carried out with Topspin 3.2 software.

Column chromatography was performed over Sephadex LH-20. HPLC separations were carried out on a Waters 590 system equipped with a Waters R401 refractive index detector, a Waters XTerra Prep MSC18 column (300 mm × 7.8 mm i.d.), and a Rheodyne injector. HRESIMS were carried out on a Thermo Scientific liquid chromatography system constituted of a quaternary Accela 600 pump and an Accela autosampler, connected to a linear trap-Orbitrap hybrid mass spectrometer (LTQ-Orbitrap XL, Thermo Fisher Scientific, Bremen, Germany) with ESI. The α-glucosidase assay was determined with a microplate spectrophotometer (Thermofisher) equipped with a 620 nm filter.

### Plant Material

The leaves, male flowers, shells, and green leafy involucres of *C. avellana* L., cultivar “Tonda di Giffoni” were collected at Giffoni, Salerno, Italy, and identified by Prof. V. De Feo (Department of Pharmacy, University of Salerno, Fisciano, Italy). In particular, the male flowers and the leaves were collected in January and November 2018, respectively; the green leafy involucres and the shells were collected in August 2018.

### Extraction, Isolation, and Identification

The leaves, male flowers, shells as well as green leafy involucres of *C. avellana* were dried in the oven at 40°C and extracted at room temperature using EtOH (3 days, three times). After filtration and evaporation of the solvent to dryness *in vacuo*, the dried EtOH extract (3 g) of leaves was fractionated on a Sephadex LH-20 (Pharmacia) column (100 mm × 5 cm), using MeOH as mobile phase, affording 86 fractions (8 mL), monitored by TLC. Further purifications were carried out by C18 RP semipreparative HPLC using MeOH-H_2_O as mobile phase and a flow rate of 2.5 mL/min.

Fractions 28–30 (51.0 mg) were chromatographed by semi-preparative HPLC using MeOH-H_2_O (3:2) as mobile phase (flow rate 2.5 mL/min) to yield giffonin J (**10**) (1.6 mg, *t*_*R*_ = 9.4 min) and giffonin K (**11**) (2.5 mg, *t*_*R*_ = 10.5 min). Fractions 31–33 (53.7 mg) were chromatographed by semipreparative HPLC using MeOH-H_2_O (3:2) as mobile phase to yield giffonin B (**2**) (1.5 mg, *t*_*R*_ = 12.0 min), giffonin F (**6**) (1.7 mg, *t*_*R*_ = 20.5 min), giffonin C (**3**) (2.4 mg, *t*_*R*_ = 30.6 min), alnusone (**21**) (2.3 mg, *t*_*R*_ = 36.2 min), and giffonin E (**5**) (1.2 mg, *t*_*R*_ = 53.1 min). Fractions 35–38 (78.2 mg) were chromatographed by semipreparative HPLC using MeOH-H_2_O (13:7) as mobile phase to yield giffonin H (**8**) (2.2 mg, *t*_*R*_ = 19.8 min), giffonin A (**1**) (2.1 mg, *t*_*R*_ = 20.3 min), giffonin G (**7**) (3.2 mg, *t*_*R*_ = 21.7 min), giffonin D (**4**) (4.0 mg, *t*_*R*_ = 22.3 min), and giffonin V (**25**) (1.5 mg, *t*_*R*_ = 25.6 min). Fractions 39–40 (26.1 mg) were chromatographed by semipreparative HPLC using MeOH-H_2_O (9:11) as mobile phase to yield giffonin I (**9**) (1.4 mg, *t*_*R*_ = 21.0 min). Fractions 41–44 (29.5 mg) were chromatographed by semi-preparative HPLC using MeOH-H_2_O (2:3) as mobile phase to yield giffonin N (**14**) (1.8 mg, *t*_*R*_ = 19.3 min). Fractions 45–48 (39.6 mg) were chromatographed by semi-preparative HPLC using MeOH-H_2_O (2:3) as mobile phase to yield giffonin P (**16**) (3.1 mg, *t*_*R*_ = 10.2 min), giffonin L (**12**) (2.3 mg, *t*_*R*_ = 20.0 min), giffonin O (**15**) (1.3 mg, *t*_*R*_ = 20.4 min), and giffonin M (**13**) (1.6 mg, *t*_*R*_ = 22.2 min). Fractions 49–51 (39.5 mg) were chromatographed by semipreparative HPLC using MeOH-H_2_O (2:3) as mobile phase (flow rate 2.5 mL/min) to yield giffonin T (**22**) (1.2 mg, *t*_*R*_ = 7.6 min), giffonin U (**23**) (3.1 mg, *t*_*R*_ = 10.5 min), and carpinontriol B (**24**) (3.8 mg, *t*_*R*_ = 21.7 min). Fractions 53–54 (25.3 mg) were chromatographed by semipreparative HPLC using MeOH-H_2_O (3:2) as mobile phase to obtain oregonin (**17**) (2.0 mg, *t*_*R*_ = 13.3 min).

To isolate giffonins Q, R, and S (**18**–**20**), dried EtOH extract (3 g) of male flowers was fractionated on a Sephadex LH-20, affording 55 fractions (8 mL), monitored by TLC. Fractions 18–20 (16.8 mg) were chromatographed by semipreparative HPLC using MeOH-H_2_O (2:3) as mobile phase (flow rate 2.5 mL/min) to yield giffonin Q (**18**) (1.6 mg, *t*_*R*_ = 35.6 min), giffonin R (**19**) (1.1 mg, *t*_*R*_ = 56.3 min), and giffonin S (**20**) (1.3 mg, *t*_*R*_ = 61.8 min). Subsequently, isolated diarylheptanoids were unambiguously elucidated by 1D- (^1^H and ^13^C) and 2D- (HSQC, HMBC, and COSY) NMR experiments (Supplementary Figures 1–25 and Supplementary Tables 1–3). The purity of these compounds (>99%) was determined by HPLC analysis.

### α-Glucosidase Inhibition Assay

α-Glucosidase inhibitory activity was evaluated as previously reported (Kilinc et al., 2020). Briefly, 0.1 M phosphate buffer at pH 7.0 (150 μL) was added to each well; successively 10 μL of samples, dissolved in MeOH at different concentrations (200, 300, and 400 μM) were added; at this point, the reaction was initiated by the addition of 15 μL of the α-glucosidase enzyme (from *Saccharomyces cerevisiae*) (2 U/mL) in each well, and the plate was incubated for 5 min at 37°C; after 5 min 75 μL of the substrate (2.5 mM) 4-nitrophenyl α-D-glucopyranoside were added, and successively the plate was incubated for 10 min at 37°C. The absorbance was measured at 405 nm before (T0′) and after the incubation with the enzyme (T10′). The positive control was acarbose. Negative control absorbance (phosphate buffer in place of the sample) was also recorded. Inhibition of the enzyme was calculated, and the results were expressed as IC_50_ values. All experiments were carried out in triplicate. IC_50_ values were calculated through non-linear regression using Graphpad Prism 5 Software and expressed as means ± SD (standard deviation). They were considered statistically significant with values of *p* < 0.05.

### Molecular Modeling

To be consistent with the biological assays performed (see section “α-Glucosidase Inhibition Assay”), the 3D protein model of α-glucosidase from *Saccharomyces cerevisiae* was built through homology modeling procedure due to the absence of any solved structure in the Protein Data Bank (PDB). The crystal structure of isomaltase (oligo 1,6-glucosidase) from *S. cerevisiae* available in the (PDB code: 3A47)^1^ was used as a template because it showed high similarity and the highest amino acid sequence identity [71.89%, as checked with Basic Local Alignment Search Tool (BLAST), (Altschul et al., 1990; McGinnis and Madden, 2004)]^2^ with α-glucosidase. The protein model was generated using Prime software (Jacobson et al., 2004; Glide, 2021); afterward, the quality of the generated model was assessed through PROCHECK (Supplementary Figure 26; Morris et al., 1992; Laskowski et al., 1993).

On the built 3D protein model of α-glucosidase (Supplementary Table 8) from *S. cerevisiae* the putative binding sites were identified through SiteMap software (Halgren, 2007, 2009; Glide, 2021), generating a ranking of five possible druggable binding pockets based on the SiteScore parameter (Supplementary Table 9). The best-ranked one was accounted for the subsequent molecular docking experiments (Supplementary Tables 8, 9 and see sections “Results” and “Discussion”). Specifically, starting from Site 1 (Supplementary Table 9) the following coordinates: 30.13 (x), –1.39 (y), 7.32 (z), 10 × 10 × 10 as innerbox, and 30 × 30 × 30 as outerbox were accounted for Glide experiments (*vide infra*).

The library of investigated diarylheptanoids (see sections “Results” and “Discussion”) was prepared using LigPrep software (Schrodinger Suite) (Glide, 2021), generating all the possible tautomers and protonation states (pH = 7.4 ± 1.0) for each compound. The obtained structures were minimized using the OPLS 2005 force field.

Molecular docking experiments were performed using Glide software (Schrödinger Suite) (Friesner et al., 2004, 2006; Halgren et al., 2004; Glide, 2021), using the Extra Precision (XP) mode. In detail, 10,000 poses were kept in the starting phase of docking for energy minimization, setting the scoring window for keeping the initial poses to 400.0 and a scaling factor of 0.8 related to van der Waals radii, with a partial charge cutoff of 0.15, based on a 0.5 kcal/mol rejection cutoff for the obtained minimized poses. Eventually, 10 maximum number of poses for each compound were saved in the output file.

### LC–ESI/(HR)Orbitrap/MS Analysis

The qualitative profiles of the EtOH extract of *C. avellana* leaves, flowers, shells, and green leafy involucres were obtained by HPLC coupled to multiple-stage linear ion-trap and orbitrap high-resolution MS (LC–ESI/LTQOrbitrap/MS/MS^n^) (Supplementary Tables 4–6). LC-MS analysis was carried out on Kinetex C18 2.6 μm (100 mm × 2.10 mm) column (Phenomenex, Aschaffenburg, Germany), using a flow rate of 0.2 mL/min and a mobile phase consisting of a combination of A (0.1% formic acid in water, v/v) and B (0.1% formic acid in acetonitrile, v/v); a linear gradient was set to 5% B at 0 min, remained for 5 min at 5% B and then increased to 95% B from 5 to 50 min, holding it for 5 min, before returning to the initial percentage.

The mass spectrometer operated in negative ion mode. ESI source parameters were as follows: capillary voltage –48 V; tube lens voltage –176.47; capillary temperature 280°C; sheath and auxiliary gas flow (N_2_), 15 and 5, respectively; sweep gas 0; spray voltage 5. MS spectra were acquired by full range acquisition covering *m/z* 180–1,600 at a resolution of 30,000. For the data-dependent scan, the first and the second most intense ions from the HRMS scan event were selected to offer their tandem mass (MS/MS) product ions with a normalization collision energy at 30%, a minimum signal threshold at 250, and an isolation width at 2.0; multiple-stage tandem mass (MS^n^ with *n* = 3, 4…) experiments on selected product ions were carried out by the same collision. The molecular formulae were assigned with an accuracy inferior to 6 ppm for all the compounds.

### Quantitative Analysis by LC–ESI/QTrap/MS/MS Analysis and Calibration Curves

Quantitative analysis was performed on an LC–ESI/QTrap/MS system, using the MRM mode, operating with a Luna Omega Polar 1.6 μm RP C18 column (Phenomenex, 50 mm × 2.1 mm) kept at 30°C, at a flow rate of 0.30 mL/min; mobile phases consisting of 0.1% HCOOH in water (v/v) and 0.1% HCOOH in acetonitrile (v/v) were used as phase A and B, respectively. A linear gradient from 5 to 95% B in 7.20 min, held to 95% B for 40 s, and from 95 to 5% in 1 min, was used. *C. avellana* extracts were diluted by using MeOH, and the autosampler was set to inject 4 μL of extract (1.00 mg/mL) in triplicate.

Stock solutions (l mg/mL) of the external standards (ES) **10**, **11,** and **16** were prepared by dissolving compounds in a solution of MeOH. Stock solutions were diluted with appropriate amounts of MeOH to give solutions containing 0.001, 0.01, 0.05, 0.1, 0.5, 1.0, and 5.0 μg/mL of each standard. To each standard solution, an appropriate amount of internal standard (IS; quercetin) was added to yield a final concentration of 5.0 μg/mL. The instrument operated in the negative ion mode with declustering potential, entrance potential, collision energy, and collision cell exit potential optimized for internal and external standards (Supplementary Table 7).

To validate the LC–ESI/QTrap/MS/MS method, precision (at seven concentrations), for each compound through triplicate intra-day assays and inter-day assays over 3 days was determined. Specificity was defined as the non-interference by other analytes detected in the region of interest. Linearity was evaluated by correlation values of calibration curves; limit of detection (LOD), and limit of quantification (LOQ) were evaluated according to previously described procedures (Masullo et al., 2015c; Cerulli et al., 2021). The LOD for each analyte varied from 0.008 to 0.018 μg/mL and LOQ from 0.024 to 0.059 μg/mL.

## Results

### Extraction and Isolation

The leaves, male flowers, shells, and green leafy involucres of *C. avellana* were dried and extracted with ethanol. The extracts were fractionated on a Sephadex column, and successively the obtained fractions were further purified by HPLC analysis. The isolated compounds **1**–**25** (Figure 1) were identified by analysis of their spectroscopic NMR (^1^H and ^13^C NMR, HSQC, HMBC, and COSY experiments) along with ESIMS and HRMS analysis (Supplementary Figures 1–25 and Supplementary Tables 1–3).

**FIGURE 1:**
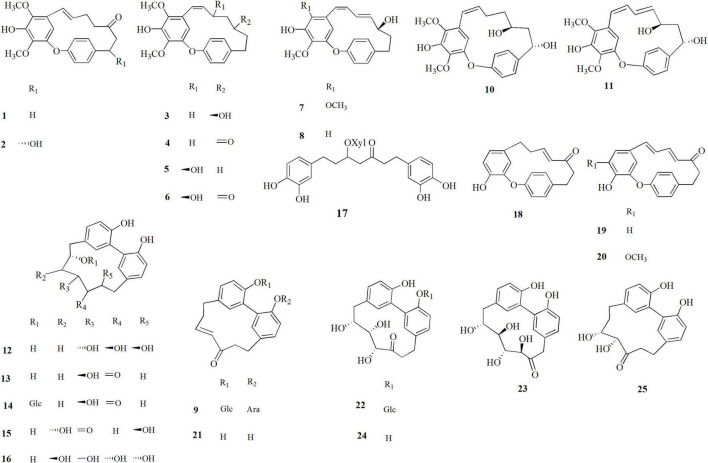
Diarylheptanoids isolated from *Corylus avellana*, cultivar Giffoni, byproducts.

### α-Glucosidase Activity of Diarylheptanoids

The inhibitory effects of the diarylheptanoids isolated from *C. avellana* byproducts against α-glucosidase in comparison with the antidiabetic acarbose used as reference compound were evaluated; the α-glucosidase enzyme is involved in the carbohydrate metabolism and the release of glucose. Therefore, its inhibition is an effective strategy of delaying glucose absorption and lowering the post-prandial blood glucose level, which can potentially suppress the progression of diabetes mellitus (Kumar et al., 2011).

Diarylheptanoids isolated from *C. avellana* “Tonda di Giffoni” byproducts and positive control (acarbose) were evaluated in the concentration range 200–400 μM (Table 1). Giffonins J, K, and P showed significant activity with IC_50_ values ranging from 55.3 to 70.0 μM and were more active than the positive control, acarbose (IC_50_ = 115.1 μM). Giffonins C, D, G–I, M, and carpinontriol B exhibited moderate activity with IC_50_ values ranging from 104.6 to 113.9 μM, comparable to acarbose, whereas the other compounds were less active.

**TABLE 1:** α-Glucosidase inhibitory activity of diarylheptanoids isolated by *Corylus avellana*, cultivar Giffoni, byproducts.

Compound	IC_50_ ± SD (μM)
Giffonin A **(1)**	184.1 ± 6.9
Giffonin B **(2)**	163.6 ± 5.2
Giffonin C **(3)**	104.7 ± 6.9
Giffonin D **(4)**	108.0 ± 6.1
Giffonin E **(5)**	167.1 ± 4.6
Giffonin F **(6)**	146.5 ± 4.3
Giffonin G **(7)**	108.1 ± 9.2
Giffonin H **(8)**	108.5 ± 2.9
Giffonin I **(9)**	104.6 ± 4.9
Giffonin J **(10)**	56.6 ± 9.6
Giffonin K **(11)**	70.0 ± 3.1
Giffonin L **(12)**	171.8 ± 8.9
Giffonin M **(13)**	113.9 ± 9.7
Giffonin N **(14)**	128.3 ± 11.3
Giffonin O **(15)**	123.9 ± 11.1
Giffonin P **(16)**	55.3 ± 7.7
Oregonin **(17)**	143.9 ± 7.2
Giffonin Q **(18)**	126.6 ± 5.9
Giffonin R **(19)**	128.5 ± 6.1
Giffonin S **(20)**	126.1 ± 8.9
Alnusone **(21)**	154.1 ± 8.3
Giffonin T **(22)**	130.9 ± 6.1
Giffonin U **(23)**	175.3 ± 9.8
Carpinontriol B **(24)**	106.8 ± 6.1
Giffonin V **(25)**	122.6 ± 4.4
Acarbose	115.1 ± 2.2

*Data are expressed as the mean values ± SD of three experiments.*

### Molecular Docking Experiments

Since the inhibitory effects of the investigated diarylheptanoids were evaluated *in vitro* against α-glucosidase from *S. cerevisiae* and due to the absence of the related solved structure of this protein, the related 3D structure for the subsequent molecular docking was first built through homology modeling procedure (see section “Materials and Methods”) (Jacobson et al., 2004; Prime, 2021). The binding site featuring the highest score (SiteScore value from SiteMap software) (Halgren, 2007, 2009; SiteMap, 2021) corresponded to the protein region already investigated in previous studies (Ma et al., 2015; Jabeen et al., 2016; Milella et al., 2016; Wang et al., 2017) and recognized as that responsible for the catalytic activity of this enzyme.

**FIGURE 2:**
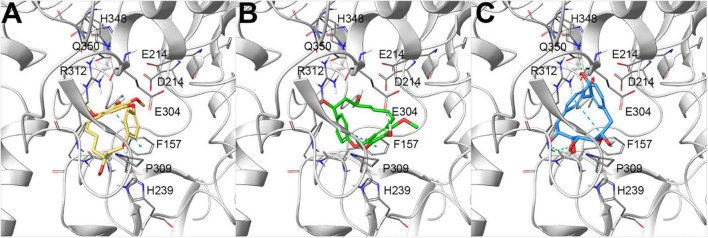
Docking poses of **(A)** giffonin J **(10)** (colored by atom type: C yellow, O red, polar H light gray), **(B)** K **(11)** (colored by atom type: C green, O red, polar H light gray), and **(C)** P **(16)** (colored by atom type: C light blue, O red, polar H light gray), showing the most promising inhibitory activity against α-glucosidase from *Saccharomyces cerevisiae*, built by homology modeling (represented with light gray ribbons; key residues are reported as sticks and colored by atom type: C gray, O red, N blue, S yellow, polar H light gray). H-bond and π–π stacking interactions are represented with dotted green and light blue lines, respectively.

To rationalize the biological outcomes for the 25 screened diarylheptanoids, output poses from molecular docking experiments (Friesner et al., 2004, 2006; Halgren et al., 2004; Glide, 2021) were then carefully analyzed, selecting those that showed a set of interactions with key residues on the investigated protein and identified in previous studies as responsible for the observed biological activity (Ma et al., 2015; Jabeen et al., 2016; Milella et al., 2016; Wang et al., 2017). In particular, we first checked whether the investigated compounds were able to conveniently interact with the key residues involved in the catalytic activity (D214, E276, and D349, corresponding to D215, E277, and D352 of isomaltase, respectively) (Yamamoto et al., 2010; Jenis et al., 2019). All the investigated compounds were able to establish contacts with these residues, thus representing promising items, as confirmed by the biological outcomes (Table 1). Focusing on the most active compounds giffonin J (**10**), K (**11**), and P (**16**) (Table 1), the careful analysis of the docking poses highlighted a network of interactions with a large number of residues (F157, F158, H239, E304, P309, R312, and Q350) (Jenis et al., 2019). In particular, all these compounds were able to establish different contacts with F157, D214, and an edge-to-face π–π stacking with F157 (Jabeen et al., 2016); moreover, H bonds were detected with E304 (for compound **11**), F157, and Q350 (for compound **16**) (Figure 2).

### LC-MS Analysis of Secondary Metabolites in *Corylus avellana* “Green” Extracts of Leaves, Male Flowers, Shells, and Green Leafy Involucres

**FIGURE 3:**
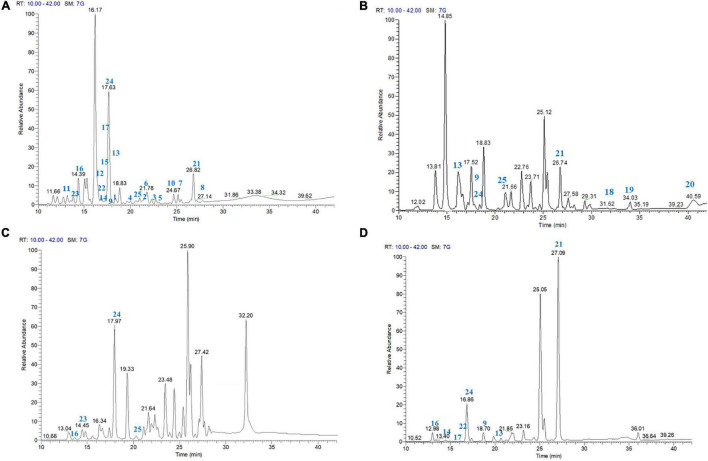
LC–ESI/LTQOrbitrap/MS/MS^n^ profile (negative-ion mode) of the EtOH extract of *C. avellana*, cultivar Giffoni, leaves **(A)**, male flowers **(B)**, shells **(C)**, and green leafy involucres **(D)**.

To define the occurrence of the most active diarylheptanoids in the leaves, male flowers, shells, as well as green leafy involucres of *C. avellana*, these byproducts were submitted to eco-friendly extraction using ethanol as extraction solvent. The metabolite profiling of the ethanol extracts of the different parts of *C. avellana* was successfully obtained by HPLC–ESI–Orbitrap MS analysis in negative ionization mode (Figure 3). In particular, LC–(-)ESI/HRMS analyses were performed using the “data-dependent scan” mode. The MS software selected precursor ions corresponding to the most intense peaks in the LC-MS spectrum.

The careful analysis of accurate masses, characteristic fragmentation patterns, chromatographic behavior, and literature data allowed us to putatively ascertain the occurrence of phenolic derivatives mainly belonging to diarylheptanoid, flavonoid, and neolignan classes (Table 1 and Supplementary Tables 4–6). According to our previous investigations (Masullo et al., 2016, 2017; Napolitano et al., 2018), compounds **1**–**25** could be promptly identified as diarylheptanoid derivatives. The structural information useful to define the chemistry of diarylheptanoids was obtained by analyzing neutral losses generated in their fragmentation. Typical fragmentation patterns mainly involved the hydroxyl and carbonyl groups located on the heptanoid chain and were characterized by the opening of the diarylheptanoid cycle after the dienone–phenol rearrangement of the [M-H]^–^ pseudomolecular ion. In detail, loss of 18 Da suggested the presence of a hydroxyl group on the heptane chain instead of on the aromatic rings. The elimination of 16 Da indicated the hydroxyl group on the aromatic rings, while the loss of 15 Da suggested the presence of the methoxy group. By neutral losses produced during collision-induced dissociation, the saccharide moieties on the diarylheptanoid could be assumed. If the compound was glycosylated through a hydroxyl group on the C7 chain, the neutral loss of 180 Da referred to a hexose residue (giffonin N). Nevertheless, when the sugar residues were attached to a hydroxyl group on the aromatic rings (e.g., giffonins I and T), the loss of 162 Da corresponding to the hexose moiety was observed.

Furthermore, the LC–(-)ESI/HRMS profiles showed some peaks (**27**–**35**) which, based on their molecular formula and fragmentation pattern, could be identified as glycosylated flavonoids with one or two sugar units (Masullo et al., 2016), and myricetin, quercetin, and kaempferol as aglycone. In particular, tandem mass spectra of **27**–**32** and **35** allowed to assign them as mono-glycosylated flavonoids and gave information about the nature (pentose or hexose) of the sugar linked to the aglycons. In detail, the MS/MS spectra of compounds **27** and **35** showed the main product ion derived by neutral loss of 162 Da ascribable to a hexose unit. Instead, compounds **28**–**32** were characterized by main product ions originated by the neutral loss of 146 Da, corresponding to a deoxyhexose unit. In the same way, two di-glycosylated flavonoids (**33** and **34**) were assigned. Moreover, by LC–(-)ESI/HRMS, the occurrence of chlorogenic acid (**26**) could be defined.

Diarylheptanoids and glycosylated flavonoids characterized EtOH extracts of leaves, flowers, shells, and green leafy involucres (Table 2 and Supplementary Tables 4–6). In contrast, only the EtOH extract of shells showed some peaks (**38**–**47**) whose molecular formula and the resulting fragmentation patterns were typical of compounds belonging to the class of neolignans (Masullo et al., 2017).

**TABLE 2:** Retention times (R_*t*_), Δ ppm, molecular formula, [M-H]^–^, and MS/MS values of compounds occurring in the EtOH extract of *Corylus avellana* leaves, cultivar “Tonda di Giffoni PGI,” identified by high-resolution LC–ESI/LTQOrbitrap/MS/MS^n^ (negative ion mode).

	R_*t*_ (min)	Compound	Δ ppm	Formula	[M-H]^–^	MS/MS
**26**	12.02	Chlorogenic acid	0.66	C_16_H_18_O_9_	353.0869	191
**11**	13.02	Giffonin K	–5.94	C_21_H_22_O_6_	369.1311	351
**23**	13.81	Giffonin U	0.79	C_19_H_20_O_7_	359.1130	341, 329
**16**	14.39	Giffonin P	1.66	C_19_H_22_O_7_	361.1288	343, 301, 271, 241
**27**	15.33	Myricetin *O*-β-D-glucopyranoside	–0.92	C_21_H_20_O_13_	479.0816	316
**28**	16.17	Myricetin 3-*O*-α-L-rhamnopyranoside	1.12	C_21_H_20_O_12_	463.0876	316
**12**	16.27	Giffonin L	1.35	C_19_H_22_O_6_	345.1337	323, 311
**17**	16.27	Oregonin	–0.03	C_24_H_30_O_10_	477.1755	327
**22**	16.48	Giffonin T	0.18	C_25_H_30_O_11_	505.1705	343, 325, 299
**15**	16.84	Giffonin O	1.15	C_19_H_20_O_6_	343.1180	299, 283, 269
**14**	16.84	Giffonin N	0.40	C_25_H_30_O_10_	489.1757	309,281
**9**	17.52	Giffonin I	–2.03	C_30_H_36_O_12_	587.2435	455, 425, 293
**29**	17.60	Quercetin 3-*O*-α-L-rhamnopyranoside	1.03	C_21_H_20_O_11_	447.0926	301
**24**	17.63	Carpinontriol B	2.23	C_19_H_20_O_6_	343.1184	325, 313, 299, 283, 269
**1**	18.03	Giffonin A	0.65	C_21_H_22_O_5_	353.1386	333, 263
**13**	18.52	Giffonin M	2.26	C_19_H_20_O_5_	327.1234	253, 205, 179
**30**	18.83	Kaempferol 3-*O*-α-L-rhamnopyranoside	0.90	C_21_H_20_O_10_	431.0978	285
**4**	19.70	Giffonin D	1.24	C_21_H_22_O_5_	353.1388	333, 263
**25**	20.57	Giffonin V	1.80	C_18_H_32_O_5_	327.1233	205, 179, 147, 121
**2**	20.68	Giffonin B	0.93	C_21_H_22_O_6_	369.1336	351
**6**	21.78	Giffonin F	1.26	C_21_H_22_O_6_	369.1337	351
**3**	22.47	Giffonin C	–1.57	C_21_H_23_O_5_	355.1534	311
**5**	22.60	Giffonin E	2.98	C_21_H_23_O_5_	355.1550	311
**10**	24.10	Giffonin J	2.82	C_21_H_24_O_6_	371.1499	327, 339
**31**	25.14	Kaempferol 3-*O*-(4″-cis-*p*-coumaroyl)-α-L-rhamnopyranoside	0.19	C_30_H_26_O_12_	577.1342	431, 285
**7**	25.20	Giffonin G	1.26	C_21_H_22_O_5_	353.1389	333, 263
**32**	25.46	Kaempferol 3-*O*-(4″-trans-*p*-coumaroyl)-α-L-rhamnopyranoside	–0.25	C_30_H_26_O_12_	577.1339	431, 285
**21**	26.82	Alnusone	1.77	C_19_H_18_O_3_	293.1177	251, 83
**8**	27.14	Giffonin H	1.78	C_20_H_20_O_4_	323.1284	305
						

### Quantitative Analysis of Giffonins J (10), K (11), and P (16) in *Corylus avellana* Byproducts

An LC–ESI/QTrap/MS/MS analysis, using the MRM mode, was carried out for the most active compounds. In particular, MRM is a very high selective and sensitive tandem mass spectrometric technique. A specific transition from a precursor ion to a product ion is monitored for each compound. In this way, a quantitative determination of giffonin J (**10**), K (**11**), and P (**16**) has been performed (Table 3). The [M-H]^–^ pseudomolecular ions at *m/z* 371 and 369, corresponding to giffonin J (**10**) and giffonin K (**11**), respectively, showed a main [(M-18)-H]^–^ product ion at *m/z* 353 and 351, due to the neutral loss of water. The mass tandem spectrum of the [M-H]^–^ pseudomolecular ion at *m/z* 361 corresponding to giffonin P (**16**) displayed a very intense product ion at *m/z* 241 associated with the opening of the diarylheptanoid cycle after the dienone–phenol rearrangement.

**TABLE 3:** Quantitative results of giffonin J (**10**), K (**11**), and P (**16**) occurring in EtOH extract of *Corylus avellana* leaves, shells, and green leafy involucres.

Compound	MRM transition	*R* ^2^	Regression line	mg/100g leaves ± SD	mg/100g shells ± SD	mg/100g green leafy involucres ± SD
Giffonin J **(10)**	371→353	0.996	*y* = 3.64e^5^x + 0.0087	0.88 ± 0.08	ND*	ND*
Giffonin K **(11)**	369→351	0.997	*y* = 3.66 e^5^x + 0.00744	1.12 ± 0.24	ND*	ND*
Giffonin P **(16)**	361→241	0.993	*y* = 0.000171x + 0.0874	8.86 ± 0.89	0.17 ± 0.02	6.52 ± 0.51

*Not detected (ND) compounds.

The quantitative results displayed that giffonin P (**16**) occurred in all the extracts in a concentration ranging from 8.86 to 0.17 mg/100 g dry weight except for male flowers; in fact, the amount of giffonin P (**16**) in *C. avellana* flowers was found below the LOD of the applied method. Moreover, following LC–ESI/LTQOrbitrap/MS, which showed giffonin J (**10**) and K (**11**) in *C. avellana* leaves and not in the other byproducts, these two compounds were quantified only in the leaves in a concentration of 0.88 and 1.12 mg/100 g, respectively. In shells, male flowers, as well as in green leafy involucres, giffonin J (**10**) and K (**11**) were found below the LOD.

## Discussion

### Isolation of Diarylheptanoids From *Corylus avellana*

Previous investigations on *C. avellana*, cultivar “Tonda di Giffoni” highlighted the presence of natural diarylheptanoid derivatives. Diarylheptanoids are a class of natural compounds with a restricted distribution, found mainly in the plants of the Betulaceae family but so far isolated also in other eight families, such as Aceraceae, Actinidiaceae, Burseraceae, Casuarinaceae, Juglandaceae, Leguminosae, Myricaceae, and Zingiberaceae. The diarylheptanoids isolated from *Alnus*, *Betula*, *Corylus*, *Carpinus*, *Ostrya*, and *Ostruopsis* genus (Betulaceae family) are represented by both linear and cyclized forms (Jahng and Park, 2018). Noteworthy, the diarylheptanoids isolated from *C. avellana*, cultivar “Tonda di Giffoni,” named giffonins, are cyclized diarylheptanoids and diaryletherheptanoids, differing from the linear compounds reported in other *Corylus* species, and their potential in terms of biological activity is still unexplored.

### α-Glucosidase Activity and Molecular Docking Studies of Diarylheptanoids

Disorders in insulin production or insulin sensitivity and function cause one of the major chronic metabolic diseases, diabetes mellitus, characterized by a dysfunction in the balance of glucose homeostasis. So, hyperglycemia is inevitably correlated to diabetes (Sharma et al., 2021). One of the causes of morbidity and mortality in low- and middle-income countries is represented by diabetes mellitus, which is growing worldwide (Sharma et al., 2021). Data of the International Diabetes Federation (IDF) estimate that people (18–99 years) affected by diabetes mellitus are increasing to 693 million by 2045 (Sharma et al., 2021). For these reasons, the treatment of diabetes, mainly type-2 diabetes more than type-1 diabetes, gets big attention. Type 2 diabetes is considered an avoidable disorder, and its treatment focuses on the reversible inhibition of α-glucosidases. However, antidiabetic drugs have shown side effects when used for a long time, along with specific limitations; for example, acarbose, miglitol, and voglibose have shown unwanted gastrointestinal symptoms as adverse effects (Cakar et al., 2017; Čakar et al., 2018).

In this context, the search for new reversible inhibitors from natural sources is placed. Considering the α-glucosidase inhibitory activity shown by the linear diarylheptanoids amomutsaokols A–K, isolated from the fruits of *Amomum tsao-ko* (He et al., 2020) and by the cyclic diarylheptanoids found in *Alnus sieboldiana* (Chiba et al., 2013), giffonins along with the other diarylheptanoids occurring in *C. avellana* were evaluated as inhibitors of α-glucosidase.

Oregonin (**17**) is the only linear diarylheptanoid tested. Its IC_50_ value was found of 143.9 μM, higher with respect to acarbose (IC_50_ = 115.1 μM). Considering the cyclic diaryletherheptanoids, differences occurring in the aromatic ring seem not to influence the activity: giffonins G (**7**) and H (**8**) differed each other for a methoxy group and their IC_50_ values were comparable (108.1 and 108.5 μM, respectively). The same behavior was observed for giffonins R (**19**) and S (**20**) (128.5 and 126.1 μM, respectively). Among all tested compounds, two diaryletherheptanoids, giffonins J (**10**) and K (**11**), and a diarylheptanoid giffonin P (**16**), showed the highest inhibitory activity with IC_50_ values of 56.6, 70.0, and 55.3 μM, respectively.

Starting from the protein model built by homology modeling, molecular docking experiments highlighted the good accommodation of the investigated diarylheptanoids onto the ligand-binding site. Specifically, all the compounds were placed in a region responsible for the catalytic activity of this enzyme, establishing a network of polar, H-bond, hydrophobic, and π–π stacking interactions. Specifically, the analysis of the binding modes was focused on evaluating the ability of the investigated compounds to interact with the key residues responsible for the catalytic activity of α-glucosidase, namely, D214, E276, H348, and D349. Also, the careful analysis of the most active compounds (**10**, **11**, and **16**) disclosed a large pattern of H bond (F157, E304, and Q350) and π–π stacking (F157) contacts stabilizing the ligand–protein predicted complex and corroborating the biological outcomes.

### LC-MS Profiles of “Green” Extracts of *Corylus avellana* Byproducts and Quantification of Giffonins J (10), K (11), and P (16)

The choice of green solvents to replace the use of petrochemical solvents is growing, oriented mainly to ethanol. Ethanol is a solvent coming from the fermentation of sugar-rich material, with attractable features due to its high purity and low price. It is also biodegradable and easily available (Chemat et al., 2012). So far, the chemical profiles of ethanol extracts of *C. avellana* byproducts were not described. For this reason, LC-MS profiles of the extracts were performed to highlight the occurrence of diarylheptanoids in *C. avellana* byproducts and provide useful information on the choice of the byproduct. The accurate analysis of LC-(-)ESI/HRMS profiles highlighted how the leaves extract represents a source of cyclic diarylheptanoids richer than the other extracts (Figure 3) since in the leaves all diarylheptanoids tested were identified except giffonins Q–S (**18**–**20**). The LC-(-)ESI/HRMS analysis of the ethanol extracts showed the occurrence of giffonins Q–S (**18**–**20**), alnusone (**21**) along with giffonins I (**9**), M (**13**), V (**24**), and carpinontriol B (**24**) in the hazelnut flowers. Alnusone (**21**) and carpinontriol B (**24**) were also identified in the green leafy involucres extract along with giffonins I (**9**), M (**13**), N (**14**), P (**16**), and T (**22**). Giffonin V (**25**), along with giffonins P (**16**), U (**23**), and carpinontriol B (**24**) were found in the hazelnut shells. As evident from the LC-MS profiles (Figure 3), the ethanol extracts, besides diarylheptanoid compounds, were further characterized by the presence of flavonoids and neo-lignan derivatives (Table 1 and Supplementary Tables 4–6).

Focusing on the most active diarylheptanoids, giffonin J (**10**), K (**11**), and P (**16**), LC–MS analysis showed their presence in the ethanol extract of the leaves while in the extracts of shells and green leafy involucres only giffonin P (**16**) was evident. Their amount was also determined in the green extracts of *C. avellana* byproducts by an LC–ESI/QTrap/MS/MS method.

The highest amount of giffonin P (**16**) (8.86 mg/100 g leaves) was first observed in the leaves, then in the green leafy involucres (6.52 mg/100 g green leafy involucres), and finally in the shells (0.17 mg/100 g shells). The amounts of giffonin J (**10**) and K (**11**) were found comparable, with a value of 0.88 and 1.12 mg/100 g leaves, respectively. The amount of cited compounds in all the other byproducts was found below the LOD of the applied method.

This study confirms the potential benefits of *C. avellana* as a rich source of diarylheptanoid derivatives and extends the knowledge of their biological activity. Diarylheptanoids from hazelnut are inhibitors of the α-glucosidase enzyme, but further studies are required to address this point deeper. So, this work represents the first step to promote the use of hazelnut byproducts, in particular the leaves, as a source of functional ingredients for the treatment of diabetes disease.

## Data Availability Statement

The raw data supporting the conclusions of this article will be made available by the authors, without undue reservation.

## Author Contributions

SP and GB contributed toward conceptualization, writing, review and editing of the manuscript, supervision, project administration, and funding acquisition. AC, GL, and MM contributed to the methodology, data curation, formal analysis, and writing – original draft preparation. AC contributed to investigation. SP contributed to resources. All authors contributed to the article and approved the submitted version.

## Conflict of Interest

The authors declare that the research was conducted in the absence of any commercial or financial relationships that could be construed as a potential conflict of interest.

## Publisher’s Note

All claims expressed in this article are solely those of the authors and do not necessarily represent those of their affiliated organizations, or those of the publisher, the editors and the reviewers. Any product that may be evaluated in this article, or claim that may be made by its manufacturer, is not guaranteed or endorsed by the publisher.

## Footnotes

1
https://www.rcsb.org/structure/3a47

2
https://blast.ncbi.nlm.nih.gov/Blast.cgi
