# Supplementary material for: Corylus avellana: A Source of Diarylheptanoids With α-Glucosidase Inhibitory Activity Evaluated by in vitro and in silico Studies
Source: Front Plant Sci. 2022 Feb 14;13:805660. doi: 10.3389/fpls.2022.805660 (PMC8882611; doi:10.3389/fpls.2022.805660)
Supplement: Supplementary file 1 [file Data_Sheet_1.docx]

**Supplementary materials**

**Figure S1.** ^1^H NMR Spectrum (600 MHz, CD_3_OD) of giffonin A (**1**).

**Figure S2.** ^1^H NMR Spectrum (600 MHz, CD_3_OD) of giffonin B (**2**).

**Figure S3.** ^1^H NMR Spectrum (600 MHz, CD_3_OD) of giffonin C (**3**).

**Figure S4.** ^1^H NMR Spectrum (600 MHz, CD_3_OD) of giffonin D (**4**).

**Figure S5.** ^1^H NMR Spectrum (600 MHz, CD_3_OD) of giffonin E (**5**).

**Figure S6.** ^1^H NMR Spectrum (600 MHz, CD_3_OD) of giffonin F (**6**).

**Figure S7.** ^1^H NMR Spectrum (600 MHz, CD_3_OD) of giffonin G (**7**).

**Figure S8.** ^1^H NMR Spectrum (600 MHz, CD_3_OD) of giffonin H (**8**).

**Figure S9.** ^1^H NMR Spectrum (600 MHz, CD_3_OD) of giffonin I (**9**).

**Figure S10.** ^1^H NMR Spectrum (600 MHz, CD_3_OD) of giffonin J (**10**).

**Figure S11.** ^1^H NMR Spectrum (600 MHz, CD_3_OD) of giffonin K (**11**).

**Figure S12.** ^1^H NMR Spectrum (600 MHz, CD_3_OD) of giffonin L (**12**).

**Figure S13.** ^1^H NMR Spectrum (600 MHz, CD_3_OD) of giffonin M (**13**).

**Figure S14.** ^1^H NMR Spectrum (600 MHz, CD_3_OD) of giffonin N (**14**).

**Figure S15.** ^1^H NMR Spectrum (600 MHz, CD_3_OD) of giffonin O (**15**).

**Figure S16.** ^1^H NMR Spectrum (600 MHz, CD_3_OD) of giffonin P (**16**).

**Figure S17.** ^1^H NMR Spectrum (600 MHz, CD_3_OD) of oregonin (**17**).

**Figure S18.** ^1^H NMR Spectrum (600 MHz, CD_3_OD) of giffonin Q (**18**).

**Figure S19.** ^1^H NMR Spectrum (600 MHz, CD_3_OD) of giffonin R (**19**).

**Figure S20.** ^1^H NMR Spectrum (600 MHz, CD_3_OD) of giffonin S (**20**).

**Figure S21.** ^1^H NMR Spectrum (600 MHz, CD_3_OD) of alnusone (**21**).

**Figure S22.** ^1^H NMR Spectrum (600 MHz, CD_3_OD) of giffonin T (**22**).

**Figure S23.** ^1^H NMR Spectrum (600 MHz, CD_3_OD) of giffonin U (**23**).

**Figure S24.** ^1^H NMR Spectrum (600 MHz, CD_3_OD) of carpinontriol B (**24**).

**Figure S25.** ^1^H NMR Spectrum (600 MHz, CD_3_OD) of giffonin V (**25**).

**Figure S26.** Ramachandran plot

**Table S1.** ^13^C NMR Data (150 MHz, CD_3_OD, δ ppm) of diaryletherheptanoids **1-8, 10, 11, 18-20**

**Table S2.** ^13^C NMR Data (150 MHz, CD_3_OD, δ ppm) of diarylheptanoids **9, 12-16, 21-25**

**Table S3.** ^13^C NMR Data (150 MHz, CD_3_OD, δ ppm) of oregonin (**17**)

**Table S4.** Retention times (R_t_), Δ ppm, molecular formula, [M-H]^-^, MS/MS values, of compounds occurring in the EtOH extract of *C. avellana* flowers, cultivar “Tonda di Giffoni PGI”, identified by high resolution LC-ESI/LTQOrbitrap/MS/MS^n^ (negative ion mode).

**Table S5.** Retention times (R_t_), Δ ppm, molecular formula, [M-H]^-^, MS/MS values, of compounds occurring in the EtOH extract of *C. avellana* shells, cultivar “Tonda di Giffoni PGI”, identified by high resolution LC-ESI/LTQOrbitrap/MS/MS^n^ (negative ion mode).

**Table S6.** Retention times (R_t_), Δ ppm, molecular formula, [M-H]^-^, MS/MS values, of compounds occurring in the EtOH extract of *C. avellana* green leafy involucres, cultivar “Tonda di Giffoni PGI”, identified by high resolution LC-ESI/LTQOrbitrap/MS/MS^n^ (negative ion mode).

**Table S7.** LC–MS/MS conditions for quantitation of compounds **10, 11** and **16** by negative ion MRM mode.

**Table S8.** 3D coordinates of α-glucosidase protein structure built by homology modeling experiments.

**Table S9.** SiteScore from related to the five putative binding sites detected from SiteMap. Related coordinates are reported, referred to the protein structure built by homology modeling experiments (Table S8).


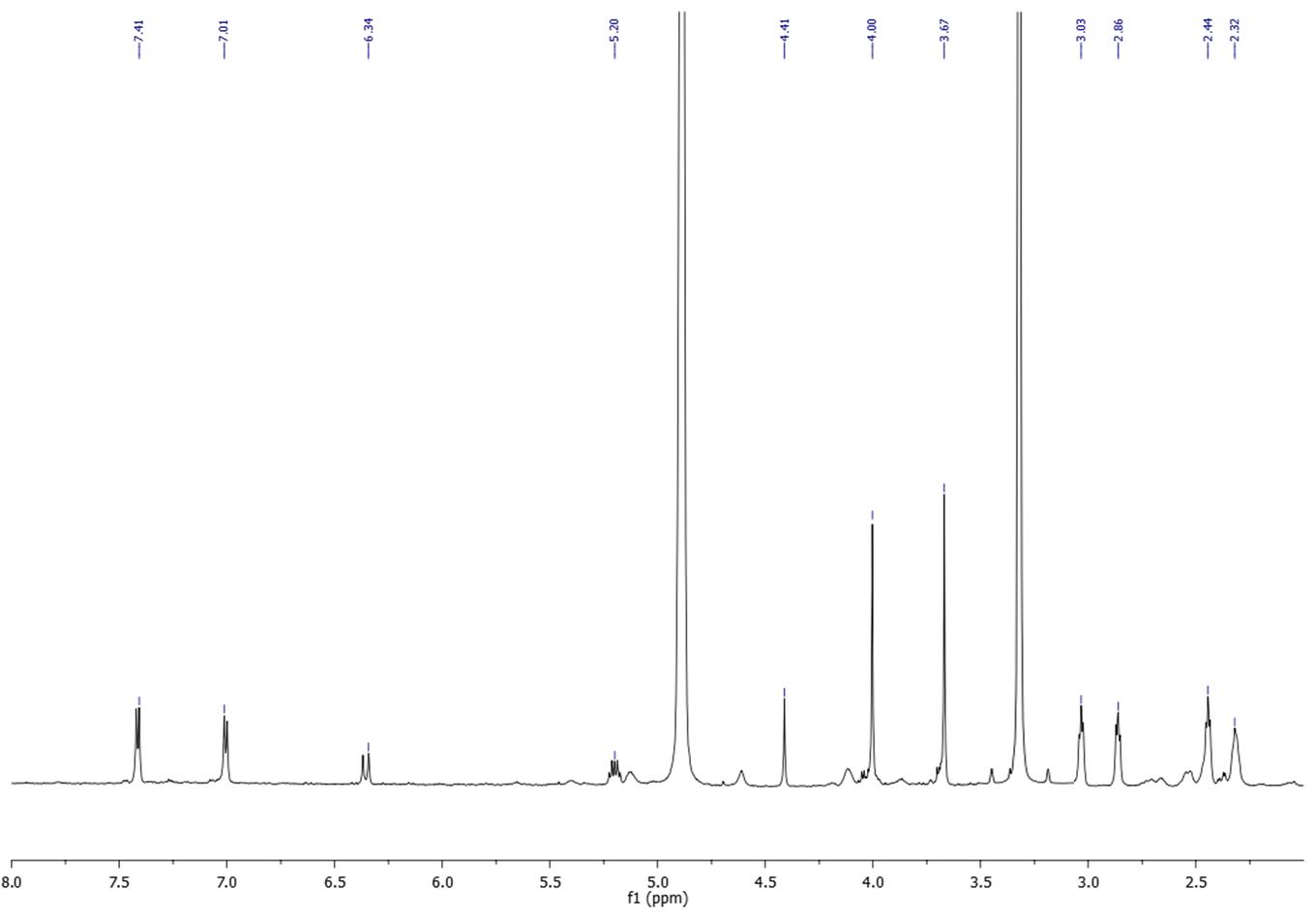


**Figure S1**. ^1^H NMR Spectrum (600 MHz, CD_3_OD) of giffonin A (**1**).


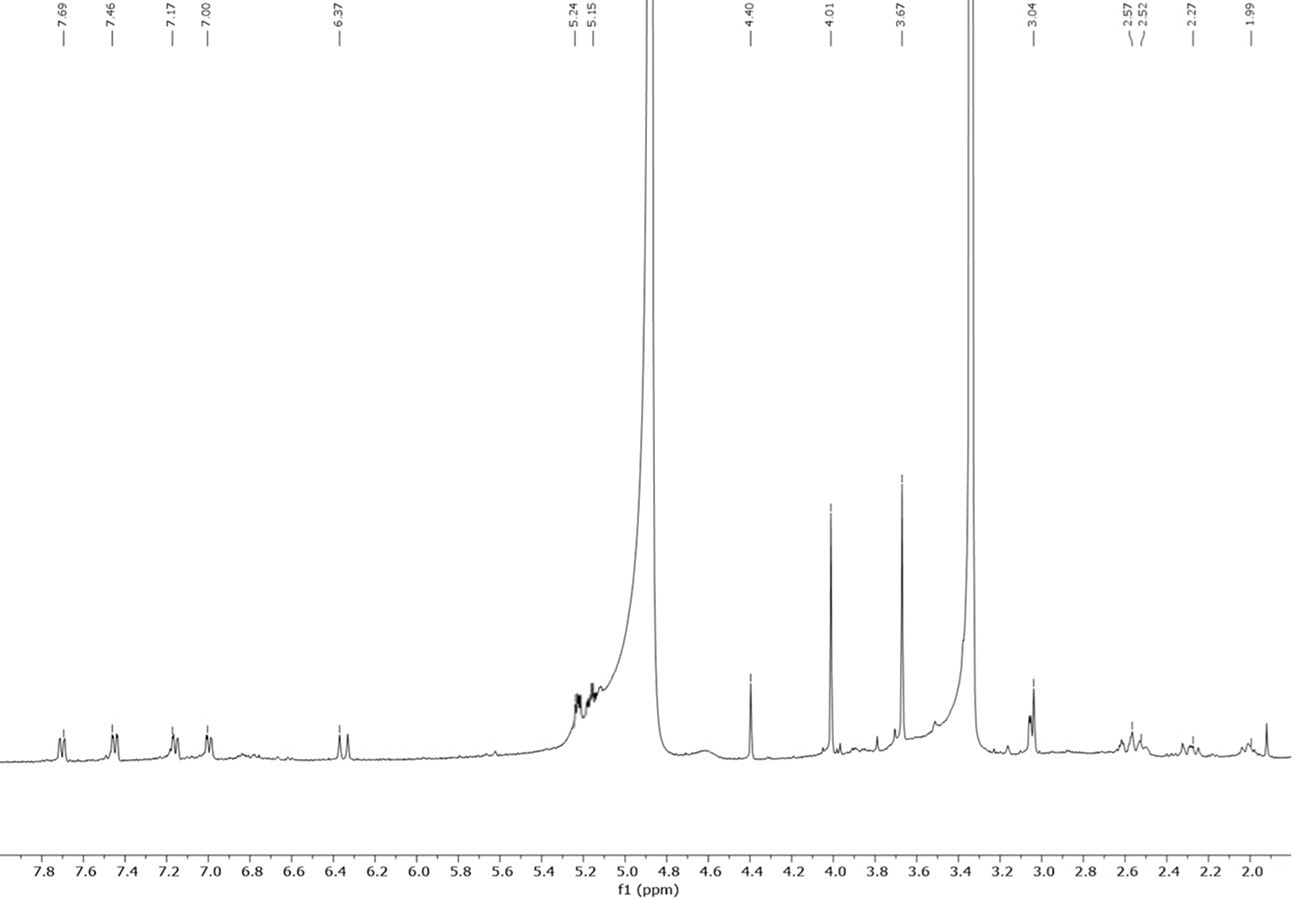


**Figure S2.** ^1^H NMR Spectrum (600 MHz, CD_3_OD) of giffonin B (**2**).


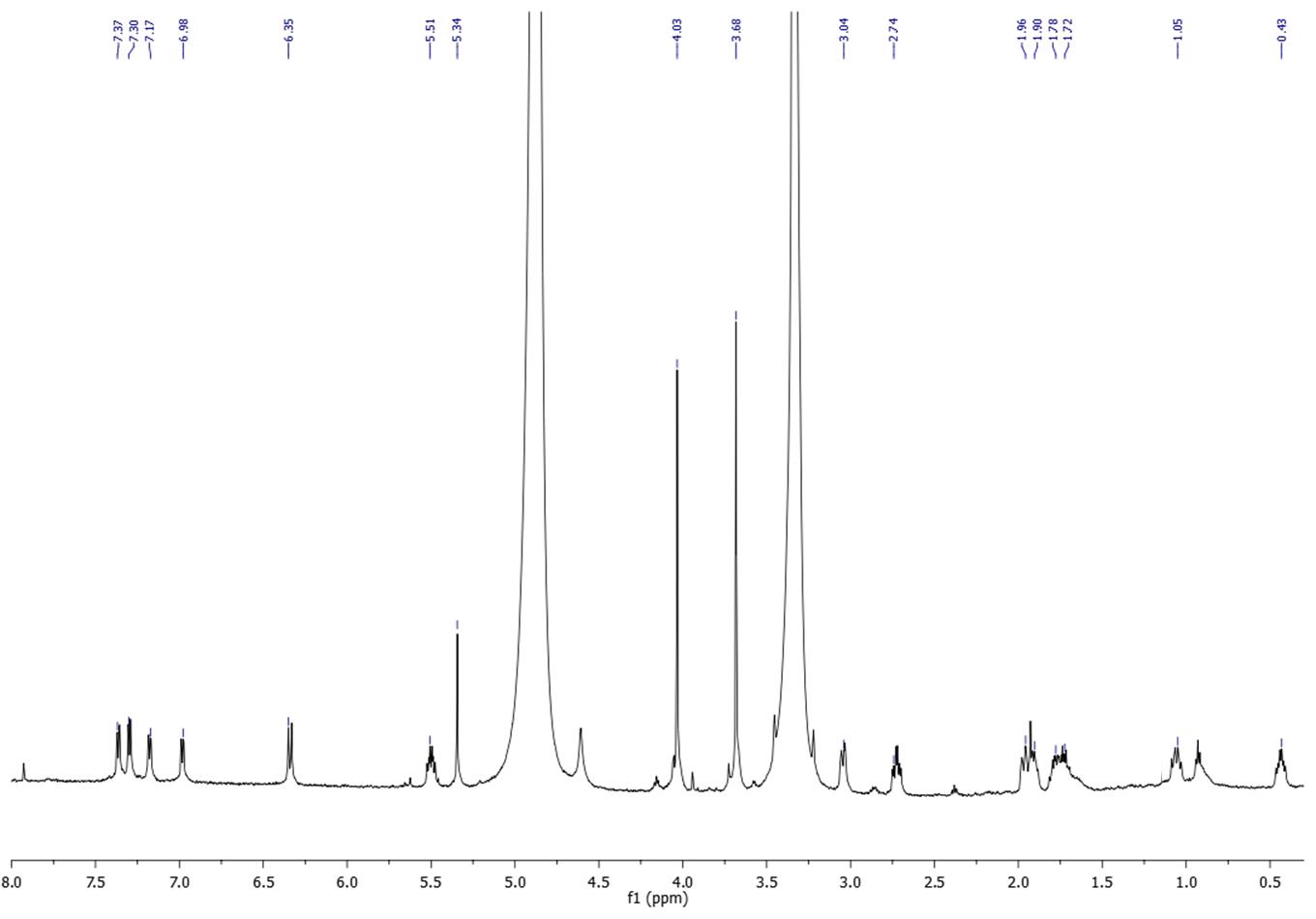


**Figure S3**. ^1^H NMR Spectrum (600 MHz, CD_3_OD) of giffonin C (**3)**.


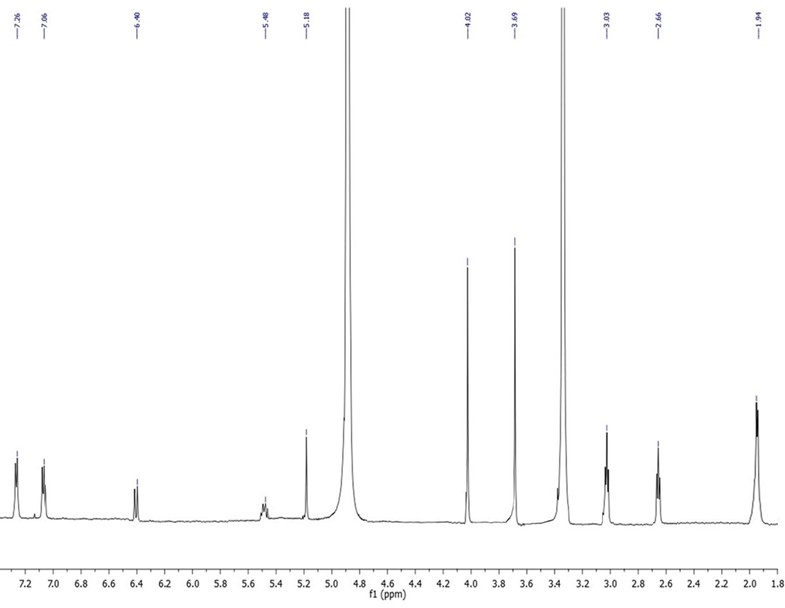


**Figure S4.** ^1^H NMR Spectrum (600 MHz, CD_3_OD) of giffonin D (**4**).


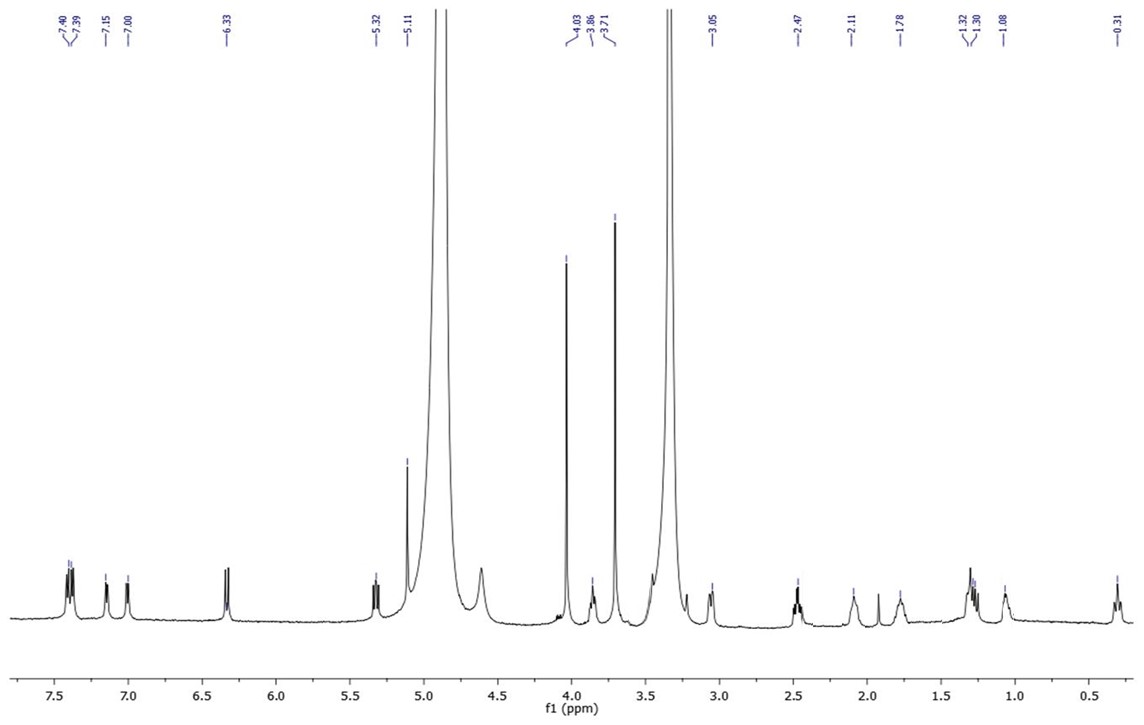


**Figure S5.** ^1^H NMR Spectrum (600 MHz, CD_3_OD) of giffonin E (**5**).


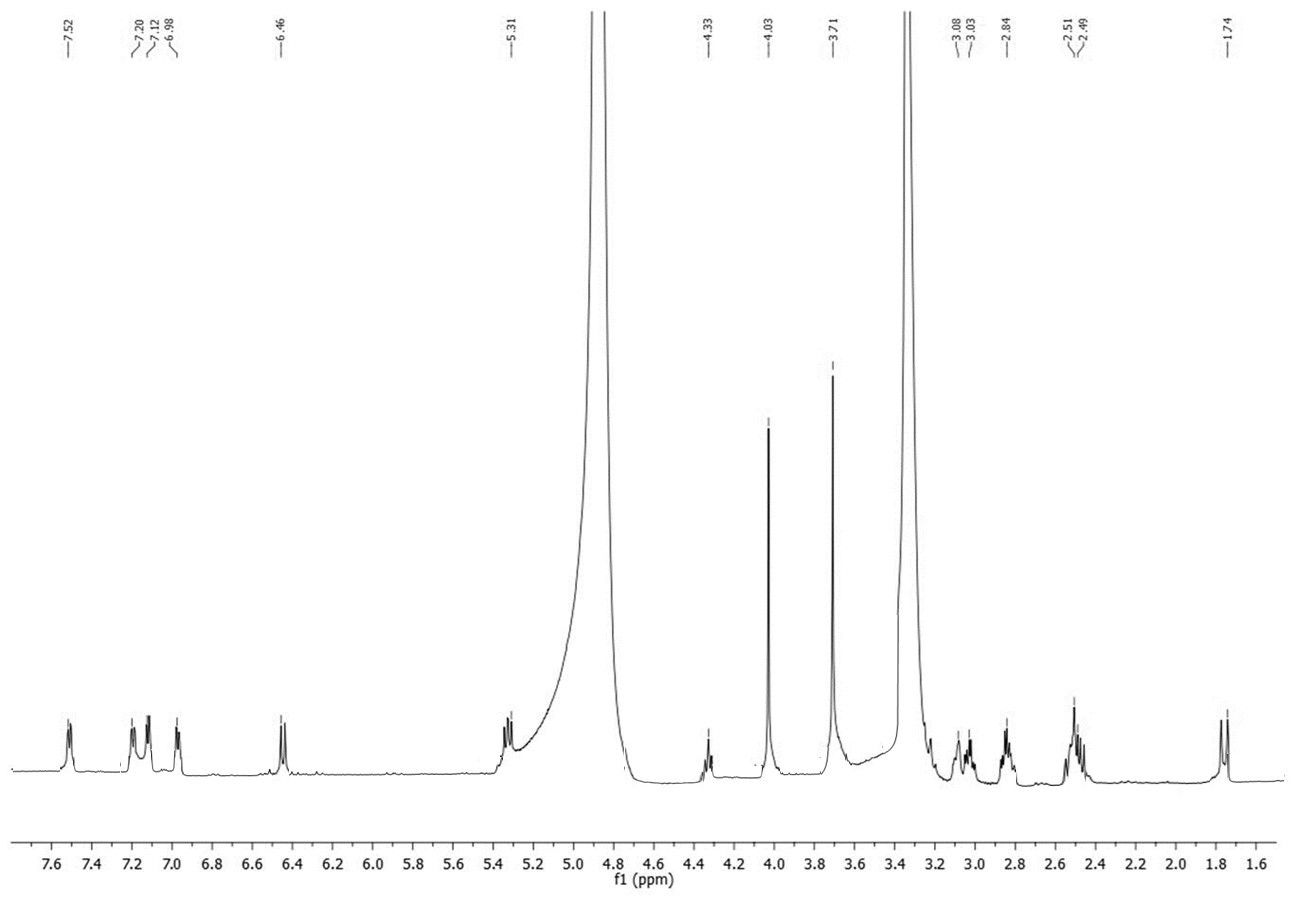


**Figure S6.** ^1^H NMR Spectrum (600 MHz, CD_3_OD) of giffonin F (**6**).


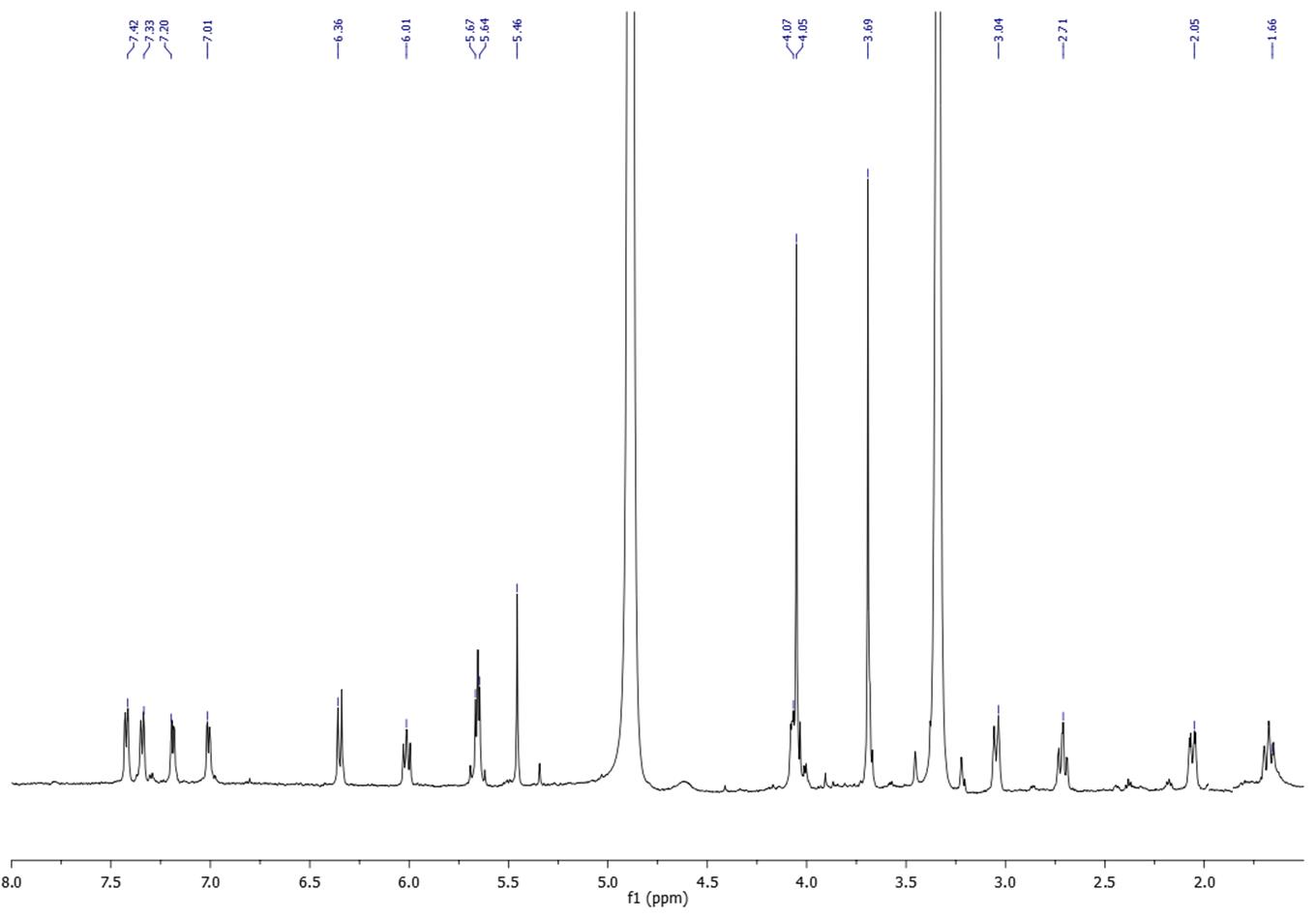


**Figure S7.** ^1^H NMR Spectrum (600 MHz, CD_3_OD) of giffonin G (**7**).


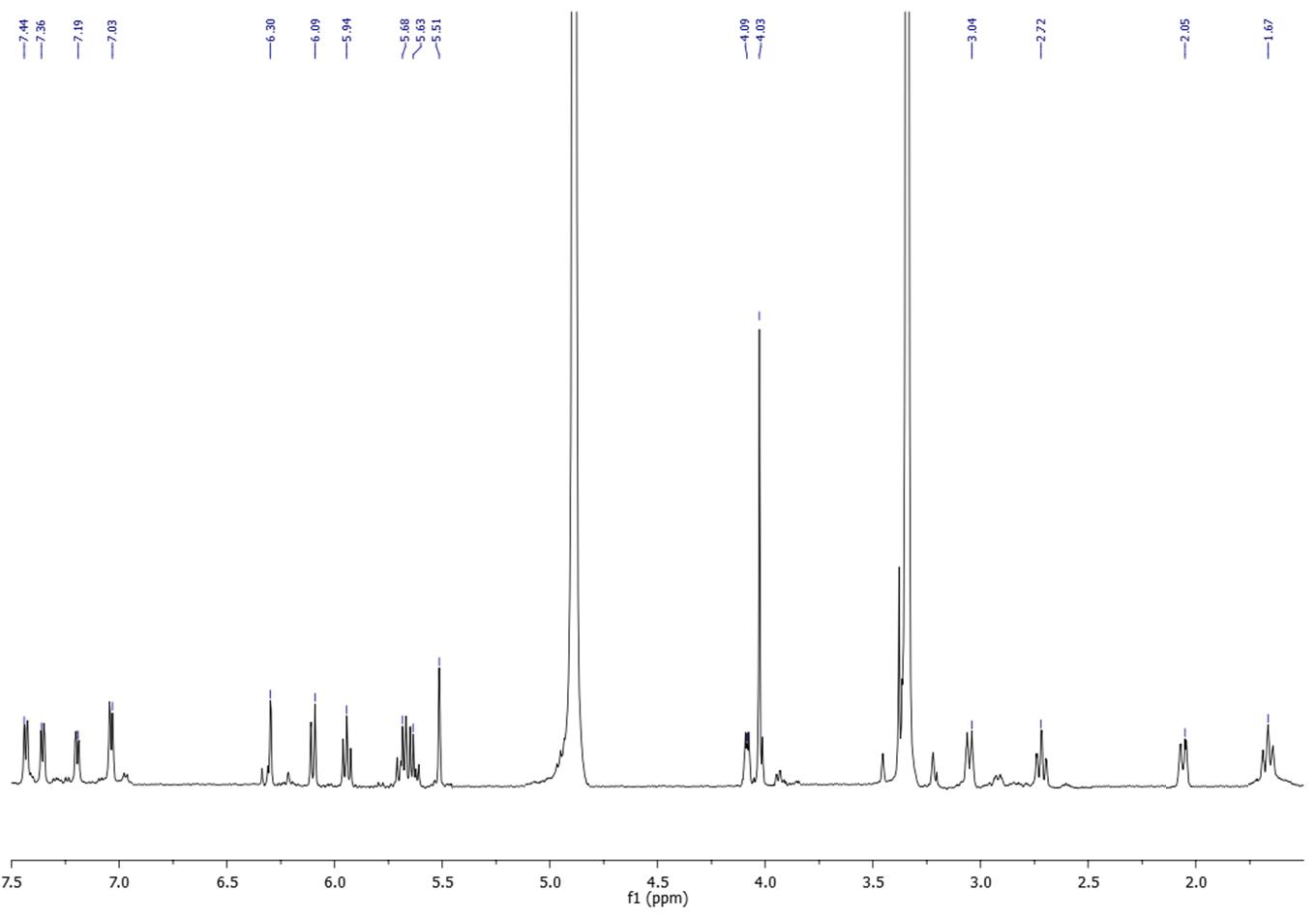


**Figure S8.** ^1^H NMR Spectrum (600 MHz, CD_3_OD) of giffonin H (**8**).


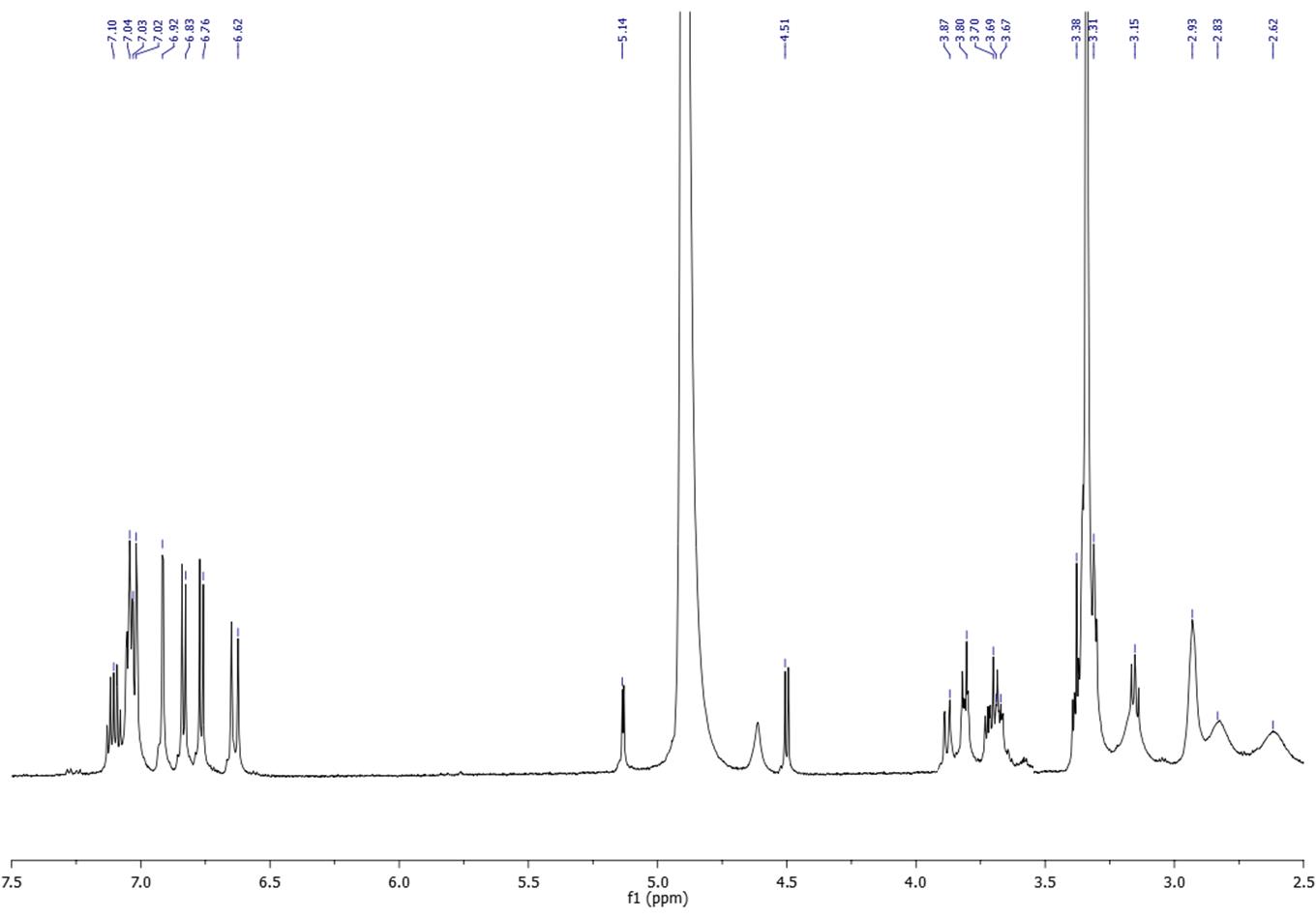


**Figure S9.** ^1^H NMR Spectrum (600 MHz, CD_3_OD) of giffonin I (**9**).


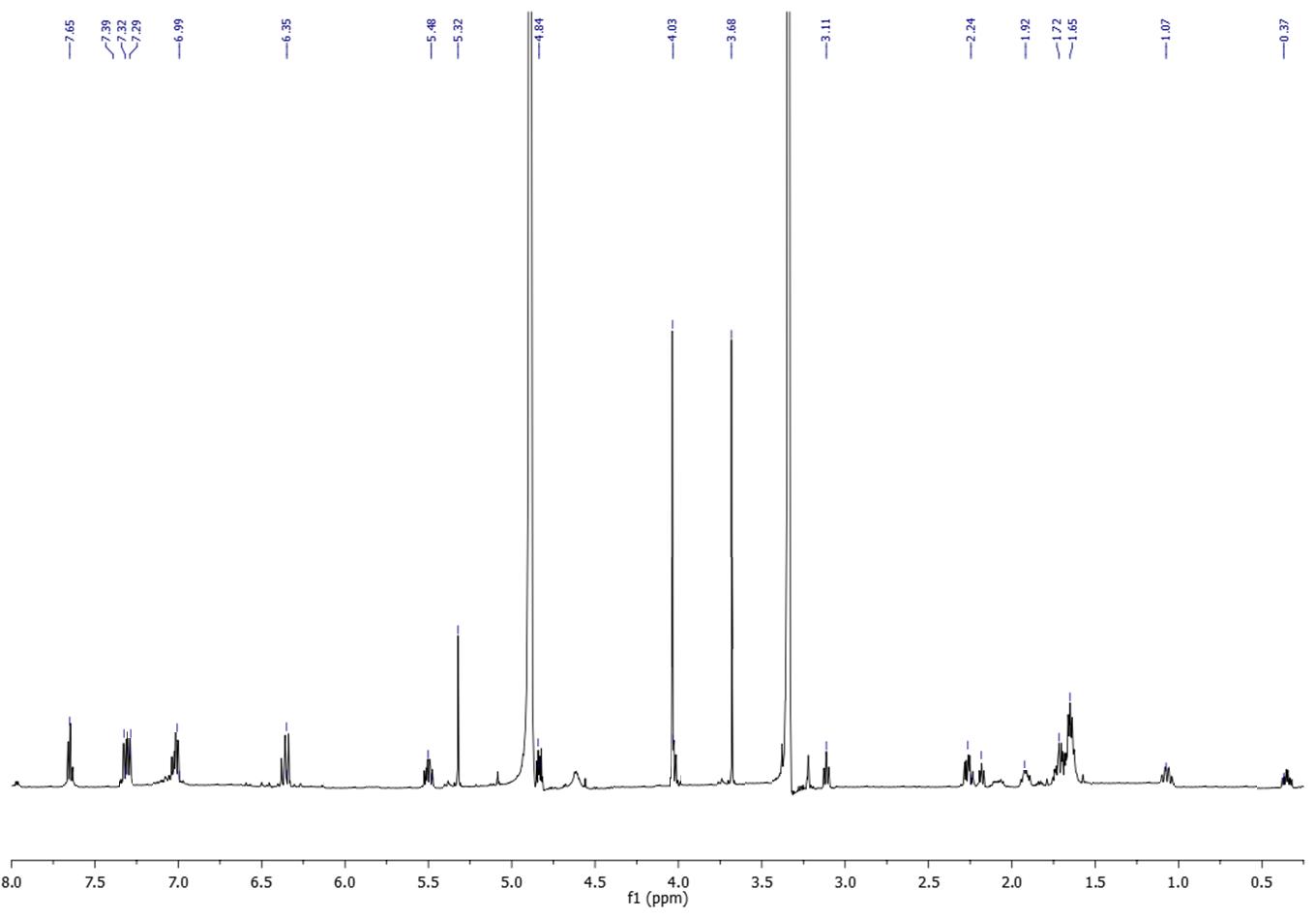


**Figure S10.** ^1^H NMR Spectrum (600 MHz, CD_3_OD) of giffonin J (**10**).


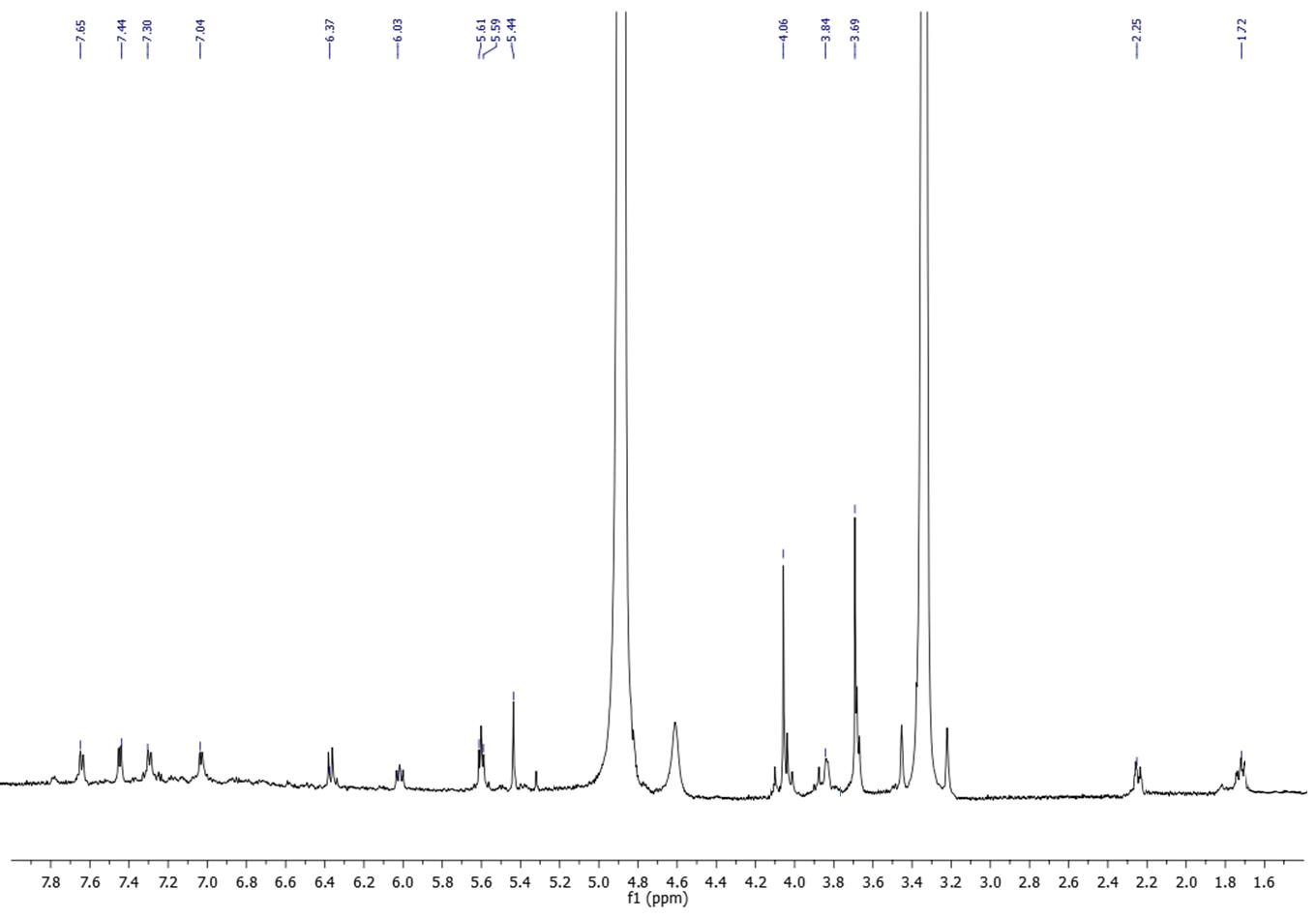


**Figure S11.** ^1^H NMR Spectrum (600 MHz, CD_3_OD) of giffonin K (**11**).


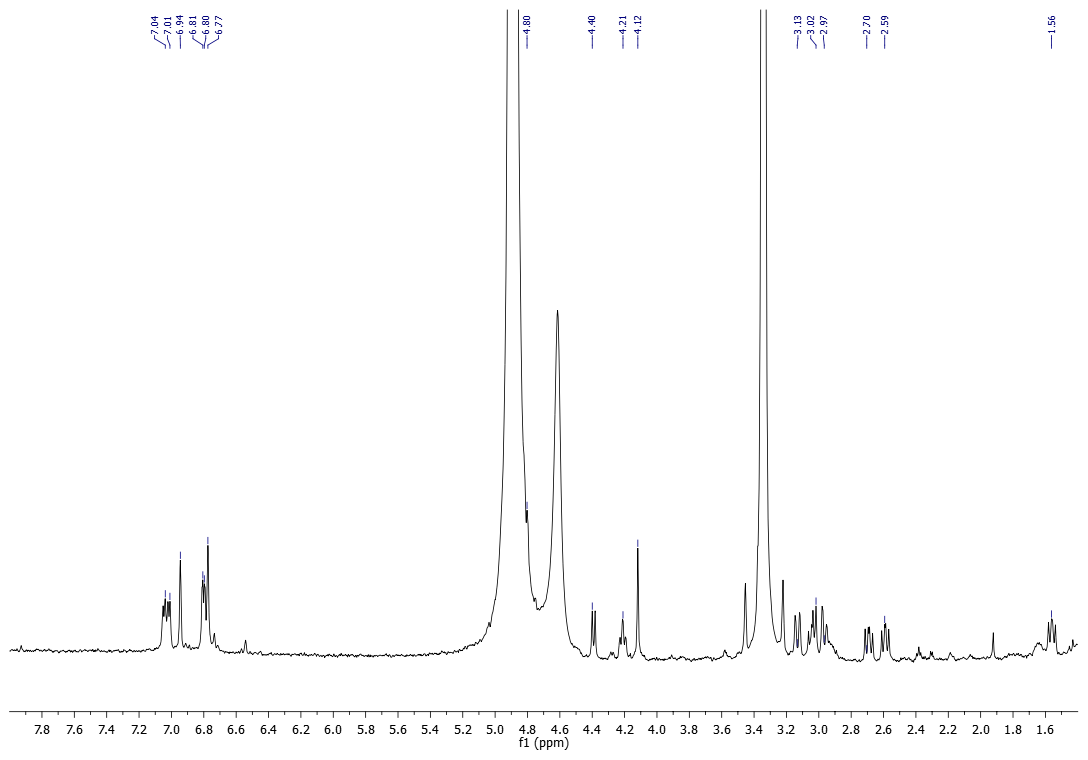


**Figure S12.** ^1^H NMR Spectrum (600 MHz, CD_3_OD) of giffonin L (**12**).


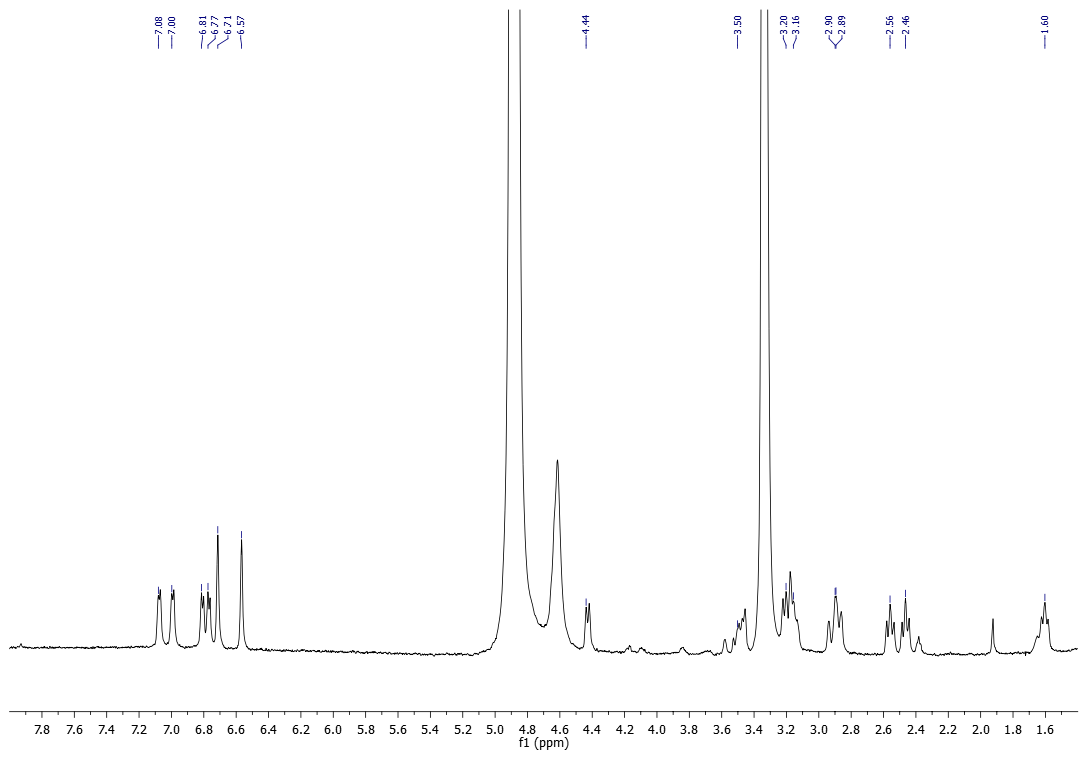


**Figure S13.** ^1^H NMR Spectrum (600 MHz, CD_3_OD) of giffonin M (**13**).


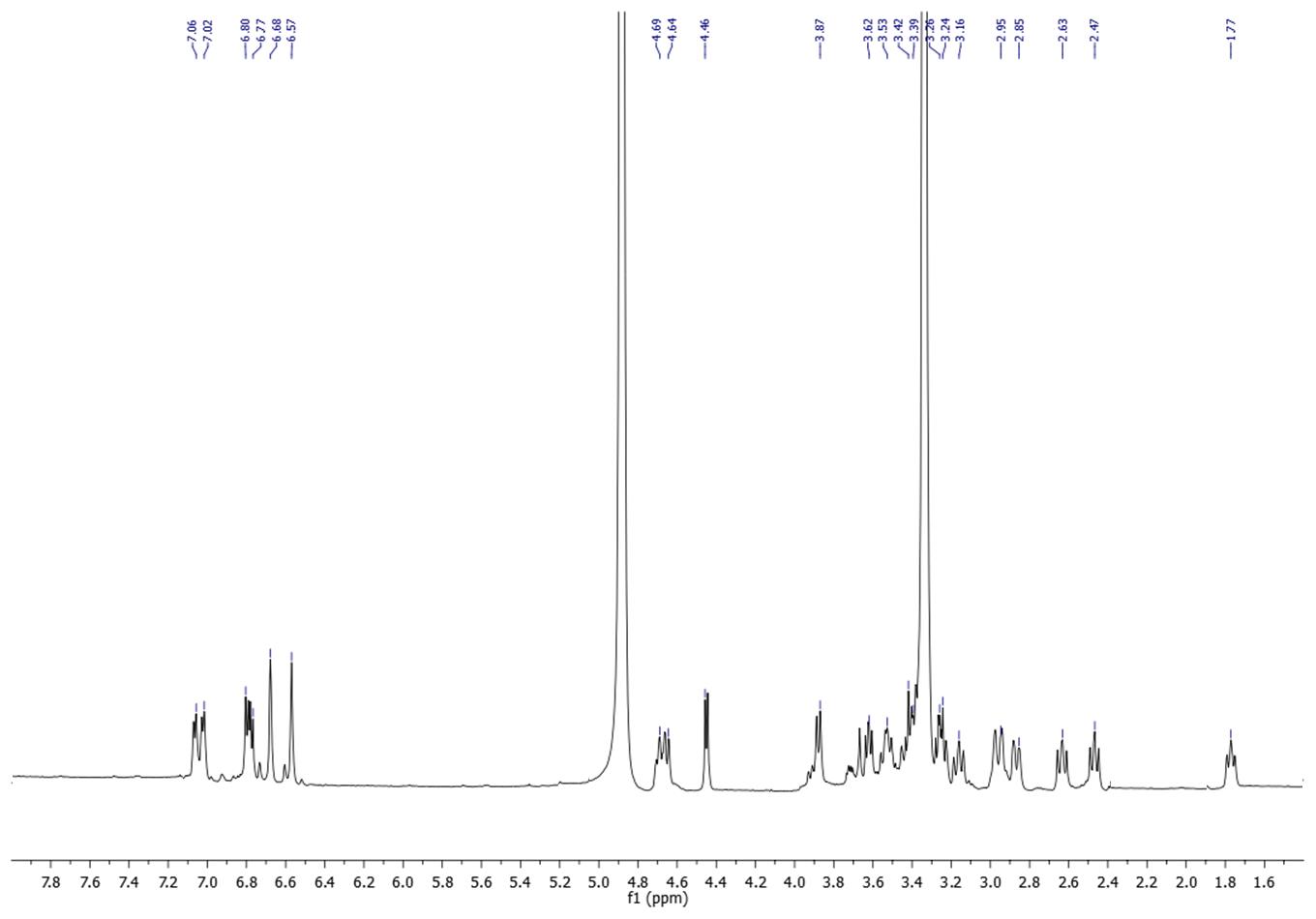


**Figure S14.** ^1^H NMR Spectrum (600 MHz, CD_3_OD) of giffonin N (**14**).


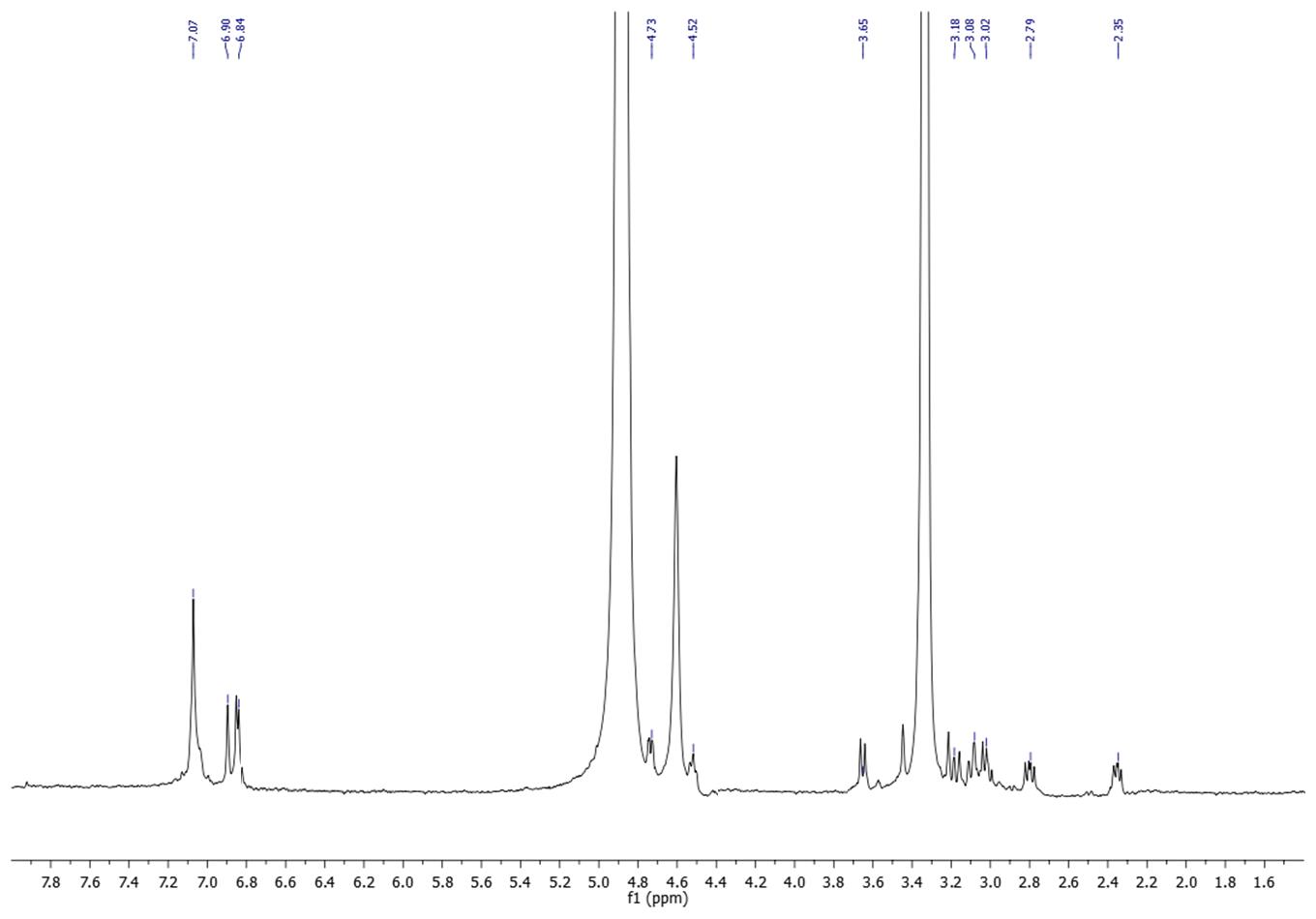


**Figure S15.** ^1^H NMR Spectrum (600 MHz, CD_3_OD) of giffonin O (**15**).


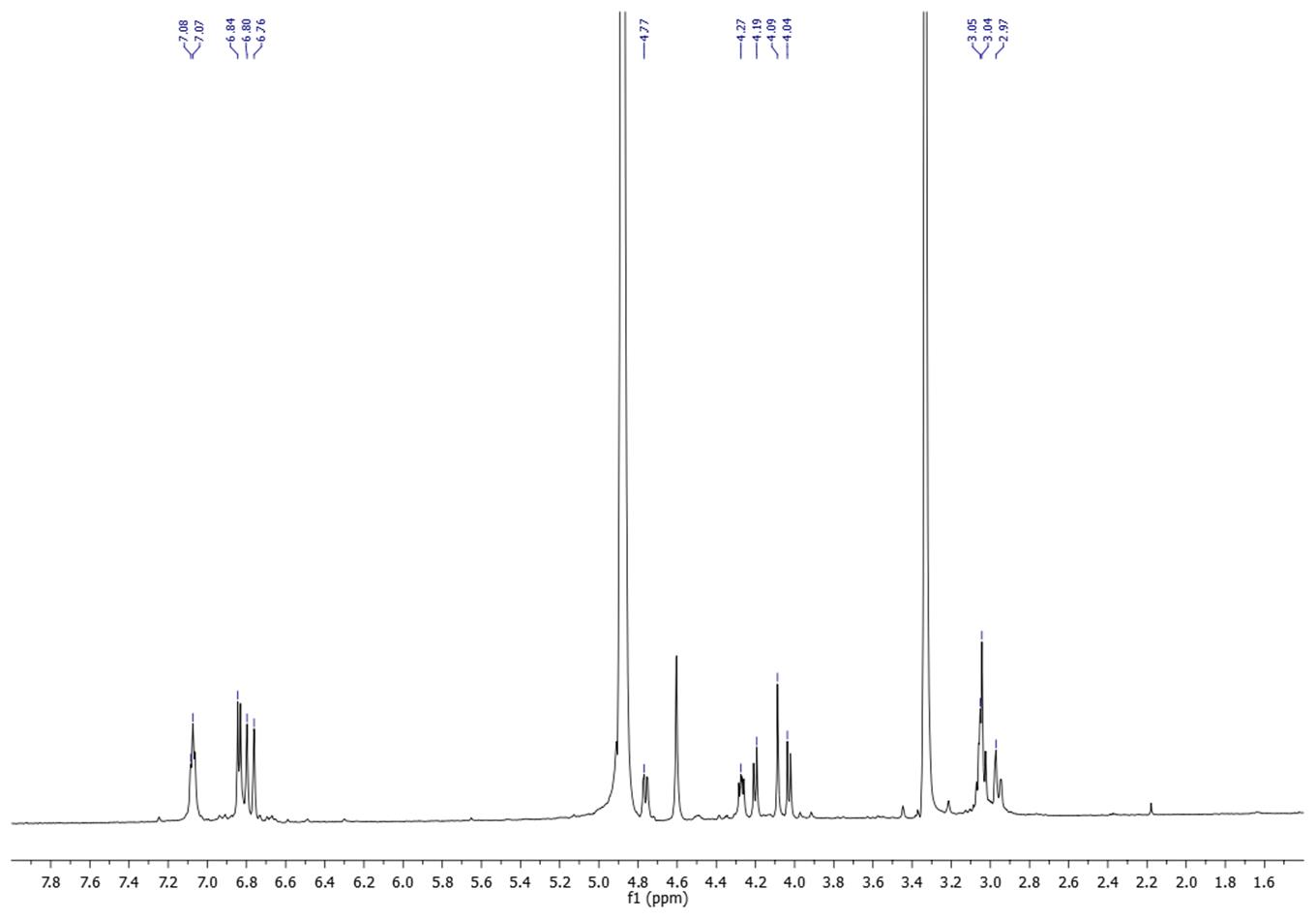


**Figure S16.** ^1^H NMR Spectrum (600 MHz, CD_3_OD) of giffonin P (**16**).


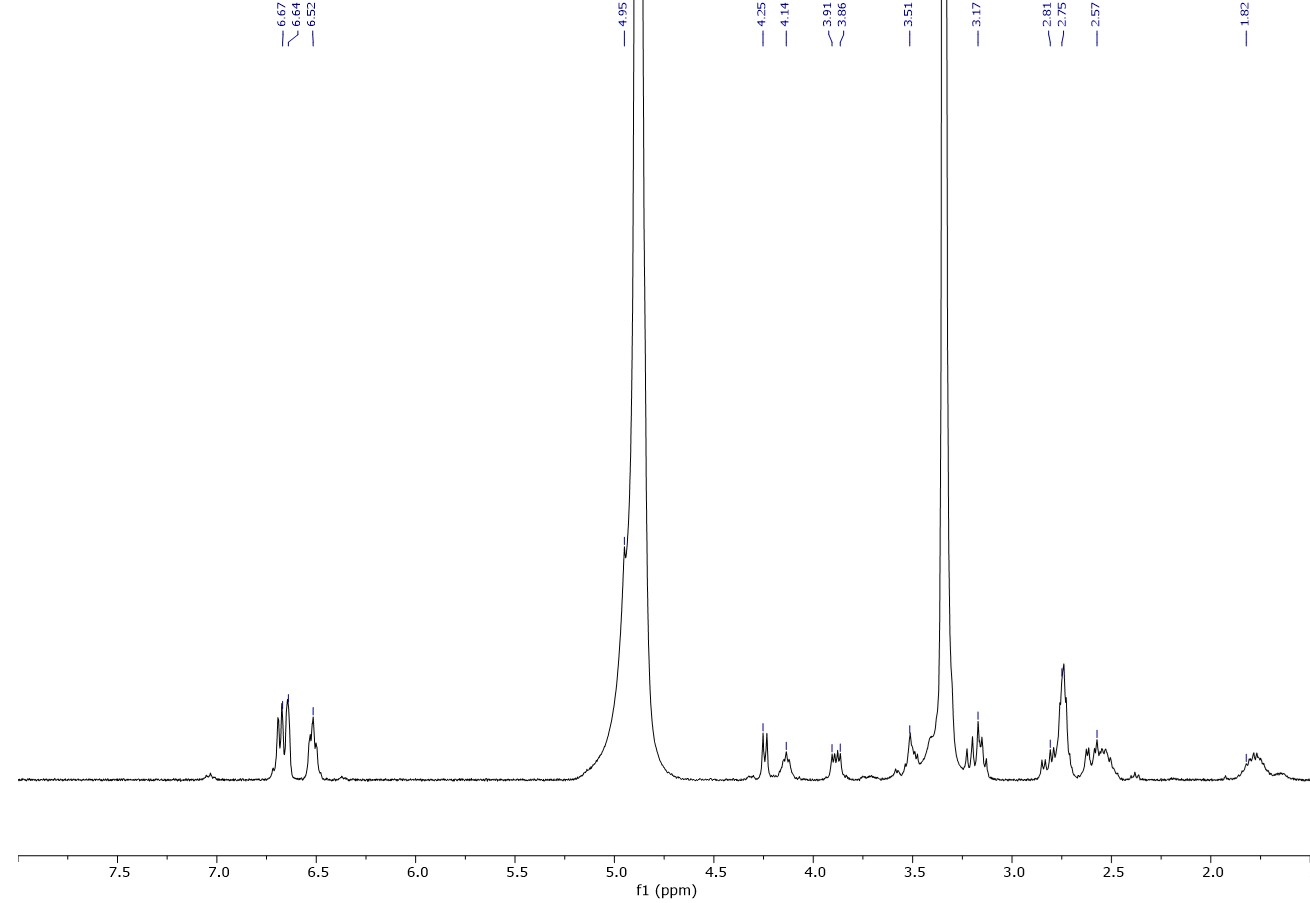


**Figure S17.** ^1^H NMR Spectrum (600 MHz, CD_3_OD) of oregonin (**17**).


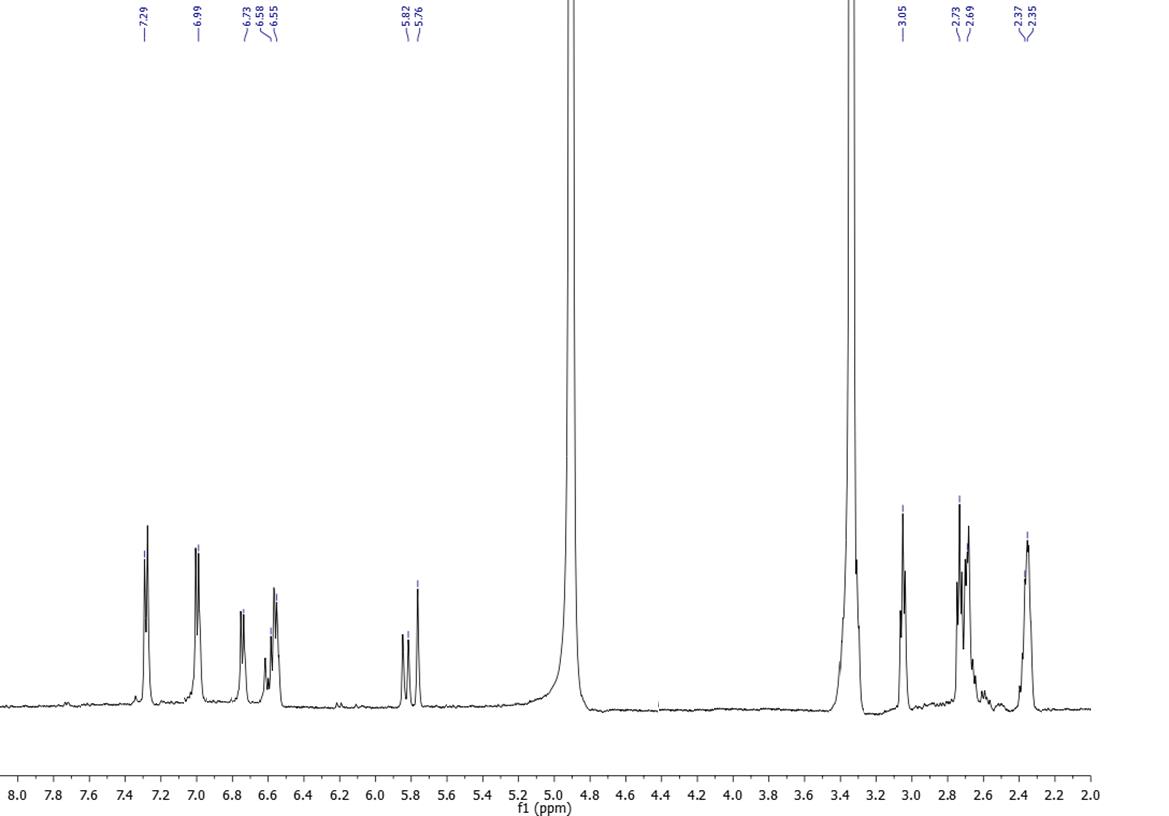


**Figure S18.** ^1^H NMR Spectrum (600 MHz, CD_3_OD) of giffonin Q (**18**).


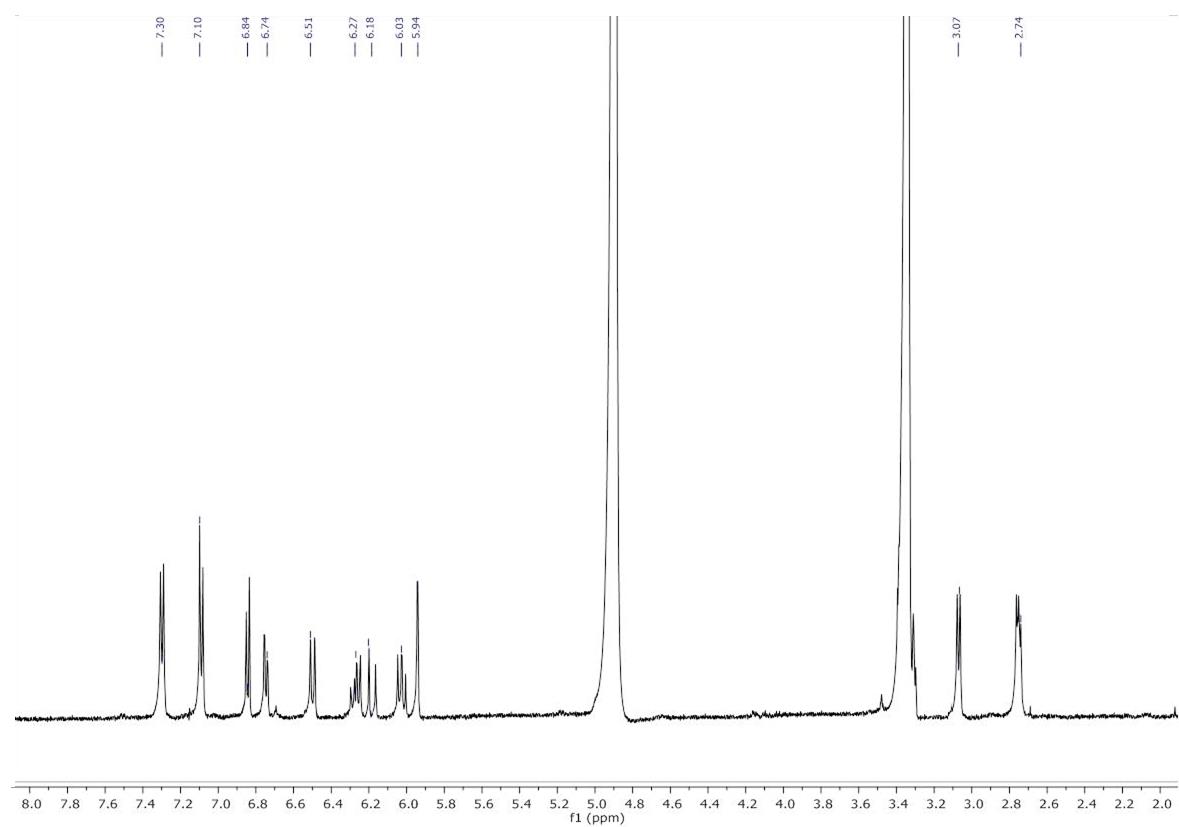


**Figure S19.** ^1^H NMR Spectrum (600 MHz, CD_3_OD) of giffonin R (**19**).


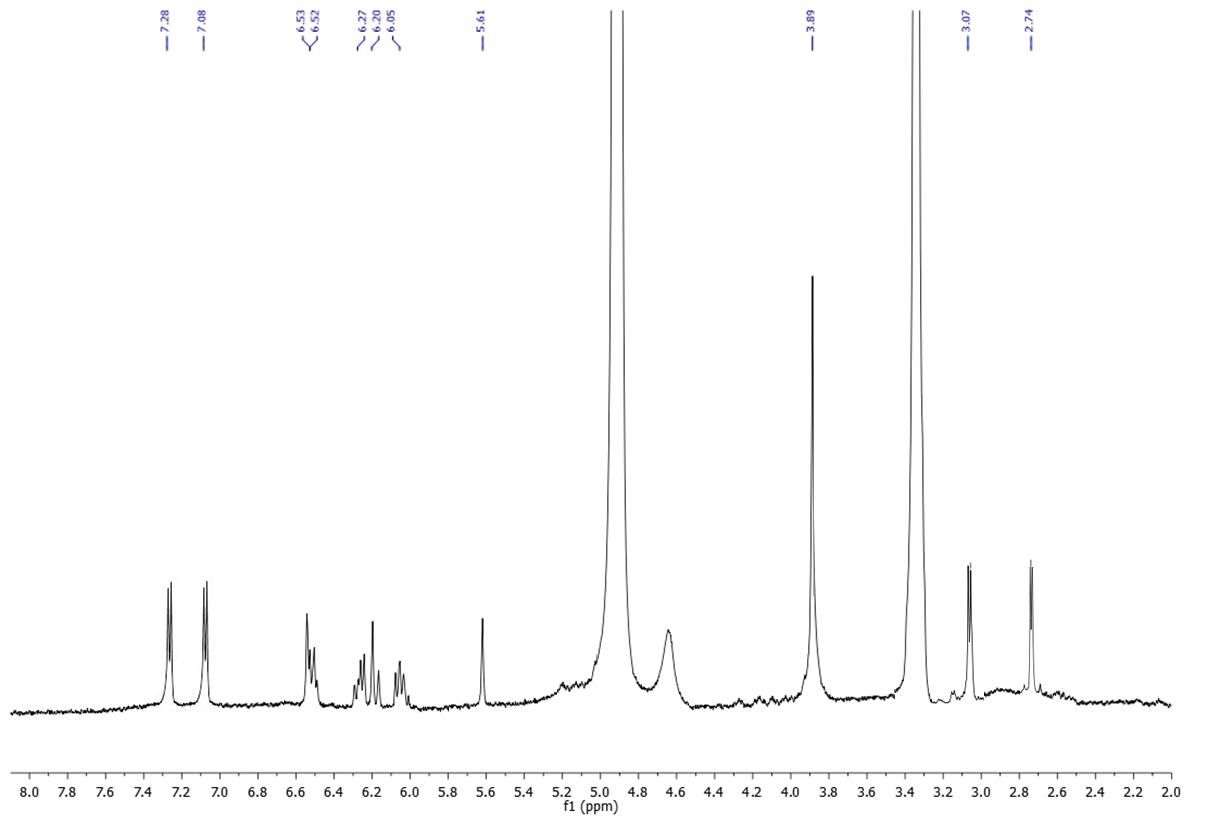


**Figure S20.** ^1^H NMR Spectrum (600 MHz, CD_3_OD) of giffonin S (**20**).

**
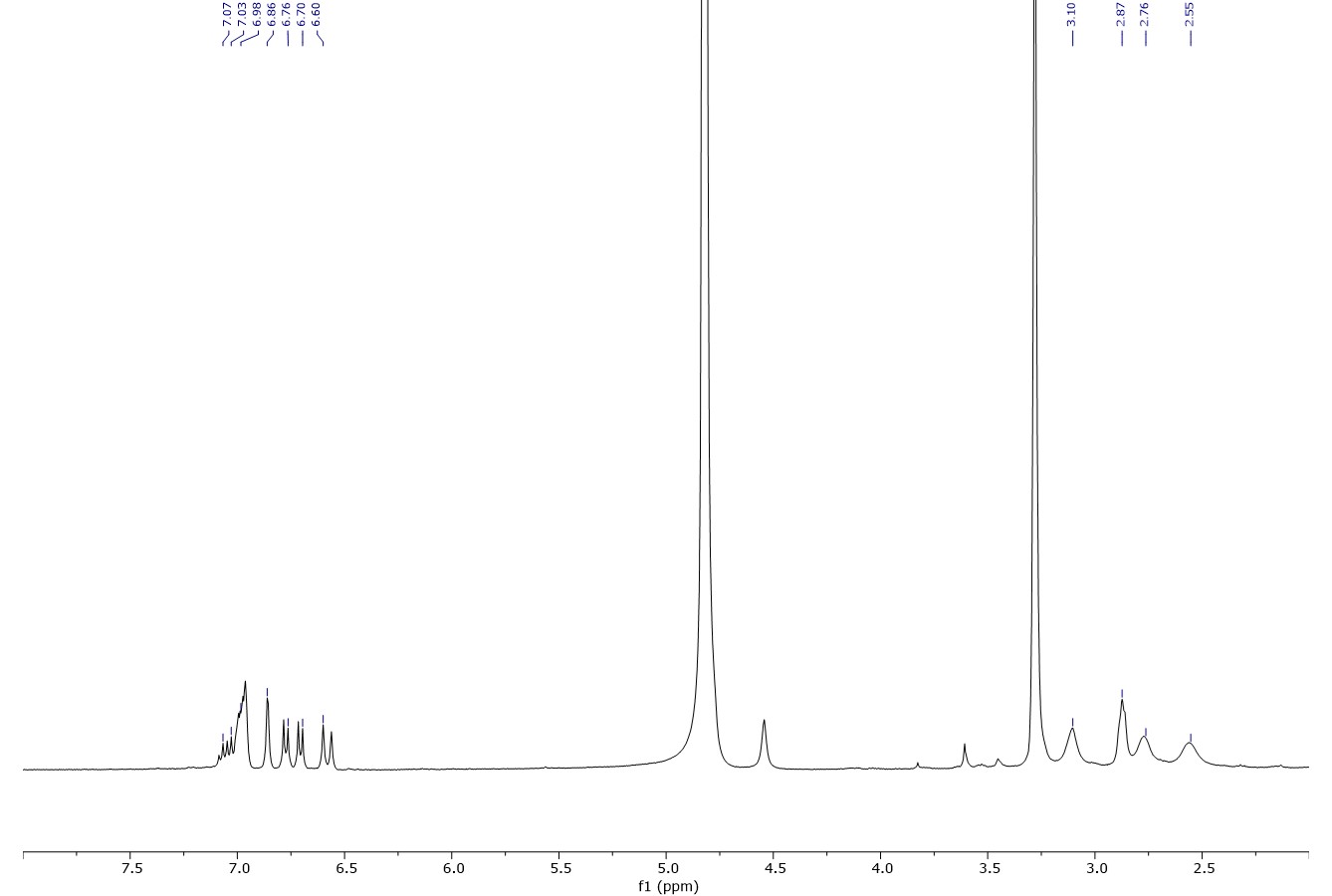
**

**Figure S21.** ^1^H NMR Spectrum (600 MHz, CD_3_OD) of alnusone (**21**).


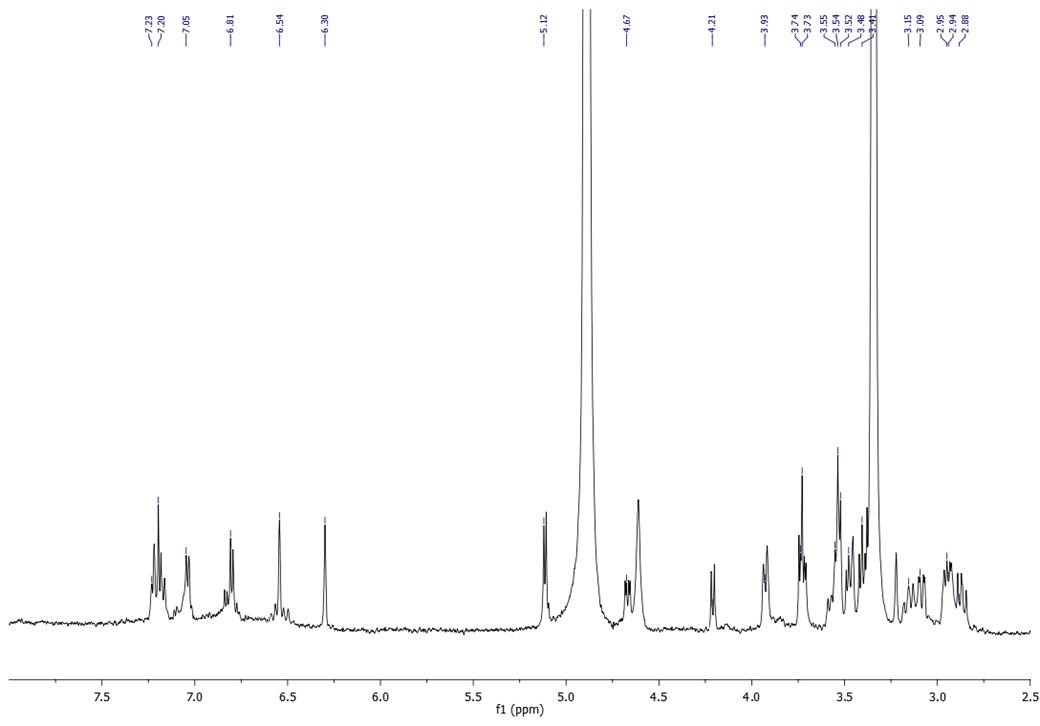


**Figure S22.** ^1^H NMR Spectrum (600 MHz, CD_3_OD) of giffonin T (**22**).


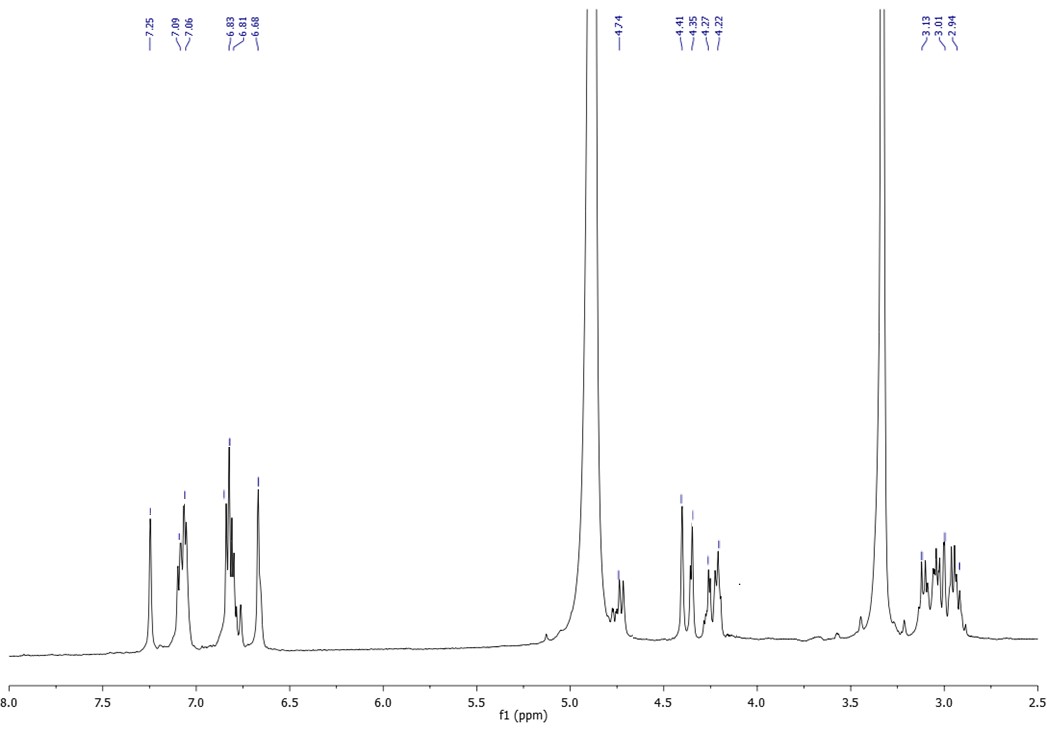


**Figure S23.** ^1^H NMR Spectrum (600 MHz, CD_3_OD) of giffonin U (**23**).

**
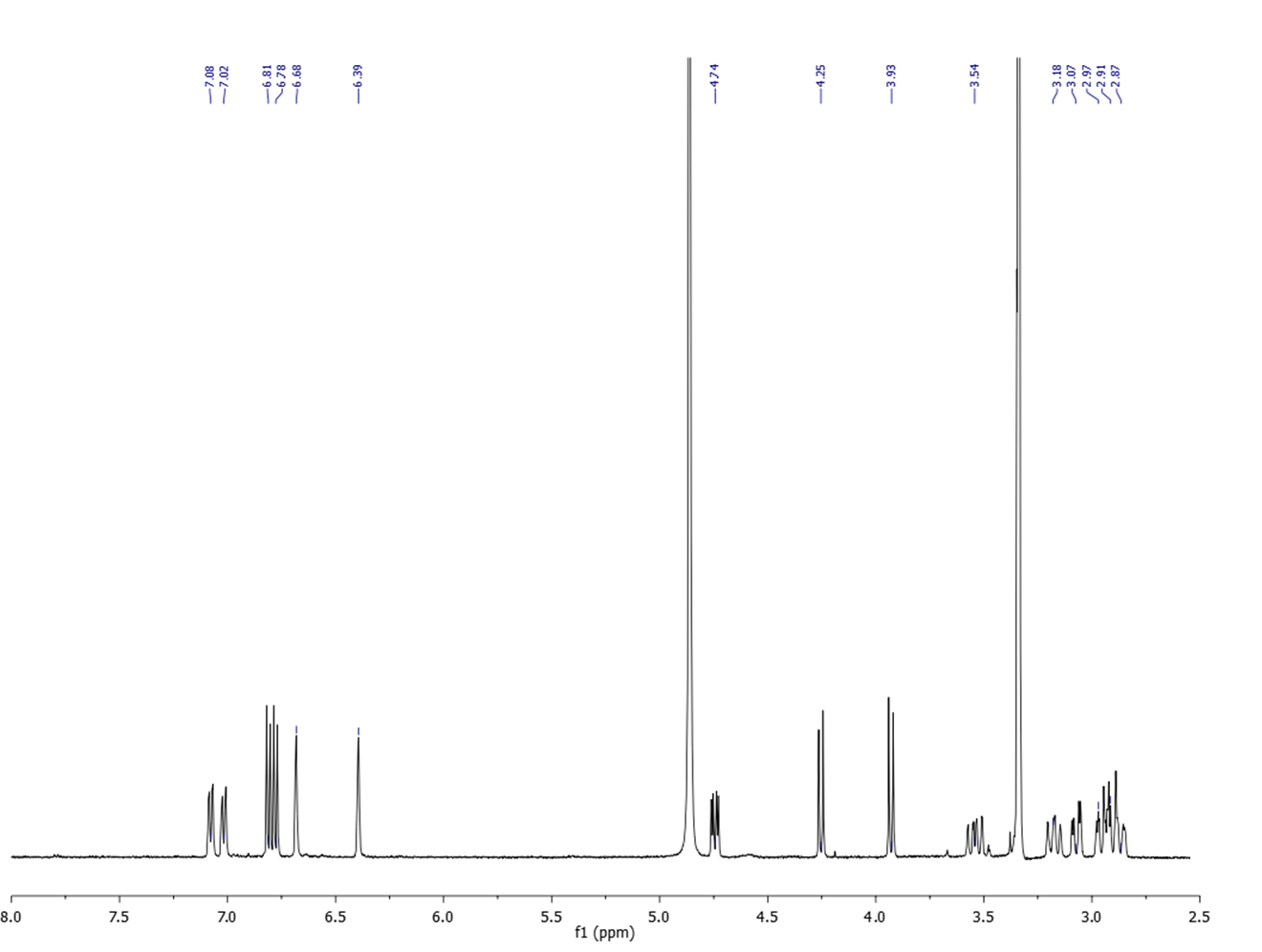
**

**Figure S24.** ^1^H NMR Spectrum (600 MHz, CD_3_OD) of carpinontriol B (**24**).

**
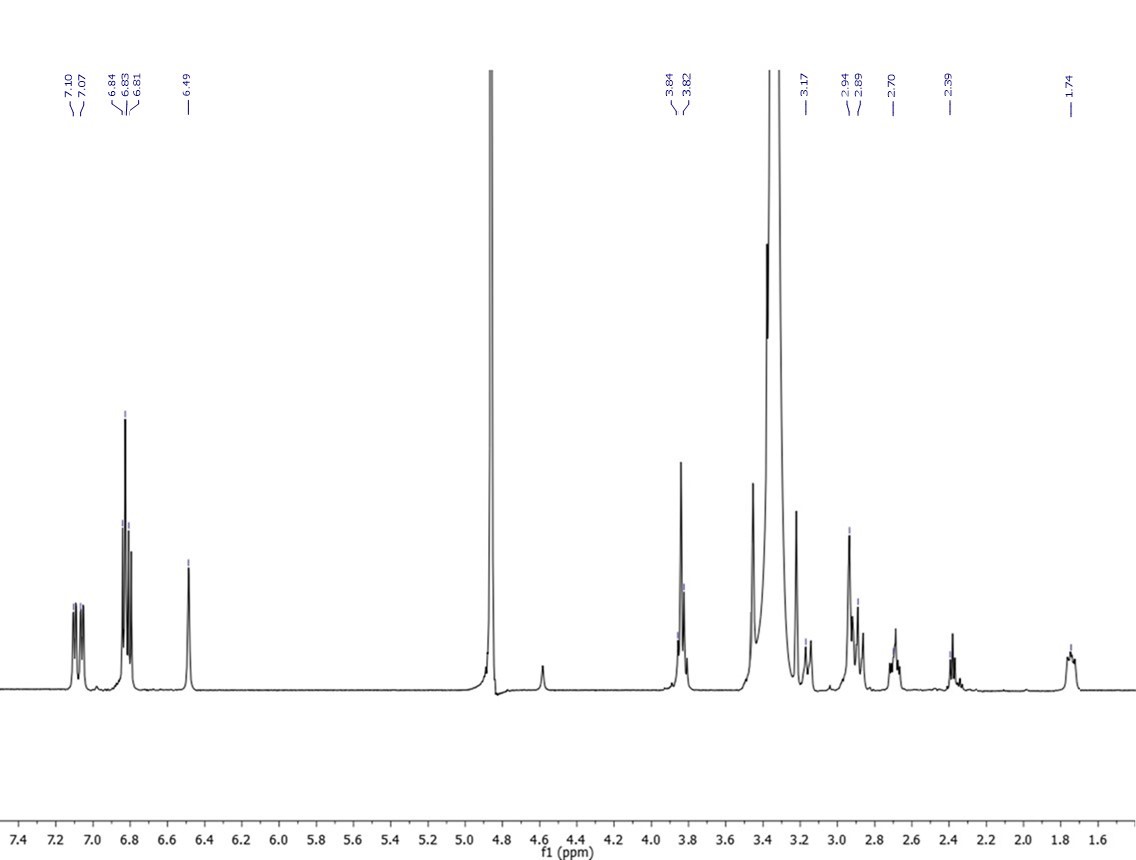
**

**Figure S25.** ^1^H NMR Spectrum (600 MHz, CD_3_OD) of giffonin V (**25**).


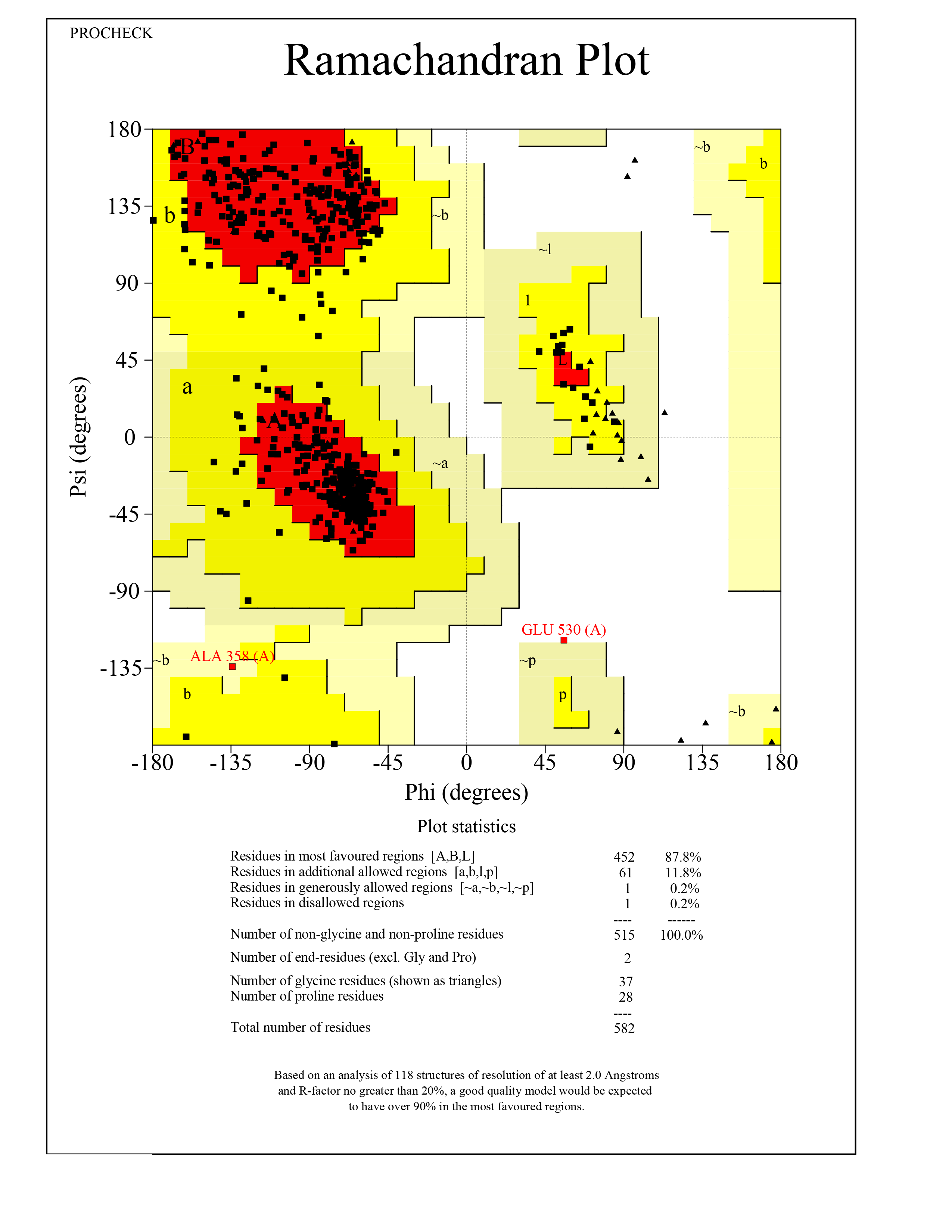


**Figure S26.** The Ramachandran plot showing the phi-psi torsion angles for all residues (except those at the chain termini) for the protein model of α-glucosidase from *Saccharomyces cerevisiae* built by homology modeling. Glycine residues are separately identified by triangles as these are not restricted to the regions of the plot appropriate to the other sidechain types. The colouring/shading on the plot represents the different regions described in Morris et al. (1992): the darkest areas (here shown in red) correspond to the "core" regions representing the most favourable combinations of phi-psi values. Ideally, one would hope to have over 90% of the residues in these "core" regions; in this protein model, this value was reached thus highlighting the reliability of the built structure.

**Table S1.** ^13^C NMR Data (150 MHz, CD_3_OD, δ ppm) of diaryletherheptanoids **1-8, 10, 11, 18-20**

| position | **giffonin A (1)** | **giffonin B (2)** | **giffonin C (3)** | **giffonin D (4)** | **giffonin E (5)** | **giffonin F (6)** | **giffonin G (7)** | **giffonin H (8)** | **giffonin J (10)** | **giffonin K (11)** | **giffonin Q (18)** | **giffonin R (19)** | **giffonin S (20)** |
| --- | --- | --- | --- | --- | --- | --- | --- | --- | --- | --- | --- | --- | --- |
| 1 | 152.7, C | 152.7, C | 151.8, C | 151.8, C | 151.9, C | 152.1, C | 150.4, C | 155.6, C | 150.1, C | 151.8, C | 150.7, C | 151.3, C | 151.2, C |
| 2 | 136.9, C | 137.0, C | 137.5, C | 137.6, C | 137.6, C | 138.3, C | 136.7, C | 136.9, C | 136.2, C | 138.0, C | 144.8, C | 147.9, C | 137.3, C |
| 3 | 144.3, C | 144.1, C | 144.7, C | 145.0, C | 144.5, C | 145.2, C | 144.2, C | 151.7, C | 143.1, C | 144.8, C | 116.2, CH | 117.4, CH | 149.2, C |
| 4 | 140.4, C | 140.6, C | 141.6, C | 141.9, C | 141.6, C | 142.4, C | 140.4, C | 110.8, CH | 141.1, C | 141.9, C | 122.5, CH | 125.0, CH | 108.0, CH |
| 5 | 127.8, C | 128.0, C | 124.7, C | 125.7, C | 125.7, C | 124.7, C | 125.1, C | 133.5, C | 123.6, C | 125.3, C | 134.4, C | 129.8, C | 136.8, C |
| 6 | 109.0, CH | 109.2, CH | 108.3, CH | 108.0, CH | 108.5, CH | 108.3, CH | 110.0, CH | 109.6, CH | 108.0, CH | 110.3, CH | 115.8, CH | 119.6, CH | 113.7, CH |
| 7 | 125.5, CH | 125.6, CH | 124.6, CH | 125.9, CH | 123.0, CH | 124.9, CH | 124.5, CH | 129.9, CH | 124.9, CH | 125.2, CH | 32.0, CH_2_ | 136.2, CH | 136.6, CH |
| 8 | 135.5, CH | 135.5, CH | 132.5, CH | 131.0, CH | 136.8, CH | 132.2, CH | 129.0, CH | 128.8, CH | 132.2, CH | 129.1, CH | 33.7, CH_2_ | 124.3, CH | 125.6, CH |
| 9 | 26.7, CH_2_ | 26.1, CH_2_ | 27.0, CH_2_ | 23.0, CH_2_ | 69.5, CH | 65.3, CH | 126.6, CH | 126.8, CH | 27.3, CH_2_ | 127.0, CH | 149.7, CH | 138.5, CH | 138.9, CH |
| 10 | 44.3, CH_2_ | 45.2, CH_2_ | 40.1, CH_2_ | 46.5, CH_2_ | 39.0, CH_2_ | 51.3, CH_2_ | 139.9, CH | 140.1, CH | 39.5, CH_2_ | 139.2, CH | 131.8, CH | 135.5, CH | 135.8, CH |
| 11 | 211.1, C | 209.5, C | 72.6, CH | 213.9, C | 22.9, CH_2_ | 215.4, C | 73.2, CH | 73.2, CH | 69.4, CH | 71.3, CH | 203.9, C | 206.0, C | 206.1, C |
| 12 | 46.2, , CH_2_ | 54.2, CH_2_ | 40.9, CH_2_ | 45.0, CH_2_ | 30.5, CH_2_ | 44.7, CH_2_ | 42.3, CH_2_ | 42.5, CH_2_ | 50.1, CH_2_ | 50.6, CH_2_ | 43.9, CH_2_ | 43.2, CH_2_ | 43.5, CH_2_ |
| 13 | 29.8, CH_2_ | 71.2, CH | 34.2, CH_2_ | 33.6, CH_2_ | 36.3, CH_2_ | 32.9, CH_2_ | 34.3, CH_2_ | 34.6, CH_2_ | 73.5, CH | 73.7, CH | 33.6, CH_2_ | 32.8, CH_2_ | 32.8, CH_2_ |
| 14 | 141.0, C | 143.4, C | 140.0, C | 138.6, C | 140.1, C | 138.6, C | 139.3, C | 140.9, C | 141.8, C | 143.0, C | 138.0, C | 140.1, C | 140.1, C |
| 15 | 132.2, CH | 131.4, CH | 130.8, CH | 131.8, CH | 130.9, CH | 130.5, CH | 132.8, CH | 133.2, CH | 130.2, CH | 127.9, CH | 131.5, CH | 131.7, CH | 131.7, CH |
| 16 | 125.2, CH | 124.4, CH | 124.9, CH | 124.5, CH | 124.9, CH | 125.0, CH | 122.8, CH | 123.0, CH | 123.1, CH | 126.5, CH | 124.0, CH | 124.6, CH | 125.0, CH |
| 17 | 155.8, C | 158.5, C | 156.3, C | 157.0, C | 155.8, CH | 156.9, C | 155.2, C | 156.7, C | 156.4, C | 157.6, C | 156.8, C | 157.8, C | 158.3, C |
| 18 | 125.2, CH | 125.8, CH | 123.8, CH | 124.5, CH | 123.2, CH | 123.8, CH | 125.6, CH | 126.3, CH | 125.4, CH | 122.2, CH | 124.0, CH | 124.6, CH | 125.0, CH |
| 19 | 132.2, CH | 128.4, CH | 132.8, CH | 131.8, CH | 134.0, CH | 134.0, CH | 130.5, CH | 130.7, CH | 127.9, CH | 131.0, CH | 131.5, CH | 131.7, CH | 131.7CH |
| 2-OCH_3_ | 61.7, CH_3_ | 61.6, CH_3_ | 61.3, CH_3_ | 61.4, CH_3_ | 61.4, CH_3_ | 61.2, CH_3_ | 61.2, CH_3_ | 60.0, CH_3_ | 61.3, CH_3_ | 61.3, CH_3_ |  |  |  |
| 3-OCH_3_ |  |  |  |  |  |  |  |  |  |  |  |  | 56.5, CH_3_ |
| 4-OCH_3_ | 61.2, CH_3_ | 61.0, CH_3_ | 61.0, CH_3_ | 61.2, CH_3_ | 61.2, CH_3_ | 61.2, CH_3_ | 61.0, CH_3_ |  | 61.3, CH_3_ | 61.2, CH_3_ |  |  |  |

**Table S2.** ^13^C NMR Data (150 MHz, CD_3_OD, δ ppm) of diarylheptanoids **9, 12-16, 21-25**

| position | **giffonin I (9)** | **giffonin L (12)** | **giffonin M (13)** | **giffonin N (14)** | **giffonin O (15)** | **giffonin P (16)** | **alnusone (21)** | **giffonin T (22)** | **giffonin U (23)** | **Carpinontriol B (24)** | **giffonin V (25)** |
| --- | --- | --- | --- | --- | --- | --- | --- | --- | --- | --- | --- |
| 1 | 127.4, C | 127.6, C | 127.0, C | 128.0, C | 128.9, C | 128.7, C | 125.1, C | 128.4, C | 126.8, C | 127.1, C | 127.2, C |
| 2 | 127.5, C | 128.0, C | 127.7, C | 127.0, C | 128.7, C | 128.7, C | 128.8, C | 127.7, C | 128.4, C | 127.0, C | 27.1, C |
| 3 | 152.4, C | 153.8, C | 153.8, C | 154.0, C | 153.9, C | 152.8, C | 149.0, C | 151.9, C | 153.1, C | 153.3, C | 151.8, C |
| 4 | 117.6, CH | 117.3, CH | 117.0, CH | 117.2, CH | 117.2, CH | 116.8, CH | 115.7, CH | 117.3, CH | 116.8, CH | 117.1, CH | 116.2, CH |
| 5 | 128.9, CH | 129.8, CH | 130.0, CH | 130.2, CH | 130.3, CH | 130.3, CH | 128.1, CH | 130.6, CH | 130.6, CH | 129.2, CH | 130.6, CH |
| 6 | 137.6, C | 131.4, C | 130.1, C | 134.0, C | 130.1, C | 130.3, C | 131.2, C | 130.2, C | 130.6, C | 130.7, C | 130.8, C |
| 7 | 30.4, CH | 40.4, CH_2_ | 40.9, CH_2_ | 37.0, CH_2_ | 36.7, CH_2_ | 34.7, CH_2_ | 29.2, CH | 37.1, CH_2_ | 39.5, CH_2_ | 37.1, CH_2_ | 27.7, CH_2_ |
| 8 | 40.6, CH | 68.9, CH | 67.6, CH | 75.2, CH | 69.5, CH | 70.0, CH | 39.5, CH | 68.5, CH | 71.9, CH | 68.5, CH | 31.6 CH_2_ |
| 9 | 203.0, C | 42.8, CH_2_ | 42.1, CH_2_ | 40.3, CH_2_ | 76.1, CH | 68.8, CH | 201.9, C | 69.5, CH | 70.1, CH | 69.7, CH | 69.1, CH |
| 10 | 134.6, CH | 78.7, CH | 76.1, CH | 75.2, CH | 210.3, C | 79.2, CH | 134.9, CH | 78.4, CH | 77.6, CH | 78.6, CH | 82.5, CH |
| 11 | 150.4, CH | 70.9, CH | 217.4, C | 219.3, C | 46.5, CH_2_ | 68.8, CH | 149.2, CH | 215.3, C | 81.7, CH | 215.0, C | 214.4, C |
| 12 | 36.2, CH_2_ | 69.7, CH | 36.6, CH_2_ | 36.7, CH_2_ | 68.0, CH | 69.7, CH | 34.9, CH_2_ | 37.0, CH_2_ | 216.9, C | 37.1, CH_2_ | 34.2, CH_2_ |
| 13 | 33.8, CH_2_ | 35.3, CH_2_ | 24.8, CH_2_ | 24.7, CH_2_ | 40.7, CH_2_ | 36.2, CH_2_ | 32.5, CH_2_ | 24.7, CH_2_ | 43.7, CH_2_ | 24.9, CH_2_ | 24.6, CH_2_ |
| 14 | 133.4, C | 130.6, C | 130.8, C | 130.6, C | 129.3, C | 131.4, C | 132.3, C | 132.8, C | 129.9, C | 130.7, C | 131.2, C |
| 15 | 129.3, CH | 129.8, CH | 129.3, CH | 129.3, CH | 130.3, CH | 130.3, CH | 129.0, CH | 129.7, CH | 130.1, CH | 129.0, CH | 129.1, CH |
| 16 | 116.4, CH | 117.3, CH | 117.1, CH | 117.2, CH | 117.2, CH | 116.8, CH | 116.3, CH | 115.4, CH | 116.8, CH | 117.1, CH | 116.3, CH |
| 17 | 153.2, C | 153.9, C | 153.1, C | 153.1, C | 153.7, C | 152.8, C | 151.0, C | 152.6, C | 153.1, C | 152.7, CH | 151.0, C |
| 18 | 136.0, CH | 134.5, CH | 134.3, CH | 134.2, CH | 135.1, CH | 135.4, CH | 133.5, CH | 135.9, CH | 136.9, CH | 134.6, CH | 135.3, CH |
| 19 | 134.2, CH | 134.8, CH | 134.3, CH | 134.2, CH | 135.1, CH | 135.2, CH | 133.0, CH | 135.8, CH | 135.6, CH | 134.4, CH | 134.0, CH |
|  | β-Glc (at C-17) |  |  | β-Glc (at C-8) |  |  |  | β-Glc (at C-17) |  |  |  |
| 1 | 97.9, CH |  |  | 102.3, CH |  |  |  | 102.3, CH |  |  |  |
| 2 | 76.0, CH |  |  | 74.9, CH |  |  |  | 74.6, CH |  |  |  |
| 3 | 77.7, CH |  |  | 77.9, CH |  |  |  | 78.0, CH |  |  |  |
| 4 | 71.6, CH |  |  | 72.2, CH |  |  |  | 71.2, CH |  |  |  |
| 5 | 77.7, CH |  |  | 77.7, CH |  |  |  | 77.9, CH |  |  |  |
| 6 | 62.5, CH_2_ |  |  | 63.6, CH_2_ |  |  |  | 62.3, CH_2_ |  |  |  |
|  | α-Ara (at C-3) |  |  |  |  |  |  |  |  |  |  |
| 1 | 93.5, CH |  |  |  |  |  |  |  |  |  |  |
| 2 | 73.7, CH |  |  |  |  |  |  |  |  |  |  |
| 3 | 74.5, CH |  |  |  |  |  |  |  |  |  |  |
| 4 | 72.7, CH |  |  |  |  |  |  |  |  |  |  |
| 5 | 62.7, CH_2_ |  |  |  |  |  |  |  |  |  |  |

**Table S3.** ^13^C NMR Data (150 MHz, CD_3_OD, δ ppm) of oregonin (**17**)

| position | **oregonin (17)** |
| --- | --- |
| 1 | 29.2, CH_2_ |
| 2 | 46.2, CH_2_ |
| 3 | 211.7, C |
| 4 | 48.6, CH_2_ |
| 5 | 76.1, CH |
| 6 | 38.3, CH_2_ |
| 7 | 30.9, CH_2_ |
| 1' | 134.5, C |
| 2' | 116.1, CH |
| 3' | 145.7, C |
| 4' | 144.3, C |
| 5' | 115.9, CH |
| 6' | 120.1, CH |
| 1'' | 134.5, C |
| 2'' | 116.2, CH |
| 3'' | 145.5, C |
| 4'' | 144.1, C |
| 5'' | 115.9, CH |
| 6'' | 120.2, CH |
|  | β-Xyl (at C-5) |
| 1 | 104, CH |
| 2 | 74.8, CH |
| 3 | 77.6, CH |
| 4 | 70.9, CH |
| 5 | 66.9, CH_2_ |

**Table S4.** Retention times (R_t_), Δ ppm, molecular formula, [M-H]^-^, MS/MS values, of compounds occurring in the EtOH extract of C*. avellana* flowers, cultivar “Tonda di Giffoni PGI”, identified by high resolution LC-ESI/LTQOrbitrap/MS/MS^n^ (negative ion mode).

|  | R_t_ (min) | Compound | Δ ppm | Formula | [M-H]^-^ | MSMS |
| --- | --- | --- | --- | --- | --- | --- |
| **33** | 14.85 | quercetin 3-*O*-β-D-galactopyranosyl-(1→2)-β-D-glucopyranoside | 0.53 | C_27_H_30_O_17_ | 625.1003 | 463, 445, 301 |
| **13** | 15.63 | giffonin M | 1.71 | C_19_H_20_O_5_ | 327.1233 | 253, 205, 179 |
| **34** | 15.63 | kaempferol 3-*O*-β-D-glucopyranosyl-(1→2)-β-D-glucopyranoside | 0.21 | C_27_H_30_O_16_ | 609.1451 | 447, 429, 285 |
| **35** | 16.16 | quercetin 3-*O*- β-D-glucopyranoside | -0.20 | C_21_H_20_O_12_ | 463.0870 | 301 |
| **29** | 17.52 | quercetin 3-*O*-α-L-rhamnopyranoside | 0.14 | C_21_H_20_O_11_ | 447.0923 | 301 |
| **9** | 17.57 | giffonin I | 1.42 | C_30_H_36_O_12_ | 587.2126 | 455, 425, 293 |
| **24** | 17.67 | carpinontriol B | 1.50 | C_19_H_20_O_6_ | 343.1181 | 325, 299, 283, 269 |
| **30** | 18.83 | kaempferol 3-*O*- α-L-rhamnopyranoside | 0.25 | C_21_H_20_O_10_ | 431.0974 | 285 |
| **25** | 21.08 | giffonin V | 1.44 | C_18_H_32_O_5_ | 327.1232 | 205, 179, 147, |
| **36** | 22.76 | octadecenoic acid-trihydroxy | 1.18 | C_18_H_34_O_5_ | 329.2326 | 229, 211, 171 |
| **37** | 23.71 | hexadecanoic acid-dihydroxy | 1.27 | C_16_H_32_O_4_ | 287.2220 | 269, 241 |
| **31** | 25.12 | kaempferol 3-*O*-(4''-cis-*p*-coumaroyl)-α-L-rhamnopyranoside | 0.06 | C_30_H_26_O_12_ | 577.1341 | 431, 285 |
| **32** | 25.43 | kaempferol 3-*O*-(4''-trans-*p*-coumaroyl)-α-L-rhamnopyranoside | -2.15 | C_30_H_26_O_12_ | 577.1328 | 431, 285 |
| **21** | 26.74 | Alnusone | 1.46 | C_19_H_18_O_3_ | 293.1176 | 251, 83 |
| **18** | 32.20 | giffonin Q | 0.20 | C_19_H_18_O_3_ | 293.2112 | - |
| **19** | 34.03 | giffonin R | 0.17 | C_19_H_16_O_3_ | 291.1016 | - |
| **20** | 40.59 | giffonin S | 0.25 | C_20_H_17_O_4_ | 321.1120 | - |

**Table S5.** Retention times (R_t_), Δ ppm, molecular formula, [M-H]^-^, MS/MS values, of compounds occurring in the EtOH extract of C*. avellana* shells, cultivar “Tonda di Giffoni PGI”, identified by high resolution LC-ESI/LTQOrbitrap/MS/MS^n^ (negative ion mode).

|  | **R_t_ (min)** | **Compound** | **Δ ppm** | **Formula** | **[M-H]^-^** | **[M+HCOOH]-H]^-^** | **MSMS** |
| --- | --- | --- | --- | --- | --- | --- | --- |
| **38** | 5.68 | 2,3-dihydroxy-1-(4-hydroxy-3-methoxyphenyl)-propan-1-one | 1.99 | C_11_H_14_O_7_ |  | 257.0661 | - |
| **39** | 7.49 | 1-(-4-hydroxy-3-methoxy)-1,2,3-propanetriol | 2.37 | C_11_H_14_O_7_ |  | 257.0662 | - |
| **40** | 8.83 | threo-1,2-bis(4-hydroxy-3-methoxyphenyl)-1,3-propandiol | -0.67 | C_17_H_20_O_6_ | 319.1174 |  | 271 |
| **16** | 13.04 | giffonin P | -0.44 | C_19_H_22_O_7_ | 361.1280 |  | 241, 271, 301, 343 |
| **23** | 14.45 | giffonin U | -0.53 | C_19_H_20_O_7_ | 359.1123 | 341, 329 | 341, 329 |
| **41** | 15.08 | erythro-(7*s*,8*r*)-guaiacylglycerol-*β*-*O*-4'-dihydroconiferyl alcohol | -1.19 | C_20_H_26_O_7_ | 377.1590 |  | 329, 195, 165 |
| **28** | 16.34 | myricetin 3-*O*-α-L-rhamnopyranoside | -1.19 | C_21_H_20_O_12_ | 463.0865 |  | 316 |
| **42** | 16.45 | ceplignan | -0.08 | C_18_H_18_O_7_ | 345.0974 |  | 193, 151, 315, 221 |
| **43** | 16.50 | *ent*-cedrusin | -0.96 | C_20_H_24_O_8_ |  | 391.1384 | 327, 315 |
| **44** | 17.44 | erythro-(7*s*,8*r*)-guaiacylglycerol-*β*-coniferyl aldehyde ether | -0.64 | C_21_H_24_O_9_ |  | 419.1334 | 373, 325, 177, 195 |
| **45** | 17.91 | ficusal | -0.38 | C_19_H_20_O_8_ |  | 375.1073 | 269, 209, 239, 167, 299 |
| **24** | 17.97 | carpinontriol B | 0.27 | C_19_H_20_O_6_ | 343.1177 |  | 269, 283, 299, 313, 325 |
| **30** | 19.33 | kaempferol 3-*O*- α-L-rhamnopyranoside | -1.17 | C_21_H_20_O_10_ | 431.0968 |  | 285 |
| **25** | 21.12 | giffonin V | 0.27 | C_19_H_20_O_5_ | 327.1228 |  | 253, 283, 241, 271 |
| **46** | 22.49 | balanophonin | -0.13 | C_20_H_20_O_6_ | 355.1176 |  | 337, 281, 249, 325, 219 |
| **36** | 23.48 | octadecenoic acid-trihydroxy | 0.61 | C_18_H_34_O_5_ | 329.2325 | 229, 211, 171 | 229, 211, 171 |
| **37** | 23.71 | hexadecanoic acid-dihydroxy | -0.23 | C_16_H_32_O_4_ | 287.2216 | 269, 241 | 269, 241 |
| **31** | 25.90 | kaempferol 3-*O*-(4''-cis-*p*-coumaroyl)-α-L-rhamnopyranoside | -1.82 | C_30_H_26_O_12_ | 577.1130 |  | 431, 285 |
| **32** | 26.22 | kaempferol 3-*O*-(4''-trans-*p*-coumaroyl)-α-L-rhamnopyranoside | -1.09 | C_30_H_26_O_12_ | 577.1334 |  | 431, 285 |
| **47** | 29.20 | dihydrodehydrodiconiferyl alcohol | 1.49 | C_20_H_24_O_6_ | 359.1495 |  | 135, 177, 329, 341 |

**Table S6.** Retention times (R_t_), Δ ppm, molecular formula, [M-H]^-^, MS/MS values, of compounds occurring in the EtOH extract of C*. avellana* green leafy involucres, cultivar “Tonda di Giffoni PGI”, identified by high resolution LC-ESI/LTQOrbitrap/MS/MS^n^ (negative ion mode).

|  | **R_t_ (min)** | **Compound** | **Δ ppm** | **Formula** | **[M-H]^-^** | **MS/MS** |
| --- | --- | --- | --- | --- | --- | --- |
| **48** | 1.52 | citric acid | 4.09 | C_6_H_8_O_7_ | 191.0194 | 173, 123, 85 |
| **49** | 3.25 | 1-methylcitrate | 3.66 | C_7_H_10_O_7_ | 205.0350 |  |
| **50** | 3.62 | trimethylcitrate formiate | 1.36 | C_10_H_16_O_9_ | 279.0714 |  |
| **16** | 12.98 | giffonin P | 0.36 | C_19_H_22_O_7_ | 361.1283 | 343, 301, 271, 241 |
| **14** | 13.40 | giffonin N | -0.87 | C_25_H_30_O_10_ | 489.1751 | 309,281 |
| **28** | 13.72 | myricetin 3-*O*-α-L-rhamnopyranoside | -0.99 | C_21_H_20_O_12_ | 463.0866 | 316 |
| **17** | 13.72 | oregonin | -0.99 | C_24_H_30_O_10_ | 477.1750 | 327 |
| **21** | 16.35 | giffonin T | 0.20 | C_25_H_30_O_11_ | 505.1712 | 343, 325, 299 |
| **24** | 16.86 | carpinontriol B | 0.66 | C_19_H_20_O_6_ | 343.1178 | 299, 283, 269 |
| **30** | 18.70 | kaempferol 3-*O*- α-L-rhamnopyranoside | -0.75 | C_21_H_20_O_10_ | 431.0970 | 285 |
| **9** | 19.85 | giffonin I | -1.95 | C_30_H_36_O_12_ | 587.2426 | 455, 425, 293 |
| **13** | 20.64 | giffonin M | 0.92 | C_19_H_20_O_5_ | 327.123 | 253, 205, 179 |
| **36** | 23.16 | octadecenoic acid-trihydroxy | 1.06 | C_18_H_34_O_5_ | 329.2326 | 229, 211, 171 |
| **31** | 25.05 | kaempferol 3-*O*-(4''-cis-*p*-coumaroyl)-α-L-rhamnopyranoside | -0.37 | C_30_H_26_O_12_ | 577.1342 | 431, 285 |
| **32** | 25.46 | kaempferol 3-*O*-(4''-trans-*p*-coumaroyl)-α-L-rhamnopyranoside | -1.09 | C_30_H_26_O_12_ | 577.1334 | 431, 285 |
| **21** | 27.09 | alnusone | 1.16 | C_19_H_18_O_3_ | 293.1176 | 251, 83 |

**Table S7.** LC–MS/MS conditions for quantitation of compounds **10, 11** and **16** by negative ion MRM mode.

| **Compound** | DP | CE | EP | CXP |
| --- | --- | --- | --- | --- |
| giffonin J (**10**) | -55.2 | -34.0 | -3.48 | -15.01 |
| giffonin K (**11**) | -55.3 | -36.0 | -4.59 | -14.98 |
| giffonin P (**16**) | -55.3 | -36.0 | -4.59 | -14.98 |

**DP**, Declustering Potential; **CE**, Collision energy; **EP**, Entrance potential; **CXP**, Collision Cell Exit Potential.

**Table S8.** 3D coordinates of α-glucosidase protein structure built by homology modeling experiments.

REMARK 4 COMPLIES WITH FORMAT V. 3.0, 1-DEC-2006

REMARK 888

REMARK 888 WRITTEN BY MAESTRO (A PRODUCT OF SCHRODINGER, LLC)

TITLE sp|P38158|MAL32_YEAST - homology model built on

TITLE 2 3a47-isomaltase-sacc-cer_A

MODEL 1

ATOM 1 CH3 ACE A 3 43.061 28.279 15.445 1.00 0.00 C

ATOM 2 C ACE A 3 42.527 27.132 14.597 1.00 0.00 C

ATOM 3 O ACE A 3 43.283 26.230 14.236 1.00 0.00 O

ATOM 4 1H ACE A 3 44.142 28.204 15.563 1.00 0.00 H

ATOM 5 2H ACE A 3 42.833 29.237 14.977 1.00 0.00 H

ATOM 6 3H ACE A 3 42.605 28.262 16.435 1.00 0.00 H

ATOM 7 N SER A 4 41.219 27.177 14.291 1.00 29.56 N

ATOM 8 CA SER A 4 40.519 26.231 13.422 1.00 29.55 C

ATOM 9 C SER A 4 40.943 26.437 11.953 1.00 29.37 C

ATOM 10 O SER A 4 40.366 27.264 11.247 1.00 29.60 O

ATOM 11 CB SER A 4 38.997 26.344 13.677 1.00 29.43 C

ATOM 12 OG SER A 4 38.451 27.585 13.277 1.00 30.83 O

ATOM 13 H1 SER A 4 40.656 27.946 14.628 1.00 0.00 H

ATOM 14 HA SER A 4 40.814 25.223 13.721 1.00 0.00 H

ATOM 15 HB3 SER A 4 38.776 26.202 14.735 1.00 0.00 H

ATOM 16 HB2 SER A 4 38.470 25.552 13.145 1.00 0.00 H

ATOM 17 HG SER A 4 38.626 27.703 12.339 1.00 0.00 H

ATOM 18 N ASP A 5 41.989 25.700 11.544 1.00 31.02 N

ATOM 19 CA ASP A 5 42.689 25.821 10.257 1.00 32.28 C

ATOM 20 C ASP A 5 41.806 25.482 9.025 1.00 31.44 C

ATOM 21 O ASP A 5 42.172 25.824 7.902 1.00 32.07 O

ATOM 22 CB ASP A 5 43.988 24.976 10.325 1.00 0.00 C

ATOM 23 CG ASP A 5 44.951 25.144 9.146 1.00 0.00 C

ATOM 24 OD1 ASP A 5 45.389 26.294 8.929 1.00 0.00 O

ATOM 25 OD2 ASP A 5 45.280 24.109 8.526 1.00 0.00 O1-

ATOM 26 HB2 ASP A 5 44.546 25.211 11.233 1.00 0.00 H

ATOM 27 HB3 ASP A 5 43.737 23.921 10.427 1.00 0.00 H

ATOM 28 H ASP A 5 42.419 25.071 12.207 1.00 0.00 H

ATOM 29 HA ASP A 5 42.973 26.870 10.155 1.00 0.00 H

ATOM 30 N HIS A 6 40.666 24.812 9.271 1.00 31.02 N

ATOM 31 CA HIS A 6 39.674 24.290 8.331 1.00 30.54 C

ATOM 32 C HIS A 6 39.907 22.892 7.687 1.00 30.29 C

ATOM 33 O HIS A 6 39.094 22.562 6.827 1.00 30.82 O

ATOM 34 CB HIS A 6 39.100 25.397 7.390 1.00 0.00 C

ATOM 35 CG HIS A 6 39.403 25.303 5.911 1.00 0.00 C

ATOM 36 ND1 HIS A 6 40.682 25.384 5.392 1.00 0.00 N

ATOM 37 CD2 HIS A 6 38.578 25.091 4.826 1.00 0.00 C

ATOM 38 CE1 HIS A 6 40.585 25.204 4.072 1.00 0.00 C

ATOM 39 NE2 HIS A 6 39.337 25.021 3.657 1.00 0.00 N

ATOM 40 HB2 HIS A 6 38.013 25.389 7.480 1.00 0.00 H

ATOM 41 HB3 HIS A 6 39.397 26.390 7.730 1.00 0.00 H

ATOM 42 HD2 HIS A 6 37.506 24.961 4.798 1.00 0.00 H

ATOM 43 HE1 HIS A 6 41.437 25.205 3.408 1.00 0.00 H

ATOM 44 H HIS A 6 40.460 24.607 10.238 1.00 0.00 H

ATOM 45 HA HIS A 6 38.849 24.075 9.000 1.00 0.00 H

ATOM 46 HD1 HIS A 6 41.530 25.531 5.926 1.00 0.00 H

ATOM 47 N PRO A 7 40.897 22.042 8.082 1.00 27.98 N

ATOM 48 CA PRO A 7 41.030 20.710 7.457 1.00 27.56 C

ATOM 49 C PRO A 7 39.829 19.777 7.744 1.00 27.51 C

ATOM 50 O PRO A 7 39.083 19.506 6.801 1.00 28.08 O

ATOM 51 CD PRO A 7 41.976 22.249 9.051 1.00 0.00 C

ATOM 52 CB PRO A 7 42.406 20.209 7.922 1.00 0.00 C

ATOM 53 CG PRO A 7 42.666 20.913 9.235 1.00 0.00 C

ATOM 54 HD3 PRO A 7 42.688 22.947 8.613 1.00 0.00 H

ATOM 55 HD2 PRO A 7 41.631 22.617 10.017 1.00 0.00 H

ATOM 56 HB2 PRO A 7 42.478 19.134 8.023 1.00 0.00 H

ATOM 57 HB3 PRO A 7 43.161 20.512 7.196 1.00 0.00 H

ATOM 58 HG3 PRO A 7 43.725 21.000 9.479 1.00 0.00 H

ATOM 59 HG2 PRO A 7 42.185 20.377 10.046 1.00 0.00 H

ATOM 60 HA PRO A 7 41.082 20.833 6.373 1.00 0.00 H

ATOM 61 N GLU A 8 39.601 19.356 9.005 1.00 10.50 N

ATOM 62 CA GLU A 8 38.390 18.608 9.382 1.00 10.96 C

ATOM 63 C GLU A 8 37.206 19.530 9.722 1.00 11.02 C

ATOM 64 O GLU A 8 36.064 19.097 9.570 1.00 11.07 O

ATOM 65 CB GLU A 8 38.626 17.569 10.515 1.00 11.16 C

ATOM 66 CG GLU A 8 38.920 18.066 11.953 1.00 11.12 C

ATOM 67 CD GLU A 8 40.369 18.465 12.236 1.00 11.75 C

ATOM 68 OE1 GLU A 8 41.157 18.577 11.276 1.00 10.75 O

ATOM 69 OE2 GLU A 8 40.683 18.626 13.433 1.00 10.31 O1-

ATOM 70 HA GLU A 8 38.080 18.014 8.520 1.00 0.00 H

ATOM 71 HB3 GLU A 8 39.392 16.856 10.218 1.00 0.00 H

ATOM 72 HB2 GLU A 8 37.726 16.959 10.577 1.00 0.00 H

ATOM 73 HG3 GLU A 8 38.688 17.255 12.643 1.00 0.00 H

ATOM 74 HG2 GLU A 8 38.262 18.887 12.235 1.00 0.00 H

ATOM 75 H GLU A 8 40.275 19.525 9.742 1.00 0.00 H

ATOM 76 N THR A 9 37.482 20.763 10.179 1.00 10.52 N

ATOM 77 CA THR A 9 36.464 21.687 10.683 1.00 10.24 C

ATOM 78 C THR A 9 35.646 22.421 9.596 1.00 10.36 C

ATOM 79 O THR A 9 34.695 23.106 9.970 1.00 11.04 O

ATOM 80 CB THR A 9 37.045 22.718 11.690 1.00 10.42 C

ATOM 81 OG1 THR A 9 37.625 23.865 11.105 1.00 10.66 O

ATOM 82 CG2 THR A 9 38.007 22.128 12.729 1.00 10.36 C

ATOM 83 H THR A 9 38.443 21.061 10.271 1.00 0.00 H

ATOM 84 HA THR A 9 35.743 21.091 11.245 1.00 0.00 H

ATOM 85 HB THR A 9 36.189 23.098 12.243 1.00 0.00 H

ATOM 86 HG1 THR A 9 36.925 24.388 10.705 1.00 0.00 H

ATOM 87 HG21 THR A 9 38.273 22.877 13.475 1.00 0.00 H

ATOM 88 HG22 THR A 9 37.557 21.283 13.248 1.00 0.00 H

ATOM 89 HG23 THR A 9 38.934 21.780 12.271 1.00 0.00 H

ATOM 90 N GLU A 10 35.983 22.250 8.298 1.00 10.60 N

ATOM 91 CA GLU A 10 35.199 22.742 7.154 1.00 11.47 C

ATOM 92 C GLU A 10 33.769 22.154 7.179 1.00 10.47 C

ATOM 93 O GLU A 10 33.651 20.936 7.029 1.00 10.28 O

ATOM 94 CB GLU A 10 35.910 22.361 5.834 1.00 11.36 C

ATOM 95 CG GLU A 10 35.139 22.788 4.559 1.00 14.10 C

ATOM 96 CD GLU A 10 35.822 22.415 3.241 1.00 14.98 C

ATOM 97 OE1 GLU A 10 35.169 22.642 2.198 1.00 20.25 O

ATOM 98 OE2 GLU A 10 36.957 21.892 3.282 1.00 18.60 O1-

ATOM 99 H GLU A 10 36.789 21.687 8.068 1.00 0.00 H

ATOM 100 HA GLU A 10 35.203 23.828 7.195 1.00 0.00 H

ATOM 101 HB3 GLU A 10 36.109 21.288 5.811 1.00 0.00 H

ATOM 102 HB2 GLU A 10 36.880 22.849 5.813 1.00 0.00 H

ATOM 103 HG3 GLU A 10 34.989 23.868 4.568 1.00 0.00 H

ATOM 104 HG2 GLU A 10 34.147 22.337 4.530 1.00 0.00 H

ATOM 105 N PRO A 11 32.724 22.992 7.379 1.00 10.30 N

ATOM 106 CA PRO A 11 31.332 22.513 7.412 1.00 9.87 C

ATOM 107 C PRO A 11 30.846 21.870 6.104 1.00 9.52 C

ATOM 108 O PRO A 11 31.035 22.444 5.032 1.00 10.42 O

ATOM 109 CB PRO A 11 30.502 23.761 7.764 1.00 10.09 C

ATOM 110 CG PRO A 11 31.486 24.711 8.418 1.00 11.38 C

ATOM 111 CD PRO A 11 32.778 24.428 7.668 1.00 10.59 C

ATOM 112 HA PRO A 11 31.277 21.796 8.226 1.00 0.00 H

ATOM 113 HB3 PRO A 11 29.659 23.529 8.417 1.00 0.00 H

ATOM 114 HB2 PRO A 11 30.098 24.232 6.866 1.00 0.00 H

ATOM 115 HG3 PRO A 11 31.609 24.436 9.466 1.00 0.00 H

ATOM 116 HG2 PRO A 11 31.177 25.755 8.376 1.00 0.00 H

ATOM 117 HD2 PRO A 11 32.812 24.974 6.724 1.00 0.00 H

ATOM 118 HD3 PRO A 11 33.624 24.742 8.278 1.00 0.00 H

ATOM 119 N LYS A 12 30.210 20.702 6.247 1.00 8.54 N

ATOM 120 CA LYS A 12 29.418 20.030 5.217 1.00 7.89 C

ATOM 121 C LYS A 12 28.094 19.612 5.871 1.00 7.60 C

ATOM 122 O LYS A 12 28.071 19.415 7.088 1.00 6.59 O

ATOM 123 CB LYS A 12 30.151 18.772 4.707 1.00 8.12 C

ATOM 124 CG LYS A 12 31.563 18.990 4.135 1.00 9.44 C

ATOM 125 CD LYS A 12 31.577 19.827 2.850 1.00 12.73 C

ATOM 126 CE LYS A 12 32.947 19.824 2.165 1.00 15.64 C

ATOM 127 NZ LYS A 12 32.918 20.604 0.918 1.00 19.14 N1+

ATOM 128 H LYS A 12 30.102 20.319 7.177 1.00 0.00 H

ATOM 129 HA LYS A 12 29.200 20.704 4.387 1.00 0.00 H

ATOM 130 HB3 LYS A 12 29.539 18.293 3.943 1.00 0.00 H

ATOM 131 HB2 LYS A 12 30.216 18.053 5.517 1.00 0.00 H

ATOM 132 HG3 LYS A 12 31.999 18.015 3.925 1.00 0.00 H

ATOM 133 HG2 LYS A 12 32.211 19.447 4.883 1.00 0.00 H

ATOM 134 HD3 LYS A 12 31.293 20.853 3.071 1.00 0.00 H

ATOM 135 HD2 LYS A 12 30.820 19.453 2.167 1.00 0.00 H

ATOM 136 HE3 LYS A 12 33.250 18.804 1.928 1.00 0.00 H

ATOM 137 HE2 LYS A 12 33.705 20.241 2.829 1.00 0.00 H

ATOM 138 HZ1 LYS A 12 32.235 20.207 0.288 1.00 0.00 H

ATOM 139 HZ2 LYS A 12 32.674 21.561 1.129 1.00 0.00 H

ATOM 140 HZ3 LYS A 12 33.833 20.587 0.491 1.00 0.00 H

ATOM 141 N TRP A 13 27.025 19.438 5.067 1.00 7.07 N

ATOM 142 CA TRP A 13 25.702 19.025 5.567 1.00 7.01 C

ATOM 143 C TRP A 13 25.752 17.699 6.341 1.00 6.57 C

ATOM 144 O TRP A 13 25.118 17.586 7.386 1.00 7.19 O

ATOM 145 CB TRP A 13 24.651 18.964 4.427 1.00 7.46 C

ATOM 146 CG TRP A 13 24.695 17.819 3.444 1.00 7.65 C

ATOM 147 CD1 TRP A 13 25.232 17.873 2.203 1.00 8.54 C

ATOM 148 CD2 TRP A 13 24.234 16.435 3.613 1.00 7.82 C

ATOM 149 NE1 TRP A 13 25.115 16.643 1.591 1.00 8.75 N

ATOM 150 CE2 TRP A 13 24.530 15.710 2.420 1.00 8.20 C

ATOM 151 CE3 TRP A 13 23.612 15.705 4.657 1.00 8.18 C

ATOM 152 CZ2 TRP A 13 24.240 14.343 2.274 1.00 8.54 C

ATOM 153 CZ3 TRP A 13 23.323 14.330 4.527 1.00 7.84 C

ATOM 154 CH2 TRP A 13 23.634 13.647 3.336 1.00 8.19 C

ATOM 155 H TRP A 13 27.106 19.596 4.073 1.00 0.00 H

ATOM 156 HA TRP A 13 25.387 19.806 6.262 1.00 0.00 H

ATOM 157 HB3 TRP A 13 24.661 19.903 3.872 1.00 0.00 H

ATOM 158 HB2 TRP A 13 23.663 18.917 4.886 1.00 0.00 H

ATOM 159 HD1 TRP A 13 25.680 18.755 1.768 1.00 0.00 H

ATOM 160 HE1 TRP A 13 25.453 16.457 0.655 1.00 0.00 H

ATOM 161 HE3 TRP A 13 23.362 16.207 5.579 1.00 0.00 H

ATOM 162 HZ2 TRP A 13 24.496 13.836 1.357 1.00 0.00 H

ATOM 163 HZ3 TRP A 13 22.857 13.799 5.345 1.00 0.00 H

ATOM 164 HH2 TRP A 13 23.412 12.595 3.236 1.00 0.00 H

ATOM 165 N TRP A 14 26.524 16.743 5.803 1.00 6.54 N

ATOM 166 CA TRP A 14 26.644 15.382 6.300 1.00 6.23 C

ATOM 167 C TRP A 14 27.513 15.247 7.554 1.00 6.25 C

ATOM 168 O TRP A 14 27.295 14.296 8.299 1.00 6.64 O

ATOM 169 CB TRP A 14 27.125 14.466 5.167 1.00 6.70 C

ATOM 170 CG TRP A 14 28.336 14.877 4.380 1.00 6.80 C

ATOM 171 CD1 TRP A 14 28.315 15.575 3.222 1.00 7.02 C

ATOM 172 CD2 TRP A 14 29.747 14.666 4.689 1.00 7.12 C

ATOM 173 NE1 TRP A 14 29.605 15.785 2.784 1.00 7.31 N

ATOM 174 CE2 TRP A 14 30.532 15.248 3.648 1.00 7.11 C

ATOM 175 CE3 TRP A 14 30.450 14.032 5.737 1.00 7.33 C

ATOM 176 CZ2 TRP A 14 31.936 15.208 3.646 1.00 8.31 C

ATOM 177 CZ3 TRP A 14 31.858 13.978 5.743 1.00 7.19 C

ATOM 178 CH2 TRP A 14 32.604 14.568 4.705 1.00 6.79 C

ATOM 179 H TRP A 14 27.024 16.941 4.948 1.00 0.00 H

ATOM 180 HA TRP A 14 25.651 15.034 6.591 1.00 0.00 H

ATOM 181 HB3 TRP A 14 26.309 14.327 4.461 1.00 0.00 H

ATOM 182 HB2 TRP A 14 27.325 13.486 5.587 1.00 0.00 H

ATOM 183 HD1 TRP A 14 27.419 15.906 2.723 1.00 0.00 H

ATOM 184 HE1 TRP A 14 29.827 16.300 1.936 1.00 0.00 H

ATOM 185 HE3 TRP A 14 29.897 13.577 6.545 1.00 0.00 H

ATOM 186 HZ2 TRP A 14 32.495 15.662 2.841 1.00 0.00 H

ATOM 187 HZ3 TRP A 14 32.367 13.467 6.541 1.00 0.00 H

ATOM 188 HH2 TRP A 14 33.683 14.518 4.718 1.00 0.00 H

ATOM 189 N LYS A 15 28.442 16.192 7.780 1.00 6.44 N

ATOM 190 CA LYS A 15 29.276 16.246 8.980 1.00 5.96 C

ATOM 191 C LYS A 15 28.471 16.560 10.251 1.00 6.16 C

ATOM 192 O LYS A 15 28.683 15.900 11.268 1.00 6.06 O

ATOM 193 CB LYS A 15 30.414 17.269 8.790 1.00 6.34 C

ATOM 194 CG LYS A 15 31.520 16.809 7.829 1.00 5.68 C

ATOM 195 CD LYS A 15 32.701 17.788 7.805 1.00 6.38 C

ATOM 196 CE LYS A 15 33.843 17.351 6.881 1.00 7.76 C

ATOM 197 NZ LYS A 15 34.971 18.296 6.951 1.00 8.32 N1+

ATOM 198 H LYS A 15 28.549 16.953 7.125 1.00 0.00 H

ATOM 199 HA LYS A 15 29.719 15.259 9.106 1.00 0.00 H

ATOM 200 HB3 LYS A 15 30.879 17.457 9.758 1.00 0.00 H

ATOM 201 HB2 LYS A 15 30.013 18.229 8.466 1.00 0.00 H

ATOM 202 HG3 LYS A 15 31.122 16.674 6.828 1.00 0.00 H

ATOM 203 HG2 LYS A 15 31.873 15.826 8.118 1.00 0.00 H

ATOM 204 HD3 LYS A 15 33.091 17.904 8.816 1.00 0.00 H

ATOM 205 HD2 LYS A 15 32.334 18.767 7.496 1.00 0.00 H

ATOM 206 HE3 LYS A 15 33.495 17.287 5.850 1.00 0.00 H

ATOM 207 HE2 LYS A 15 34.200 16.362 7.164 1.00 0.00 H

ATOM 208 HZ1 LYS A 15 35.325 18.336 7.899 1.00 0.00 H

ATOM 209 HZ2 LYS A 15 35.709 18.003 6.328 1.00 0.00 H

ATOM 210 HZ3 LYS A 15 34.649 19.219 6.688 1.00 0.00 H

ATOM 211 N GLU A 16 27.565 17.549 10.158 1.00 6.41 N

ATOM 212 CA GLU A 16 26.728 18.019 11.264 1.00 6.56 C

ATOM 213 C GLU A 16 25.310 17.435 11.276 1.00 6.66 C

ATOM 214 O GLU A 16 24.585 17.703 12.235 1.00 7.49 O

ATOM 215 CB GLU A 16 26.717 19.561 11.291 1.00 6.11 C

ATOM 216 CG GLU A 16 26.157 20.252 10.034 1.00 7.09 C

ATOM 217 CD GLU A 16 26.246 21.768 10.177 1.00 8.23 C

ATOM 218 OE1 GLU A 16 27.360 22.300 9.981 1.00 10.00 O

ATOM 219 OE2 GLU A 16 25.207 22.370 10.525 1.00 10.52 O1-

ATOM 220 H GLU A 16 27.456 18.039 9.280 1.00 0.00 H

ATOM 221 HA GLU A 16 27.162 17.681 12.204 1.00 0.00 H

ATOM 222 HB3 GLU A 16 27.728 19.915 11.484 1.00 0.00 H

ATOM 223 HB2 GLU A 16 26.132 19.899 12.147 1.00 0.00 H

ATOM 224 HG3 GLU A 16 25.119 19.965 9.863 1.00 0.00 H

ATOM 225 HG2 GLU A 16 26.713 19.952 9.149 1.00 0.00 H

ATOM 226 N ALA A 17 24.937 16.639 10.259 1.00 7.21 N

ATOM 227 CA ALA A 17 23.669 15.916 10.240 1.00 7.13 C

ATOM 228 C ALA A 17 23.663 14.791 11.280 1.00 7.30 C

ATOM 229 O ALA A 17 24.539 13.928 11.249 1.00 8.27 O

ATOM 230 CB ALA A 17 23.389 15.343 8.841 1.00 8.18 C

ATOM 231 H ALA A 17 25.573 16.460 9.496 1.00 0.00 H

ATOM 232 HA ALA A 17 22.879 16.629 10.472 1.00 0.00 H

ATOM 233 HB1 ALA A 17 22.524 14.678 8.851 1.00 0.00 H

ATOM 234 HB2 ALA A 17 23.164 16.135 8.128 1.00 0.00 H

ATOM 235 HB3 ALA A 17 24.237 14.772 8.464 1.00 0.00 H

ATOM 236 N THR A 18 22.646 14.806 12.152 1.00 6.87 N

ATOM 237 CA THR A 18 22.310 13.671 13.001 1.00 7.15 C

ATOM 238 C THR A 18 21.456 12.709 12.161 1.00 6.43 C

ATOM 239 O THR A 18 20.364 13.094 11.745 1.00 7.10 O

ATOM 240 CB THR A 18 21.490 14.111 14.240 1.00 7.31 C

ATOM 241 OG1 THR A 18 22.291 14.965 15.034 1.00 8.19 O

ATOM 242 CG2 THR A 18 21.003 12.957 15.135 1.00 8.98 C

ATOM 243 H THR A 18 21.980 15.571 12.137 1.00 0.00 H

ATOM 244 HA THR A 18 23.219 13.172 13.341 1.00 0.00 H

ATOM 245 HB THR A 18 20.627 14.697 13.921 1.00 0.00 H

ATOM 246 HG1 THR A 18 22.482 15.754 14.521 1.00 0.00 H

ATOM 247 HG21 THR A 18 20.419 13.347 15.966 1.00 0.00 H

ATOM 248 HG22 THR A 18 20.355 12.259 14.605 1.00 0.00 H

ATOM 249 HG23 THR A 18 21.839 12.388 15.544 1.00 0.00 H

ATOM 250 N ILE A 19 21.976 11.499 11.907 1.00 6.44 N

ATOM 251 CA ILE A 19 21.256 10.445 11.198 1.00 6.28 C

ATOM 252 C ILE A 19 20.409 9.610 12.171 1.00 6.55 C

ATOM 253 O ILE A 19 20.847 9.341 13.289 1.00 7.29 O

ATOM 254 CB ILE A 19 22.203 9.542 10.342 1.00 0.00 C

ATOM 255 CG1 ILE A 19 22.571 10.305 9.047 1.00 0.00 C

ATOM 256 CG2 ILE A 19 21.639 8.144 9.974 1.00 0.00 C

ATOM 257 CD1 ILE A 19 23.794 9.786 8.286 1.00 0.00 C

ATOM 258 HB ILE A 19 23.122 9.387 10.909 1.00 0.00 H

ATOM 259 HG12 ILE A 19 21.714 10.302 8.373 1.00 0.00 H

ATOM 260 HG13 ILE A 19 22.768 11.351 9.286 1.00 0.00 H

ATOM 261 HG21 ILE A 19 22.306 7.595 9.314 1.00 0.00 H

ATOM 262 HG22 ILE A 19 21.494 7.512 10.850 1.00 0.00 H

ATOM 263 HG23 ILE A 19 20.684 8.228 9.455 1.00 0.00 H

ATOM 264 HD11 ILE A 19 23.965 10.375 7.385 1.00 0.00 H

ATOM 265 HD12 ILE A 19 24.689 9.870 8.900 1.00 0.00 H

ATOM 266 HD13 ILE A 19 23.680 8.749 7.976 1.00 0.00 H

ATOM 267 H ILE A 19 22.885 11.252 12.280 1.00 0.00 H

ATOM 268 HA ILE A 19 20.582 10.930 10.500 1.00 0.00 H

ATOM 269 N TYR A 20 19.227 9.193 11.696 1.00 5.66 N

ATOM 270 CA TYR A 20 18.393 8.196 12.350 1.00 5.22 C

ATOM 271 C TYR A 20 18.363 6.942 11.483 1.00 5.12 C

ATOM 272 O TYR A 20 17.884 7.009 10.350 1.00 5.25 O

ATOM 273 CB TYR A 20 16.986 8.751 12.558 1.00 5.77 C

ATOM 274 CG TYR A 20 16.087 7.924 13.450 1.00 5.75 C

ATOM 275 CD1 TYR A 20 16.181 8.052 14.850 1.00 6.80 C

ATOM 276 CD2 TYR A 20 15.140 7.049 12.887 1.00 7.06 C

ATOM 277 CE1 TYR A 20 15.302 7.335 15.680 1.00 5.59 C

ATOM 278 CE2 TYR A 20 14.272 6.320 13.718 1.00 7.06 C

ATOM 279 CZ TYR A 20 14.335 6.480 15.115 1.00 5.70 C

ATOM 280 OH TYR A 20 13.453 5.816 15.913 1.00 5.76 O

ATOM 281 H TYR A 20 18.923 9.497 10.781 1.00 0.00 H

ATOM 282 HA TYR A 20 18.794 7.967 13.329 1.00 0.00 H

ATOM 283 HB3 TYR A 20 16.488 8.939 11.608 1.00 0.00 H

ATOM 284 HB2 TYR A 20 17.095 9.709 13.040 1.00 0.00 H

ATOM 285 HD1 TYR A 20 16.913 8.714 15.290 1.00 0.00 H

ATOM 286 HD2 TYR A 20 15.069 6.954 11.815 1.00 0.00 H

ATOM 287 HE1 TYR A 20 15.373 7.461 16.747 1.00 0.00 H

ATOM 288 HE2 TYR A 20 13.545 5.658 13.275 1.00 0.00 H

ATOM 289 HH TYR A 20 13.518 6.058 16.848 1.00 0.00 H

ATOM 290 N GLN A 21 18.854 5.821 12.029 1.00 4.45 N

ATOM 291 CA GLN A 21 18.779 4.543 11.338 1.00 4.40 C

ATOM 292 C GLN A 21 17.428 3.859 11.598 1.00 4.80 C

ATOM 293 O GLN A 21 17.046 3.658 12.750 1.00 5.48 O

ATOM 294 CB GLN A 21 19.978 3.652 11.678 1.00 4.06 C

ATOM 295 CG GLN A 21 19.911 2.288 10.952 1.00 5.20 C

ATOM 296 CD GLN A 21 21.013 1.324 11.357 1.00 4.38 C

ATOM 297 OE1 GLN A 21 21.858 1.628 12.193 1.00 5.13 O

ATOM 298 NE2 GLN A 21 20.991 0.139 10.759 1.00 4.85 N

ATOM 299 H GLN A 21 19.208 5.821 12.977 1.00 0.00 H

ATOM 300 HA GLN A 21 18.881 4.738 10.275 1.00 0.00 H

ATOM 301 HB3 GLN A 21 20.040 3.526 12.756 1.00 0.00 H

ATOM 302 HB2 GLN A 21 20.895 4.166 11.388 1.00 0.00 H

ATOM 303 HG3 GLN A 21 19.947 2.439 9.872 1.00 0.00 H

ATOM 304 HG2 GLN A 21 18.977 1.769 11.164 1.00 0.00 H

ATOM 305 HE22 GLN A 21 21.706 -0.552 10.942 1.00 0.00 H

ATOM 306 HE21 GLN A 21 20.285 -0.071 10.067 1.00 0.00 H

ATOM 307 N ILE A 22 16.777 3.483 10.492 1.00 5.11 N

ATOM 308 CA ILE A 22 15.515 2.762 10.439 1.00 5.64 C

ATOM 309 C ILE A 22 15.797 1.343 9.916 1.00 5.50 C

ATOM 310 O ILE A 22 16.436 1.191 8.875 1.00 6.24 O

ATOM 311 CB ILE A 22 14.529 3.476 9.463 1.00 5.47 C

ATOM 312 CG1 ILE A 22 14.189 4.891 9.985 1.00 6.42 C

ATOM 313 CG2 ILE A 22 13.233 2.683 9.191 1.00 5.27 C

ATOM 314 CD1 ILE A 22 13.431 5.787 8.996 1.00 7.06 C

ATOM 315 H ILE A 22 17.191 3.691 9.591 1.00 0.00 H

ATOM 316 HA ILE A 22 15.060 2.705 11.430 1.00 0.00 H

ATOM 317 HB ILE A 22 15.035 3.601 8.505 1.00 0.00 H

ATOM 318 HG13 ILE A 22 15.108 5.414 10.244 1.00 0.00 H

ATOM 319 HG12 ILE A 22 13.619 4.810 10.912 1.00 0.00 H

ATOM 320 HG21 ILE A 22 12.562 3.239 8.541 1.00 0.00 H

ATOM 321 HG22 ILE A 22 13.414 1.727 8.701 1.00 0.00 H

ATOM 322 HG23 ILE A 22 12.705 2.494 10.121 1.00 0.00 H

ATOM 323 HD11 ILE A 22 13.928 6.751 8.888 1.00 0.00 H

ATOM 324 HD12 ILE A 22 13.357 5.348 8.002 1.00 0.00 H

ATOM 325 HD13 ILE A 22 12.422 5.981 9.357 1.00 0.00 H

ATOM 326 N TYR A 23 15.275 0.340 10.635 1.00 5.79 N

ATOM 327 CA TYR A 23 15.241 -1.055 10.211 1.00 5.31 C

ATOM 328 C TYR A 23 13.806 -1.315 9.715 1.00 5.67 C

ATOM 329 O TYR A 23 12.901 -1.311 10.551 1.00 5.46 O

ATOM 330 CB TYR A 23 15.622 -1.945 11.409 1.00 6.37 C

ATOM 331 CG TYR A 23 15.961 -3.378 11.040 1.00 5.35 C

ATOM 332 CD1 TYR A 23 17.198 -3.661 10.427 1.00 5.67 C

ATOM 333 CD2 TYR A 23 15.068 -4.432 11.319 1.00 5.89 C

ATOM 334 CE1 TYR A 23 17.559 -4.989 10.135 1.00 5.46 C

ATOM 335 CE2 TYR A 23 15.432 -5.763 11.039 1.00 6.06 C

ATOM 336 CZ TYR A 23 16.685 -6.042 10.463 1.00 5.69 C

ATOM 337 OH TYR A 23 17.053 -7.332 10.235 1.00 6.88 O

ATOM 338 H TYR A 23 14.749 0.553 11.474 1.00 0.00 H

ATOM 339 HA TYR A 23 15.978 -1.228 9.436 1.00 0.00 H

ATOM 340 HB3 TYR A 23 14.854 -1.930 12.184 1.00 0.00 H

ATOM 341 HB2 TYR A 23 16.515 -1.533 11.880 1.00 0.00 H

ATOM 342 HD1 TYR A 23 17.883 -2.859 10.199 1.00 0.00 H

ATOM 343 HD2 TYR A 23 14.108 -4.229 11.757 1.00 0.00 H

ATOM 344 HE1 TYR A 23 18.516 -5.197 9.679 1.00 0.00 H

ATOM 345 HE2 TYR A 23 14.753 -6.568 11.276 1.00 0.00 H

ATOM 346 HH TYR A 23 17.920 -7.415 9.831 1.00 0.00 H

ATOM 347 N PRO A 24 13.594 -1.443 8.381 1.00 5.72 N

ATOM 348 CA PRO A 24 12.255 -1.327 7.780 1.00 6.03 C

ATOM 349 C PRO A 24 11.257 -2.404 8.219 1.00 6.17 C

ATOM 350 O PRO A 24 10.088 -2.067 8.363 1.00 6.43 O

ATOM 351 CB PRO A 24 12.507 -1.348 6.263 1.00 6.75 C

ATOM 352 CG PRO A 24 13.813 -2.104 6.113 1.00 5.75 C

ATOM 353 CD PRO A 24 14.598 -1.658 7.341 1.00 5.55 C

ATOM 354 HA PRO A 24 11.830 -0.358 8.052 1.00 0.00 H

ATOM 355 HB3 PRO A 24 12.635 -0.325 5.904 1.00 0.00 H

ATOM 356 HB2 PRO A 24 11.698 -1.797 5.684 1.00 0.00 H

ATOM 357 HG3 PRO A 24 14.320 -1.909 5.169 1.00 0.00 H

ATOM 358 HG2 PRO A 24 13.623 -3.176 6.173 1.00 0.00 H

ATOM 359 HD2 PRO A 24 15.330 -2.417 7.619 1.00 0.00 H

ATOM 360 HD3 PRO A 24 15.101 -0.712 7.140 1.00 0.00 H

ATOM 361 N ALA A 25 11.731 -3.635 8.488 1.00 5.69 N

ATOM 362 CA ALA A 25 10.927 -4.739 9.019 1.00 6.21 C

ATOM 363 C ALA A 25 10.316 -4.474 10.407 1.00 6.06 C

ATOM 364 O ALA A 25 9.283 -5.065 10.709 1.00 6.24 O

ATOM 365 CB ALA A 25 11.790 -6.009 9.082 1.00 6.13 C

ATOM 366 H ALA A 25 12.710 -3.833 8.341 1.00 0.00 H

ATOM 367 HA ALA A 25 10.102 -4.914 8.327 1.00 0.00 H

ATOM 368 HB1 ALA A 25 11.206 -6.867 9.418 1.00 0.00 H

ATOM 369 HB2 ALA A 25 12.199 -6.266 8.107 1.00 0.00 H

ATOM 370 HB3 ALA A 25 12.628 -5.891 9.771 1.00 0.00 H

ATOM 371 N SER A 26 10.962 -3.625 11.221 1.00 5.90 N

ATOM 372 CA SER A 26 10.661 -3.475 12.645 1.00 6.32 C

ATOM 373 C SER A 26 10.229 -2.061 13.045 1.00 6.65 C

ATOM 374 O SER A 26 9.947 -1.868 14.226 1.00 8.03 O

ATOM 375 CB SER A 26 11.911 -3.884 13.444 1.00 6.15 C

ATOM 376 OG SER A 26 12.140 -5.259 13.244 1.00 6.33 O

ATOM 377 H SER A 26 11.789 -3.148 10.889 1.00 0.00 H

ATOM 378 HA SER A 26 9.840 -4.130 12.943 1.00 0.00 H

ATOM 379 HB3 SER A 26 11.793 -3.721 14.514 1.00 0.00 H

ATOM 380 HB2 SER A 26 12.779 -3.299 13.145 1.00 0.00 H

ATOM 381 HG SER A 26 12.665 -5.601 13.977 1.00 0.00 H

ATOM 382 N PHE A 27 10.193 -1.091 12.114 1.00 6.30 N

ATOM 383 CA PHE A 27 9.919 0.300 12.475 1.00 6.44 C

ATOM 384 C PHE A 27 8.425 0.585 12.673 1.00 6.73 C

ATOM 385 O PHE A 27 8.051 0.991 13.768 1.00 6.56 O

ATOM 386 CB PHE A 27 10.580 1.281 11.497 1.00 6.67 C

ATOM 387 CG PHE A 27 10.587 2.721 11.992 1.00 6.11 C

ATOM 388 CD1 PHE A 27 11.211 3.051 13.215 1.00 7.26 C

ATOM 389 CD2 PHE A 27 9.964 3.744 11.249 1.00 6.96 C

ATOM 390 CE1 PHE A 27 11.209 4.361 13.667 1.00 6.99 C

ATOM 391 CE2 PHE A 27 10.009 5.056 11.700 1.00 8.06 C

ATOM 392 CZ PHE A 27 10.626 5.363 12.906 1.00 7.51 C

ATOM 393 H PHE A 27 10.437 -1.286 11.153 1.00 0.00 H

ATOM 394 HA PHE A 27 10.399 0.469 13.440 1.00 0.00 H

ATOM 395 HB3 PHE A 27 10.114 1.219 10.512 1.00 0.00 H

ATOM 396 HB2 PHE A 27 11.615 0.982 11.349 1.00 0.00 H

ATOM 397 HD1 PHE A 27 11.682 2.291 13.818 1.00 0.00 H

ATOM 398 HD2 PHE A 27 9.455 3.511 10.328 1.00 0.00 H

ATOM 399 HE1 PHE A 27 11.668 4.597 14.614 1.00 0.00 H

ATOM 400 HE2 PHE A 27 9.564 5.841 11.109 1.00 0.00 H

ATOM 401 HZ PHE A 27 10.647 6.385 13.256 1.00 0.00 H

ATOM 402 N LYS A 28 7.591 0.368 11.643 1.00 6.48 N

ATOM 403 CA LYS A 28 6.152 0.609 11.742 1.00 6.80 C

ATOM 404 C LYS A 28 5.423 -0.135 10.621 1.00 6.34 C

ATOM 405 O LYS A 28 5.732 0.086 9.451 1.00 6.89 O

ATOM 406 CB LYS A 28 5.854 2.129 11.723 1.00 6.55 C

ATOM 407 CG LYS A 28 4.376 2.506 11.944 1.00 7.82 C

ATOM 408 CD LYS A 28 3.938 2.340 13.409 1.00 10.35 C

ATOM 409 CE LYS A 28 2.438 2.567 13.635 1.00 12.97 C

ATOM 410 NZ LYS A 28 1.656 1.350 13.358 1.00 15.61 N1+

ATOM 411 H LYS A 28 7.936 0.025 10.758 1.00 0.00 H

ATOM 412 HA LYS A 28 5.804 0.198 12.690 1.00 0.00 H

ATOM 413 HB3 LYS A 28 6.195 2.529 10.771 1.00 0.00 H

ATOM 414 HB2 LYS A 28 6.448 2.648 12.476 1.00 0.00 H

ATOM 415 HG3 LYS A 28 3.725 1.930 11.286 1.00 0.00 H

ATOM 416 HG2 LYS A 28 4.235 3.547 11.653 1.00 0.00 H

ATOM 417 HD3 LYS A 28 4.492 3.054 14.016 1.00 0.00 H

ATOM 418 HD2 LYS A 28 4.221 1.359 13.790 1.00 0.00 H

ATOM 419 HE3 LYS A 28 2.071 3.395 13.028 1.00 0.00 H

ATOM 420 HE2 LYS A 28 2.264 2.838 14.677 1.00 0.00 H

ATOM 421 HZ1 LYS A 28 1.784 1.056 12.397 1.00 0.00 H

ATOM 422 HZ2 LYS A 28 1.972 0.606 13.966 1.00 0.00 H

ATOM 423 HZ3 LYS A 28 0.677 1.523 13.528 1.00 0.00 H

ATOM 424 N ASP A 29 4.455 -0.977 11.012 1.00 7.02 N

ATOM 425 CA ASP A 29 3.506 -1.671 10.139 1.00 7.53 C

ATOM 426 C ASP A 29 2.268 -0.771 9.983 1.00 8.46 C

ATOM 427 O ASP A 29 1.756 -0.277 10.989 1.00 10.23 O

ATOM 428 CB ASP A 29 3.138 -3.034 10.774 1.00 7.28 C

ATOM 429 CG ASP A 29 2.172 -3.940 10.002 1.00 7.28 C

ATOM 430 OD1 ASP A 29 1.845 -3.623 8.841 1.00 8.76 O

ATOM 431 OD2 ASP A 29 1.761 -4.954 10.607 1.00 9.13 O1-

ATOM 432 H ASP A 29 4.279 -1.098 12.001 1.00 0.00 H

ATOM 433 HA ASP A 29 3.952 -1.842 9.158 1.00 0.00 H

ATOM 434 HB3 ASP A 29 2.656 -2.823 11.725 1.00 0.00 H

ATOM 435 HB2 ASP A 29 4.052 -3.598 10.966 1.00 0.00 H

ATOM 436 N SER A 30 1.802 -0.597 8.738 1.00 8.55 N

ATOM 437 CA SER A 30 0.609 0.190 8.413 1.00 9.04 C

ATOM 438 C SER A 30 -0.565 -0.659 7.890 1.00 9.38 C

ATOM 439 O SER A 30 -1.653 -0.101 7.746 1.00 10.17 O

ATOM 440 CB SER A 30 0.998 1.297 7.413 1.00 8.94 C

ATOM 441 OG SER A 30 1.188 0.797 6.105 1.00 10.27 O

ATOM 442 H SER A 30 2.265 -1.058 7.962 1.00 0.00 H

ATOM 443 HA SER A 30 0.241 0.692 9.309 1.00 0.00 H

ATOM 444 HB3 SER A 30 1.902 1.810 7.739 1.00 0.00 H

ATOM 445 HB2 SER A 30 0.212 2.051 7.368 1.00 0.00 H

ATOM 446 HG SER A 30 2.059 0.379 6.066 1.00 0.00 H

ATOM 447 N ASN A 31 -0.339 -1.950 7.590 1.00 9.02 N

ATOM 448 CA ASN A 31 -1.293 -2.796 6.861 1.00 9.61 C

ATOM 449 C ASN A 31 -1.549 -4.163 7.519 1.00 10.04 C

ATOM 450 O ASN A 31 -2.204 -5.004 6.903 1.00 10.66 O

ATOM 451 CB ASN A 31 -0.897 -2.879 5.365 1.00 9.82 C

ATOM 452 CG ASN A 31 0.394 -3.648 5.051 1.00 9.11 C

ATOM 453 OD1 ASN A 31 1.098 -4.111 5.941 1.00 9.57 O

ATOM 454 ND2 ASN A 31 0.713 -3.786 3.764 1.00 12.10 N

ATOM 455 H ASN A 31 0.573 -2.361 7.763 1.00 0.00 H

ATOM 456 HA ASN A 31 -2.285 -2.342 6.889 1.00 0.00 H

ATOM 457 HB3 ASN A 31 -0.786 -1.869 4.968 1.00 0.00 H

ATOM 458 HB2 ASN A 31 -1.708 -3.338 4.799 1.00 0.00 H

ATOM 459 HD22 ASN A 31 1.557 -4.285 3.519 1.00 0.00 H

ATOM 460 HD21 ASN A 31 0.133 -3.396 3.038 1.00 0.00 H

ATOM 461 N ASN A 32 -1.114 -4.329 8.778 1.00 10.17 N

ATOM 462 CA ASN A 32 -1.505 -5.411 9.686 1.00 11.15 C

ATOM 463 C ASN A 32 -0.919 -6.801 9.329 1.00 11.03 C

ATOM 464 O ASN A 32 -1.480 -7.800 9.781 1.00 11.86 O

ATOM 465 CB ASN A 32 -3.059 -5.500 9.854 1.00 0.00 C

ATOM 466 CG ASN A 32 -3.805 -4.170 10.011 1.00 0.00 C

ATOM 467 OD1 ASN A 32 -3.482 -3.352 10.869 1.00 0.00 O

ATOM 468 ND2 ASN A 32 -4.818 -3.957 9.170 1.00 0.00 N

ATOM 469 HB2 ASN A 32 -3.477 -6.011 8.985 1.00 0.00 H

ATOM 470 HB3 ASN A 32 -3.340 -6.120 10.698 1.00 0.00 H

ATOM 471 HD22 ASN A 32 -5.349 -3.100 9.228 1.00 0.00 H

ATOM 472 HD21 ASN A 32 -5.056 -4.645 8.470 1.00 0.00 H

ATOM 473 H ASN A 32 -0.524 -3.615 9.183 1.00 0.00 H

ATOM 474 HA ASN A 32 -1.059 -5.176 10.649 1.00 0.00 H

ATOM 475 N ASP A 33 0.171 -6.875 8.539 1.00 10.07 N

ATOM 476 CA ASP A 33 0.773 -8.157 8.122 1.00 9.82 C

ATOM 477 C ASP A 33 1.874 -8.655 9.084 1.00 9.51 C

ATOM 478 O ASP A 33 2.304 -9.801 8.945 1.00 10.13 O

ATOM 479 CB ASP A 33 1.278 -8.170 6.654 1.00 9.42 C

ATOM 480 CG ASP A 33 2.319 -7.117 6.257 1.00 9.26 C

ATOM 481 OD1 ASP A 33 2.865 -6.448 7.159 1.00 8.47 O

ATOM 482 OD2 ASP A 33 2.596 -7.050 5.039 1.00 10.67 O1-

ATOM 483 H ASP A 33 0.625 -6.032 8.208 1.00 0.00 H

ATOM 484 HA ASP A 33 -0.000 -8.926 8.155 1.00 0.00 H

ATOM 485 HB3 ASP A 33 0.410 -8.023 6.010 1.00 0.00 H

ATOM 486 HB2 ASP A 33 1.690 -9.152 6.419 1.00 0.00 H

ATOM 487 N GLY A 34 2.316 -7.806 10.025 1.00 9.09 N

ATOM 488 CA GLY A 34 3.362 -8.124 10.993 1.00 8.60 C

ATOM 489 C GLY A 34 4.724 -7.554 10.594 1.00 8.52 C

ATOM 490 O GLY A 34 5.640 -7.564 11.415 1.00 8.11 O

ATOM 491 H GLY A 34 1.936 -6.866 10.060 1.00 0.00 H

ATOM 492 HA3 GLY A 34 3.486 -9.199 11.096 1.00 0.00 H

ATOM 493 HA2 GLY A 34 3.079 -7.740 11.967 1.00 0.00 H

ATOM 494 N TRP A 35 4.866 -7.069 9.355 1.00 8.29 N

ATOM 495 CA TRP A 35 6.089 -6.483 8.834 1.00 7.79 C

ATOM 496 C TRP A 35 5.917 -4.974 8.759 1.00 7.77 C

ATOM 497 O TRP A 35 4.887 -4.494 8.288 1.00 7.30 O

ATOM 498 CB TRP A 35 6.374 -7.064 7.440 1.00 8.47 C

ATOM 499 CG TRP A 35 6.832 -8.488 7.442 1.00 9.28 C

ATOM 500 CD1 TRP A 35 6.049 -9.580 7.599 1.00 10.02 C

ATOM 501 CD2 TRP A 35 8.200 -8.984 7.348 1.00 9.89 C

ATOM 502 NE1 TRP A 35 6.836 -10.714 7.594 1.00 9.21 N

ATOM 503 CE2 TRP A 35 8.173 -10.408 7.432 1.00 9.66 C

ATOM 504 CE3 TRP A 35 9.464 -8.371 7.192 1.00 9.05 C

ATOM 505 CZ2 TRP A 35 9.342 -11.186 7.355 1.00 8.93 C

ATOM 506 CZ3 TRP A 35 10.637 -9.140 7.079 1.00 9.20 C

ATOM 507 CH2 TRP A 35 10.581 -10.545 7.168 1.00 9.34 C

ATOM 508 H TRP A 35 4.068 -7.038 8.730 1.00 0.00 H

ATOM 509 HA TRP A 35 6.946 -6.701 9.476 1.00 0.00 H

ATOM 510 HB3 TRP A 35 7.158 -6.480 6.954 1.00 0.00 H

ATOM 511 HB2 TRP A 35 5.498 -6.974 6.796 1.00 0.00 H

ATOM 512 HD1 TRP A 35 4.975 -9.548 7.716 1.00 0.00 H

ATOM 513 HE1 TRP A 35 6.450 -11.642 7.696 1.00 0.00 H

ATOM 514 HE3 TRP A 35 9.528 -7.295 7.127 1.00 0.00 H

ATOM 515 HZ2 TRP A 35 9.291 -12.262 7.425 1.00 0.00 H

ATOM 516 HZ3 TRP A 35 11.584 -8.646 6.918 1.00 0.00 H

ATOM 517 HH2 TRP A 35 11.488 -11.127 7.089 1.00 0.00 H

ATOM 518 N GLY A 36 6.970 -4.252 9.163 1.00 6.93 N

ATOM 519 CA GLY A 36 7.063 -2.817 8.949 1.00 7.28 C

ATOM 520 C GLY A 36 7.205 -2.534 7.447 1.00 7.40 C

ATOM 521 O GLY A 36 7.653 -3.398 6.696 1.00 7.53 O

ATOM 522 H GLY A 36 7.782 -4.715 9.550 1.00 0.00 H

ATOM 523 HA3 GLY A 36 7.889 -2.400 9.522 1.00 0.00 H

ATOM 524 HA2 GLY A 36 6.149 -2.377 9.322 1.00 0.00 H

ATOM 525 N ASP A 37 6.811 -1.331 7.017 1.00 7.71 N

ATOM 526 CA ASP A 37 6.660 -0.991 5.600 1.00 7.86 C

ATOM 527 C ASP A 37 6.937 0.504 5.384 1.00 8.41 C

ATOM 528 O ASP A 37 7.046 1.255 6.355 1.00 8.14 O

ATOM 529 CB ASP A 37 5.274 -1.428 5.049 1.00 8.41 C

ATOM 530 CG ASP A 37 4.080 -0.985 5.903 1.00 8.41 C

ATOM 531 OD1 ASP A 37 3.927 0.243 6.062 1.00 8.82 O

ATOM 532 OD2 ASP A 37 3.295 -1.855 6.330 1.00 8.78 O1-

ATOM 533 H ASP A 37 6.466 -0.646 7.676 1.00 0.00 H

ATOM 534 HA ASP A 37 7.434 -1.510 5.033 1.00 0.00 H

ATOM 535 HB3 ASP A 37 5.257 -2.518 4.992 1.00 0.00 H

ATOM 536 HB2 ASP A 37 5.110 -1.094 4.025 1.00 0.00 H

ATOM 537 N LEU A 38 7.039 0.913 4.108 1.00 8.78 N

ATOM 538 CA LEU A 38 7.322 2.292 3.705 1.00 9.89 C

ATOM 539 C LEU A 38 6.217 3.286 4.102 1.00 9.57 C

ATOM 540 O LEU A 38 6.558 4.388 4.521 1.00 9.28 O

ATOM 541 CB LEU A 38 7.603 2.373 2.189 1.00 0.00 C

ATOM 542 CG LEU A 38 8.786 1.521 1.675 1.00 0.00 C

ATOM 543 CD1 LEU A 38 8.786 1.490 0.142 1.00 0.00 C

ATOM 544 CD2 LEU A 38 10.163 1.934 2.236 1.00 0.00 C

ATOM 545 HB2 LEU A 38 6.700 2.063 1.659 1.00 0.00 H

ATOM 546 HB3 LEU A 38 7.767 3.412 1.900 1.00 0.00 H

ATOM 547 HG LEU A 38 8.629 0.497 2.004 1.00 0.00 H

ATOM 548 HD11 LEU A 38 9.782 1.316 -0.262 1.00 0.00 H

ATOM 549 HD12 LEU A 38 8.137 0.693 -0.221 1.00 0.00 H

ATOM 550 HD13 LEU A 38 8.412 2.422 -0.274 1.00 0.00 H

ATOM 551 HD21 LEU A 38 10.696 1.066 2.625 1.00 0.00 H

ATOM 552 HD22 LEU A 38 10.798 2.392 1.478 1.00 0.00 H

ATOM 553 HD23 LEU A 38 10.085 2.644 3.053 1.00 0.00 H

ATOM 554 H LEU A 38 6.915 0.245 3.357 1.00 0.00 H

ATOM 555 HA LEU A 38 8.218 2.602 4.241 1.00 0.00 H

ATOM 556 N LYS A 39 4.934 2.884 4.026 1.00 9.73 N

ATOM 557 CA LYS A 39 3.793 3.692 4.484 1.00 10.36 C

ATOM 558 C LYS A 39 3.796 3.920 6.012 1.00 9.56 C

ATOM 559 O LYS A 39 3.367 4.981 6.463 1.00 9.37 O

ATOM 560 CB LYS A 39 2.478 3.077 3.949 1.00 10.75 C

ATOM 561 CG LYS A 39 1.130 3.671 4.439 1.00 14.76 C

ATOM 562 CD LYS A 39 0.594 4.902 3.675 1.00 18.98 C

ATOM 563 CE LYS A 39 1.336 6.232 3.869 1.00 21.33 C

ATOM 564 NZ LYS A 39 1.343 6.669 5.276 1.00 21.02 N1+

ATOM 565 H LYS A 39 4.721 1.952 3.699 1.00 0.00 H

ATOM 566 HA LYS A 39 3.909 4.661 4.006 1.00 0.00 H

ATOM 567 HB3 LYS A 39 2.487 2.025 4.230 1.00 0.00 H

ATOM 568 HB2 LYS A 39 2.493 3.072 2.858 1.00 0.00 H

ATOM 569 HG3 LYS A 39 1.137 3.864 5.510 1.00 0.00 H

ATOM 570 HG2 LYS A 39 0.383 2.885 4.321 1.00 0.00 H

ATOM 571 HD3 LYS A 39 -0.453 5.044 3.944 1.00 0.00 H

ATOM 572 HD2 LYS A 39 0.582 4.668 2.609 1.00 0.00 H

ATOM 573 HE3 LYS A 39 0.848 7.007 3.276 1.00 0.00 H

ATOM 574 HE2 LYS A 39 2.351 6.155 3.487 1.00 0.00 H

ATOM 575 HZ1 LYS A 39 1.838 5.982 5.830 1.00 0.00 H

ATOM 576 HZ2 LYS A 39 1.827 7.553 5.344 1.00 0.00 H

ATOM 577 HZ3 LYS A 39 0.396 6.767 5.612 1.00 0.00 H

ATOM 578 N GLY A 40 4.323 2.960 6.785 1.00 8.91 N

ATOM 579 CA GLY A 40 4.513 3.105 8.222 1.00 8.44 C

ATOM 580 C GLY A 40 5.701 4.030 8.525 1.00 8.14 C

ATOM 581 O GLY A 40 5.609 4.823 9.460 1.00 8.96 O

ATOM 582 H GLY A 40 4.600 2.077 6.372 1.00 0.00 H

ATOM 583 HA3 GLY A 40 4.693 2.126 8.649 1.00 0.00 H

ATOM 584 HA2 GLY A 40 3.606 3.487 8.692 1.00 0.00 H

ATOM 585 N ILE A 41 6.786 3.970 7.729 1.00 7.87 N

ATOM 586 CA ILE A 41 7.942 4.867 7.853 1.00 7.74 C

ATOM 587 C ILE A 41 7.573 6.331 7.532 1.00 8.17 C

ATOM 588 O ILE A 41 7.950 7.204 8.313 1.00 8.73 O

ATOM 589 CB ILE A 41 9.165 4.424 6.988 1.00 7.26 C

ATOM 590 CG1 ILE A 41 9.703 3.041 7.430 1.00 7.73 C

ATOM 591 CG2 ILE A 41 10.334 5.437 6.990 1.00 7.14 C

ATOM 592 CD1 ILE A 41 10.584 2.342 6.382 1.00 8.51 C

ATOM 593 H ILE A 41 6.823 3.286 6.985 1.00 0.00 H

ATOM 594 HA ILE A 41 8.253 4.842 8.898 1.00 0.00 H

ATOM 595 HB ILE A 41 8.819 4.327 5.959 1.00 0.00 H

ATOM 596 HG13 ILE A 41 8.887 2.371 7.692 1.00 0.00 H

ATOM 597 HG12 ILE A 41 10.278 3.154 8.346 1.00 0.00 H

ATOM 598 HG21 ILE A 41 11.200 5.056 6.450 1.00 0.00 H

ATOM 599 HG22 ILE A 41 10.074 6.386 6.525 1.00 0.00 H

ATOM 600 HG23 ILE A 41 10.648 5.651 8.010 1.00 0.00 H

ATOM 601 HD11 ILE A 41 10.543 1.260 6.505 1.00 0.00 H

ATOM 602 HD12 ILE A 41 10.261 2.568 5.368 1.00 0.00 H

ATOM 603 HD13 ILE A 41 11.627 2.645 6.468 1.00 0.00 H

ATOM 604 N THR A 42 6.805 6.564 6.446 1.00 8.28 N

ATOM 605 CA THR A 42 6.290 7.885 6.050 1.00 8.98 C

ATOM 606 C THR A 42 5.405 8.539 7.134 1.00 9.15 C

ATOM 607 O THR A 42 5.533 9.745 7.345 1.00 9.33 O

ATOM 608 CB THR A 42 5.455 7.871 4.739 1.00 0.00 C

ATOM 609 OG1 THR A 42 4.305 7.079 4.845 1.00 0.00 O

ATOM 610 CG2 THR A 42 6.220 7.484 3.471 1.00 0.00 C

ATOM 611 HB THR A 42 5.054 8.867 4.567 1.00 0.00 H

ATOM 612 HG1 THR A 42 4.579 6.161 4.916 1.00 0.00 H

ATOM 613 HG21 THR A 42 5.546 7.274 2.639 1.00 0.00 H

ATOM 614 HG22 THR A 42 6.849 8.315 3.165 1.00 0.00 H

ATOM 615 HG23 THR A 42 6.862 6.620 3.610 1.00 0.00 H

ATOM 616 H THR A 42 6.557 5.788 5.844 1.00 0.00 H

ATOM 617 HA THR A 42 7.154 8.517 5.871 1.00 0.00 H

ATOM 618 N SER A 43 4.566 7.739 7.821 1.00 9.32 N

ATOM 619 CA SER A 43 3.685 8.191 8.903 1.00 9.92 C

ATOM 620 C SER A 43 4.430 8.630 10.182 1.00 10.00 C

ATOM 621 O SER A 43 3.821 9.289 11.023 1.00 10.90 O

ATOM 622 CB SER A 43 2.612 7.110 9.176 1.00 9.91 C

ATOM 623 OG SER A 43 3.059 6.054 10.005 1.00 11.30 O

ATOM 624 H SER A 43 4.525 6.755 7.593 1.00 0.00 H

ATOM 625 HA SER A 43 3.162 9.072 8.526 1.00 0.00 H

ATOM 626 HB3 SER A 43 2.223 6.699 8.244 1.00 0.00 H

ATOM 627 HB2 SER A 43 1.761 7.571 9.680 1.00 0.00 H

ATOM 628 HG SER A 43 3.822 5.633 9.595 1.00 0.00 H

ATOM 629 N LYS A 44 5.717 8.259 10.296 1.00 9.46 N

ATOM 630 CA LYS A 44 6.570 8.494 11.457 1.00 9.11 C

ATOM 631 C LYS A 44 7.791 9.377 11.137 1.00 8.84 C

ATOM 632 O LYS A 44 8.650 9.515 12.007 1.00 8.90 O

ATOM 633 CB LYS A 44 6.978 7.124 12.046 1.00 9.56 C

ATOM 634 CG LYS A 44 5.811 6.280 12.597 1.00 10.23 C

ATOM 635 CD LYS A 44 4.996 6.979 13.696 1.00 11.88 C

ATOM 636 CE LYS A 44 3.974 6.047 14.356 1.00 13.19 C

ATOM 637 NZ LYS A 44 3.208 6.751 15.397 1.00 16.82 N1+

ATOM 638 H LYS A 44 6.141 7.727 9.549 1.00 0.00 H

ATOM 639 HA LYS A 44 6.026 9.055 12.218 1.00 0.00 H

ATOM 640 HB3 LYS A 44 7.699 7.265 12.851 1.00 0.00 H

ATOM 641 HB2 LYS A 44 7.487 6.545 11.277 1.00 0.00 H

ATOM 642 HG3 LYS A 44 6.212 5.341 12.980 1.00 0.00 H

ATOM 643 HG2 LYS A 44 5.144 5.998 11.784 1.00 0.00 H

ATOM 644 HD3 LYS A 44 4.457 7.824 13.268 1.00 0.00 H

ATOM 645 HD2 LYS A 44 5.668 7.395 14.446 1.00 0.00 H

ATOM 646 HE3 LYS A 44 4.479 5.198 14.813 1.00 0.00 H

ATOM 647 HE2 LYS A 44 3.282 5.654 13.610 1.00 0.00 H

ATOM 648 HZ1 LYS A 44 3.841 7.093 16.107 1.00 0.00 H

ATOM 649 HZ2 LYS A 44 2.714 7.531 14.986 1.00 0.00 H

ATOM 650 HZ3 LYS A 44 2.544 6.117 15.819 1.00 0.00 H

ATOM 651 N LEU A 45 7.848 10.007 9.946 1.00 8.25 N

ATOM 652 CA LEU A 45 8.894 10.984 9.610 1.00 8.52 C

ATOM 653 C LEU A 45 8.806 12.274 10.445 1.00 8.37 C

ATOM 654 O LEU A 45 9.851 12.863 10.718 1.00 9.01 O

ATOM 655 CB LEU A 45 8.863 11.364 8.117 1.00 8.47 C

ATOM 656 CG LEU A 45 9.220 10.251 7.119 1.00 9.30 C

ATOM 657 CD1 LEU A 45 9.027 10.762 5.675 1.00 10.45 C

ATOM 658 CD2 LEU A 45 10.633 9.673 7.346 1.00 9.28 C

ATOM 659 H LEU A 45 7.126 9.854 9.256 1.00 0.00 H

ATOM 660 HA LEU A 45 9.857 10.528 9.838 1.00 0.00 H

ATOM 661 HB3 LEU A 45 9.575 12.172 7.957 1.00 0.00 H

ATOM 662 HB2 LEU A 45 7.879 11.766 7.870 1.00 0.00 H

ATOM 663 HG LEU A 45 8.517 9.440 7.275 1.00 0.00 H

ATOM 664 HD11 LEU A 45 9.800 10.420 4.992 1.00 0.00 H

ATOM 665 HD12 LEU A 45 8.081 10.425 5.262 1.00 0.00 H

ATOM 666 HD13 LEU A 45 9.025 11.851 5.624 1.00 0.00 H

ATOM 667 HD21 LEU A 45 11.188 9.557 6.418 1.00 0.00 H

ATOM 668 HD22 LEU A 45 11.239 10.302 7.998 1.00 0.00 H

ATOM 669 HD23 LEU A 45 10.575 8.686 7.806 1.00 0.00 H

ATOM 670 N GLN A 46 7.588 12.676 10.856 1.00 8.65 N

ATOM 671 CA GLN A 46 7.375 13.793 11.780 1.00 9.47 C

ATOM 672 C GLN A 46 7.972 13.526 13.168 1.00 8.31 C

ATOM 673 O GLN A 46 8.653 14.406 13.682 1.00 8.59 O

ATOM 674 CB GLN A 46 5.884 14.167 11.883 1.00 0.00 C

ATOM 675 CG GLN A 46 5.337 14.849 10.618 1.00 0.00 C

ATOM 676 CD GLN A 46 3.916 15.369 10.834 1.00 0.00 C

ATOM 677 OE1 GLN A 46 3.713 16.373 11.512 1.00 0.00 O

ATOM 678 NE2 GLN A 46 2.924 14.691 10.254 1.00 0.00 N

ATOM 679 HB2 GLN A 46 5.292 13.279 12.113 1.00 0.00 H

ATOM 680 HB3 GLN A 46 5.749 14.845 12.729 1.00 0.00 H

ATOM 681 HG2 GLN A 46 5.967 15.699 10.353 1.00 0.00 H

ATOM 682 HG3 GLN A 46 5.368 14.163 9.770 1.00 0.00 H

ATOM 683 HE22 GLN A 46 1.970 15.002 10.373 1.00 0.00 H

ATOM 684 HE21 GLN A 46 3.113 13.862 9.711 1.00 0.00 H

ATOM 685 H GLN A 46 6.770 12.147 10.590 1.00 0.00 H

ATOM 686 HA GLN A 46 7.904 14.656 11.371 1.00 0.00 H

ATOM 687 N TYR A 47 7.779 12.306 13.709 1.00 8.18 N

ATOM 688 CA TYR A 47 8.410 11.845 14.951 1.00 7.50 C

ATOM 689 C TYR A 47 9.947 11.926 14.913 1.00 7.31 C

ATOM 690 O TYR A 47 10.546 12.396 15.880 1.00 7.64 O

ATOM 691 CB TYR A 47 7.920 10.421 15.310 1.00 7.48 C

ATOM 692 CG TYR A 47 8.736 9.724 16.388 1.00 7.66 C

ATOM 693 CD1 TYR A 47 8.514 10.037 17.744 1.00 8.08 C

ATOM 694 CD2 TYR A 47 9.765 8.821 16.037 1.00 7.72 C

ATOM 695 CE1 TYR A 47 9.319 9.455 18.740 1.00 8.36 C

ATOM 696 CE2 TYR A 47 10.564 8.233 17.036 1.00 7.99 C

ATOM 697 CZ TYR A 47 10.338 8.551 18.389 1.00 8.27 C

ATOM 698 OH TYR A 47 11.102 7.990 19.367 1.00 8.09 O

ATOM 699 H TYR A 47 7.220 11.627 13.213 1.00 0.00 H

ATOM 700 HA TYR A 47 8.072 12.517 15.742 1.00 0.00 H

ATOM 701 HB3 TYR A 47 7.944 9.783 14.427 1.00 0.00 H

ATOM 702 HB2 TYR A 47 6.874 10.454 15.616 1.00 0.00 H

ATOM 703 HD1 TYR A 47 7.743 10.740 18.022 1.00 0.00 H

ATOM 704 HD2 TYR A 47 9.954 8.590 14.999 1.00 0.00 H

ATOM 705 HE1 TYR A 47 9.160 9.711 19.776 1.00 0.00 H

ATOM 706 HE2 TYR A 47 11.350 7.545 16.760 1.00 0.00 H

ATOM 707 HH TYR A 47 11.818 7.435 19.028 1.00 0.00 H

ATOM 708 N ILE A 48 10.538 11.476 13.794 1.00 6.95 N

ATOM 709 CA ILE A 48 11.977 11.520 13.553 1.00 7.33 C

ATOM 710 C ILE A 48 12.494 12.972 13.475 1.00 7.45 C

ATOM 711 O ILE A 48 13.513 13.268 14.092 1.00 7.51 O

ATOM 712 CB ILE A 48 12.367 10.723 12.273 1.00 6.88 C

ATOM 713 CG1 ILE A 48 12.047 9.218 12.437 1.00 7.65 C

ATOM 714 CG2 ILE A 48 13.840 10.888 11.854 1.00 7.19 C

ATOM 715 CD1 ILE A 48 12.098 8.428 11.120 1.00 7.94 C

ATOM 716 H ILE A 48 9.971 11.095 13.048 1.00 0.00 H

ATOM 717 HA ILE A 48 12.466 11.044 14.406 1.00 0.00 H

ATOM 718 HB ILE A 48 11.755 11.100 11.452 1.00 0.00 H

ATOM 719 HG13 ILE A 48 11.061 9.078 12.875 1.00 0.00 H

ATOM 720 HG12 ILE A 48 12.742 8.778 13.152 1.00 0.00 H

ATOM 721 HG21 ILE A 48 14.080 10.274 10.990 1.00 0.00 H

ATOM 722 HG22 ILE A 48 14.077 11.913 11.571 1.00 0.00 H

ATOM 723 HG23 ILE A 48 14.508 10.595 12.664 1.00 0.00 H

ATOM 724 HD11 ILE A 48 11.425 7.573 11.151 1.00 0.00 H

ATOM 725 HD12 ILE A 48 11.805 9.040 10.268 1.00 0.00 H

ATOM 726 HD13 ILE A 48 13.102 8.051 10.929 1.00 0.00 H

ATOM 727 N LYS A 49 11.752 13.870 12.804 1.00 7.95 N

ATOM 728 CA LYS A 49 12.077 15.297 12.734 1.00 8.78 C

ATOM 729 C LYS A 49 11.997 16.000 14.108 1.00 8.98 C

ATOM 730 O LYS A 49 12.921 16.740 14.444 1.00 9.38 O

ATOM 731 CB LYS A 49 11.191 15.982 11.668 1.00 8.92 C

ATOM 732 CG LYS A 49 11.607 17.425 11.316 1.00 10.33 C

ATOM 733 CD LYS A 49 12.905 17.488 10.495 1.00 16.03 C

ATOM 734 CE LYS A 49 13.464 18.905 10.327 1.00 17.41 C

ATOM 735 NZ LYS A 49 14.685 18.893 9.503 1.00 18.62 N1+

ATOM 736 H LYS A 49 10.914 13.573 12.322 1.00 0.00 H

ATOM 737 HA LYS A 49 13.116 15.351 12.408 1.00 0.00 H

ATOM 738 HB3 LYS A 49 10.154 15.980 12.006 1.00 0.00 H

ATOM 739 HB2 LYS A 49 11.202 15.392 10.750 1.00 0.00 H

ATOM 740 HG3 LYS A 49 11.707 18.026 12.220 1.00 0.00 H

ATOM 741 HG2 LYS A 49 10.806 17.892 10.742 1.00 0.00 H

ATOM 742 HD3 LYS A 49 12.687 17.079 9.511 1.00 0.00 H

ATOM 743 HD2 LYS A 49 13.674 16.850 10.928 1.00 0.00 H

ATOM 744 HE3 LYS A 49 13.701 19.333 11.301 1.00 0.00 H

ATOM 745 HE2 LYS A 49 12.725 19.556 9.859 1.00 0.00 H

ATOM 746 HZ1 LYS A 49 15.382 18.306 9.941 1.00 0.00 H

ATOM 747 HZ2 LYS A 49 14.469 18.533 8.584 1.00 0.00 H

ATOM 748 HZ3 LYS A 49 15.046 19.832 9.417 1.00 0.00 H

ATOM 749 N ASP A 50 10.936 15.712 14.889 1.00 9.80 N

ATOM 750 CA ASP A 50 10.741 16.157 16.278 1.00 11.70 C

ATOM 751 C ASP A 50 11.791 15.654 17.283 1.00 10.65 C

ATOM 752 O ASP A 50 12.011 16.340 18.282 1.00 11.09 O

ATOM 753 CB ASP A 50 9.319 15.872 16.821 1.00 0.00 C

ATOM 754 CG ASP A 50 8.246 16.819 16.280 1.00 0.00 C

ATOM 755 OD1 ASP A 50 7.539 16.434 15.325 1.00 0.00 O

ATOM 756 OD2 ASP A 50 8.122 17.909 16.879 1.00 0.00 O1-

ATOM 757 HB2 ASP A 50 9.024 14.849 16.586 1.00 0.00 H

ATOM 758 HB3 ASP A 50 9.278 15.914 17.911 1.00 0.00 H

ATOM 759 H ASP A 50 10.205 15.116 14.519 1.00 0.00 H

ATOM 760 HA ASP A 50 10.865 17.242 16.254 1.00 0.00 H

ATOM 761 N LEU A 51 12.442 14.509 17.009 1.00 9.84 N

ATOM 762 CA LEU A 51 13.582 14.015 17.786 1.00 9.21 C

ATOM 763 C LEU A 51 14.815 14.942 17.685 1.00 9.10 C

ATOM 764 O LEU A 51 15.555 15.051 18.662 1.00 8.77 O

ATOM 765 CB LEU A 51 13.887 12.550 17.386 1.00 9.16 C

ATOM 766 CG LEU A 51 15.130 11.907 18.040 1.00 9.91 C

ATOM 767 CD1 LEU A 51 15.056 11.905 19.576 1.00 11.66 C

ATOM 768 CD2 LEU A 51 15.365 10.497 17.477 1.00 10.08 C

ATOM 769 H LEU A 51 12.191 13.976 16.187 1.00 0.00 H

ATOM 770 HA LEU A 51 13.273 14.004 18.831 1.00 0.00 H

ATOM 771 HB3 LEU A 51 14.027 12.497 16.312 1.00 0.00 H

ATOM 772 HB2 LEU A 51 13.012 11.935 17.600 1.00 0.00 H

ATOM 773 HG LEU A 51 16.009 12.481 17.749 1.00 0.00 H

ATOM 774 HD11 LEU A 51 15.426 10.972 19.987 1.00 0.00 H

ATOM 775 HD12 LEU A 51 15.667 12.701 20.002 1.00 0.00 H

ATOM 776 HD13 LEU A 51 14.038 12.042 19.940 1.00 0.00 H

ATOM 777 HD21 LEU A 51 16.349 10.119 17.755 1.00 0.00 H

ATOM 778 HD22 LEU A 51 14.619 9.793 17.840 1.00 0.00 H

ATOM 779 HD23 LEU A 51 15.307 10.490 16.389 1.00 0.00 H

ATOM 780 N GLY A 52 14.981 15.617 16.535 1.00 8.61 N

ATOM 781 CA GLY A 52 16.092 16.528 16.256 1.00 9.10 C

ATOM 782 C GLY A 52 17.000 15.978 15.147 1.00 9.21 C

ATOM 783 O GLY A 52 18.114 16.473 14.980 1.00 9.48 O

ATOM 784 H GLY A 52 14.288 15.514 15.806 1.00 0.00 H

ATOM 785 HA3 GLY A 52 16.691 16.736 17.144 1.00 0.00 H

ATOM 786 HA2 GLY A 52 15.680 17.481 15.923 1.00 0.00 H

ATOM 787 N VAL A 53 16.545 14.957 14.404 1.00 8.26 N

ATOM 788 CA VAL A 53 17.273 14.320 13.311 1.00 8.22 C

ATOM 789 C VAL A 53 17.196 15.152 12.015 1.00 8.23 C

ATOM 790 O VAL A 53 16.154 15.731 11.705 1.00 9.55 O

ATOM 791 CB VAL A 53 16.692 12.904 13.084 1.00 0.00 C

ATOM 792 CG1 VAL A 53 17.035 12.236 11.747 1.00 0.00 C

ATOM 793 CG2 VAL A 53 17.065 11.994 14.271 1.00 0.00 C

ATOM 794 HB VAL A 53 15.616 13.010 13.074 1.00 0.00 H

ATOM 795 HG11 VAL A 53 16.552 11.272 11.642 1.00 0.00 H

ATOM 796 HG12 VAL A 53 16.704 12.812 10.883 1.00 0.00 H

ATOM 797 HG13 VAL A 53 18.107 12.078 11.683 1.00 0.00 H

ATOM 798 HG21 VAL A 53 16.357 11.173 14.364 1.00 0.00 H

ATOM 799 HG22 VAL A 53 18.070 11.584 14.164 1.00 0.00 H

ATOM 800 HG23 VAL A 53 17.030 12.520 15.225 1.00 0.00 H

ATOM 801 H VAL A 53 15.622 14.589 14.584 1.00 0.00 H

ATOM 802 HA VAL A 53 18.321 14.221 13.603 1.00 0.00 H

ATOM 803 N ASP A 54 18.323 15.156 11.289 1.00 7.72 N

ATOM 804 CA ASP A 54 18.564 15.913 10.059 1.00 7.78 C

ATOM 805 C ASP A 54 18.610 15.020 8.806 1.00 7.31 C

ATOM 806 O ASP A 54 18.658 15.562 7.702 1.00 7.58 O

ATOM 807 CB ASP A 54 19.889 16.706 10.142 1.00 7.64 C

ATOM 808 CG ASP A 54 20.060 17.524 11.424 1.00 8.66 C

ATOM 809 OD1 ASP A 54 20.988 17.199 12.198 1.00 9.87 O

ATOM 810 OD2 ASP A 54 19.311 18.512 11.572 1.00 12.63 O1-

ATOM 811 H ASP A 54 19.112 14.617 11.621 1.00 0.00 H

ATOM 812 HA ASP A 54 17.753 16.628 9.906 1.00 0.00 H

ATOM 813 HB3 ASP A 54 20.014 17.375 9.290 1.00 0.00 H

ATOM 814 HB2 ASP A 54 20.721 16.006 10.074 1.00 0.00 H

ATOM 815 N ALA A 55 18.638 13.689 8.975 1.00 6.76 N

ATOM 816 CA ALA A 55 18.832 12.732 7.889 1.00 6.10 C

ATOM 817 C ALA A 55 18.376 11.334 8.318 1.00 6.40 C

ATOM 818 O ALA A 55 18.428 11.029 9.504 1.00 5.91 O

ATOM 819 CB ALA A 55 20.321 12.718 7.504 1.00 6.65 C

ATOM 820 H ALA A 55 18.599 13.298 9.906 1.00 0.00 H

ATOM 821 HA ALA A 55 18.228 13.047 7.041 1.00 0.00 H

ATOM 822 HB1 ALA A 55 20.529 11.998 6.711 1.00 0.00 H

ATOM 823 HB2 ALA A 55 20.660 13.696 7.163 1.00 0.00 H

ATOM 824 HB3 ALA A 55 20.937 12.468 8.366 1.00 0.00 H

ATOM 825 N ILE A 56 17.974 10.481 7.366 1.00 5.93 N

ATOM 826 CA ILE A 56 17.642 9.087 7.664 1.00 6.14 C

ATOM 827 C ILE A 56 18.449 8.124 6.789 1.00 5.88 C

ATOM 828 O ILE A 56 18.693 8.397 5.615 1.00 5.91 O

ATOM 829 CB ILE A 56 16.124 8.772 7.539 1.00 6.35 C

ATOM 830 CG1 ILE A 56 15.527 8.923 6.119 1.00 7.02 C

ATOM 831 CG2 ILE A 56 15.326 9.578 8.580 1.00 6.73 C

ATOM 832 CD1 ILE A 56 14.073 8.443 6.021 1.00 6.56 C

ATOM 833 H ILE A 56 17.931 10.769 6.397 1.00 0.00 H

ATOM 834 HA ILE A 56 17.928 8.855 8.689 1.00 0.00 H

ATOM 835 HB ILE A 56 16.004 7.722 7.815 1.00 0.00 H

ATOM 836 HG13 ILE A 56 16.105 8.366 5.384 1.00 0.00 H

ATOM 837 HG12 ILE A 56 15.586 9.968 5.819 1.00 0.00 H

ATOM 838 HG21 ILE A 56 14.303 9.218 8.684 1.00 0.00 H

ATOM 839 HG22 ILE A 56 15.789 9.502 9.562 1.00 0.00 H

ATOM 840 HG23 ILE A 56 15.281 10.636 8.321 1.00 0.00 H

ATOM 841 HD11 ILE A 56 13.718 8.463 4.993 1.00 0.00 H

ATOM 842 HD12 ILE A 56 13.965 7.422 6.387 1.00 0.00 H

ATOM 843 HD13 ILE A 56 13.409 9.083 6.597 1.00 0.00 H

ATOM 844 N TRP A 57 18.823 7.001 7.410 1.00 5.61 N

ATOM 845 CA TRP A 57 19.386 5.819 6.779 1.00 5.55 C

ATOM 846 C TRP A 57 18.347 4.710 6.941 1.00 6.04 C

ATOM 847 O TRP A 57 18.040 4.342 8.071 1.00 5.90 O

ATOM 848 CB TRP A 57 20.731 5.475 7.460 1.00 5.99 C

ATOM 849 CG TRP A 57 21.317 4.098 7.276 1.00 5.15 C

ATOM 850 CD1 TRP A 57 21.101 3.231 6.256 1.00 4.80 C

ATOM 851 CD2 TRP A 57 22.254 3.426 8.166 1.00 5.01 C

ATOM 852 NE1 TRP A 57 21.698 2.022 6.548 1.00 5.59 N

ATOM 853 CE2 TRP A 57 22.411 2.080 7.724 1.00 5.24 C

ATOM 854 CE3 TRP A 57 22.955 3.807 9.332 1.00 5.23 C

ATOM 855 CZ2 TRP A 57 23.172 1.142 8.441 1.00 5.72 C

ATOM 856 CZ3 TRP A 57 23.781 2.895 10.020 1.00 5.20 C

ATOM 857 CH2 TRP A 57 23.881 1.560 9.584 1.00 4.97 C

ATOM 858 H TRP A 57 18.573 6.883 8.384 1.00 0.00 H

ATOM 859 HA TRP A 57 19.561 5.974 5.718 1.00 0.00 H

ATOM 860 HB3 TRP A 57 20.600 5.579 8.539 1.00 0.00 H

ATOM 861 HB2 TRP A 57 21.483 6.215 7.198 1.00 0.00 H

ATOM 862 HD1 TRP A 57 20.488 3.434 5.391 1.00 0.00 H

ATOM 863 HE1 TRP A 57 21.624 1.201 5.956 1.00 0.00 H

ATOM 864 HE3 TRP A 57 22.851 4.816 9.697 1.00 0.00 H

ATOM 865 HZ2 TRP A 57 23.239 0.120 8.096 1.00 0.00 H

ATOM 866 HZ3 TRP A 57 24.310 3.213 10.907 1.00 0.00 H

ATOM 867 HH2 TRP A 57 24.488 0.855 10.132 1.00 0.00 H

ATOM 868 N VAL A 58 17.846 4.177 5.821 1.00 5.54 N

ATOM 869 CA VAL A 58 16.959 3.020 5.845 1.00 6.11 C

ATOM 870 C VAL A 58 17.770 1.797 5.377 1.00 5.53 C

ATOM 871 O VAL A 58 18.360 1.831 4.295 1.00 5.89 O

ATOM 872 CB VAL A 58 15.709 3.206 4.937 1.00 0.00 C

ATOM 873 CG1 VAL A 58 14.654 2.103 5.166 1.00 0.00 C

ATOM 874 CG2 VAL A 58 15.038 4.577 5.151 1.00 0.00 C

ATOM 875 HB VAL A 58 16.015 3.159 3.890 1.00 0.00 H

ATOM 876 HG11 VAL A 58 13.785 2.251 4.523 1.00 0.00 H

ATOM 877 HG12 VAL A 58 15.038 1.110 4.954 1.00 0.00 H

ATOM 878 HG13 VAL A 58 14.301 2.102 6.198 1.00 0.00 H

ATOM 879 HG21 VAL A 58 14.124 4.668 4.564 1.00 0.00 H

ATOM 880 HG22 VAL A 58 14.777 4.728 6.198 1.00 0.00 H

ATOM 881 HG23 VAL A 58 15.690 5.398 4.851 1.00 0.00 H

ATOM 882 H VAL A 58 18.132 4.517 4.913 1.00 0.00 H

ATOM 883 HA VAL A 58 16.592 2.826 6.853 1.00 0.00 H

ATOM 884 N CYS A 59 17.767 0.741 6.209 1.00 5.26 N

ATOM 885 CA CYS A 59 18.288 -0.605 5.927 1.00 4.91 C

ATOM 886 C CYS A 59 17.612 -1.219 4.675 1.00 5.03 C

ATOM 887 O CYS A 59 16.520 -0.777 4.321 1.00 4.91 O

ATOM 888 CB CYS A 59 18.088 -1.493 7.171 1.00 0.00 C

ATOM 889 SG CYS A 59 19.472 -1.304 8.313 1.00 0.00 S

ATOM 890 HB2 CYS A 59 17.949 -2.550 6.950 1.00 0.00 H

ATOM 891 HB3 CYS A 59 17.201 -1.181 7.703 1.00 0.00 H

ATOM 892 HG CYS A 59 20.375 -1.947 7.561 1.00 0.00 H

ATOM 893 H CYS A 59 17.287 0.835 7.095 1.00 0.00 H

ATOM 894 HA CYS A 59 19.353 -0.504 5.712 1.00 0.00 H

ATOM 895 N PRO A 60 18.284 -2.175 3.989 1.00 5.34 N

ATOM 896 CA PRO A 60 17.978 -2.536 2.591 1.00 5.53 C

ATOM 897 C PRO A 60 16.540 -3.034 2.365 1.00 5.55 C

ATOM 898 O PRO A 60 16.204 -4.163 2.724 1.00 5.88 O

ATOM 899 CB PRO A 60 19.048 -3.580 2.228 1.00 6.12 C

ATOM 900 CG PRO A 60 19.491 -4.155 3.556 1.00 5.66 C

ATOM 901 CD PRO A 60 19.406 -2.963 4.489 1.00 5.68 C

ATOM 902 HA PRO A 60 18.138 -1.655 1.964 1.00 0.00 H

ATOM 903 HB3 PRO A 60 19.895 -3.087 1.750 1.00 0.00 H

ATOM 904 HB2 PRO A 60 18.698 -4.351 1.543 1.00 0.00 H

ATOM 905 HG3 PRO A 60 20.475 -4.618 3.517 1.00 0.00 H

ATOM 906 HG2 PRO A 60 18.776 -4.907 3.889 1.00 0.00 H

ATOM 907 HD2 PRO A 60 20.317 -2.377 4.443 1.00 0.00 H

ATOM 908 HD3 PRO A 60 19.281 -3.310 5.510 1.00 0.00 H

ATOM 909 N PHE A 61 15.727 -2.138 1.785 1.00 5.48 N

ATOM 910 CA PHE A 61 14.312 -2.330 1.473 1.00 5.49 C

ATOM 911 C PHE A 61 14.057 -2.998 0.107 1.00 5.66 C

ATOM 912 O PHE A 61 12.903 -3.112 -0.305 1.00 6.53 O

ATOM 913 CB PHE A 61 13.569 -0.982 1.628 1.00 5.60 C

ATOM 914 CG PHE A 61 14.167 0.170 0.838 1.00 5.88 C

ATOM 915 CD1 PHE A 61 13.990 0.247 -0.559 1.00 6.83 C

ATOM 916 CD2 PHE A 61 15.053 1.070 1.467 1.00 6.89 C

ATOM 917 CE1 PHE A 61 14.660 1.215 -1.291 1.00 7.05 C

ATOM 918 CE2 PHE A 61 15.690 2.051 0.721 1.00 6.91 C

ATOM 919 CZ PHE A 61 15.498 2.120 -0.653 1.00 6.83 C

ATOM 920 H PHE A 61 16.095 -1.227 1.551 1.00 0.00 H

ATOM 921 HA PHE A 61 13.906 -3.002 2.221 1.00 0.00 H

ATOM 922 HB3 PHE A 61 12.529 -1.092 1.317 1.00 0.00 H

ATOM 923 HB2 PHE A 61 13.525 -0.709 2.683 1.00 0.00 H

ATOM 924 HD1 PHE A 61 13.351 -0.459 -1.067 1.00 0.00 H

ATOM 925 HD2 PHE A 61 15.242 0.999 2.528 1.00 0.00 H

ATOM 926 HE1 PHE A 61 14.526 1.265 -2.360 1.00 0.00 H

ATOM 927 HE2 PHE A 61 16.353 2.750 1.210 1.00 0.00 H

ATOM 928 HZ PHE A 61 16.009 2.877 -1.230 1.00 0.00 H

ATOM 929 N TYR A 62 15.129 -3.403 -0.586 1.00 5.44 N

ATOM 930 CA TYR A 62 15.091 -4.012 -1.913 1.00 5.84 C

ATOM 931 C TYR A 62 14.567 -5.450 -1.860 1.00 6.21 C

ATOM 932 O TYR A 62 14.650 -6.087 -0.807 1.00 6.69 O

ATOM 933 CB TYR A 62 16.500 -4.016 -2.523 1.00 5.22 C

ATOM 934 CG TYR A 62 17.314 -2.774 -2.256 1.00 5.70 C

ATOM 935 CD1 TYR A 62 17.141 -1.609 -3.028 1.00 7.25 C

ATOM 936 CD2 TYR A 62 18.244 -2.791 -1.202 1.00 6.00 C

ATOM 937 CE1 TYR A 62 17.948 -0.484 -2.775 1.00 7.11 C

ATOM 938 CE2 TYR A 62 19.052 -1.674 -0.959 1.00 5.57 C

ATOM 939 CZ TYR A 62 18.927 -0.529 -1.767 1.00 5.51 C

ATOM 940 OH TYR A 62 19.769 0.526 -1.591 1.00 5.95 O

ATOM 941 H TYR A 62 16.048 -3.273 -0.188 1.00 0.00 H

ATOM 942 HA TYR A 62 14.439 -3.413 -2.546 1.00 0.00 H

ATOM 943 HB3 TYR A 62 16.438 -4.169 -3.596 1.00 0.00 H

ATOM 944 HB2 TYR A 62 17.069 -4.863 -2.136 1.00 0.00 H

ATOM 945 HD1 TYR A 62 16.404 -1.581 -3.817 1.00 0.00 H

ATOM 946 HD2 TYR A 62 18.356 -3.674 -0.593 1.00 0.00 H

ATOM 947 HE1 TYR A 62 17.833 0.410 -3.364 1.00 0.00 H

ATOM 948 HE2 TYR A 62 19.769 -1.724 -0.159 1.00 0.00 H

ATOM 949 HH TYR A 62 20.480 0.313 -0.974 1.00 0.00 H

ATOM 950 N ASP A 63 14.078 -5.934 -3.015 1.00 6.64 N

ATOM 951 CA ASP A 63 13.580 -7.291 -3.244 1.00 7.03 C

ATOM 952 C ASP A 63 14.630 -8.324 -2.799 1.00 7.04 C

ATOM 953 O ASP A 63 15.774 -8.256 -3.247 1.00 7.07 O

ATOM 954 CB ASP A 63 13.164 -7.474 -4.723 1.00 7.22 C

ATOM 955 CG ASP A 63 12.304 -8.708 -5.023 1.00 9.09 C

ATOM 956 OD1 ASP A 63 12.072 -8.939 -6.230 1.00 12.33 O

ATOM 957 OD2 ASP A 63 11.853 -9.374 -4.065 1.00 9.91 O1-

ATOM 958 H ASP A 63 14.062 -5.326 -3.824 1.00 0.00 H

ATOM 959 HA ASP A 63 12.703 -7.406 -2.611 1.00 0.00 H

ATOM 960 HB3 ASP A 63 14.067 -7.541 -5.327 1.00 0.00 H

ATOM 961 HB2 ASP A 63 12.606 -6.595 -5.047 1.00 0.00 H

ATOM 962 N SER A 64 14.231 -9.195 -1.864 1.00 6.81 N

ATOM 963 CA SER A 64 15.130 -10.021 -1.067 1.00 6.88 C

ATOM 964 C SER A 64 14.321 -11.188 -0.467 1.00 7.17 C

ATOM 965 O SER A 64 13.276 -10.927 0.131 1.00 6.86 O

ATOM 966 CB SER A 64 15.746 -9.130 0.040 1.00 6.56 C

ATOM 967 OG SER A 64 16.413 -9.888 1.032 1.00 6.37 O

ATOM 968 H SER A 64 13.262 -9.205 -1.578 1.00 0.00 H

ATOM 969 HA SER A 64 15.941 -10.366 -1.703 1.00 0.00 H

ATOM 970 HB3 SER A 64 14.980 -8.528 0.525 1.00 0.00 H

ATOM 971 HB2 SER A 64 16.460 -8.427 -0.390 1.00 0.00 H

ATOM 972 HG SER A 64 17.218 -10.251 0.647 1.00 0.00 H

ATOM 973 N PRO A 65 14.831 -12.439 -0.577 1.00 7.33 N

ATOM 974 CA PRO A 65 14.308 -13.601 0.175 1.00 7.57 C

ATOM 975 C PRO A 65 14.317 -13.518 1.720 1.00 7.46 C

ATOM 976 O PRO A 65 13.716 -14.390 2.346 1.00 7.74 O

ATOM 977 CB PRO A 65 15.182 -14.771 -0.311 1.00 7.38 C

ATOM 978 CG PRO A 65 15.679 -14.351 -1.677 1.00 7.84 C

ATOM 979 CD PRO A 65 15.884 -12.856 -1.506 1.00 7.44 C

ATOM 980 HA PRO A 65 13.281 -13.761 -0.157 1.00 0.00 H

ATOM 981 HB3 PRO A 65 14.649 -15.722 -0.333 1.00 0.00 H

ATOM 982 HB2 PRO A 65 16.049 -14.889 0.335 1.00 0.00 H

ATOM 983 HG3 PRO A 65 14.900 -14.530 -2.419 1.00 0.00 H

ATOM 984 HG2 PRO A 65 16.577 -14.882 -1.993 1.00 0.00 H

ATOM 985 HD2 PRO A 65 16.859 -12.642 -1.069 1.00 0.00 H

ATOM 986 HD3 PRO A 65 15.816 -12.367 -2.476 1.00 0.00 H

ATOM 987 N GLN A 66 14.978 -12.498 2.302 1.00 6.56 N

ATOM 988 CA GLN A 66 15.084 -12.207 3.740 1.00 6.73 C

ATOM 989 C GLN A 66 15.910 -13.244 4.534 1.00 6.73 C

ATOM 990 O GLN A 66 15.741 -13.338 5.750 1.00 7.00 O

ATOM 991 CB GLN A 66 13.690 -11.969 4.392 1.00 6.73 C

ATOM 992 CG GLN A 66 12.786 -10.918 3.719 1.00 6.92 C

ATOM 993 CD GLN A 66 13.373 -9.505 3.683 1.00 7.35 C

ATOM 994 OE1 GLN A 66 13.840 -8.989 4.696 1.00 9.01 O

ATOM 995 NE2 GLN A 66 13.311 -8.856 2.522 1.00 7.58 N

ATOM 996 H GLN A 66 15.464 -11.840 1.707 1.00 0.00 H

ATOM 997 HA GLN A 66 15.652 -11.278 3.806 1.00 0.00 H

ATOM 998 HB3 GLN A 66 13.823 -11.675 5.434 1.00 0.00 H

ATOM 999 HB2 GLN A 66 13.140 -12.910 4.433 1.00 0.00 H

ATOM 1000 HG3 GLN A 66 11.846 -10.866 4.269 1.00 0.00 H

ATOM 1001 HG2 GLN A 66 12.525 -11.239 2.711 1.00 0.00 H

ATOM 1002 HE22 GLN A 66 13.667 -7.915 2.447 1.00 0.00 H

ATOM 1003 HE21 GLN A 66 12.918 -9.308 1.707 1.00 0.00 H

ATOM 1004 N GLN A 67 16.819 -13.976 3.862 1.00 6.24 N

ATOM 1005 CA GLN A 67 17.745 -14.936 4.480 1.00 6.63 C

ATOM 1006 C GLN A 67 18.780 -14.269 5.401 1.00 6.53 C

ATOM 1007 O GLN A 67 19.234 -14.916 6.341 1.00 7.37 O

ATOM 1008 CB GLN A 67 18.460 -15.768 3.394 1.00 0.00 C

ATOM 1009 CG GLN A 67 17.510 -16.573 2.489 1.00 0.00 C

ATOM 1010 CD GLN A 67 16.817 -17.745 3.189 1.00 0.00 C

ATOM 1011 OE1 GLN A 67 17.428 -18.463 3.980 1.00 0.00 O

ATOM 1012 NE2 GLN A 67 15.540 -17.965 2.872 1.00 0.00 N

ATOM 1013 HB2 GLN A 67 19.059 -15.106 2.768 1.00 0.00 H

ATOM 1014 HB3 GLN A 67 19.182 -16.444 3.856 1.00 0.00 H

ATOM 1015 HG2 GLN A 67 16.754 -15.912 2.074 1.00 0.00 H

ATOM 1016 HG3 GLN A 67 18.071 -16.955 1.639 1.00 0.00 H

ATOM 1017 HE22 GLN A 67 15.036 -18.729 3.297 1.00 0.00 H

ATOM 1018 HE21 GLN A 67 15.061 -17.353 2.226 1.00 0.00 H

ATOM 1019 H GLN A 67 16.954 -13.807 2.872 1.00 0.00 H

ATOM 1020 HA GLN A 67 17.154 -15.611 5.102 1.00 0.00 H

ATOM 1021 N ASP A 68 19.088 -12.988 5.139 1.00 5.88 N

ATOM 1022 CA ASP A 68 19.846 -12.096 6.013 1.00 6.22 C

ATOM 1023 C ASP A 68 19.094 -10.751 6.132 1.00 5.94 C

ATOM 1024 O ASP A 68 19.721 -9.699 6.246 1.00 6.34 O

ATOM 1025 CB ASP A 68 21.306 -11.952 5.528 1.00 5.97 C

ATOM 1026 CG ASP A 68 22.264 -11.231 6.488 1.00 6.81 C

ATOM 1027 OD1 ASP A 68 22.004 -11.251 7.711 1.00 6.06 O

ATOM 1028 OD2 ASP A 68 23.285 -10.715 5.985 1.00 5.52 O1-

ATOM 1029 H ASP A 68 18.701 -12.551 4.310 1.00 0.00 H

ATOM 1030 HA ASP A 68 19.850 -12.515 7.021 1.00 0.00 H

ATOM 1031 HB3 ASP A 68 21.291 -11.411 4.581 1.00 0.00 H

ATOM 1032 HB2 ASP A 68 21.712 -12.945 5.337 1.00 0.00 H

ATOM 1033 N MET A 69 17.749 -10.809 6.115 1.00 5.90 N

ATOM 1034 CA MET A 69 16.829 -9.698 6.373 1.00 6.07 C

ATOM 1035 C MET A 69 17.096 -8.435 5.525 1.00 6.01 C

ATOM 1036 O MET A 69 17.243 -7.340 6.072 1.00 6.00 O

ATOM 1037 CB MET A 69 16.790 -9.407 7.890 1.00 6.56 C

ATOM 1038 CG MET A 69 16.136 -10.518 8.729 1.00 7.28 C

ATOM 1039 SD MET A 69 14.373 -10.790 8.388 1.00 7.76 S

ATOM 1040 CE MET A 69 13.737 -9.119 8.694 1.00 9.86 C

ATOM 1041 H MET A 69 17.311 -11.714 6.012 1.00 0.00 H

ATOM 1042 HA MET A 69 15.844 -10.045 6.065 1.00 0.00 H

ATOM 1043 HB3 MET A 69 16.243 -8.484 8.070 1.00 0.00 H

ATOM 1044 HB2 MET A 69 17.798 -9.222 8.264 1.00 0.00 H

ATOM 1045 HG3 MET A 69 16.235 -10.274 9.787 1.00 0.00 H

ATOM 1046 HG2 MET A 69 16.661 -11.462 8.587 1.00 0.00 H

ATOM 1047 HE1 MET A 69 12.675 -9.156 8.930 1.00 0.00 H

ATOM 1048 HE2 MET A 69 14.251 -8.656 9.536 1.00 0.00 H

ATOM 1049 HE3 MET A 69 13.877 -8.490 7.815 1.00 0.00 H

ATOM 1050 N GLY A 70 17.158 -8.621 4.198 1.00 5.83 N

ATOM 1051 CA GLY A 70 17.305 -7.536 3.230 1.00 5.14 C

ATOM 1052 C GLY A 70 18.732 -7.449 2.672 1.00 5.09 C

ATOM 1053 O GLY A 70 18.892 -6.951 1.559 1.00 5.07 O

ATOM 1054 H GLY A 70 17.065 -9.555 3.822 1.00 0.00 H

ATOM 1055 HA3 GLY A 70 17.041 -6.572 3.666 1.00 0.00 H

ATOM 1056 HA2 GLY A 70 16.602 -7.692 2.415 1.00 0.00 H

ATOM 1057 N TYR A 71 19.758 -7.929 3.400 1.00 4.99 N

ATOM 1058 CA TYR A 71 21.166 -7.912 2.968 1.00 5.21 C

ATOM 1059 C TYR A 71 21.514 -8.976 1.907 1.00 5.53 C

ATOM 1060 O TYR A 71 22.640 -8.985 1.412 1.00 5.40 O

ATOM 1061 CB TYR A 71 22.089 -7.964 4.203 1.00 5.47 C

ATOM 1062 CG TYR A 71 22.069 -6.666 4.987 1.00 4.61 C

ATOM 1063 CD1 TYR A 71 21.144 -6.469 6.034 1.00 4.75 C

ATOM 1064 CD2 TYR A 71 22.929 -5.616 4.608 1.00 5.12 C

ATOM 1065 CE1 TYR A 71 21.055 -5.216 6.665 1.00 5.66 C

ATOM 1066 CE2 TYR A 71 22.831 -4.360 5.231 1.00 5.43 C

ATOM 1067 CZ TYR A 71 21.887 -4.157 6.252 1.00 5.02 C

ATOM 1068 OH TYR A 71 21.754 -2.928 6.821 1.00 5.14 O

ATOM 1069 H TYR A 71 19.577 -8.335 4.308 1.00 0.00 H

ATOM 1070 HA TYR A 71 21.342 -6.951 2.478 1.00 0.00 H

ATOM 1071 HB3 TYR A 71 23.122 -8.146 3.903 1.00 0.00 H

ATOM 1072 HB2 TYR A 71 21.814 -8.795 4.851 1.00 0.00 H

ATOM 1073 HD1 TYR A 71 20.474 -7.261 6.330 1.00 0.00 H

ATOM 1074 HD2 TYR A 71 23.643 -5.761 3.812 1.00 0.00 H

ATOM 1075 HE1 TYR A 71 20.322 -5.057 7.442 1.00 0.00 H

ATOM 1076 HE2 TYR A 71 23.471 -3.552 4.909 1.00 0.00 H

ATOM 1077 HH TYR A 71 22.174 -2.216 6.319 1.00 0.00 H

ATOM 1078 N ASP A 72 20.535 -9.815 1.542 1.00 5.48 N

ATOM 1079 CA ASP A 72 20.559 -10.753 0.425 1.00 5.68 C

ATOM 1080 C ASP A 72 19.659 -10.168 -0.683 1.00 5.48 C

ATOM 1081 O ASP A 72 18.496 -10.551 -0.790 1.00 5.68 O

ATOM 1082 CB ASP A 72 20.106 -12.185 0.835 1.00 5.49 C

ATOM 1083 CG ASP A 72 18.795 -12.314 1.632 1.00 6.26 C

ATOM 1084 OD1 ASP A 72 18.584 -11.540 2.592 1.00 6.24 O

ATOM 1085 OD2 ASP A 72 18.036 -13.247 1.293 1.00 6.08 O1-

ATOM 1086 H ASP A 72 19.653 -9.773 2.037 1.00 0.00 H

ATOM 1087 HA ASP A 72 21.569 -10.831 0.019 1.00 0.00 H

ATOM 1088 HB3 ASP A 72 20.875 -12.656 1.441 1.00 0.00 H

ATOM 1089 HB2 ASP A 72 20.027 -12.797 -0.065 1.00 0.00 H

ATOM 1090 N ILE A 73 20.185 -9.226 -1.481 1.00 5.76 N

ATOM 1091 CA ILE A 73 19.396 -8.500 -2.484 1.00 6.04 C

ATOM 1092 C ILE A 73 19.292 -9.284 -3.809 1.00 6.38 C

ATOM 1093 O ILE A 73 20.319 -9.571 -4.423 1.00 6.20 O

ATOM 1094 CB ILE A 73 19.992 -7.089 -2.761 1.00 5.77 C

ATOM 1095 CG1 ILE A 73 19.961 -6.230 -1.476 1.00 6.30 C

ATOM 1096 CG2 ILE A 73 19.294 -6.344 -3.923 1.00 7.47 C

ATOM 1097 CD1 ILE A 73 20.773 -4.936 -1.577 1.00 6.41 C

ATOM 1098 H ILE A 73 21.143 -8.930 -1.355 1.00 0.00 H

ATOM 1099 HA ILE A 73 18.387 -8.348 -2.094 1.00 0.00 H

ATOM 1100 HB ILE A 73 21.041 -7.216 -3.039 1.00 0.00 H

ATOM 1101 HG13 ILE A 73 20.367 -6.793 -0.636 1.00 0.00 H

ATOM 1102 HG12 ILE A 73 18.930 -6.000 -1.207 1.00 0.00 H

ATOM 1103 HG21 ILE A 73 19.653 -5.324 -4.031 1.00 0.00 H

ATOM 1104 HG22 ILE A 73 19.474 -6.822 -4.885 1.00 0.00 H

ATOM 1105 HG23 ILE A 73 18.216 -6.295 -3.766 1.00 0.00 H

ATOM 1106 HD11 ILE A 73 20.891 -4.474 -0.597 1.00 0.00 H

ATOM 1107 HD12 ILE A 73 21.762 -5.139 -1.981 1.00 0.00 H

ATOM 1108 HD13 ILE A 73 20.302 -4.203 -2.229 1.00 0.00 H

ATOM 1109 N SER A 74 18.054 -9.585 -4.233 1.00 6.57 N

ATOM 1110 CA SER A 74 17.726 -10.230 -5.510 1.00 6.76 C

ATOM 1111 C SER A 74 17.452 -9.232 -6.651 1.00 6.62 C

ATOM 1112 O SER A 74 17.484 -9.639 -7.811 1.00 6.98 O

ATOM 1113 CB SER A 74 16.555 -11.214 -5.307 1.00 0.00 C

ATOM 1114 OG SER A 74 15.299 -10.575 -5.181 1.00 0.00 O

ATOM 1115 HB2 SER A 74 16.486 -11.878 -6.168 1.00 0.00 H

ATOM 1116 HB3 SER A 74 16.738 -11.853 -4.444 1.00 0.00 H

ATOM 1117 HG SER A 74 15.293 -10.053 -4.375 1.00 0.00 H

ATOM 1118 H SER A 74 17.260 -9.288 -3.679 1.00 0.00 H

ATOM 1119 HA SER A 74 18.584 -10.825 -5.822 1.00 0.00 H

ATOM 1120 N ASN A 75 17.200 -7.954 -6.331 1.00 7.10 N

ATOM 1121 CA ASN A 75 16.995 -6.905 -7.324 1.00 6.79 C

ATOM 1122 C ASN A 75 17.197 -5.557 -6.628 1.00 6.57 C

ATOM 1123 O ASN A 75 16.354 -5.167 -5.824 1.00 6.55 O

ATOM 1124 CB ASN A 75 15.590 -7.020 -7.975 1.00 6.84 C

ATOM 1125 CG ASN A 75 15.406 -6.202 -9.260 1.00 7.61 C

ATOM 1126 OD1 ASN A 75 16.183 -5.301 -9.569 1.00 8.11 O

ATOM 1127 ND2 ASN A 75 14.347 -6.507 -10.012 1.00 7.95 N

ATOM 1128 H ASN A 75 17.160 -7.673 -5.362 1.00 0.00 H

ATOM 1129 HA ASN A 75 17.759 -6.975 -8.103 1.00 0.00 H

ATOM 1130 HB3 ASN A 75 14.808 -6.753 -7.268 1.00 0.00 H

ATOM 1131 HB2 ASN A 75 15.398 -8.058 -8.248 1.00 0.00 H

ATOM 1132 HD22 ASN A 75 14.175 -6.001 -10.868 1.00 0.00 H

ATOM 1133 HD21 ASN A 75 13.721 -7.251 -9.738 1.00 0.00 H

ATOM 1134 N TYR A 76 18.299 -4.870 -6.969 1.00 6.08 N

ATOM 1135 CA TYR A 76 18.633 -3.531 -6.475 1.00 6.44 C

ATOM 1136 C TYR A 76 17.662 -2.438 -6.949 1.00 6.56 C

ATOM 1137 O TYR A 76 17.528 -1.431 -6.261 1.00 7.33 O

ATOM 1138 CB TYR A 76 20.055 -3.157 -6.930 1.00 6.78 C

ATOM 1139 CG TYR A 76 21.161 -4.025 -6.367 1.00 7.20 C

ATOM 1140 CD1 TYR A 76 21.701 -3.727 -5.102 1.00 7.41 C

ATOM 1141 CD2 TYR A 76 21.656 -5.124 -7.100 1.00 6.45 C

ATOM 1142 CE1 TYR A 76 22.725 -4.528 -4.568 1.00 6.41 C

ATOM 1143 CE2 TYR A 76 22.668 -5.938 -6.555 1.00 7.26 C

ATOM 1144 CZ TYR A 76 23.201 -5.643 -5.285 1.00 7.37 C

ATOM 1145 OH TYR A 76 24.177 -6.435 -4.756 1.00 7.26 O

ATOM 1146 H TYR A 76 18.942 -5.264 -7.639 1.00 0.00 H

ATOM 1147 HA TYR A 76 18.605 -3.551 -5.384 1.00 0.00 H

ATOM 1148 HB3 TYR A 76 20.260 -2.131 -6.629 1.00 0.00 H

ATOM 1149 HB2 TYR A 76 20.114 -3.162 -8.018 1.00 0.00 H

ATOM 1150 HD1 TYR A 76 21.334 -2.881 -4.538 1.00 0.00 H

ATOM 1151 HD2 TYR A 76 21.256 -5.345 -8.078 1.00 0.00 H

ATOM 1152 HE1 TYR A 76 23.134 -4.273 -3.604 1.00 0.00 H

ATOM 1153 HE2 TYR A 76 23.034 -6.789 -7.111 1.00 0.00 H

ATOM 1154 HH TYR A 76 24.432 -6.177 -3.863 1.00 0.00 H

ATOM 1155 N GLU A 77 17.023 -2.649 -8.109 1.00 6.81 N

ATOM 1156 CA GLU A 77 16.162 -1.679 -8.784 1.00 7.33 C

ATOM 1157 C GLU A 77 14.669 -1.881 -8.475 1.00 7.45 C

ATOM 1158 O GLU A 77 13.847 -1.195 -9.080 1.00 8.23 O

ATOM 1159 CB GLU A 77 16.428 -1.756 -10.300 1.00 7.34 C

ATOM 1160 CG GLU A 77 17.898 -1.486 -10.671 1.00 7.53 C

ATOM 1161 CD GLU A 77 18.156 -1.580 -12.173 1.00 8.91 C

ATOM 1162 OE1 GLU A 77 17.338 -1.030 -12.943 1.00 11.87 O

ATOM 1163 OE2 GLU A 77 19.179 -2.206 -12.527 1.00 8.50 O1-

ATOM 1164 H GLU A 77 17.171 -3.518 -8.605 1.00 0.00 H

ATOM 1165 HA GLU A 77 16.418 -0.672 -8.450 1.00 0.00 H

ATOM 1166 HB3 GLU A 77 15.795 -1.034 -10.817 1.00 0.00 H

ATOM 1167 HB2 GLU A 77 16.133 -2.737 -10.677 1.00 0.00 H

ATOM 1168 HG3 GLU A 77 18.549 -2.195 -10.163 1.00 0.00 H

ATOM 1169 HG2 GLU A 77 18.199 -0.498 -10.329 1.00 0.00 H

ATOM 1170 N LYS A 78 14.337 -2.798 -7.551 1.00 7.78 N

ATOM 1171 CA LYS A 78 12.969 -3.078 -7.136 1.00 7.88 C

ATOM 1172 C LYS A 78 12.911 -3.147 -5.608 1.00 7.54 C

ATOM 1173 O LYS A 78 13.710 -3.859 -5.003 1.00 7.64 O

ATOM 1174 CB LYS A 78 12.486 -4.378 -7.811 1.00 8.49 C

ATOM 1175 CG LYS A 78 11.026 -4.746 -7.499 1.00 10.48 C

ATOM 1176 CD LYS A 78 10.543 -5.975 -8.276 1.00 14.37 C

ATOM 1177 CE LYS A 78 9.082 -6.319 -7.960 1.00 16.20 C

ATOM 1178 NZ LYS A 78 8.660 -7.543 -8.660 1.00 18.59 N1+

ATOM 1179 H LYS A 78 15.061 -3.323 -7.081 1.00 0.00 H

ATOM 1180 HA LYS A 78 12.314 -2.272 -7.460 1.00 0.00 H

ATOM 1181 HB3 LYS A 78 13.129 -5.206 -7.516 1.00 0.00 H

ATOM 1182 HB2 LYS A 78 12.596 -4.272 -8.891 1.00 0.00 H

ATOM 1183 HG3 LYS A 78 10.379 -3.894 -7.710 1.00 0.00 H

ATOM 1184 HG2 LYS A 78 10.928 -4.960 -6.437 1.00 0.00 H

ATOM 1185 HD3 LYS A 78 11.182 -6.826 -8.033 1.00 0.00 H

ATOM 1186 HD2 LYS A 78 10.658 -5.800 -9.347 1.00 0.00 H

ATOM 1187 HE3 LYS A 78 8.426 -5.497 -8.250 1.00 0.00 H

ATOM 1188 HE2 LYS A 78 8.955 -6.472 -6.887 1.00 0.00 H

ATOM 1189 HZ1 LYS A 78 9.246 -8.312 -8.366 1.00 0.00 H

ATOM 1190 HZ2 LYS A 78 7.697 -7.749 -8.432 1.00 0.00 H

ATOM 1191 HZ3 LYS A 78 8.751 -7.413 -9.657 1.00 0.00 H

ATOM 1192 N VAL A 79 11.932 -2.438 -5.029 1.00 6.93 N

ATOM 1193 CA VAL A 79 11.558 -2.518 -3.617 1.00 6.71 C

ATOM 1194 C VAL A 79 10.837 -3.855 -3.361 1.00 6.85 C

ATOM 1195 O VAL A 79 10.025 -4.265 -4.193 1.00 7.36 O

ATOM 1196 CB VAL A 79 10.583 -1.365 -3.249 1.00 7.13 C

ATOM 1197 CG1 VAL A 79 10.069 -1.396 -1.795 1.00 7.23 C

ATOM 1198 CG2 VAL A 79 11.209 0.011 -3.521 1.00 7.23 C

ATOM 1199 H VAL A 79 11.322 -1.876 -5.609 1.00 0.00 H

ATOM 1200 HA VAL A 79 12.452 -2.447 -2.998 1.00 0.00 H

ATOM 1201 HB VAL A 79 9.708 -1.447 -3.897 1.00 0.00 H

ATOM 1202 HG11 VAL A 79 9.380 -0.572 -1.629 1.00 0.00 H

ATOM 1203 HG12 VAL A 79 9.516 -2.302 -1.559 1.00 0.00 H

ATOM 1204 HG13 VAL A 79 10.884 -1.304 -1.077 1.00 0.00 H

ATOM 1205 HG21 VAL A 79 10.479 0.794 -3.335 1.00 0.00 H

ATOM 1206 HG22 VAL A 79 12.071 0.197 -2.882 1.00 0.00 H

ATOM 1207 HG23 VAL A 79 11.535 0.121 -4.555 1.00 0.00 H

ATOM 1208 N TRP A 80 11.124 -4.498 -2.215 1.00 6.01 N

ATOM 1209 CA TRP A 80 10.467 -5.736 -1.789 1.00 7.05 C

ATOM 1210 C TRP A 80 8.953 -5.491 -1.607 1.00 7.07 C

ATOM 1211 O TRP A 80 8.622 -4.618 -0.806 1.00 6.76 O

ATOM 1212 CB TRP A 80 11.098 -6.209 -0.465 1.00 6.92 C

ATOM 1213 CG TRP A 80 10.685 -7.575 -0.000 1.00 6.88 C

ATOM 1214 CD1 TRP A 80 10.667 -8.694 -0.761 1.00 7.10 C

ATOM 1215 CD2 TRP A 80 10.290 -8.006 1.335 1.00 8.10 C

ATOM 1216 NE1 TRP A 80 10.256 -9.769 -0.003 1.00 9.01 N

ATOM 1217 CE2 TRP A 80 10.007 -9.404 1.302 1.00 8.33 C

ATOM 1218 CE3 TRP A 80 10.137 -7.355 2.577 1.00 8.80 C

ATOM 1219 CZ2 TRP A 80 9.580 -10.113 2.439 1.00 7.67 C

ATOM 1220 CZ3 TRP A 80 9.718 -8.051 3.726 1.00 7.73 C

ATOM 1221 CH2 TRP A 80 9.432 -9.428 3.658 1.00 7.56 C

ATOM 1222 H TRP A 80 11.785 -4.093 -1.564 1.00 0.00 H

ATOM 1223 HA TRP A 80 10.689 -6.478 -2.550 1.00 0.00 H

ATOM 1224 HB3 TRP A 80 10.896 -5.491 0.330 1.00 0.00 H

ATOM 1225 HB2 TRP A 80 12.178 -6.237 -0.559 1.00 0.00 H

ATOM 1226 HD1 TRP A 80 10.926 -8.728 -1.807 1.00 0.00 H

ATOM 1227 HE1 TRP A 80 10.174 -10.702 -0.383 1.00 0.00 H

ATOM 1228 HE3 TRP A 80 10.322 -6.298 2.633 1.00 0.00 H

ATOM 1229 HZ2 TRP A 80 9.372 -11.171 2.379 1.00 0.00 H

ATOM 1230 HZ3 TRP A 80 9.599 -7.517 4.655 1.00 0.00 H

ATOM 1231 HH2 TRP A 80 9.103 -9.957 4.539 1.00 0.00 H

ATOM 1232 N PRO A 81 8.076 -6.195 -2.368 1.00 8.09 N

ATOM 1233 CA PRO A 81 6.617 -5.943 -2.383 1.00 8.40 C

ATOM 1234 C PRO A 81 5.883 -5.791 -1.035 1.00 8.83 C

ATOM 1235 O PRO A 81 4.930 -5.016 -0.975 1.00 9.31 O

ATOM 1236 CB PRO A 81 6.054 -7.112 -3.201 1.00 8.71 C

ATOM 1237 CG PRO A 81 7.162 -7.437 -4.184 1.00 8.46 C

ATOM 1238 CD PRO A 81 8.427 -7.215 -3.365 1.00 8.13 C

ATOM 1239 HA PRO A 81 6.474 -5.022 -2.950 1.00 0.00 H

ATOM 1240 HB3 PRO A 81 5.117 -6.865 -3.701 1.00 0.00 H

ATOM 1241 HB2 PRO A 81 5.871 -7.977 -2.560 1.00 0.00 H

ATOM 1242 HG3 PRO A 81 7.133 -6.720 -5.006 1.00 0.00 H

ATOM 1243 HG2 PRO A 81 7.091 -8.439 -4.607 1.00 0.00 H

ATOM 1244 HD2 PRO A 81 8.713 -8.131 -2.847 1.00 0.00 H

ATOM 1245 HD3 PRO A 81 9.239 -6.923 -4.031 1.00 0.00 H

ATOM 1246 N THR A 82 6.350 -6.490 0.015 1.00 8.62 N

ATOM 1247 CA THR A 82 5.836 -6.393 1.387 1.00 9.14 C

ATOM 1248 C THR A 82 6.091 -5.017 2.047 1.00 8.76 C

ATOM 1249 O THR A 82 5.233 -4.555 2.798 1.00 9.73 O

ATOM 1250 CB THR A 82 6.465 -7.494 2.278 1.00 9.31 C

ATOM 1251 OG1 THR A 82 6.135 -8.766 1.755 1.00 10.58 O

ATOM 1252 CG2 THR A 82 6.104 -7.478 3.776 1.00 9.98 C

ATOM 1253 H THR A 82 7.124 -7.126 -0.115 1.00 0.00 H

ATOM 1254 HA THR A 82 4.756 -6.549 1.352 1.00 0.00 H

ATOM 1255 HB THR A 82 7.543 -7.407 2.193 1.00 0.00 H

ATOM 1256 HG1 THR A 82 6.383 -9.433 2.400 1.00 0.00 H

ATOM 1257 HG21 THR A 82 6.515 -8.347 4.290 1.00 0.00 H

ATOM 1258 HG22 THR A 82 6.501 -6.599 4.284 1.00 0.00 H

ATOM 1259 HG23 THR A 82 5.024 -7.489 3.922 1.00 0.00 H

ATOM 1260 N TYR A 83 7.224 -4.366 1.723 1.00 8.68 N

ATOM 1261 CA TYR A 83 7.518 -2.984 2.121 1.00 8.14 C

ATOM 1262 C TYR A 83 6.763 -1.948 1.270 1.00 8.44 C

ATOM 1263 O TYR A 83 6.498 -0.858 1.774 1.00 8.43 O

ATOM 1264 CB TYR A 83 9.043 -2.723 2.081 1.00 8.16 C

ATOM 1265 CG TYR A 83 9.919 -3.537 3.026 1.00 7.83 C

ATOM 1266 CD1 TYR A 83 9.424 -4.037 4.247 1.00 7.34 C

ATOM 1267 CD2 TYR A 83 11.264 -3.781 2.691 1.00 8.19 C

ATOM 1268 CE1 TYR A 83 10.260 -4.747 5.128 1.00 7.20 C

ATOM 1269 CE2 TYR A 83 12.102 -4.514 3.556 1.00 8.06 C

ATOM 1270 CZ TYR A 83 11.601 -4.992 4.780 1.00 7.31 C

ATOM 1271 OH TYR A 83 12.406 -5.714 5.610 1.00 7.99 O

ATOM 1272 H TYR A 83 7.876 -4.788 1.076 1.00 0.00 H

ATOM 1273 HA TYR A 83 7.161 -2.834 3.139 1.00 0.00 H

ATOM 1274 HB3 TYR A 83 9.236 -1.679 2.325 1.00 0.00 H

ATOM 1275 HB2 TYR A 83 9.409 -2.861 1.063 1.00 0.00 H

ATOM 1276 HD1 TYR A 83 8.391 -3.895 4.509 1.00 0.00 H

ATOM 1277 HD2 TYR A 83 11.644 -3.413 1.756 1.00 0.00 H

ATOM 1278 HE1 TYR A 83 9.860 -5.125 6.056 1.00 0.00 H

ATOM 1279 HE2 TYR A 83 13.124 -4.724 3.279 1.00 0.00 H

ATOM 1280 HH TYR A 83 13.276 -5.886 5.243 1.00 0.00 H

ATOM 1281 N GLY A 84 6.402 -2.307 0.030 1.00 8.67 N

ATOM 1282 CA GLY A 84 5.635 -1.459 -0.873 1.00 8.75 C

ATOM 1283 C GLY A 84 6.231 -1.531 -2.279 1.00 8.61 C

ATOM 1284 O GLY A 84 7.045 -2.399 -2.599 1.00 8.75 O

ATOM 1285 H GLY A 84 6.661 -3.220 -0.319 1.00 0.00 H

ATOM 1286 HA3 GLY A 84 5.623 -0.417 -0.545 1.00 0.00 H

ATOM 1287 HA2 GLY A 84 4.602 -1.806 -0.899 1.00 0.00 H

ATOM 1288 N THR A 85 5.780 -0.599 -3.126 1.00 8.21 N

ATOM 1289 CA THR A 85 6.218 -0.419 -4.507 1.00 8.44 C

ATOM 1290 C THR A 85 7.394 0.577 -4.585 1.00 8.24 C

ATOM 1291 O THR A 85 7.725 1.234 -3.595 1.00 8.06 O

ATOM 1292 CB THR A 85 5.046 0.119 -5.382 1.00 8.23 C

ATOM 1293 OG1 THR A 85 4.729 1.478 -5.132 1.00 9.09 O

ATOM 1294 CG2 THR A 85 3.763 -0.721 -5.272 1.00 9.00 C

ATOM 1295 H THR A 85 5.107 0.080 -2.784 1.00 0.00 H

ATOM 1296 HA THR A 85 6.551 -1.374 -4.918 1.00 0.00 H

ATOM 1297 HB THR A 85 5.359 0.074 -6.426 1.00 0.00 H

ATOM 1298 HG1 THR A 85 4.223 1.532 -4.315 1.00 0.00 H

ATOM 1299 HG21 THR A 85 3.004 -0.368 -5.970 1.00 0.00 H

ATOM 1300 HG22 THR A 85 3.961 -1.768 -5.502 1.00 0.00 H

ATOM 1301 HG23 THR A 85 3.332 -0.675 -4.271 1.00 0.00 H

ATOM 1302 N ASN A 86 7.989 0.689 -5.786 1.00 8.12 N

ATOM 1303 CA ASN A 86 8.995 1.704 -6.119 1.00 8.21 C

ATOM 1304 C ASN A 86 8.454 3.133 -5.973 1.00 8.46 C

ATOM 1305 O ASN A 86 9.193 3.973 -5.470 1.00 8.67 O

ATOM 1306 CB ASN A 86 9.540 1.468 -7.543 1.00 8.07 C

ATOM 1307 CG ASN A 86 10.547 0.319 -7.632 1.00 9.13 C

ATOM 1308 OD1 ASN A 86 10.379 -0.722 -7.001 1.00 9.42 O

ATOM 1309 ND2 ASN A 86 11.596 0.509 -8.433 1.00 8.69 N

ATOM 1310 H ASN A 86 7.689 0.098 -6.547 1.00 0.00 H

ATOM 1311 HA ASN A 86 9.804 1.554 -5.405 1.00 0.00 H

ATOM 1312 HB3 ASN A 86 10.056 2.367 -7.879 1.00 0.00 H

ATOM 1313 HB2 ASN A 86 8.727 1.306 -8.252 1.00 0.00 H

ATOM 1314 HD22 ASN A 86 12.298 -0.214 -8.548 1.00 0.00 H

ATOM 1315 HD21 ASN A 86 11.700 1.374 -8.942 1.00 0.00 H

ATOM 1316 N GLU A 87 7.184 3.371 -6.350 1.00 8.92 N

ATOM 1317 CA GLU A 87 6.521 4.665 -6.185 1.00 9.90 C

ATOM 1318 C GLU A 87 6.304 5.058 -4.711 1.00 9.34 C

ATOM 1319 O GLU A 87 6.454 6.236 -4.396 1.00 9.97 O

ATOM 1320 CB GLU A 87 5.222 4.709 -7.023 1.00 9.73 C

ATOM 1321 CG GLU A 87 4.438 6.044 -6.980 1.00 14.65 C

ATOM 1322 CD GLU A 87 5.248 7.268 -7.427 1.00 19.32 C

ATOM 1323 OE1 GLU A 87 6.109 7.103 -8.320 1.00 21.23 O

ATOM 1324 OE2 GLU A 87 4.990 8.357 -6.872 1.00 22.48 O1-

ATOM 1325 H GLU A 87 6.620 2.625 -6.728 1.00 0.00 H

ATOM 1326 HA GLU A 87 7.205 5.406 -6.600 1.00 0.00 H

ATOM 1327 HB3 GLU A 87 4.558 3.910 -6.692 1.00 0.00 H

ATOM 1328 HB2 GLU A 87 5.460 4.475 -8.061 1.00 0.00 H

ATOM 1329 HG3 GLU A 87 4.050 6.223 -5.976 1.00 0.00 H

ATOM 1330 HG2 GLU A 87 3.564 5.970 -7.627 1.00 0.00 H

ATOM 1331 N ASP A 88 6.035 4.077 -3.828 1.00 9.05 N

ATOM 1332 CA ASP A 88 5.994 4.268 -2.369 1.00 8.77 C

ATOM 1333 C ASP A 88 7.349 4.712 -1.794 1.00 8.70 C

ATOM 1334 O ASP A 88 7.365 5.546 -0.890 1.00 9.38 O

ATOM 1335 CB ASP A 88 5.480 3.038 -1.578 1.00 8.75 C

ATOM 1336 CG ASP A 88 4.133 2.483 -2.045 1.00 9.55 C

ATOM 1337 OD1 ASP A 88 3.287 3.287 -2.488 1.00 12.50 O

ATOM 1338 OD2 ASP A 88 3.944 1.257 -1.892 1.00 10.29 O1-

ATOM 1339 H ASP A 88 5.912 3.131 -4.162 1.00 0.00 H

ATOM 1340 HA ASP A 88 5.295 5.086 -2.185 1.00 0.00 H

ATOM 1341 HB3 ASP A 88 5.397 3.279 -0.517 1.00 0.00 H

ATOM 1342 HB2 ASP A 88 6.216 2.237 -1.662 1.00 0.00 H

ATOM 1343 N CYS A 89 8.453 4.171 -2.341 1.00 8.12 N

ATOM 1344 CA CYS A 89 9.819 4.550 -1.982 1.00 7.69 C

ATOM 1345 C CYS A 89 10.175 5.960 -2.483 1.00 7.91 C

ATOM 1346 O CYS A 89 10.806 6.708 -1.740 1.00 7.52 O

ATOM 1347 CB CYS A 89 10.848 3.498 -2.438 1.00 7.84 C

ATOM 1348 SG CYS A 89 12.532 3.893 -1.886 1.00 9.12 S

ATOM 1349 H CYS A 89 8.354 3.486 -3.079 1.00 0.00 H

ATOM 1350 HA CYS A 89 9.864 4.576 -0.891 1.00 0.00 H

ATOM 1351 HB3 CYS A 89 10.848 3.382 -3.521 1.00 0.00 H

ATOM 1352 HB2 CYS A 89 10.587 2.534 -2.010 1.00 0.00 H

ATOM 1353 HG CYS A 89 12.654 5.015 -2.601 1.00 0.00 H

ATOM 1354 N PHE A 90 9.744 6.309 -3.708 1.00 7.62 N

ATOM 1355 CA PHE A 90 9.966 7.628 -4.304 1.00 8.02 C

ATOM 1356 C PHE A 90 9.180 8.719 -3.556 1.00 7.90 C

ATOM 1357 O PHE A 90 9.785 9.732 -3.214 1.00 8.47 O

ATOM 1358 CB PHE A 90 9.649 7.608 -5.816 1.00 8.38 C

ATOM 1359 CG PHE A 90 10.358 6.541 -6.645 1.00 8.53 C

ATOM 1360 CD1 PHE A 90 11.637 6.043 -6.296 1.00 10.31 C

ATOM 1361 CD2 PHE A 90 9.776 6.115 -7.858 1.00 8.95 C

ATOM 1362 CE1 PHE A 90 12.240 5.066 -7.076 1.00 10.21 C

ATOM 1363 CE2 PHE A 90 10.405 5.151 -8.634 1.00 8.86 C

ATOM 1364 CZ PHE A 90 11.623 4.616 -8.235 1.00 10.99 C

ATOM 1365 H PHE A 90 9.245 5.638 -4.277 1.00 0.00 H

ATOM 1366 HA PHE A 90 11.023 7.872 -4.188 1.00 0.00 H

ATOM 1367 HB3 PHE A 90 9.885 8.581 -6.249 1.00 0.00 H

ATOM 1368 HB2 PHE A 90 8.575 7.474 -5.953 1.00 0.00 H

ATOM 1369 HD1 PHE A 90 12.145 6.384 -5.407 1.00 0.00 H

ATOM 1370 HD2 PHE A 90 8.826 6.519 -8.176 1.00 0.00 H

ATOM 1371 HE1 PHE A 90 13.203 4.667 -6.792 1.00 0.00 H

ATOM 1372 HE2 PHE A 90 9.943 4.814 -9.550 1.00 0.00 H

ATOM 1373 HZ PHE A 90 12.107 3.867 -8.844 1.00 0.00 H

ATOM 1374 N GLU A 91 7.901 8.454 -3.221 1.00 7.86 N

ATOM 1375 CA GLU A 91 7.081 9.291 -2.335 1.00 7.72 C

ATOM 1376 C GLU A 91 7.674 9.468 -0.929 1.00 7.84 C

ATOM 1377 O GLU A 91 7.564 10.565 -0.386 1.00 8.24 O

ATOM 1378 CB GLU A 91 5.640 8.752 -2.210 1.00 0.00 C

ATOM 1379 CG GLU A 91 4.746 9.083 -3.415 1.00 0.00 C

ATOM 1380 CD GLU A 91 3.296 8.657 -3.180 1.00 0.00 C

ATOM 1381 OE1 GLU A 91 2.660 9.262 -2.287 1.00 0.00 O

ATOM 1382 OE2 GLU A 91 2.845 7.734 -3.892 1.00 0.00 O1-

ATOM 1383 HB2 GLU A 91 5.665 7.671 -2.067 1.00 0.00 H

ATOM 1384 HB3 GLU A 91 5.170 9.154 -1.310 1.00 0.00 H

ATOM 1385 HG2 GLU A 91 4.759 10.154 -3.616 1.00 0.00 H

ATOM 1386 HG3 GLU A 91 5.133 8.593 -4.307 1.00 0.00 H

ATOM 1387 H GLU A 91 7.463 7.603 -3.553 1.00 0.00 H

ATOM 1388 HA GLU A 91 7.038 10.287 -2.781 1.00 0.00 H

ATOM 1389 N LEU A 92 8.296 8.414 -0.373 1.00 7.52 N

ATOM 1390 CA LEU A 92 8.981 8.460 0.919 1.00 7.62 C

ATOM 1391 C LEU A 92 10.245 9.334 0.887 1.00 7.44 C

ATOM 1392 O LEU A 92 10.436 10.114 1.818 1.00 7.35 O

ATOM 1393 CB LEU A 92 9.230 7.023 1.435 1.00 7.94 C

ATOM 1394 CG LEU A 92 10.130 6.860 2.684 1.00 8.81 C

ATOM 1395 CD1 LEU A 92 9.639 7.628 3.925 1.00 11.31 C

ATOM 1396 CD2 LEU A 92 10.339 5.378 3.013 1.00 8.87 C

ATOM 1397 H LEU A 92 8.324 7.531 -0.867 1.00 0.00 H

ATOM 1398 HA LEU A 92 8.293 8.947 1.609 1.00 0.00 H

ATOM 1399 HB3 LEU A 92 9.684 6.444 0.632 1.00 0.00 H

ATOM 1400 HB2 LEU A 92 8.266 6.549 1.619 1.00 0.00 H

ATOM 1401 HG LEU A 92 11.109 7.262 2.434 1.00 0.00 H

ATOM 1402 HD11 LEU A 92 10.486 7.989 4.510 1.00 0.00 H

ATOM 1403 HD12 LEU A 92 9.039 8.499 3.675 1.00 0.00 H

ATOM 1404 HD13 LEU A 92 9.035 6.996 4.575 1.00 0.00 H

ATOM 1405 HD21 LEU A 92 10.609 5.225 4.058 1.00 0.00 H

ATOM 1406 HD22 LEU A 92 9.431 4.809 2.823 1.00 0.00 H

ATOM 1407 HD23 LEU A 92 11.138 4.953 2.405 1.00 0.00 H

ATOM 1408 N ILE A 93 11.048 9.227 -0.186 1.00 7.00 N

ATOM 1409 CA ILE A 93 12.223 10.069 -0.436 1.00 6.82 C

ATOM 1410 C ILE A 93 11.840 11.558 -0.597 1.00 6.91 C

ATOM 1411 O ILE A 93 12.470 12.402 0.038 1.00 6.61 O

ATOM 1412 CB ILE A 93 13.044 9.574 -1.670 1.00 6.68 C

ATOM 1413 CG1 ILE A 93 13.720 8.214 -1.373 1.00 7.00 C

ATOM 1414 CG2 ILE A 93 14.108 10.566 -2.193 1.00 7.31 C

ATOM 1415 CD1 ILE A 93 14.147 7.437 -2.627 1.00 7.70 C

ATOM 1416 H ILE A 93 10.826 8.551 -0.906 1.00 0.00 H

ATOM 1417 HA ILE A 93 12.865 9.994 0.444 1.00 0.00 H

ATOM 1418 HB ILE A 93 12.336 9.409 -2.483 1.00 0.00 H

ATOM 1419 HG13 ILE A 93 13.063 7.576 -0.783 1.00 0.00 H

ATOM 1420 HG12 ILE A 93 14.594 8.380 -0.744 1.00 0.00 H

ATOM 1421 HG21 ILE A 93 14.679 10.143 -3.018 1.00 0.00 H

ATOM 1422 HG22 ILE A 93 13.666 11.485 -2.576 1.00 0.00 H

ATOM 1423 HG23 ILE A 93 14.816 10.827 -1.406 1.00 0.00 H

ATOM 1424 HD11 ILE A 93 14.324 6.388 -2.388 1.00 0.00 H

ATOM 1425 HD12 ILE A 93 13.383 7.473 -3.403 1.00 0.00 H

ATOM 1426 HD13 ILE A 93 15.072 7.833 -3.047 1.00 0.00 H

ATOM 1427 N ASP A 94 10.779 11.838 -1.377 1.00 7.68 N

ATOM 1428 CA ASP A 94 10.161 13.163 -1.519 1.00 8.73 C

ATOM 1429 C ASP A 94 9.620 13.742 -0.205 1.00 8.00 C

ATOM 1430 O ASP A 94 9.889 14.908 0.069 1.00 8.47 O

ATOM 1431 CB ASP A 94 9.066 13.217 -2.608 1.00 0.00 C

ATOM 1432 CG ASP A 94 9.589 12.999 -4.028 1.00 0.00 C

ATOM 1433 OD1 ASP A 94 9.020 12.136 -4.727 1.00 0.00 O

ATOM 1434 OD2 ASP A 94 10.483 13.779 -4.423 1.00 0.00 O1-

ATOM 1435 HB2 ASP A 94 8.327 12.442 -2.397 1.00 0.00 H

ATOM 1436 HB3 ASP A 94 8.517 14.157 -2.587 1.00 0.00 H

ATOM 1437 H ASP A 94 10.326 11.087 -1.883 1.00 0.00 H

ATOM 1438 HA ASP A 94 10.966 13.832 -1.831 1.00 0.00 H

ATOM 1439 N LYS A 95 8.897 12.935 0.589 1.00 8.06 N

ATOM 1440 CA LYS A 95 8.329 13.347 1.876 1.00 7.85 C

ATOM 1441 C LYS A 95 9.406 13.652 2.932 1.00 7.75 C

ATOM 1442 O LYS A 95 9.242 14.610 3.685 1.00 7.60 O

ATOM 1443 CB LYS A 95 7.321 12.289 2.364 1.00 8.06 C

ATOM 1444 CG LYS A 95 6.520 12.713 3.608 1.00 10.53 C

ATOM 1445 CD LYS A 95 5.514 11.640 4.035 1.00 12.94 C

ATOM 1446 CE LYS A 95 4.680 12.022 5.266 1.00 16.17 C

ATOM 1447 NZ LYS A 95 3.785 13.158 4.990 1.00 17.61 N1+

ATOM 1448 H LYS A 95 8.692 11.990 0.289 1.00 0.00 H

ATOM 1449 HA LYS A 95 7.775 14.272 1.699 1.00 0.00 H

ATOM 1450 HB3 LYS A 95 7.836 11.345 2.554 1.00 0.00 H

ATOM 1451 HB2 LYS A 95 6.608 12.090 1.563 1.00 0.00 H

ATOM 1452 HG3 LYS A 95 6.004 13.650 3.398 1.00 0.00 H

ATOM 1453 HG2 LYS A 95 7.191 12.913 4.443 1.00 0.00 H

ATOM 1454 HD3 LYS A 95 6.072 10.733 4.255 1.00 0.00 H

ATOM 1455 HD2 LYS A 95 4.854 11.393 3.202 1.00 0.00 H

ATOM 1456 HE3 LYS A 95 5.330 12.269 6.106 1.00 0.00 H

ATOM 1457 HE2 LYS A 95 4.065 11.176 5.571 1.00 0.00 H

ATOM 1458 HZ1 LYS A 95 3.244 13.373 5.815 1.00 0.00 H

ATOM 1459 HZ2 LYS A 95 4.334 13.965 4.730 1.00 0.00 H

ATOM 1460 HZ3 LYS A 95 3.160 12.922 4.232 1.00 0.00 H

ATOM 1461 N THR A 96 10.500 12.869 2.931 1.00 6.77 N

ATOM 1462 CA THR A 96 11.693 13.094 3.750 1.00 7.09 C

ATOM 1463 C THR A 96 12.367 14.441 3.409 1.00 7.08 C

ATOM 1464 O THR A 96 12.656 15.207 4.327 1.00 7.02 O

ATOM 1465 CB THR A 96 12.732 11.948 3.574 1.00 7.39 C

ATOM 1466 OG1 THR A 96 12.157 10.718 3.964 1.00 7.40 O

ATOM 1467 CG2 THR A 96 14.035 12.086 4.375 1.00 8.46 C

ATOM 1468 H THR A 96 10.549 12.096 2.280 1.00 0.00 H

ATOM 1469 HA THR A 96 11.379 13.124 4.796 1.00 0.00 H

ATOM 1470 HB THR A 96 12.993 11.852 2.521 1.00 0.00 H

ATOM 1471 HG1 THR A 96 11.473 10.485 3.327 1.00 0.00 H

ATOM 1472 HG21 THR A 96 14.694 11.240 4.187 1.00 0.00 H

ATOM 1473 HG22 THR A 96 14.595 12.972 4.093 1.00 0.00 H

ATOM 1474 HG23 THR A 96 13.845 12.121 5.447 1.00 0.00 H

ATOM 1475 N HIS A 97 12.534 14.733 2.104 1.00 6.88 N

ATOM 1476 CA HIS A 97 13.091 15.992 1.592 1.00 7.06 C

ATOM 1477 C HIS A 97 12.225 17.227 1.871 1.00 7.57 C

ATOM 1478 O HIS A 97 12.782 18.279 2.182 1.00 8.19 O

ATOM 1479 CB HIS A 97 13.383 15.883 0.084 1.00 7.06 C

ATOM 1480 CG HIS A 97 14.498 14.932 -0.259 1.00 7.27 C

ATOM 1481 ND1 HIS A 97 14.490 14.195 -1.434 1.00 8.01 N1+

ATOM 1482 CD2 HIS A 97 15.769 14.817 0.272 1.00 7.39 C

ATOM 1483 CE1 HIS A 97 15.669 13.585 -1.520 1.00 7.69 C

ATOM 1484 NE2 HIS A 97 16.465 13.919 -0.511 1.00 7.25 N

ATOM 1485 H HIS A 97 12.256 14.054 1.407 1.00 0.00 H

ATOM 1486 HA HIS A 97 14.036 16.176 2.100 1.00 0.00 H

ATOM 1487 HB3 HIS A 97 13.673 16.858 -0.312 1.00 0.00 H

ATOM 1488 HB2 HIS A 97 12.482 15.590 -0.456 1.00 0.00 H

ATOM 1489 HD2 HIS A 97 16.237 15.281 1.127 1.00 0.00 H

ATOM 1490 HE1 HIS A 97 15.943 12.917 -2.322 1.00 0.00 H

ATOM 1491 HE2 HIS A 97 17.427 13.619 -0.350 1.00 0.00 H

ATOM 1492 HD1 HIS A 97 13.729 14.121 -2.095 1.00 0.00 H

ATOM 1493 N LYS A 98 10.894 17.081 1.777 1.00 7.35 N

ATOM 1494 CA LYS A 98 9.916 18.143 2.038 1.00 7.96 C

ATOM 1495 C LYS A 98 9.769 18.488 3.534 1.00 8.54 C

ATOM 1496 O LYS A 98 9.327 19.594 3.843 1.00 9.46 O

ATOM 1497 CB LYS A 98 8.575 17.758 1.382 1.00 7.80 C

ATOM 1498 CG LYS A 98 8.632 17.837 -0.157 1.00 8.35 C

ATOM 1499 CD LYS A 98 7.467 17.115 -0.850 1.00 10.12 C

ATOM 1500 CE LYS A 98 7.635 17.083 -2.377 1.00 10.61 C

ATOM 1501 NZ LYS A 98 6.558 16.315 -3.024 1.00 14.64 N1+

ATOM 1502 H LYS A 98 10.513 16.188 1.493 1.00 0.00 H

ATOM 1503 HA LYS A 98 10.276 19.049 1.547 1.00 0.00 H

ATOM 1504 HB3 LYS A 98 7.779 18.420 1.726 1.00 0.00 H

ATOM 1505 HB2 LYS A 98 8.294 16.754 1.704 1.00 0.00 H

ATOM 1506 HG3 LYS A 98 9.573 17.424 -0.521 1.00 0.00 H

ATOM 1507 HG2 LYS A 98 8.639 18.885 -0.459 1.00 0.00 H

ATOM 1508 HD3 LYS A 98 6.531 17.611 -0.592 1.00 0.00 H

ATOM 1509 HD2 LYS A 98 7.390 16.096 -0.468 1.00 0.00 H

ATOM 1510 HE3 LYS A 98 8.589 16.627 -2.642 1.00 0.00 H

ATOM 1511 HE2 LYS A 98 7.640 18.096 -2.779 1.00 0.00 H

ATOM 1512 HZ1 LYS A 98 6.567 15.364 -2.682 1.00 0.00 H

ATOM 1513 HZ2 LYS A 98 5.665 16.739 -2.819 1.00 0.00 H

ATOM 1514 HZ3 LYS A 98 6.704 16.307 -4.024 1.00 0.00 H

ATOM 1515 N LEU A 99 10.203 17.577 4.422 1.00 9.03 N

ATOM 1516 CA LEU A 99 10.374 17.807 5.860 1.00 9.82 C

ATOM 1517 C LEU A 99 11.760 18.392 6.209 1.00 10.17 C

ATOM 1518 O LEU A 99 12.013 18.654 7.384 1.00 11.20 O

ATOM 1519 CB LEU A 99 10.129 16.476 6.604 1.00 9.90 C

ATOM 1520 CG LEU A 99 8.640 16.064 6.649 1.00 11.95 C

ATOM 1521 CD1 LEU A 99 8.489 14.583 7.028 1.00 12.95 C

ATOM 1522 CD2 LEU A 99 7.801 16.980 7.563 1.00 13.48 C

ATOM 1523 H LEU A 99 10.530 16.683 4.082 1.00 0.00 H

ATOM 1524 HA LEU A 99 9.641 18.540 6.199 1.00 0.00 H

ATOM 1525 HB3 LEU A 99 10.499 16.525 7.629 1.00 0.00 H

ATOM 1526 HB2 LEU A 99 10.721 15.697 6.121 1.00 0.00 H

ATOM 1527 HG LEU A 99 8.241 16.159 5.639 1.00 0.00 H

ATOM 1528 HD11 LEU A 99 7.644 14.133 6.508 1.00 0.00 H

ATOM 1529 HD12 LEU A 99 9.376 14.016 6.748 1.00 0.00 H

ATOM 1530 HD13 LEU A 99 8.335 14.445 8.098 1.00 0.00 H

ATOM 1531 HD21 LEU A 99 7.200 16.418 8.277 1.00 0.00 H

ATOM 1532 HD22 LEU A 99 8.424 17.665 8.139 1.00 0.00 H

ATOM 1533 HD23 LEU A 99 7.113 17.587 6.973 1.00 0.00 H

ATOM 1534 N GLY A 100 12.628 18.605 5.204 1.00 9.88 N

ATOM 1535 CA GLY A 100 13.950 19.203 5.372 1.00 10.06 C

ATOM 1536 C GLY A 100 14.992 18.194 5.878 1.00 10.19 C

ATOM 1537 O GLY A 100 16.006 18.623 6.426 1.00 12.13 O

ATOM 1538 H GLY A 100 12.352 18.377 4.258 1.00 0.00 H

ATOM 1539 HA3 GLY A 100 14.280 19.585 4.405 1.00 0.00 H

ATOM 1540 HA2 GLY A 100 13.900 20.057 6.049 1.00 0.00 H

ATOM 1541 N MET A 101 14.747 16.881 5.720 1.00 8.85 N

ATOM 1542 CA MET A 101 15.696 15.817 6.045 1.00 8.37 C

ATOM 1543 C MET A 101 16.322 15.252 4.765 1.00 7.87 C

ATOM 1544 O MET A 101 15.696 15.258 3.707 1.00 7.47 O

ATOM 1545 CB MET A 101 15.001 14.692 6.838 1.00 8.58 C

ATOM 1546 CG MET A 101 14.411 15.157 8.173 1.00 9.76 C

ATOM 1547 SD MET A 101 13.838 13.828 9.264 1.00 11.00 S

ATOM 1548 CE MET A 101 12.331 13.311 8.402 1.00 10.74 C

ATOM 1549 H MET A 101 13.895 16.582 5.263 1.00 0.00 H

ATOM 1550 HA MET A 101 16.496 16.217 6.665 1.00 0.00 H

ATOM 1551 HB3 MET A 101 15.705 13.880 7.028 1.00 0.00 H

ATOM 1552 HB2 MET A 101 14.202 14.264 6.235 1.00 0.00 H

ATOM 1553 HG3 MET A 101 13.575 15.826 7.976 1.00 0.00 H

ATOM 1554 HG2 MET A 101 15.157 15.732 8.723 1.00 0.00 H

ATOM 1555 HE1 MET A 101 11.866 12.488 8.943 1.00 0.00 H

ATOM 1556 HE2 MET A 101 11.619 14.135 8.346 1.00 0.00 H

ATOM 1557 HE3 MET A 101 12.554 12.971 7.391 1.00 0.00 H

ATOM 1558 N LYS A 102 17.546 14.735 4.906 1.00 6.02 N

ATOM 1559 CA LYS A 102 18.260 13.999 3.865 1.00 6.12 C

ATOM 1560 C LYS A 102 17.907 12.499 3.934 1.00 5.66 C

ATOM 1561 O LYS A 102 17.396 12.031 4.951 1.00 5.95 O

ATOM 1562 CB LYS A 102 19.764 14.266 4.060 1.00 6.33 C

ATOM 1563 CG LYS A 102 20.169 15.733 3.790 1.00 7.65 C

ATOM 1564 CD LYS A 102 20.526 15.976 2.320 1.00 9.10 C

ATOM 1565 CE LYS A 102 21.029 17.389 2.011 1.00 10.88 C

ATOM 1566 NZ LYS A 102 21.523 17.465 0.625 1.00 15.42 N1+

ATOM 1567 H LYS A 102 18.002 14.783 5.808 1.00 0.00 H

ATOM 1568 HA LYS A 102 17.963 14.373 2.884 1.00 0.00 H

ATOM 1569 HB3 LYS A 102 20.357 13.603 3.428 1.00 0.00 H

ATOM 1570 HB2 LYS A 102 20.034 14.009 5.080 1.00 0.00 H

ATOM 1571 HG3 LYS A 102 21.012 15.996 4.424 1.00 0.00 H

ATOM 1572 HG2 LYS A 102 19.374 16.418 4.084 1.00 0.00 H

ATOM 1573 HD3 LYS A 102 19.644 15.784 1.712 1.00 0.00 H

ATOM 1574 HD2 LYS A 102 21.286 15.256 2.020 1.00 0.00 H

ATOM 1575 HE3 LYS A 102 21.839 17.665 2.684 1.00 0.00 H

ATOM 1576 HE2 LYS A 102 20.229 18.116 2.153 1.00 0.00 H

ATOM 1577 HZ1 LYS A 102 22.301 16.830 0.512 1.00 0.00 H

ATOM 1578 HZ2 LYS A 102 20.784 17.198 -0.012 1.00 0.00 H

ATOM 1579 HZ3 LYS A 102 21.825 18.406 0.421 1.00 0.00 H

ATOM 1580 N PHE A 103 18.162 11.772 2.839 1.00 5.62 N

ATOM 1581 CA PHE A 103 17.852 10.349 2.711 1.00 5.85 C

ATOM 1582 C PHE A 103 19.100 9.648 2.162 1.00 6.13 C

ATOM 1583 O PHE A 103 19.567 10.018 1.086 1.00 6.64 O

ATOM 1584 CB PHE A 103 16.641 10.188 1.758 1.00 6.36 C

ATOM 1585 CG PHE A 103 15.988 8.811 1.739 1.00 5.85 C

ATOM 1586 CD1 PHE A 103 16.639 7.701 1.158 1.00 6.66 C

ATOM 1587 CD2 PHE A 103 14.753 8.605 2.385 1.00 6.83 C

ATOM 1588 CE1 PHE A 103 16.051 6.443 1.192 1.00 7.33 C

ATOM 1589 CE2 PHE A 103 14.182 7.340 2.413 1.00 7.07 C

ATOM 1590 CZ PHE A 103 14.823 6.265 1.813 1.00 7.01 C

ATOM 1591 H PHE A 103 18.565 12.219 2.021 1.00 0.00 H

ATOM 1592 HA PHE A 103 17.596 9.916 3.679 1.00 0.00 H

ATOM 1593 HB3 PHE A 103 16.935 10.432 0.736 1.00 0.00 H

ATOM 1594 HB2 PHE A 103 15.875 10.922 2.014 1.00 0.00 H

ATOM 1595 HD1 PHE A 103 17.601 7.821 0.692 1.00 0.00 H

ATOM 1596 HD2 PHE A 103 14.242 9.428 2.858 1.00 0.00 H

ATOM 1597 HE1 PHE A 103 16.555 5.600 0.742 1.00 0.00 H

ATOM 1598 HE2 PHE A 103 13.241 7.187 2.918 1.00 0.00 H

ATOM 1599 HZ PHE A 103 14.373 5.283 1.843 1.00 0.00 H

ATOM 1600 N ILE A 104 19.577 8.617 2.875 1.00 5.73 N

ATOM 1601 CA ILE A 104 20.604 7.693 2.393 1.00 5.91 C

ATOM 1602 C ILE A 104 20.040 6.260 2.430 1.00 5.52 C

ATOM 1603 O ILE A 104 19.165 5.961 3.245 1.00 5.40 O

ATOM 1604 CB ILE A 104 21.941 7.794 3.194 1.00 6.32 C

ATOM 1605 CG1 ILE A 104 21.989 7.118 4.584 1.00 6.86 C

ATOM 1606 CG2 ILE A 104 22.420 9.259 3.269 1.00 6.96 C

ATOM 1607 CD1 ILE A 104 23.395 7.097 5.202 1.00 6.21 C

ATOM 1608 H ILE A 104 19.147 8.375 3.760 1.00 0.00 H

ATOM 1609 HA ILE A 104 20.831 7.910 1.350 1.00 0.00 H

ATOM 1610 HB ILE A 104 22.677 7.265 2.591 1.00 0.00 H

ATOM 1611 HG13 ILE A 104 21.646 6.085 4.536 1.00 0.00 H

ATOM 1612 HG12 ILE A 104 21.311 7.641 5.257 1.00 0.00 H

ATOM 1613 HG21 ILE A 104 23.466 9.327 3.564 1.00 0.00 H

ATOM 1614 HG22 ILE A 104 22.323 9.759 2.307 1.00 0.00 H

ATOM 1615 HG23 ILE A 104 21.836 9.831 3.991 1.00 0.00 H

ATOM 1616 HD11 ILE A 104 23.485 6.312 5.952 1.00 0.00 H

ATOM 1617 HD12 ILE A 104 24.166 6.922 4.451 1.00 0.00 H

ATOM 1618 HD13 ILE A 104 23.610 8.044 5.694 1.00 0.00 H

ATOM 1619 N THR A 105 20.539 5.411 1.520 1.00 5.10 N

ATOM 1620 CA THR A 105 20.070 4.037 1.339 1.00 5.19 C

ATOM 1621 C THR A 105 21.242 3.053 1.522 1.00 4.98 C

ATOM 1622 O THR A 105 22.393 3.440 1.340 1.00 4.78 O

ATOM 1623 CB THR A 105 19.442 3.878 -0.074 1.00 5.25 C

ATOM 1624 OG1 THR A 105 18.707 2.676 -0.135 1.00 6.76 O

ATOM 1625 CG2 THR A 105 20.436 3.865 -1.254 1.00 5.38 C

ATOM 1626 H THR A 105 21.267 5.719 0.890 1.00 0.00 H

ATOM 1627 HA THR A 105 19.316 3.783 2.087 1.00 0.00 H

ATOM 1628 HB THR A 105 18.733 4.692 -0.231 1.00 0.00 H

ATOM 1629 HG1 THR A 105 19.156 2.059 -0.724 1.00 0.00 H

ATOM 1630 HG21 THR A 105 19.910 3.910 -2.208 1.00 0.00 H

ATOM 1631 HG22 THR A 105 21.114 4.716 -1.210 1.00 0.00 H

ATOM 1632 HG23 THR A 105 21.052 2.965 -1.267 1.00 0.00 H

ATOM 1633 N ASP A 106 20.948 1.798 1.892 1.00 4.70 N

ATOM 1634 CA ASP A 106 21.980 0.796 2.168 1.00 5.17 C

ATOM 1635 C ASP A 106 22.499 0.178 0.855 1.00 5.53 C

ATOM 1636 O ASP A 106 21.718 -0.404 0.112 1.00 6.05 O

ATOM 1637 CB ASP A 106 21.457 -0.281 3.136 1.00 5.36 C

ATOM 1638 CG ASP A 106 22.514 -0.710 4.153 1.00 5.29 C

ATOM 1639 OD1 ASP A 106 23.622 -1.102 3.726 1.00 5.45 O

ATOM 1640 OD2 ASP A 106 22.180 -0.680 5.357 1.00 5.27 O1-

ATOM 1641 H ASP A 106 19.987 1.517 2.028 1.00 0.00 H

ATOM 1642 HA ASP A 106 22.800 1.319 2.660 1.00 0.00 H

ATOM 1643 HB3 ASP A 106 21.090 -1.165 2.615 1.00 0.00 H

ATOM 1644 HB2 ASP A 106 20.599 0.110 3.686 1.00 0.00 H

ATOM 1645 N LEU A 107 23.804 0.312 0.592 1.00 4.91 N

ATOM 1646 CA LEU A 107 24.478 -0.169 -0.611 1.00 4.58 C

ATOM 1647 C LEU A 107 25.216 -1.476 -0.284 1.00 4.66 C

ATOM 1648 O LEU A 107 26.300 -1.432 0.296 1.00 4.49 O

ATOM 1649 CB LEU A 107 25.416 0.955 -1.104 1.00 5.21 C

ATOM 1650 CG LEU A 107 26.200 0.644 -2.392 1.00 4.89 C

ATOM 1651 CD1 LEU A 107 25.249 0.459 -3.586 1.00 6.64 C

ATOM 1652 CD2 LEU A 107 27.265 1.721 -2.667 1.00 5.02 C

ATOM 1653 H LEU A 107 24.395 0.764 1.280 1.00 0.00 H

ATOM 1654 HA LEU A 107 23.743 -0.367 -1.393 1.00 0.00 H

ATOM 1655 HB3 LEU A 107 26.120 1.213 -0.314 1.00 0.00 H

ATOM 1656 HB2 LEU A 107 24.824 1.852 -1.266 1.00 0.00 H

ATOM 1657 HG LEU A 107 26.740 -0.291 -2.258 1.00 0.00 H

ATOM 1658 HD11 LEU A 107 25.774 0.471 -4.538 1.00 0.00 H

ATOM 1659 HD12 LEU A 107 24.732 -0.497 -3.524 1.00 0.00 H

ATOM 1660 HD13 LEU A 107 24.491 1.243 -3.613 1.00 0.00 H

ATOM 1661 HD21 LEU A 107 27.547 1.764 -3.716 1.00 0.00 H

ATOM 1662 HD22 LEU A 107 26.926 2.714 -2.371 1.00 0.00 H

ATOM 1663 HD23 LEU A 107 28.179 1.504 -2.116 1.00 0.00 H

ATOM 1664 N VAL A 108 24.601 -2.614 -0.644 1.00 4.52 N

ATOM 1665 CA VAL A 108 25.066 -3.949 -0.264 1.00 4.45 C

ATOM 1666 C VAL A 108 25.566 -4.687 -1.518 1.00 4.83 C

ATOM 1667 O VAL A 108 24.796 -5.385 -2.177 1.00 4.77 O

ATOM 1668 CB VAL A 108 23.927 -4.764 0.417 1.00 4.60 C

ATOM 1669 CG1 VAL A 108 24.411 -6.123 0.954 1.00 4.22 C

ATOM 1670 CG2 VAL A 108 23.197 -3.977 1.522 1.00 4.43 C

ATOM 1671 H VAL A 108 23.712 -2.573 -1.121 1.00 0.00 H

ATOM 1672 HA VAL A 108 25.909 -3.889 0.421 1.00 0.00 H

ATOM 1673 HB VAL A 108 23.164 -4.987 -0.321 1.00 0.00 H

ATOM 1674 HG11 VAL A 108 23.631 -6.612 1.533 1.00 0.00 H

ATOM 1675 HG12 VAL A 108 24.693 -6.808 0.154 1.00 0.00 H

ATOM 1676 HG13 VAL A 108 25.275 -6.004 1.607 1.00 0.00 H

ATOM 1677 HG21 VAL A 108 22.407 -4.579 1.972 1.00 0.00 H

ATOM 1678 HG22 VAL A 108 23.877 -3.673 2.315 1.00 0.00 H

ATOM 1679 HG23 VAL A 108 22.717 -3.078 1.137 1.00 0.00 H

ATOM 1680 N ILE A 109 26.842 -4.452 -1.862 1.00 4.36 N

ATOM 1681 CA ILE A 109 27.427 -4.818 -3.157 1.00 4.50 C

ATOM 1682 C ILE A 109 28.760 -5.590 -3.049 1.00 4.83 C

ATOM 1683 O ILE A 109 29.397 -5.821 -4.076 1.00 5.25 O

ATOM 1684 CB ILE A 109 27.592 -3.567 -4.068 1.00 3.77 C

ATOM 1685 CG1 ILE A 109 28.464 -2.467 -3.424 1.00 4.09 C

ATOM 1686 CG2 ILE A 109 26.218 -3.029 -4.502 1.00 5.75 C

ATOM 1687 CD1 ILE A 109 28.745 -1.251 -4.322 1.00 4.72 C

ATOM 1688 H ILE A 109 27.408 -3.860 -1.272 1.00 0.00 H

ATOM 1689 HA ILE A 109 26.765 -5.515 -3.674 1.00 0.00 H

ATOM 1690 HB ILE A 109 28.094 -3.876 -4.984 1.00 0.00 H

ATOM 1691 HG13 ILE A 109 29.416 -2.902 -3.131 1.00 0.00 H

ATOM 1692 HG12 ILE A 109 28.004 -2.121 -2.499 1.00 0.00 H

ATOM 1693 HG21 ILE A 109 26.320 -2.195 -5.193 1.00 0.00 H

ATOM 1694 HG22 ILE A 109 25.638 -3.799 -5.011 1.00 0.00 H

ATOM 1695 HG23 ILE A 109 25.633 -2.678 -3.653 1.00 0.00 H

ATOM 1696 HD11 ILE A 109 29.557 -0.649 -3.914 1.00 0.00 H

ATOM 1697 HD12 ILE A 109 29.028 -1.546 -5.329 1.00 0.00 H

ATOM 1698 HD13 ILE A 109 27.873 -0.607 -4.408 1.00 0.00 H

ATOM 1699 N ASN A 110 29.120 -6.066 -1.842 1.00 4.65 N

ATOM 1700 CA ASN A 110 30.099 -7.151 -1.678 1.00 5.02 C

ATOM 1701 C ASN A 110 29.527 -8.491 -2.191 1.00 5.62 C

ATOM 1702 O ASN A 110 30.283 -9.322 -2.689 1.00 5.74 O

ATOM 1703 CB ASN A 110 30.522 -7.258 -0.190 1.00 5.17 C

ATOM 1704 CG ASN A 110 31.589 -8.335 0.051 1.00 5.43 C

ATOM 1705 OD1 ASN A 110 32.659 -8.291 -0.552 1.00 7.33 O

ATOM 1706 ND2 ASN A 110 31.294 -9.317 0.905 1.00 3.81 N

ATOM 1707 H ASN A 110 28.568 -5.835 -1.029 1.00 0.00 H

ATOM 1708 HA ASN A 110 30.981 -6.912 -2.276 1.00 0.00 H

ATOM 1709 HB3 ASN A 110 29.652 -7.451 0.439 1.00 0.00 H

ATOM 1710 HB2 ASN A 110 30.933 -6.308 0.152 1.00 0.00 H

ATOM 1711 HD22 ASN A 110 31.942 -10.080 1.046 1.00 0.00 H

ATOM 1712 HD21 ASN A 110 30.393 -9.340 1.363 1.00 0.00 H

ATOM 1713 N HIS A 111 28.204 -8.649 -2.050 1.00 5.38 N

ATOM 1714 CA HIS A 111 27.442 -9.849 -2.353 1.00 5.42 C

ATOM 1715 C HIS A 111 26.021 -9.455 -2.777 1.00 5.85 C

ATOM 1716 O HIS A 111 25.561 -8.353 -2.473 1.00 5.20 O

ATOM 1717 CB HIS A 111 27.420 -10.775 -1.112 1.00 6.04 C

ATOM 1718 CG HIS A 111 26.997 -10.105 0.175 1.00 5.35 C

ATOM 1719 ND1 HIS A 111 27.914 -9.527 1.060 1.00 6.45 N

ATOM 1720 CD2 HIS A 111 25.723 -9.910 0.666 1.00 5.78 C

ATOM 1721 CE1 HIS A 111 27.171 -8.991 2.020 1.00 6.90 C

ATOM 1722 NE2 HIS A 111 25.866 -9.187 1.837 1.00 6.78 N

ATOM 1723 H HIS A 111 27.661 -7.893 -1.656 1.00 0.00 H

ATOM 1724 HA HIS A 111 27.904 -10.362 -3.197 1.00 0.00 H

ATOM 1725 HB3 HIS A 111 28.412 -11.199 -0.952 1.00 0.00 H

ATOM 1726 HB2 HIS A 111 26.762 -11.627 -1.286 1.00 0.00 H

ATOM 1727 HD2 HIS A 111 24.758 -10.195 0.273 1.00 0.00 H

ATOM 1728 HE1 HIS A 111 27.582 -8.452 2.862 1.00 0.00 H

ATOM 1729 HE2 HIS A 111 25.124 -8.856 2.438 1.00 0.00 H

ATOM 1730 N CYS A 112 25.347 -10.399 -3.439 1.00 5.68 N

ATOM 1731 CA CYS A 112 23.929 -10.335 -3.783 1.00 6.28 C

ATOM 1732 C CYS A 112 23.299 -11.701 -3.460 1.00 5.90 C

ATOM 1733 O CYS A 112 24.021 -12.658 -3.169 1.00 5.99 O

ATOM 1734 CB CYS A 112 23.727 -9.869 -5.247 1.00 6.25 C

ATOM 1735 SG CYS A 112 24.070 -11.150 -6.489 1.00 7.17 S

ATOM 1736 H CYS A 112 25.799 -11.284 -3.632 1.00 0.00 H

ATOM 1737 HA CYS A 112 23.437 -9.611 -3.131 1.00 0.00 H

ATOM 1738 HB3 CYS A 112 24.356 -9.006 -5.461 1.00 0.00 H

ATOM 1739 HB2 CYS A 112 22.701 -9.538 -5.397 1.00 0.00 H

ATOM 1740 HG CYS A 112 23.065 -11.962 -6.142 1.00 0.00 H

ATOM 1741 N SER A 113 21.961 -11.781 -3.537 1.00 6.04 N

ATOM 1742 CA SER A 113 21.222 -13.035 -3.383 1.00 6.25 C

ATOM 1743 C SER A 113 21.575 -14.042 -4.492 1.00 6.57 C

ATOM 1744 O SER A 113 21.846 -13.628 -5.621 1.00 6.75 O

ATOM 1745 CB SER A 113 19.707 -12.758 -3.387 1.00 6.72 C

ATOM 1746 OG SER A 113 18.961 -13.896 -3.004 1.00 6.48 O

ATOM 1747 H SER A 113 21.418 -10.966 -3.795 1.00 0.00 H

ATOM 1748 HA SER A 113 21.494 -13.433 -2.412 1.00 0.00 H

ATOM 1749 HB3 SER A 113 19.374 -12.439 -4.375 1.00 0.00 H

ATOM 1750 HB2 SER A 113 19.461 -11.959 -2.699 1.00 0.00 H

ATOM 1751 HG SER A 113 19.008 -13.981 -2.047 1.00 0.00 H

ATOM 1752 N THR A 114 21.468 -15.346 -4.182 1.00 6.96 N

ATOM 1753 CA THR A 114 21.434 -16.400 -5.204 1.00 7.27 C

ATOM 1754 C THR A 114 20.181 -16.316 -6.105 1.00 7.63 C

ATOM 1755 O THR A 114 20.214 -16.844 -7.216 1.00 7.80 O

ATOM 1756 CB THR A 114 21.460 -17.826 -4.594 1.00 0.00 C

ATOM 1757 OG1 THR A 114 20.346 -18.075 -3.754 1.00 0.00 O

ATOM 1758 CG2 THR A 114 22.760 -18.144 -3.852 1.00 0.00 C

ATOM 1759 HB THR A 114 21.382 -18.556 -5.401 1.00 0.00 H

ATOM 1760 HG1 THR A 114 19.547 -18.033 -4.288 1.00 0.00 H

ATOM 1761 HG21 THR A 114 22.670 -19.056 -3.262 1.00 0.00 H

ATOM 1762 HG22 THR A 114 23.570 -18.306 -4.559 1.00 0.00 H

ATOM 1763 HG23 THR A 114 23.061 -17.326 -3.201 1.00 0.00 H

ATOM 1764 H THR A 114 21.247 -15.627 -3.237 1.00 0.00 H

ATOM 1765 HA THR A 114 22.316 -16.264 -5.825 1.00 0.00 H

ATOM 1766 N GLU A 115 19.126 -15.633 -5.627 1.00 7.32 N

ATOM 1767 CA GLU A 115 17.909 -15.348 -6.378 1.00 7.92 C

ATOM 1768 C GLU A 115 18.013 -14.115 -7.292 1.00 7.79 C

ATOM 1769 O GLU A 115 17.064 -13.870 -8.038 1.00 8.78 O

ATOM 1770 CB GLU A 115 16.707 -15.246 -5.413 1.00 8.37 C

ATOM 1771 CG GLU A 115 16.440 -16.523 -4.587 1.00 10.02 C

ATOM 1772 CD GLU A 115 16.232 -17.773 -5.447 1.00 15.51 C

ATOM 1773 OE1 GLU A 115 15.409 -17.696 -6.387 1.00 17.51 O

ATOM 1774 OE2 GLU A 115 16.926 -18.775 -5.162 1.00 18.20 O1-

ATOM 1775 H GLU A 115 19.176 -15.223 -4.703 1.00 0.00 H

ATOM 1776 HA GLU A 115 17.738 -16.187 -7.050 1.00 0.00 H

ATOM 1777 HB3 GLU A 115 15.802 -14.996 -5.970 1.00 0.00 H

ATOM 1778 HB2 GLU A 115 16.860 -14.413 -4.727 1.00 0.00 H

ATOM 1779 HG3 GLU A 115 15.548 -16.383 -3.977 1.00 0.00 H

ATOM 1780 HG2 GLU A 115 17.261 -16.696 -3.890 1.00 0.00 H

ATOM 1781 N HIS A 116 19.147 -13.386 -7.275 1.00 7.67 N

ATOM 1782 CA HIS A 116 19.448 -12.371 -8.288 1.00 7.42 C

ATOM 1783 C HIS A 116 19.667 -13.040 -9.652 1.00 7.35 C

ATOM 1784 O HIS A 116 20.321 -14.080 -9.712 1.00 7.71 O

ATOM 1785 CB HIS A 116 20.672 -11.523 -7.865 1.00 7.81 C

ATOM 1786 CG HIS A 116 20.742 -10.159 -8.520 1.00 8.15 C

ATOM 1787 ND1 HIS A 116 20.841 -9.991 -9.906 1.00 7.65 N

ATOM 1788 CD2 HIS A 116 20.707 -8.910 -7.933 1.00 8.43 C

ATOM 1789 CE1 HIS A 116 20.807 -8.682 -10.102 1.00 9.28 C

ATOM 1790 NE2 HIS A 116 20.738 -7.990 -8.967 1.00 8.23 N

ATOM 1791 H HIS A 116 19.901 -13.637 -6.652 1.00 0.00 H

ATOM 1792 HA HIS A 116 18.576 -11.727 -8.355 1.00 0.00 H

ATOM 1793 HB3 HIS A 116 21.599 -12.058 -8.076 1.00 0.00 H

ATOM 1794 HB2 HIS A 116 20.660 -11.364 -6.787 1.00 0.00 H

ATOM 1795 HD2 HIS A 116 20.639 -8.610 -6.897 1.00 0.00 H

ATOM 1796 HE1 HIS A 116 20.844 -8.223 -11.079 1.00 0.00 H

ATOM 1797 HE2 HIS A 116 20.715 -6.985 -8.883 1.00 0.00 H

ATOM 1798 N GLU A 117 19.132 -12.412 -10.712 1.00 7.69 N

ATOM 1799 CA GLU A 117 19.260 -12.849 -12.104 1.00 8.38 C

ATOM 1800 C GLU A 117 20.710 -12.982 -12.605 1.00 8.01 C

ATOM 1801 O GLU A 117 20.957 -13.829 -13.463 1.00 8.46 O

ATOM 1802 CB GLU A 117 18.436 -11.923 -13.026 1.00 8.39 C

ATOM 1803 CG GLU A 117 16.905 -12.040 -12.854 1.00 11.28 C

ATOM 1804 CD GLU A 117 16.331 -13.421 -13.197 1.00 15.47 C

ATOM 1805 OE1 GLU A 117 16.935 -14.114 -14.045 1.00 18.63 O

ATOM 1806 OE2 GLU A 117 15.289 -13.766 -12.597 1.00 19.17 O1-

ATOM 1807 H GLU A 117 18.614 -11.557 -10.566 1.00 0.00 H

ATOM 1808 HA GLU A 117 18.839 -13.852 -12.149 1.00 0.00 H

ATOM 1809 HB3 GLU A 117 18.691 -12.109 -14.071 1.00 0.00 H

ATOM 1810 HB2 GLU A 117 18.727 -10.888 -12.842 1.00 0.00 H

ATOM 1811 HG3 GLU A 117 16.414 -11.310 -13.497 1.00 0.00 H

ATOM 1812 HG2 GLU A 117 16.625 -11.784 -11.831 1.00 0.00 H

ATOM 1813 N TRP A 118 21.645 -12.202 -12.028 1.00 7.58 N

ATOM 1814 CA TRP A 118 23.085 -12.358 -12.238 1.00 7.36 C

ATOM 1815 C TRP A 118 23.600 -13.725 -11.770 1.00 7.80 C

ATOM 1816 O TRP A 118 24.337 -14.357 -12.524 1.00 8.53 O

ATOM 1817 CB TRP A 118 23.884 -11.248 -11.526 1.00 7.75 C

ATOM 1818 CG TRP A 118 23.623 -9.822 -11.904 1.00 7.25 C

ATOM 1819 CD1 TRP A 118 23.036 -9.378 -13.040 1.00 7.96 C

ATOM 1820 CD2 TRP A 118 23.994 -8.627 -11.154 1.00 7.92 C

ATOM 1821 NE1 TRP A 118 22.993 -8.000 -13.031 1.00 8.88 N

ATOM 1822 CE2 TRP A 118 23.562 -7.480 -11.886 1.00 8.02 C

ATOM 1823 CE3 TRP A 118 24.635 -8.396 -9.913 1.00 7.52 C

ATOM 1824 CZ2 TRP A 118 23.737 -6.172 -11.405 1.00 8.60 C

ATOM 1825 CZ3 TRP A 118 24.813 -7.088 -9.418 1.00 8.55 C

ATOM 1826 CH2 TRP A 118 24.352 -5.981 -10.155 1.00 7.80 C

ATOM 1827 H TRP A 118 21.363 -11.522 -11.333 1.00 0.00 H

ATOM 1828 HA TRP A 118 23.271 -12.285 -13.310 1.00 0.00 H

ATOM 1829 HB3 TRP A 118 24.951 -11.417 -11.683 1.00 0.00 H

ATOM 1830 HB2 TRP A 118 23.726 -11.322 -10.449 1.00 0.00 H

ATOM 1831 HD1 TRP A 118 22.645 -10.016 -13.819 1.00 0.00 H

ATOM 1832 HE1 TRP A 118 22.576 -7.461 -13.777 1.00 0.00 H

ATOM 1833 HE3 TRP A 118 24.975 -9.236 -9.327 1.00 0.00 H

ATOM 1834 HZ2 TRP A 118 23.388 -5.324 -11.975 1.00 0.00 H

ATOM 1835 HZ3 TRP A 118 25.287 -6.935 -8.460 1.00 0.00 H

ATOM 1836 HH2 TRP A 118 24.458 -4.984 -9.759 1.00 0.00 H

ATOM 1837 N PHE A 119 23.199 -14.156 -10.557 1.00 7.59 N

ATOM 1838 CA PHE A 119 23.617 -15.442 -10.002 1.00 8.01 C

ATOM 1839 C PHE A 119 22.855 -16.637 -10.596 1.00 8.21 C

ATOM 1840 O PHE A 119 23.469 -17.689 -10.761 1.00 8.53 O

ATOM 1841 CB PHE A 119 23.602 -15.457 -8.462 1.00 8.26 C

ATOM 1842 CG PHE A 119 24.319 -16.683 -7.910 1.00 7.50 C

ATOM 1843 CD1 PHE A 119 25.722 -16.761 -8.025 1.00 7.65 C

ATOM 1844 CD2 PHE A 119 23.613 -17.839 -7.517 1.00 8.32 C

ATOM 1845 CE1 PHE A 119 26.390 -17.937 -7.721 1.00 7.08 C

ATOM 1846 CE2 PHE A 119 24.302 -18.992 -7.166 1.00 7.51 C

ATOM 1847 CZ PHE A 119 25.683 -19.042 -7.273 1.00 7.93 C

ATOM 1848 H PHE A 119 22.560 -13.604 -10.002 1.00 0.00 H

ATOM 1849 HA PHE A 119 24.661 -15.557 -10.284 1.00 0.00 H

ATOM 1850 HB3 PHE A 119 22.585 -15.388 -8.074 1.00 0.00 H

ATOM 1851 HB2 PHE A 119 24.130 -14.577 -8.091 1.00 0.00 H

ATOM 1852 HD1 PHE A 119 26.275 -15.913 -8.387 1.00 0.00 H

ATOM 1853 HD2 PHE A 119 22.535 -17.840 -7.504 1.00 0.00 H

ATOM 1854 HE1 PHE A 119 27.463 -17.993 -7.831 1.00 0.00 H

ATOM 1855 HE2 PHE A 119 23.765 -19.867 -6.838 1.00 0.00 H

ATOM 1856 HZ PHE A 119 26.207 -19.951 -7.028 1.00 0.00 H

ATOM 1857 N LYS A 120 21.566 -16.459 -10.943 1.00 8.67 N

ATOM 1858 CA LYS A 120 20.774 -17.458 -11.669 1.00 9.03 C

ATOM 1859 C LYS A 120 21.387 -17.807 -13.035 1.00 8.88 C

ATOM 1860 O LYS A 120 21.438 -18.988 -13.376 1.00 8.80 O

ATOM 1861 CB LYS A 120 19.316 -16.994 -11.843 1.00 9.77 C

ATOM 1862 CG LYS A 120 18.484 -17.024 -10.552 1.00 11.58 C

ATOM 1863 CD LYS A 120 17.003 -16.721 -10.824 1.00 15.77 C

ATOM 1864 CE LYS A 120 16.116 -16.920 -9.590 1.00 18.44 C

ATOM 1865 NZ LYS A 120 14.702 -16.656 -9.895 1.00 22.14 N1+

ATOM 1866 H LYS A 120 21.112 -15.574 -10.752 1.00 0.00 H

ATOM 1867 HA LYS A 120 20.773 -18.369 -11.067 1.00 0.00 H

ATOM 1868 HB3 LYS A 120 18.826 -17.661 -12.555 1.00 0.00 H

ATOM 1869 HB2 LYS A 120 19.285 -16.007 -12.300 1.00 0.00 H

ATOM 1870 HG3 LYS A 120 18.880 -16.309 -9.831 1.00 0.00 H

ATOM 1871 HG2 LYS A 120 18.573 -18.007 -10.086 1.00 0.00 H

ATOM 1872 HD3 LYS A 120 16.642 -17.351 -11.638 1.00 0.00 H

ATOM 1873 HD2 LYS A 120 16.912 -15.691 -11.173 1.00 0.00 H

ATOM 1874 HE3 LYS A 120 16.418 -16.247 -8.795 1.00 0.00 H

ATOM 1875 HE2 LYS A 120 16.211 -17.937 -9.207 1.00 0.00 H

ATOM 1876 HZ1 LYS A 120 14.380 -17.299 -10.603 1.00 0.00 H

ATOM 1877 HZ2 LYS A 120 14.156 -16.773 -9.053 1.00 0.00 H

ATOM 1878 HZ3 LYS A 120 14.605 -15.708 -10.232 1.00 0.00 H

ATOM 1879 N GLU A 121 21.892 -16.784 -13.750 1.00 8.62 N

ATOM 1880 CA GLU A 121 22.672 -16.954 -14.971 1.00 8.97 C

ATOM 1881 C GLU A 121 24.043 -17.592 -14.690 1.00 8.89 C

ATOM 1882 O GLU A 121 24.362 -18.575 -15.351 1.00 9.20 O

ATOM 1883 CB GLU A 121 22.765 -15.621 -15.742 1.00 9.37 C

ATOM 1884 CG GLU A 121 23.531 -15.726 -17.081 1.00 10.60 C

ATOM 1885 CD GLU A 121 23.544 -14.438 -17.906 1.00 12.07 C

ATOM 1886 OE1 GLU A 121 24.324 -14.403 -18.881 1.00 13.86 O

ATOM 1887 OE2 GLU A 121 22.798 -13.496 -17.556 1.00 12.04 O1-

ATOM 1888 H GLU A 121 21.793 -15.837 -13.410 1.00 0.00 H

ATOM 1889 HA GLU A 121 22.119 -17.648 -15.608 1.00 0.00 H

ATOM 1890 HB3 GLU A 121 23.235 -14.865 -15.111 1.00 0.00 H

ATOM 1891 HB2 GLU A 121 21.751 -15.265 -15.933 1.00 0.00 H

ATOM 1892 HG3 GLU A 121 23.094 -16.514 -17.696 1.00 0.00 H

ATOM 1893 HG2 GLU A 121 24.567 -16.008 -16.904 1.00 0.00 H

ATOM 1894 N SER A 122 24.797 -17.063 -13.707 1.00 8.65 N

ATOM 1895 CA SER A 122 26.134 -17.528 -13.309 1.00 8.30 C

ATOM 1896 C SER A 122 26.217 -19.033 -12.993 1.00 8.80 C

ATOM 1897 O SER A 122 27.157 -19.685 -13.446 1.00 8.62 O

ATOM 1898 CB SER A 122 26.635 -16.709 -12.105 1.00 8.64 C

ATOM 1899 OG SER A 122 27.975 -17.015 -11.786 1.00 8.72 O

ATOM 1900 H SER A 122 24.453 -16.250 -13.213 1.00 0.00 H

ATOM 1901 HA SER A 122 26.801 -17.327 -14.146 1.00 0.00 H

ATOM 1902 HB3 SER A 122 26.035 -16.922 -11.223 1.00 0.00 H

ATOM 1903 HB2 SER A 122 26.565 -15.641 -12.304 1.00 0.00 H

ATOM 1904 HG SER A 122 28.544 -16.546 -12.405 1.00 0.00 H

ATOM 1905 N ARG A 123 25.230 -19.542 -12.238 1.00 8.45 N

ATOM 1906 CA ARG A 123 25.163 -20.933 -11.792 1.00 9.06 C

ATOM 1907 C ARG A 123 24.603 -21.911 -12.838 1.00 9.82 C

ATOM 1908 O ARG A 123 24.689 -23.115 -12.601 1.00 10.27 O

ATOM 1909 CB ARG A 123 24.381 -21.009 -10.461 1.00 8.81 C

ATOM 1910 CG ARG A 123 22.838 -21.029 -10.563 1.00 9.01 C

ATOM 1911 CD ARG A 123 22.141 -21.026 -9.195 1.00 9.15 C

ATOM 1912 NE ARG A 123 22.632 -22.124 -8.347 1.00 10.40 N

ATOM 1913 CZ ARG A 123 22.405 -22.292 -7.035 1.00 9.87 C

ATOM 1914 NH1 ARG A 123 21.659 -21.426 -6.334 1.00 11.81 N

ATOM 1915 NH2 ARG A 123 22.944 -23.353 -6.423 1.00 11.26 N1+

ATOM 1916 H ARG A 123 24.497 -18.930 -11.901 1.00 0.00 H

ATOM 1917 HA ARG A 123 26.180 -21.272 -11.589 1.00 0.00 H

ATOM 1918 HB3 ARG A 123 24.697 -20.199 -9.803 1.00 0.00 H

ATOM 1919 HB2 ARG A 123 24.695 -21.929 -9.966 1.00 0.00 H

ATOM 1920 HG3 ARG A 123 22.449 -21.846 -11.173 1.00 0.00 H

ATOM 1921 HG2 ARG A 123 22.549 -20.114 -11.078 1.00 0.00 H

ATOM 1922 HD3 ARG A 123 21.081 -21.234 -9.337 1.00 0.00 H

ATOM 1923 HD2 ARG A 123 22.204 -20.046 -8.726 1.00 0.00 H

ATOM 1924 HE ARG A 123 23.167 -22.839 -8.824 1.00 0.00 H

ATOM 1925 HH12 ARG A 123 21.504 -21.563 -5.346 1.00 0.00 H

ATOM 1926 HH11 ARG A 123 21.236 -20.637 -6.801 1.00 0.00 H

ATOM 1927 HH22 ARG A 123 22.806 -23.510 -5.436 1.00 0.00 H

ATOM 1928 HH21 ARG A 123 23.478 -24.030 -6.964 1.00 0.00 H

ATOM 1929 N SER A 124 24.018 -21.410 -13.942 1.00 10.23 N

ATOM 1930 CA SER A 124 23.381 -22.239 -14.973 1.00 11.21 C

ATOM 1931 C SER A 124 24.356 -23.137 -15.764 1.00 11.56 C

ATOM 1932 O SER A 124 23.922 -24.161 -16.290 1.00 12.49 O

ATOM 1933 CB SER A 124 22.521 -21.355 -15.905 1.00 10.69 C

ATOM 1934 OG SER A 124 23.282 -20.648 -16.867 1.00 12.53 O

ATOM 1935 H SER A 124 23.978 -20.409 -14.079 1.00 0.00 H

ATOM 1936 HA SER A 124 22.694 -22.906 -14.448 1.00 0.00 H

ATOM 1937 HB3 SER A 124 21.923 -20.650 -15.330 1.00 0.00 H

ATOM 1938 HB2 SER A 124 21.813 -21.982 -16.448 1.00 0.00 H

ATOM 1939 HG SER A 124 23.728 -19.917 -16.424 1.00 0.00 H

ATOM 1940 N SER A 125 25.637 -22.739 -15.833 1.00 11.78 N

ATOM 1941 CA SER A 125 26.692 -23.401 -16.598 1.00 12.92 C

ATOM 1942 C SER A 125 28.064 -22.872 -16.137 1.00 13.04 C

ATOM 1943 O SER A 125 28.131 -21.904 -15.378 1.00 12.96 O

ATOM 1944 CB SER A 125 26.440 -23.169 -18.112 1.00 12.91 C

ATOM 1945 OG SER A 125 27.367 -23.858 -18.929 1.00 13.86 O

ATOM 1946 H SER A 125 25.921 -21.907 -15.335 1.00 0.00 H

ATOM 1947 HA SER A 125 26.657 -24.470 -16.380 1.00 0.00 H

ATOM 1948 HB3 SER A 125 26.491 -22.106 -18.350 1.00 0.00 H

ATOM 1949 HB2 SER A 125 25.444 -23.506 -18.400 1.00 0.00 H

ATOM 1950 HG SER A 125 27.209 -23.615 -19.846 1.00 0.00 H

ATOM 1951 N LYS A 126 29.144 -23.501 -16.627 1.00 13.71 N

ATOM 1952 CA LYS A 126 30.512 -22.998 -16.484 1.00 14.33 C

ATOM 1953 C LYS A 126 30.915 -22.011 -17.602 1.00 14.63 C

ATOM 1954 O LYS A 126 31.970 -21.389 -17.483 1.00 15.31 O

ATOM 1955 CB LYS A 126 31.502 -24.176 -16.449 1.00 14.66 C

ATOM 1956 CG LYS A 126 31.309 -25.108 -15.244 1.00 16.23 C

ATOM 1957 CD LYS A 126 32.555 -25.962 -14.979 1.00 19.68 C

ATOM 1958 CE LYS A 126 32.304 -27.030 -13.913 1.00 22.36 C

ATOM 1959 NZ LYS A 126 33.559 -27.643 -13.448 1.00 25.09 N1+

ATOM 1960 H LYS A 126 29.017 -24.289 -17.246 1.00 0.00 H

ATOM 1961 HA LYS A 126 30.598 -22.480 -15.529 1.00 0.00 H

ATOM 1962 HB3 LYS A 126 32.515 -23.772 -16.400 1.00 0.00 H

ATOM 1963 HB2 LYS A 126 31.452 -24.751 -17.375 1.00 0.00 H

ATOM 1964 HG3 LYS A 126 30.445 -25.752 -15.416 1.00 0.00 H

ATOM 1965 HG2 LYS A 126 31.082 -24.523 -14.352 1.00 0.00 H

ATOM 1966 HD3 LYS A 126 33.369 -25.311 -14.655 1.00 0.00 H

ATOM 1967 HD2 LYS A 126 32.887 -26.436 -15.904 1.00 0.00 H

ATOM 1968 HE3 LYS A 126 31.643 -27.804 -14.304 1.00 0.00 H

ATOM 1969 HE2 LYS A 126 31.805 -26.578 -13.057 1.00 0.00 H

ATOM 1970 HZ1 LYS A 126 34.142 -26.925 -13.032 1.00 0.00 H

ATOM 1971 HZ2 LYS A 126 33.350 -28.343 -12.752 1.00 0.00 H

ATOM 1972 HZ3 LYS A 126 34.044 -28.068 -14.225 1.00 0.00 H

ATOM 1973 N THR A 127 30.110 -21.917 -18.674 1.00 14.80 N

ATOM 1974 CA THR A 127 30.481 -21.265 -19.934 1.00 15.41 C

ATOM 1975 C THR A 127 29.720 -19.951 -20.214 1.00 15.01 C

ATOM 1976 O THR A 127 30.053 -19.287 -21.197 1.00 15.64 O

ATOM 1977 CB THR A 127 30.214 -22.231 -21.122 1.00 15.44 C

ATOM 1978 OG1 THR A 127 28.831 -22.469 -21.330 1.00 16.76 O

ATOM 1979 CG2 THR A 127 30.938 -23.580 -20.990 1.00 16.57 C

ATOM 1980 H THR A 127 29.257 -22.459 -18.702 1.00 0.00 H

ATOM 1981 HA THR A 127 31.544 -21.018 -19.934 1.00 0.00 H

ATOM 1982 HB THR A 127 30.587 -21.762 -22.035 1.00 0.00 H

ATOM 1983 HG1 THR A 127 28.737 -23.080 -22.065 1.00 0.00 H

ATOM 1984 HG21 THR A 127 30.801 -24.188 -21.884 1.00 0.00 H

ATOM 1985 HG22 THR A 127 32.010 -23.434 -20.852 1.00 0.00 H

ATOM 1986 HG23 THR A 127 30.569 -24.157 -20.141 1.00 0.00 H

ATOM 1987 N ASN A 128 28.730 -19.585 -19.379 1.00 14.54 N

ATOM 1988 CA ASN A 128 27.909 -18.381 -19.570 1.00 13.69 C

ATOM 1989 C ASN A 128 28.707 -17.068 -19.332 1.00 13.26 C

ATOM 1990 O ASN A 128 29.718 -17.113 -18.626 1.00 13.31 O

ATOM 1991 CB ASN A 128 26.626 -18.483 -18.707 1.00 13.37 C

ATOM 1992 CG ASN A 128 26.868 -18.323 -17.206 1.00 12.91 C

ATOM 1993 OD1 ASN A 128 26.881 -17.206 -16.698 1.00 15.18 O

ATOM 1994 ND2 ASN A 128 27.053 -19.432 -16.495 1.00 10.17 N

ATOM 1995 H ASN A 128 28.526 -20.152 -18.570 1.00 0.00 H

ATOM 1996 HA ASN A 128 27.597 -18.410 -20.612 1.00 0.00 H

ATOM 1997 HB3 ASN A 128 26.113 -19.427 -18.900 1.00 0.00 H

ATOM 1998 HB2 ASN A 128 25.919 -17.707 -18.998 1.00 0.00 H

ATOM 1999 HD22 ASN A 128 27.210 -19.378 -15.496 1.00 0.00 H

ATOM 2000 HD21 ASN A 128 27.025 -20.341 -16.931 1.00 0.00 H

ATOM 2001 N PRO A 129 28.229 -15.918 -19.874 1.00 13.16 N

ATOM 2002 CA PRO A 129 28.895 -14.606 -19.703 1.00 12.79 C

ATOM 2003 C PRO A 129 29.109 -14.105 -18.261 1.00 12.18 C

ATOM 2004 O PRO A 129 30.036 -13.324 -18.045 1.00 12.81 O

ATOM 2005 CB PRO A 129 28.001 -13.625 -20.481 1.00 13.25 C

ATOM 2006 CG PRO A 129 27.294 -14.482 -21.511 1.00 13.84 C

ATOM 2007 CD PRO A 129 27.075 -15.793 -20.770 1.00 13.09 C

ATOM 2008 HA PRO A 129 29.866 -14.675 -20.198 1.00 0.00 H

ATOM 2009 HB3 PRO A 129 28.565 -12.811 -20.937 1.00 0.00 H

ATOM 2010 HB2 PRO A 129 27.253 -13.177 -19.822 1.00 0.00 H

ATOM 2011 HG3 PRO A 129 27.959 -14.651 -22.359 1.00 0.00 H

ATOM 2012 HG2 PRO A 129 26.372 -14.039 -21.887 1.00 0.00 H

ATOM 2013 HD2 PRO A 129 26.167 -15.742 -20.170 1.00 0.00 H

ATOM 2014 HD3 PRO A 129 26.966 -16.601 -21.491 1.00 0.00 H

ATOM 2015 N LYS A 130 28.256 -14.542 -17.319 1.00 11.28 N

ATOM 2016 CA LYS A 130 28.268 -14.101 -15.925 1.00 10.24 C

ATOM 2017 C LYS A 130 28.861 -15.132 -14.953 1.00 9.73 C

ATOM 2018 O LYS A 130 28.692 -14.942 -13.750 1.00 8.95 O

ATOM 2019 CB LYS A 130 26.848 -13.673 -15.495 1.00 10.14 C

ATOM 2020 CG LYS A 130 26.340 -12.444 -16.259 1.00 10.01 C

ATOM 2021 CD LYS A 130 25.074 -11.849 -15.625 1.00 9.91 C

ATOM 2022 CE LYS A 130 24.498 -10.662 -16.410 1.00 11.63 C

ATOM 2023 NZ LYS A 130 24.135 -11.038 -17.786 1.00 11.51 N1+

ATOM 2024 H LYS A 130 27.526 -15.197 -17.568 1.00 0.00 H

ATOM 2025 HA LYS A 130 28.906 -13.224 -15.831 1.00 0.00 H

ATOM 2026 HB3 LYS A 130 26.848 -13.414 -14.435 1.00 0.00 H

ATOM 2027 HB2 LYS A 130 26.147 -14.502 -15.603 1.00 0.00 H

ATOM 2028 HG3 LYS A 130 26.147 -12.731 -17.293 1.00 0.00 H

ATOM 2029 HG2 LYS A 130 27.118 -11.682 -16.295 1.00 0.00 H

ATOM 2030 HD3 LYS A 130 25.304 -11.518 -14.612 1.00 0.00 H

ATOM 2031 HD2 LYS A 130 24.316 -12.624 -15.517 1.00 0.00 H

ATOM 2032 HE3 LYS A 130 25.214 -9.844 -16.452 1.00 0.00 H

ATOM 2033 HE2 LYS A 130 23.606 -10.285 -15.911 1.00 0.00 H

ATOM 2034 HZ1 LYS A 130 23.497 -11.829 -17.756 1.00 0.00 H

ATOM 2035 HZ2 LYS A 130 23.694 -10.262 -18.258 1.00 0.00 H

ATOM 2036 HZ3 LYS A 130 24.963 -11.319 -18.292 1.00 0.00 H

ATOM 2037 N ARG A 131 29.555 -16.182 -15.438 1.00 9.65 N

ATOM 2038 CA ARG A 131 30.188 -17.186 -14.572 1.00 9.26 C

ATOM 2039 C ARG A 131 31.186 -16.543 -13.594 1.00 9.86 C

ATOM 2040 O ARG A 131 30.979 -16.642 -12.389 1.00 10.83 O

ATOM 2041 CB ARG A 131 30.781 -18.348 -15.400 1.00 9.24 C

ATOM 2042 CG ARG A 131 31.295 -19.553 -14.575 1.00 9.20 C

ATOM 2043 CD ARG A 131 32.735 -19.436 -14.046 1.00 8.39 C

ATOM 2044 NE ARG A 131 33.225 -20.709 -13.492 1.00 8.85 N

ATOM 2045 CZ ARG A 131 34.476 -20.933 -13.047 1.00 8.27 C

ATOM 2046 NH1 ARG A 131 35.420 -19.983 -13.082 1.00 8.94 N

ATOM 2047 NH2 ARG A 131 34.800 -22.130 -12.546 1.00 9.56 N1+

ATOM 2048 H ARG A 131 29.659 -16.304 -16.436 1.00 0.00 H

ATOM 2049 HA ARG A 131 29.385 -17.620 -13.975 1.00 0.00 H

ATOM 2050 HB3 ARG A 131 31.556 -17.996 -16.082 1.00 0.00 H

ATOM 2051 HB2 ARG A 131 29.979 -18.719 -16.041 1.00 0.00 H

ATOM 2052 HG3 ARG A 131 31.241 -20.452 -15.176 1.00 0.00 H

ATOM 2053 HG2 ARG A 131 30.604 -19.716 -13.747 1.00 0.00 H

ATOM 2054 HD3 ARG A 131 32.764 -18.748 -13.203 1.00 0.00 H

ATOM 2055 HD2 ARG A 131 33.400 -19.046 -14.816 1.00 0.00 H

ATOM 2056 HE ARG A 131 32.546 -21.452 -13.405 1.00 0.00 H

ATOM 2057 HH12 ARG A 131 36.351 -20.168 -12.742 1.00 0.00 H

ATOM 2058 HH11 ARG A 131 35.192 -19.039 -13.395 1.00 0.00 H

ATOM 2059 HH22 ARG A 131 35.736 -22.314 -12.219 1.00 0.00 H

ATOM 2060 HH21 ARG A 131 34.092 -22.851 -12.414 1.00 0.00 H

ATOM 2061 N ASP A 132 32.180 -15.815 -14.126 1.00 9.92 N

ATOM 2062 CA ASP A 132 33.188 -15.085 -13.347 1.00 10.58 C

ATOM 2063 C ASP A 132 32.731 -13.715 -12.806 1.00 10.02 C

ATOM 2064 O ASP A 132 33.589 -12.916 -12.438 1.00 10.12 O

ATOM 2065 CB ASP A 132 34.559 -15.017 -14.065 1.00 12.15 C

ATOM 2066 CG ASP A 132 35.130 -16.394 -14.412 1.00 14.58 C

ATOM 2067 OD1 ASP A 132 34.986 -17.303 -13.567 1.00 17.36 O

ATOM 2068 OD2 ASP A 132 35.780 -16.497 -15.474 1.00 18.83 O1-

ATOM 2069 H ASP A 132 32.265 -15.753 -15.130 1.00 0.00 H

ATOM 2070 HA ASP A 132 33.361 -15.662 -12.436 1.00 0.00 H

ATOM 2071 HB3 ASP A 132 35.305 -14.495 -13.464 1.00 0.00 H

ATOM 2072 HB2 ASP A 132 34.436 -14.450 -14.988 1.00 0.00 H

ATOM 2073 N TRP A 133 31.414 -13.478 -12.679 1.00 8.95 N

ATOM 2074 CA TRP A 133 30.865 -12.400 -11.842 1.00 8.02 C

ATOM 2075 C TRP A 133 30.792 -12.797 -10.358 1.00 8.11 C

ATOM 2076 O TRP A 133 30.445 -11.955 -9.533 1.00 7.76 O

ATOM 2077 CB TRP A 133 29.481 -11.956 -12.353 1.00 8.26 C

ATOM 2078 CG TRP A 133 29.452 -11.163 -13.625 1.00 8.25 C

ATOM 2079 CD1 TRP A 133 30.303 -11.273 -14.673 1.00 8.87 C

ATOM 2080 CD2 TRP A 133 28.487 -10.139 -14.006 1.00 8.89 C

ATOM 2081 NE1 TRP A 133 29.953 -10.372 -15.654 1.00 8.85 N

ATOM 2082 CE2 TRP A 133 28.840 -9.645 -15.299 1.00 8.85 C

ATOM 2083 CE3 TRP A 133 27.355 -9.564 -13.383 1.00 8.09 C

ATOM 2084 CZ2 TRP A 133 28.105 -8.639 -15.945 1.00 9.25 C

ATOM 2085 CZ3 TRP A 133 26.610 -8.554 -14.023 1.00 8.66 C

ATOM 2086 CH2 TRP A 133 26.980 -8.097 -15.302 1.00 8.42 C

ATOM 2087 H TRP A 133 30.748 -14.162 -13.012 1.00 0.00 H

ATOM 2088 HA TRP A 133 31.523 -11.528 -11.882 1.00 0.00 H

ATOM 2089 HB3 TRP A 133 29.006 -11.320 -11.605 1.00 0.00 H

ATOM 2090 HB2 TRP A 133 28.816 -12.810 -12.455 1.00 0.00 H

ATOM 2091 HD1 TRP A 133 31.133 -11.957 -14.733 1.00 0.00 H

ATOM 2092 HE1 TRP A 133 30.467 -10.282 -16.520 1.00 0.00 H

ATOM 2093 HE3 TRP A 133 27.058 -9.900 -12.401 1.00 0.00 H

ATOM 2094 HZ2 TRP A 133 28.398 -8.290 -16.924 1.00 0.00 H

ATOM 2095 HZ3 TRP A 133 25.753 -8.125 -13.530 1.00 0.00 H

ATOM 2096 HH2 TRP A 133 26.396 -7.333 -15.794 1.00 0.00 H

ATOM 2097 N PHE A 134 31.120 -14.061 -10.052 1.00 7.59 N

ATOM 2098 CA PHE A 134 31.180 -14.654 -8.722 1.00 7.22 C

ATOM 2099 C PHE A 134 32.403 -15.587 -8.685 1.00 6.97 C

ATOM 2100 O PHE A 134 32.922 -15.966 -9.738 1.00 7.60 O

ATOM 2101 CB PHE A 134 29.870 -15.427 -8.451 1.00 7.32 C

ATOM 2102 CG PHE A 134 28.599 -14.594 -8.537 1.00 7.33 C

ATOM 2103 CD1 PHE A 134 27.975 -14.376 -9.784 1.00 7.21 C

ATOM 2104 CD2 PHE A 134 28.126 -13.882 -7.416 1.00 7.88 C

ATOM 2105 CE1 PHE A 134 26.870 -13.545 -9.883 1.00 8.10 C

ATOM 2106 CE2 PHE A 134 27.008 -13.070 -7.527 1.00 8.10 C

ATOM 2107 CZ PHE A 134 26.378 -12.907 -8.754 1.00 8.18 C

ATOM 2108 H PHE A 134 31.396 -14.683 -10.799 1.00 0.00 H

ATOM 2109 HA PHE A 134 31.318 -13.874 -7.972 1.00 0.00 H

ATOM 2110 HB3 PHE A 134 29.915 -15.878 -7.460 1.00 0.00 H

ATOM 2111 HB2 PHE A 134 29.779 -16.257 -9.153 1.00 0.00 H

ATOM 2112 HD1 PHE A 134 28.376 -14.832 -10.674 1.00 0.00 H

ATOM 2113 HD2 PHE A 134 28.622 -13.967 -6.464 1.00 0.00 H

ATOM 2114 HE1 PHE A 134 26.402 -13.387 -10.843 1.00 0.00 H

ATOM 2115 HE2 PHE A 134 26.644 -12.548 -6.654 1.00 0.00 H

ATOM 2116 HZ PHE A 134 25.515 -12.264 -8.839 1.00 0.00 H

ATOM 2117 N PHE A 135 32.859 -15.938 -7.473 1.00 6.70 N

ATOM 2118 CA PHE A 135 34.027 -16.800 -7.275 1.00 7.40 C

ATOM 2119 C PHE A 135 33.607 -18.275 -7.330 1.00 7.88 C

ATOM 2120 O PHE A 135 33.059 -18.766 -6.347 1.00 7.41 O

ATOM 2121 CB PHE A 135 34.679 -16.496 -5.913 1.00 7.78 C

ATOM 2122 CG PHE A 135 35.159 -15.075 -5.713 1.00 6.68 C

ATOM 2123 CD1 PHE A 135 36.434 -14.687 -6.171 1.00 8.59 C

ATOM 2124 CD2 PHE A 135 34.280 -14.097 -5.202 1.00 7.47 C

ATOM 2125 CE1 PHE A 135 36.837 -13.364 -6.048 1.00 7.06 C

ATOM 2126 CE2 PHE A 135 34.700 -12.780 -5.093 1.00 7.75 C

ATOM 2127 CZ PHE A 135 35.974 -12.418 -5.510 1.00 7.64 C

ATOM 2128 H PHE A 135 32.402 -15.596 -6.640 1.00 0.00 H

ATOM 2129 HA PHE A 135 34.769 -16.608 -8.053 1.00 0.00 H

ATOM 2130 HB3 PHE A 135 35.525 -17.167 -5.752 1.00 0.00 H

ATOM 2131 HB2 PHE A 135 33.971 -16.716 -5.123 1.00 0.00 H

ATOM 2132 HD1 PHE A 135 37.102 -15.414 -6.608 1.00 0.00 H

ATOM 2133 HD2 PHE A 135 33.283 -14.370 -4.888 1.00 0.00 H

ATOM 2134 HE1 PHE A 135 37.817 -13.066 -6.389 1.00 0.00 H

ATOM 2135 HE2 PHE A 135 34.030 -12.036 -4.691 1.00 0.00 H

ATOM 2136 HZ PHE A 135 36.292 -11.388 -5.436 1.00 0.00 H

ATOM 2137 N TRP A 136 33.888 -18.946 -8.457 1.00 8.19 N

ATOM 2138 CA TRP A 136 33.678 -20.383 -8.628 1.00 8.69 C

ATOM 2139 C TRP A 136 35.021 -21.112 -8.628 1.00 8.89 C

ATOM 2140 O TRP A 136 35.935 -20.709 -9.349 1.00 9.79 O

ATOM 2141 CB TRP A 136 32.944 -20.666 -9.942 1.00 8.55 C

ATOM 2142 CG TRP A 136 31.531 -20.193 -10.001 1.00 8.67 C

ATOM 2143 CD1 TRP A 136 31.134 -18.984 -10.442 1.00 9.08 C

ATOM 2144 CD2 TRP A 136 30.326 -20.868 -9.541 1.00 9.27 C

ATOM 2145 NE1 TRP A 136 29.761 -18.893 -10.369 1.00 8.85 N

ATOM 2146 CE2 TRP A 136 29.207 -20.027 -9.816 1.00 9.35 C

ATOM 2147 CE3 TRP A 136 30.063 -22.112 -8.927 1.00 8.85 C

ATOM 2148 CZ2 TRP A 136 27.889 -20.414 -9.525 1.00 8.91 C

ATOM 2149 CZ3 TRP A 136 28.748 -22.501 -8.616 1.00 9.38 C

ATOM 2150 CH2 TRP A 136 27.659 -21.661 -8.918 1.00 9.36 C

ATOM 2151 H TRP A 136 34.330 -18.467 -9.230 1.00 0.00 H

ATOM 2152 HA TRP A 136 33.064 -20.777 -7.820 1.00 0.00 H

ATOM 2153 HB3 TRP A 136 32.922 -21.740 -10.126 1.00 0.00 H

ATOM 2154 HB2 TRP A 136 33.491 -20.224 -10.776 1.00 0.00 H

ATOM 2155 HD1 TRP A 136 31.820 -18.237 -10.807 1.00 0.00 H

ATOM 2156 HE1 TRP A 136 29.244 -18.086 -10.703 1.00 0.00 H

ATOM 2157 HE3 TRP A 136 30.884 -22.771 -8.690 1.00 0.00 H

ATOM 2158 HZ2 TRP A 136 27.067 -19.755 -9.762 1.00 0.00 H

ATOM 2159 HZ3 TRP A 136 28.589 -23.452 -8.139 1.00 0.00 H

ATOM 2160 HH2 TRP A 136 26.655 -21.975 -8.676 1.00 0.00 H

ATOM 2161 N ARG A 137 35.094 -22.191 -7.840 1.00 8.80 N

ATOM 2162 CA ARG A 137 36.254 -23.069 -7.726 1.00 9.24 C

ATOM 2163 C ARG A 137 35.748 -24.508 -7.533 1.00 9.74 C

ATOM 2164 O ARG A 137 34.784 -24.701 -6.790 1.00 9.38 O

ATOM 2165 CB ARG A 137 37.098 -22.638 -6.505 1.00 9.92 C

ATOM 2166 CG ARG A 137 37.938 -21.365 -6.721 1.00 10.62 C

ATOM 2167 CD ARG A 137 38.872 -21.039 -5.543 1.00 13.09 C

ATOM 2168 NE ARG A 137 39.972 -22.006 -5.411 1.00 14.12 N

ATOM 2169 CZ ARG A 137 40.742 -22.177 -4.321 1.00 14.28 C

ATOM 2170 NH1 ARG A 137 40.595 -21.414 -3.228 1.00 15.48 N

ATOM 2171 NH2 ARG A 137 41.677 -23.134 -4.322 1.00 15.43 N1+

ATOM 2172 H ARG A 137 34.287 -22.462 -7.292 1.00 0.00 H

ATOM 2173 HA ARG A 137 36.842 -22.980 -8.639 1.00 0.00 H

ATOM 2174 HB3 ARG A 137 37.775 -23.451 -6.239 1.00 0.00 H

ATOM 2175 HB2 ARG A 137 36.441 -22.495 -5.649 1.00 0.00 H

ATOM 2176 HG3 ARG A 137 37.332 -20.494 -6.969 1.00 0.00 H

ATOM 2177 HG2 ARG A 137 38.561 -21.557 -7.595 1.00 0.00 H

ATOM 2178 HD3 ARG A 137 38.291 -21.182 -4.632 1.00 0.00 H

ATOM 2179 HD2 ARG A 137 39.204 -20.002 -5.538 1.00 0.00 H

ATOM 2180 HE ARG A 137 40.164 -22.568 -6.227 1.00 0.00 H

ATOM 2181 HH12 ARG A 137 41.197 -21.545 -2.426 1.00 0.00 H

ATOM 2182 HH11 ARG A 137 39.900 -20.683 -3.211 1.00 0.00 H

ATOM 2183 HH22 ARG A 137 42.245 -23.291 -3.500 1.00 0.00 H

ATOM 2184 HH21 ARG A 137 41.811 -23.723 -5.131 1.00 0.00 H

ATOM 2185 N PRO A 138 36.420 -25.506 -8.153 1.00 10.57 N

ATOM 2186 CA PRO A 138 36.100 -26.922 -7.904 1.00 10.92 C

ATOM 2187 C PRO A 138 36.449 -27.364 -6.463 1.00 10.92 C

ATOM 2188 O PRO A 138 37.201 -26.657 -5.786 1.00 10.53 O

ATOM 2189 CB PRO A 138 36.950 -27.658 -8.954 1.00 11.22 C

ATOM 2190 CG PRO A 138 38.166 -26.767 -9.154 1.00 12.00 C

ATOM 2191 CD PRO A 138 37.582 -25.364 -9.035 1.00 10.60 C

ATOM 2192 HA PRO A 138 35.042 -27.084 -8.091 1.00 0.00 H

ATOM 2193 HB3 PRO A 138 36.387 -27.726 -9.886 1.00 0.00 H

ATOM 2194 HB2 PRO A 138 37.228 -28.674 -8.668 1.00 0.00 H

ATOM 2195 HG3 PRO A 138 38.683 -26.943 -10.098 1.00 0.00 H

ATOM 2196 HG2 PRO A 138 38.875 -26.939 -8.344 1.00 0.00 H

ATOM 2197 HD2 PRO A 138 38.332 -24.671 -8.653 1.00 0.00 H

ATOM 2198 HD3 PRO A 138 37.239 -25.007 -10.007 1.00 0.00 H

ATOM 2199 N PRO A 139 35.946 -28.545 -6.032 1.00 11.49 N

ATOM 2200 CA PRO A 139 36.390 -29.180 -4.778 1.00 11.93 C

ATOM 2201 C PRO A 139 37.909 -29.397 -4.722 1.00 12.64 C

ATOM 2202 O PRO A 139 38.493 -29.855 -5.704 1.00 13.36 O

ATOM 2203 CB PRO A 139 35.633 -30.522 -4.749 1.00 11.95 C

ATOM 2204 CG PRO A 139 34.422 -30.305 -5.634 1.00 11.88 C

ATOM 2205 CD PRO A 139 34.942 -29.365 -6.712 1.00 11.44 C

ATOM 2206 HA PRO A 139 36.066 -28.542 -3.955 1.00 0.00 H

ATOM 2207 HB3 PRO A 139 35.353 -30.821 -3.738 1.00 0.00 H

ATOM 2208 HB2 PRO A 139 36.238 -31.328 -5.168 1.00 0.00 H

ATOM 2209 HG3 PRO A 139 33.648 -29.797 -5.059 1.00 0.00 H

ATOM 2210 HG2 PRO A 139 33.994 -31.227 -6.027 1.00 0.00 H

ATOM 2211 HD2 PRO A 139 35.421 -29.926 -7.516 1.00 0.00 H

ATOM 2212 HD3 PRO A 139 34.119 -28.789 -7.130 1.00 0.00 H

ATOM 2213 N LYS A 140 38.507 -29.075 -3.564 1.00 12.90 N

ATOM 2214 CA LYS A 140 39.904 -29.384 -3.241 1.00 13.72 C

ATOM 2215 C LYS A 140 40.191 -30.902 -3.251 1.00 13.70 C

ATOM 2216 O LYS A 140 41.307 -31.312 -3.568 1.00 14.90 O

ATOM 2217 CB LYS A 140 40.239 -28.714 -1.893 1.00 14.08 C

ATOM 2218 CG LYS A 140 41.647 -28.993 -1.340 1.00 17.13 C

ATOM 2219 CD LYS A 140 41.971 -28.120 -0.114 1.00 21.98 C

ATOM 2220 CE LYS A 140 42.768 -28.857 0.971 1.00 24.27 C

ATOM 2221 NZ LYS A 140 41.941 -29.878 1.639 1.00 27.68 N1+

ATOM 2222 H LYS A 140 37.948 -28.704 -2.808 1.00 0.00 H

ATOM 2223 HA LYS A 140 40.528 -28.928 -4.012 1.00 0.00 H

ATOM 2224 HB3 LYS A 140 39.507 -29.024 -1.145 1.00 0.00 H

ATOM 2225 HB2 LYS A 140 40.117 -27.637 -2.007 1.00 0.00 H

ATOM 2226 HG3 LYS A 140 42.392 -28.817 -2.116 1.00 0.00 H

ATOM 2227 HG2 LYS A 140 41.730 -30.048 -1.079 1.00 0.00 H

ATOM 2228 HD3 LYS A 140 41.058 -27.709 0.319 1.00 0.00 H

ATOM 2229 HD2 LYS A 140 42.549 -27.255 -0.443 1.00 0.00 H

ATOM 2230 HE3 LYS A 140 43.105 -28.146 1.726 1.00 0.00 H

ATOM 2231 HE2 LYS A 140 43.658 -29.322 0.547 1.00 0.00 H

ATOM 2232 HZ1 LYS A 140 41.636 -30.560 0.960 1.00 0.00 H

ATOM 2233 HZ2 LYS A 140 42.483 -30.335 2.358 1.00 0.00 H

ATOM 2234 HZ3 LYS A 140 41.134 -29.434 2.055 1.00 0.00 H

ATOM 2235 N GLY A 141 39.151 -31.691 -2.955 1.00 13.20 N

ATOM 2236 CA GLY A 141 39.136 -33.138 -3.047 1.00 12.07 C

ATOM 2237 C GLY A 141 37.765 -33.593 -2.542 1.00 11.00 C

ATOM 2238 O GLY A 141 36.803 -32.822 -2.515 1.00 10.51 O

ATOM 2239 H GLY A 141 38.267 -31.260 -2.724 1.00 0.00 H

ATOM 2240 HA3 GLY A 141 39.932 -33.566 -2.437 1.00 0.00 H

ATOM 2241 HA2 GLY A 141 39.277 -33.455 -4.080 1.00 0.00 H

ATOM 2242 N TYR A 142 37.695 -34.866 -2.138 1.00 10.26 N

ATOM 2243 CA TYR A 142 36.550 -35.468 -1.459 1.00 9.78 C

ATOM 2244 C TYR A 142 37.082 -36.163 -0.202 1.00 9.47 C

ATOM 2245 O TYR A 142 38.155 -36.767 -0.257 1.00 10.05 O

ATOM 2246 CB TYR A 142 35.832 -36.462 -2.398 1.00 10.27 C

ATOM 2247 CG TYR A 142 35.198 -35.820 -3.622 1.00 10.38 C

ATOM 2248 CD1 TYR A 142 35.964 -35.592 -4.785 1.00 11.19 C

ATOM 2249 CD2 TYR A 142 33.844 -35.428 -3.596 1.00 9.84 C

ATOM 2250 CE1 TYR A 142 35.392 -34.946 -5.897 1.00 11.79 C

ATOM 2251 CE2 TYR A 142 33.267 -34.791 -4.711 1.00 10.44 C

ATOM 2252 CZ TYR A 142 34.043 -34.541 -5.859 1.00 11.53 C

ATOM 2253 OH TYR A 142 33.487 -33.907 -6.932 1.00 14.08 O

ATOM 2254 H TYR A 142 38.516 -35.452 -2.191 1.00 0.00 H

ATOM 2255 HA TYR A 142 35.846 -34.695 -1.152 1.00 0.00 H

ATOM 2256 HB3 TYR A 142 35.046 -36.978 -1.844 1.00 0.00 H

ATOM 2257 HB2 TYR A 142 36.524 -37.239 -2.726 1.00 0.00 H

ATOM 2258 HD1 TYR A 142 37.002 -35.890 -4.817 1.00 0.00 H

ATOM 2259 HD2 TYR A 142 33.246 -35.601 -2.715 1.00 0.00 H

ATOM 2260 HE1 TYR A 142 35.992 -34.760 -6.776 1.00 0.00 H

ATOM 2261 HE2 TYR A 142 32.231 -34.487 -4.679 1.00 0.00 H

ATOM 2262 HH TYR A 142 34.099 -33.795 -7.663 1.00 0.00 H

ATOM 2263 N ASP A 143 36.335 -36.057 0.910 1.00 9.22 N

ATOM 2264 CA ASP A 143 36.708 -36.652 2.201 1.00 9.81 C

ATOM 2265 C ASP A 143 36.469 -38.178 2.209 1.00 9.82 C

ATOM 2266 O ASP A 143 36.016 -38.742 1.210 1.00 9.73 O

ATOM 2267 CB ASP A 143 36.058 -35.929 3.416 1.00 9.73 C

ATOM 2268 CG ASP A 143 34.534 -35.762 3.388 1.00 11.21 C

ATOM 2269 OD1 ASP A 143 33.853 -36.665 2.855 1.00 10.51 O

ATOM 2270 OD2 ASP A 143 34.067 -34.766 3.982 1.00 13.37 O1-

ATOM 2271 H ASP A 143 35.450 -35.566 0.882 1.00 0.00 H

ATOM 2272 HA ASP A 143 37.785 -36.524 2.318 1.00 0.00 H

ATOM 2273 HB3 ASP A 143 36.488 -34.927 3.451 1.00 0.00 H

ATOM 2274 HB2 ASP A 143 36.332 -36.407 4.357 1.00 0.00 H

ATOM 2275 N ALA A 144 36.764 -38.821 3.350 1.00 10.35 N

ATOM 2276 CA ALA A 144 36.548 -40.253 3.586 1.00 10.85 C

ATOM 2277 C ALA A 144 35.078 -40.709 3.475 1.00 11.05 C

ATOM 2278 O ALA A 144 34.843 -41.884 3.195 1.00 11.46 O

ATOM 2279 CB ALA A 144 37.125 -40.614 4.963 1.00 11.01 C

ATOM 2280 H ALA A 144 37.118 -38.295 4.136 1.00 0.00 H

ATOM 2281 HA ALA A 144 37.113 -40.798 2.829 1.00 0.00 H

ATOM 2282 HB1 ALA A 144 36.995 -41.674 5.183 1.00 0.00 H

ATOM 2283 HB2 ALA A 144 38.193 -40.400 5.009 1.00 0.00 H

ATOM 2284 HB3 ALA A 144 36.638 -40.049 5.759 1.00 0.00 H

ATOM 2285 N GLU A 145 34.130 -39.776 3.667 1.00 11.24 N

ATOM 2286 CA GLU A 145 32.685 -39.986 3.561 1.00 11.70 C

ATOM 2287 C GLU A 145 32.149 -39.716 2.137 1.00 11.62 C

ATOM 2288 O GLU A 145 30.973 -39.984 1.890 1.00 12.00 O

ATOM 2289 CB GLU A 145 31.944 -39.114 4.607 1.00 12.42 C

ATOM 2290 CG GLU A 145 32.212 -39.460 6.092 1.00 14.01 C

ATOM 2291 CD GLU A 145 33.612 -39.104 6.606 1.00 17.71 C

ATOM 2292 OE1 GLU A 145 34.223 -39.982 7.252 1.00 19.39 O

ATOM 2293 OE2 GLU A 145 34.052 -37.965 6.336 1.00 19.24 O1-

ATOM 2294 H GLU A 145 34.411 -38.832 3.900 1.00 0.00 H

ATOM 2295 HA GLU A 145 32.460 -41.031 3.784 1.00 0.00 H

ATOM 2296 HB3 GLU A 145 30.869 -39.201 4.444 1.00 0.00 H

ATOM 2297 HB2 GLU A 145 32.170 -38.061 4.433 1.00 0.00 H

ATOM 2298 HG3 GLU A 145 32.024 -40.522 6.261 1.00 0.00 H

ATOM 2299 HG2 GLU A 145 31.498 -38.923 6.717 1.00 0.00 H

ATOM 2300 N GLY A 146 33.003 -39.212 1.229 1.00 11.10 N

ATOM 2301 CA GLY A 146 32.686 -38.966 -0.178 1.00 10.99 C

ATOM 2302 C GLY A 146 32.062 -37.583 -0.426 1.00 11.19 C

ATOM 2303 O GLY A 146 31.620 -37.329 -1.546 1.00 11.70 O

ATOM 2304 H GLY A 146 33.950 -39.001 1.520 1.00 0.00 H

ATOM 2305 HA3 GLY A 146 32.022 -39.741 -0.564 1.00 0.00 H

ATOM 2306 HA2 GLY A 146 33.612 -39.032 -0.750 1.00 0.00 H

ATOM 2307 N LYS A 147 32.020 -36.696 0.583 1.00 10.78 N

ATOM 2308 CA LYS A 147 31.523 -35.324 0.460 1.00 11.13 C

ATOM 2309 C LYS A 147 32.639 -34.416 -0.088 1.00 10.50 C

ATOM 2310 O LYS A 147 33.790 -34.588 0.316 1.00 10.61 O

ATOM 2311 CB LYS A 147 31.077 -34.821 1.846 1.00 11.54 C

ATOM 2312 CG LYS A 147 29.862 -35.575 2.398 1.00 14.53 C

ATOM 2313 CD LYS A 147 29.512 -35.154 3.833 1.00 19.54 C

ATOM 2314 CE LYS A 147 28.238 -35.829 4.360 1.00 21.72 C

ATOM 2315 NZ LYS A 147 28.355 -37.298 4.359 1.00 25.21 N1+

ATOM 2316 H LYS A 147 32.452 -36.930 1.470 1.00 0.00 H

ATOM 2317 HA LYS A 147 30.656 -35.345 -0.200 1.00 0.00 H

ATOM 2318 HB3 LYS A 147 30.823 -33.761 1.784 1.00 0.00 H

ATOM 2319 HB2 LYS A 147 31.900 -34.888 2.553 1.00 0.00 H

ATOM 2320 HG3 LYS A 147 30.060 -36.647 2.374 1.00 0.00 H

ATOM 2321 HG2 LYS A 147 29.006 -35.405 1.744 1.00 0.00 H

ATOM 2322 HD3 LYS A 147 29.388 -34.071 3.874 1.00 0.00 H

ATOM 2323 HD2 LYS A 147 30.349 -35.386 4.493 1.00 0.00 H

ATOM 2324 HE3 LYS A 147 27.380 -35.544 3.749 1.00 0.00 H

ATOM 2325 HE2 LYS A 147 28.031 -35.494 5.377 1.00 0.00 H

ATOM 2326 HZ1 LYS A 147 29.128 -37.576 4.948 1.00 0.00 H

ATOM 2327 HZ2 LYS A 147 27.501 -37.709 4.709 1.00 0.00 H

ATOM 2328 HZ3 LYS A 147 28.522 -37.623 3.417 1.00 0.00 H

ATOM 2329 N PRO A 148 32.297 -33.464 -0.988 1.00 10.22 N

ATOM 2330 CA PRO A 148 33.282 -32.521 -1.540 1.00 10.04 C

ATOM 2331 C PRO A 148 33.861 -31.593 -0.455 1.00 9.92 C

ATOM 2332 O PRO A 148 33.110 -31.050 0.356 1.00 10.79 O

ATOM 2333 CB PRO A 148 32.487 -31.771 -2.621 1.00 10.10 C

ATOM 2334 CG PRO A 148 31.045 -31.813 -2.150 1.00 10.42 C

ATOM 2335 CD PRO A 148 30.947 -33.178 -1.483 1.00 10.55 C

ATOM 2336 HA PRO A 148 34.099 -33.068 -2.012 1.00 0.00 H

ATOM 2337 HB3 PRO A 148 32.576 -32.302 -3.569 1.00 0.00 H

ATOM 2338 HB2 PRO A 148 32.832 -30.758 -2.799 1.00 0.00 H

ATOM 2339 HG3 PRO A 148 30.323 -31.673 -2.954 1.00 0.00 H

ATOM 2340 HG2 PRO A 148 30.881 -31.028 -1.410 1.00 0.00 H

ATOM 2341 HD2 PRO A 148 30.193 -33.166 -0.694 1.00 0.00 H

ATOM 2342 HD3 PRO A 148 30.675 -33.942 -2.213 1.00 0.00 H

ATOM 2343 N ILE A 149 35.195 -31.473 -0.444 1.00 9.78 N

ATOM 2344 CA ILE A 149 35.944 -30.608 0.467 1.00 10.07 C

ATOM 2345 C ILE A 149 36.044 -29.213 -0.190 1.00 10.34 C

ATOM 2346 O ILE A 149 36.399 -29.155 -1.370 1.00 10.47 O

ATOM 2347 CB ILE A 149 37.390 -31.156 0.667 1.00 10.48 C

ATOM 2348 CG1 ILE A 149 37.367 -32.570 1.295 1.00 10.25 C

ATOM 2349 CG2 ILE A 149 38.290 -30.228 1.516 1.00 10.97 C

ATOM 2350 CD1 ILE A 149 38.738 -33.263 1.315 1.00 11.86 C

ATOM 2351 H ILE A 149 35.744 -31.963 -1.139 1.00 0.00 H

ATOM 2352 HA ILE A 149 35.433 -30.587 1.429 1.00 0.00 H

ATOM 2353 HB ILE A 149 37.856 -31.244 -0.316 1.00 0.00 H

ATOM 2354 HG13 ILE A 149 36.676 -33.215 0.755 1.00 0.00 H

ATOM 2355 HG12 ILE A 149 36.978 -32.512 2.312 1.00 0.00 H

ATOM 2356 HG21 ILE A 149 39.291 -30.638 1.623 1.00 0.00 H

ATOM 2357 HG22 ILE A 149 38.418 -29.242 1.075 1.00 0.00 H

ATOM 2358 HG23 ILE A 149 37.884 -30.094 2.519 1.00 0.00 H

ATOM 2359 HD11 ILE A 149 38.630 -34.337 1.456 1.00 0.00 H

ATOM 2360 HD12 ILE A 149 39.280 -33.106 0.382 1.00 0.00 H

ATOM 2361 HD13 ILE A 149 39.356 -32.897 2.135 1.00 0.00 H

ATOM 2362 N PRO A 150 35.724 -28.120 0.547 1.00 10.38 N

ATOM 2363 CA PRO A 150 35.757 -26.762 -0.026 1.00 10.52 C

ATOM 2364 C PRO A 150 37.171 -26.310 -0.466 1.00 10.22 C

ATOM 2365 O PRO A 150 38.156 -26.871 0.016 1.00 10.53 O

ATOM 2366 CB PRO A 150 35.170 -25.878 1.089 1.00 9.58 C

ATOM 2367 CG PRO A 150 35.464 -26.633 2.373 1.00 10.70 C

ATOM 2368 CD PRO A 150 35.320 -28.091 1.954 1.00 10.50 C

ATOM 2369 HA PRO A 150 35.086 -26.773 -0.879 1.00 0.00 H

ATOM 2370 HB3 PRO A 150 34.091 -25.798 0.950 1.00 0.00 H

ATOM 2371 HB2 PRO A 150 35.571 -24.864 1.106 1.00 0.00 H

ATOM 2372 HG3 PRO A 150 34.812 -26.353 3.201 1.00 0.00 H

ATOM 2373 HG2 PRO A 150 36.494 -26.440 2.676 1.00 0.00 H

ATOM 2374 HD2 PRO A 150 35.926 -28.733 2.593 1.00 0.00 H

ATOM 2375 HD3 PRO A 150 34.280 -28.414 2.023 1.00 0.00 H

ATOM 2376 N PRO A 151 37.253 -25.303 -1.369 1.00 10.12 N

ATOM 2377 CA PRO A 151 38.526 -24.835 -1.954 1.00 10.02 C

ATOM 2378 C PRO A 151 39.573 -24.309 -0.955 1.00 9.90 C

ATOM 2379 O PRO A 151 40.764 -24.383 -1.254 1.00 11.09 O

ATOM 2380 CB PRO A 151 38.106 -23.737 -2.945 1.00 10.50 C

ATOM 2381 CG PRO A 151 36.641 -23.996 -3.230 1.00 10.79 C

ATOM 2382 CD PRO A 151 36.134 -24.542 -1.913 1.00 10.30 C

ATOM 2383 HA PRO A 151 38.951 -25.671 -2.512 1.00 0.00 H

ATOM 2384 HB3 PRO A 151 38.702 -23.760 -3.855 1.00 0.00 H

ATOM 2385 HB2 PRO A 151 38.208 -22.747 -2.498 1.00 0.00 H

ATOM 2386 HG3 PRO A 151 36.542 -24.759 -4.003 1.00 0.00 H

ATOM 2387 HG2 PRO A 151 36.098 -23.112 -3.553 1.00 0.00 H

ATOM 2388 HD2 PRO A 151 35.927 -23.738 -1.214 1.00 0.00 H

ATOM 2389 HD3 PRO A 151 35.215 -25.094 -2.075 1.00 0.00 H

ATOM 2390 N ASN A 152 39.108 -23.808 0.202 1.00 8.93 N

ATOM 2391 CA ASN A 152 39.949 -23.354 1.305 1.00 8.32 C

ATOM 2392 C ASN A 152 39.224 -23.511 2.658 1.00 8.09 C

ATOM 2393 O ASN A 152 38.057 -23.908 2.704 1.00 7.93 O

ATOM 2394 CB ASN A 152 40.496 -21.921 1.030 1.00 7.79 C

ATOM 2395 CG ASN A 152 39.440 -20.811 0.944 1.00 7.88 C

ATOM 2396 OD1 ASN A 152 38.431 -20.837 1.639 1.00 7.83 O

ATOM 2397 ND2 ASN A 152 39.676 -19.798 0.114 1.00 7.20 N

ATOM 2398 H ASN A 152 38.114 -23.790 0.378 1.00 0.00 H

ATOM 2399 HA ASN A 152 40.795 -24.039 1.350 1.00 0.00 H

ATOM 2400 HB3 ASN A 152 41.079 -21.927 0.108 1.00 0.00 H

ATOM 2401 HB2 ASN A 152 41.198 -21.634 1.812 1.00 0.00 H

ATOM 2402 HD22 ASN A 152 39.027 -19.026 0.063 1.00 0.00 H

ATOM 2403 HD21 ASN A 152 40.545 -19.750 -0.405 1.00 0.00 H

ATOM 2404 N ASN A 153 39.950 -23.168 3.731 1.00 8.02 N

ATOM 2405 CA ASN A 153 39.516 -23.235 5.128 1.00 7.87 C

ATOM 2406 C ASN A 153 38.733 -21.998 5.626 1.00 7.43 C

ATOM 2407 O ASN A 153 38.594 -21.862 6.839 1.00 7.28 O

ATOM 2408 CB ASN A 153 40.753 -23.537 6.023 1.00 8.05 C

ATOM 2409 CG ASN A 153 41.781 -22.401 6.172 1.00 8.82 C

ATOM 2410 OD1 ASN A 153 41.674 -21.349 5.546 1.00 7.73 O

ATOM 2411 ND2 ASN A 153 42.793 -22.613 7.014 1.00 9.67 N

ATOM 2412 H ASN A 153 40.912 -22.887 3.589 1.00 0.00 H

ATOM 2413 HA ASN A 153 38.822 -24.069 5.247 1.00 0.00 H

ATOM 2414 HB3 ASN A 153 41.271 -24.417 5.639 1.00 0.00 H

ATOM 2415 HB2 ASN A 153 40.413 -23.807 7.024 1.00 0.00 H

ATOM 2416 HD22 ASN A 153 43.498 -21.898 7.144 1.00 0.00 H

ATOM 2417 HD21 ASN A 153 42.871 -23.484 7.516 1.00 0.00 H

ATOM 2418 N TRP A 154 38.280 -21.090 4.744 1.00 7.65 N

ATOM 2419 CA TRP A 154 37.666 -19.823 5.160 1.00 7.33 C

ATOM 2420 C TRP A 154 36.329 -19.992 5.891 1.00 7.52 C

ATOM 2421 O TRP A 154 35.491 -20.790 5.471 1.00 7.04 O

ATOM 2422 CB TRP A 154 37.512 -18.858 3.974 1.00 7.28 C

ATOM 2423 CG TRP A 154 38.772 -18.293 3.387 1.00 7.32 C

ATOM 2424 CD1 TRP A 154 40.040 -18.559 3.780 1.00 7.83 C

ATOM 2425 CD2 TRP A 154 38.889 -17.325 2.301 1.00 7.23 C

ATOM 2426 NE1 TRP A 154 40.922 -17.830 3.016 1.00 7.82 N

ATOM 2427 CE2 TRP A 154 40.273 -17.049 2.087 1.00 7.14 C

ATOM 2428 CE3 TRP A 154 37.964 -16.641 1.479 1.00 6.82 C

ATOM 2429 CZ2 TRP A 154 40.719 -16.150 1.104 1.00 7.50 C

ATOM 2430 CZ3 TRP A 154 38.398 -15.732 0.492 1.00 7.30 C

ATOM 2431 CH2 TRP A 154 39.772 -15.484 0.306 1.00 7.78 C

ATOM 2432 H TRP A 154 38.414 -21.228 3.751 1.00 0.00 H

ATOM 2433 HA TRP A 154 38.351 -19.352 5.865 1.00 0.00 H

ATOM 2434 HB3 TRP A 154 36.910 -18.001 4.280 1.00 0.00 H

ATOM 2435 HB2 TRP A 154 36.947 -19.339 3.175 1.00 0.00 H

ATOM 2436 HD1 TRP A 154 40.317 -19.241 4.569 1.00 0.00 H

ATOM 2437 HE1 TRP A 154 41.928 -17.896 3.119 1.00 0.00 H

ATOM 2438 HE3 TRP A 154 36.907 -16.813 1.620 1.00 0.00 H

ATOM 2439 HZ2 TRP A 154 41.775 -15.966 0.971 1.00 0.00 H

ATOM 2440 HZ3 TRP A 154 37.675 -15.214 -0.119 1.00 0.00 H

ATOM 2441 HH2 TRP A 154 40.099 -14.778 -0.442 1.00 0.00 H

ATOM 2442 N LYS A 155 36.179 -19.209 6.970 1.00 7.68 N

ATOM 2443 CA LYS A 155 34.997 -19.152 7.815 1.00 9.31 C

ATOM 2444 C LYS A 155 34.049 -18.073 7.286 1.00 8.73 C

ATOM 2445 O LYS A 155 34.489 -16.967 6.969 1.00 8.75 O

ATOM 2446 CB LYS A 155 35.410 -18.850 9.277 1.00 9.29 C

ATOM 2447 CG LYS A 155 34.311 -19.162 10.314 1.00 12.33 C

ATOM 2448 CD LYS A 155 34.605 -18.661 11.740 1.00 13.69 C

ATOM 2449 CE LYS A 155 35.794 -19.368 12.409 1.00 19.42 C

ATOM 2450 NZ LYS A 155 35.912 -19.020 13.838 1.00 22.39 N1+

ATOM 2451 H LYS A 155 36.942 -18.603 7.245 1.00 0.00 H

ATOM 2452 HA LYS A 155 34.506 -20.121 7.783 1.00 0.00 H

ATOM 2453 HB3 LYS A 155 35.731 -17.810 9.365 1.00 0.00 H

ATOM 2454 HB2 LYS A 155 36.280 -19.446 9.538 1.00 0.00 H

ATOM 2455 HG3 LYS A 155 34.126 -20.234 10.344 1.00 0.00 H

ATOM 2456 HG2 LYS A 155 33.367 -18.743 9.992 1.00 0.00 H

ATOM 2457 HD3 LYS A 155 33.710 -18.798 12.347 1.00 0.00 H

ATOM 2458 HD2 LYS A 155 34.787 -17.586 11.713 1.00 0.00 H

ATOM 2459 HE3 LYS A 155 36.727 -19.116 11.904 1.00 0.00 H

ATOM 2460 HE2 LYS A 155 35.663 -20.446 12.330 1.00 0.00 H

ATOM 2461 HZ1 LYS A 155 36.688 -19.523 14.250 1.00 0.00 H

ATOM 2462 HZ2 LYS A 155 36.070 -18.028 13.941 1.00 0.00 H

ATOM 2463 HZ3 LYS A 155 35.065 -19.278 14.322 1.00 0.00 H

ATOM 2464 N SER A 156 32.754 -18.406 7.256 1.00 8.01 N

ATOM 2465 CA SER A 156 31.667 -17.449 7.109 1.00 7.44 C

ATOM 2466 C SER A 156 31.566 -16.561 8.366 1.00 7.56 C

ATOM 2467 O SER A 156 31.790 -17.044 9.475 1.00 7.82 O

ATOM 2468 CB SER A 156 30.369 -18.242 6.845 1.00 7.32 C

ATOM 2469 OG SER A 156 29.207 -17.438 6.881 1.00 7.17 O

ATOM 2470 H SER A 156 32.483 -19.346 7.518 1.00 0.00 H

ATOM 2471 HA SER A 156 31.885 -16.824 6.242 1.00 0.00 H

ATOM 2472 HB3 SER A 156 30.255 -19.032 7.584 1.00 0.00 H

ATOM 2473 HB2 SER A 156 30.418 -18.733 5.873 1.00 0.00 H

ATOM 2474 HG SER A 156 29.164 -16.925 6.068 1.00 0.00 H

ATOM 2475 N PHE A 157 31.156 -15.298 8.184 1.00 7.30 N

ATOM 2476 CA PHE A 157 30.779 -14.369 9.256 1.00 7.74 C

ATOM 2477 C PHE A 157 29.698 -14.899 10.216 1.00 7.89 C

ATOM 2478 O PHE A 157 29.677 -14.491 11.376 1.00 8.73 O

ATOM 2479 CB PHE A 157 30.318 -13.037 8.637 1.00 0.00 C

ATOM 2480 CG PHE A 157 31.426 -12.063 8.288 1.00 0.00 C

ATOM 2481 CD1 PHE A 157 32.162 -12.204 7.094 1.00 0.00 C

ATOM 2482 CD2 PHE A 157 31.796 -11.061 9.209 1.00 0.00 C

ATOM 2483 CE1 PHE A 157 33.156 -11.289 6.781 1.00 0.00 C

ATOM 2484 CE2 PHE A 157 32.790 -10.152 8.877 1.00 0.00 C

ATOM 2485 CZ PHE A 157 33.444 -10.250 7.656 1.00 0.00 C

ATOM 2486 HB2 PHE A 157 29.735 -13.234 7.736 1.00 0.00 H

ATOM 2487 HB3 PHE A 157 29.632 -12.515 9.308 1.00 0.00 H

ATOM 2488 HD1 PHE A 157 31.936 -12.998 6.403 1.00 0.00 H

ATOM 2489 HD2 PHE A 157 31.279 -10.969 10.153 1.00 0.00 H

ATOM 2490 HE1 PHE A 157 33.693 -11.377 5.848 1.00 0.00 H

ATOM 2491 HE2 PHE A 157 33.041 -9.353 9.560 1.00 0.00 H

ATOM 2492 HZ PHE A 157 34.179 -9.514 7.379 1.00 0.00 H

ATOM 2493 H PHE A 157 30.983 -14.974 7.240 1.00 0.00 H

ATOM 2494 HA PHE A 157 31.667 -14.183 9.863 1.00 0.00 H

ATOM 2495 N PHE A 158 28.843 -15.803 9.712 1.00 7.64 N

ATOM 2496 CA PHE A 158 27.763 -16.447 10.454 1.00 7.54 C

ATOM 2497 C PHE A 158 28.139 -17.859 10.930 1.00 7.84 C

ATOM 2498 O PHE A 158 27.275 -18.549 11.457 1.00 7.77 O

ATOM 2499 CB PHE A 158 26.498 -16.446 9.568 1.00 7.35 C

ATOM 2500 CG PHE A 158 26.044 -15.080 9.069 1.00 6.75 C

ATOM 2501 CD1 PHE A 158 26.273 -13.903 9.820 1.00 6.97 C

ATOM 2502 CD2 PHE A 158 25.251 -14.995 7.908 1.00 6.89 C

ATOM 2503 CE1 PHE A 158 25.813 -12.680 9.364 1.00 6.98 C

ATOM 2504 CE2 PHE A 158 24.790 -13.761 7.470 1.00 6.98 C

ATOM 2505 CZ PHE A 158 25.084 -12.609 8.188 1.00 7.25 C

ATOM 2506 H PHE A 158 28.943 -16.090 8.747 1.00 0.00 H

ATOM 2507 HA PHE A 158 27.547 -15.892 11.366 1.00 0.00 H

ATOM 2508 HB3 PHE A 158 25.662 -16.891 10.104 1.00 0.00 H

ATOM 2509 HB2 PHE A 158 26.669 -17.083 8.698 1.00 0.00 H

ATOM 2510 HD1 PHE A 158 26.826 -13.926 10.746 1.00 0.00 H

ATOM 2511 HD2 PHE A 158 25.008 -15.887 7.351 1.00 0.00 H

ATOM 2512 HE1 PHE A 158 26.015 -11.782 9.929 1.00 0.00 H

ATOM 2513 HE2 PHE A 158 24.195 -13.694 6.572 1.00 0.00 H

ATOM 2514 HZ PHE A 158 24.738 -11.649 7.842 1.00 0.00 H

ATOM 2515 N GLY A 159 29.412 -18.259 10.780 1.00 7.75 N

ATOM 2516 CA GLY A 159 29.938 -19.549 11.211 1.00 7.75 C

ATOM 2517 C GLY A 159 29.850 -20.566 10.071 1.00 7.59 C

ATOM 2518 O GLY A 159 28.964 -20.505 9.217 1.00 7.75 O

ATOM 2519 H GLY A 159 30.074 -17.628 10.347 1.00 0.00 H

ATOM 2520 HA3 GLY A 159 29.396 -19.939 12.074 1.00 0.00 H

ATOM 2521 HA2 GLY A 159 30.977 -19.412 11.515 1.00 0.00 H

ATOM 2522 N GLY A 160 30.771 -21.541 10.104 1.00 6.97 N

ATOM 2523 CA GLY A 160 30.857 -22.624 9.126 1.00 7.32 C

ATOM 2524 C GLY A 160 31.685 -22.186 7.926 1.00 7.04 C

ATOM 2525 O GLY A 160 32.305 -21.126 7.948 1.00 7.50 O

ATOM 2526 H GLY A 160 31.466 -21.533 10.835 1.00 0.00 H

ATOM 2527 HA3 GLY A 160 29.870 -22.920 8.774 1.00 0.00 H

ATOM 2528 HA2 GLY A 160 31.326 -23.491 9.591 1.00 0.00 H

ATOM 2529 N SER A 161 31.710 -23.031 6.883 1.00 7.35 N

ATOM 2530 CA SER A 161 32.430 -22.779 5.635 1.00 7.09 C

ATOM 2531 C SER A 161 31.874 -21.552 4.894 1.00 7.26 C

ATOM 2532 O SER A 161 30.657 -21.400 4.789 1.00 7.78 O

ATOM 2533 CB SER A 161 32.391 -24.053 4.767 1.00 7.68 C

ATOM 2534 OG SER A 161 33.146 -23.905 3.579 1.00 8.43 O

ATOM 2535 H SER A 161 31.173 -23.884 6.921 1.00 0.00 H

ATOM 2536 HA SER A 161 33.473 -22.579 5.873 1.00 0.00 H

ATOM 2537 HB3 SER A 161 31.365 -24.306 4.496 1.00 0.00 H

ATOM 2538 HB2 SER A 161 32.795 -24.900 5.322 1.00 0.00 H

ATOM 2539 HG SER A 161 34.080 -23.878 3.805 1.00 0.00 H

ATOM 2540 N ALA A 162 32.795 -20.737 4.357 1.00 6.81 N

ATOM 2541 CA ALA A 162 32.510 -19.624 3.454 1.00 6.90 C

ATOM 2542 C ALA A 162 32.271 -20.080 2.001 1.00 6.95 C

ATOM 2543 O ALA A 162 32.395 -19.258 1.097 1.00 6.91 O

ATOM 2544 CB ALA A 162 33.655 -18.602 3.528 1.00 7.01 C

ATOM 2545 H ALA A 162 33.776 -20.920 4.527 1.00 0.00 H

ATOM 2546 HA ALA A 162 31.600 -19.132 3.797 1.00 0.00 H

ATOM 2547 HB1 ALA A 162 33.409 -17.695 2.976 1.00 0.00 H

ATOM 2548 HB2 ALA A 162 33.856 -18.301 4.552 1.00 0.00 H

ATOM 2549 HB3 ALA A 162 34.578 -19.006 3.114 1.00 0.00 H

ATOM 2550 N TRP A 163 31.922 -21.360 1.792 1.00 7.18 N

ATOM 2551 CA TRP A 163 31.622 -21.951 0.492 1.00 7.54 C

ATOM 2552 C TRP A 163 30.364 -22.824 0.583 1.00 7.85 C

ATOM 2553 O TRP A 163 30.136 -23.466 1.610 1.00 9.05 O

ATOM 2554 CB TRP A 163 32.826 -22.784 0.020 1.00 7.88 C

ATOM 2555 CG TRP A 163 34.104 -22.039 -0.210 1.00 7.35 C

ATOM 2556 CD1 TRP A 163 35.056 -21.808 0.721 1.00 8.09 C

ATOM 2557 CD2 TRP A 163 34.572 -21.387 -1.430 1.00 8.26 C

ATOM 2558 NE1 TRP A 163 36.082 -21.085 0.154 1.00 7.55 N

ATOM 2559 CE2 TRP A 163 35.845 -20.799 -1.173 1.00 7.78 C

ATOM 2560 CE3 TRP A 163 34.061 -21.246 -2.739 1.00 7.75 C

ATOM 2561 CZ2 TRP A 163 36.569 -20.109 -2.162 1.00 7.79 C

ATOM 2562 CZ3 TRP A 163 34.774 -20.568 -3.744 1.00 7.53 C

ATOM 2563 CH2 TRP A 163 36.025 -19.995 -3.456 1.00 8.17 C

ATOM 2564 H TRP A 163 31.832 -21.983 2.582 1.00 0.00 H

ATOM 2565 HA TRP A 163 31.433 -21.162 -0.234 1.00 0.00 H

ATOM 2566 HB3 TRP A 163 32.576 -23.265 -0.920 1.00 0.00 H

ATOM 2567 HB2 TRP A 163 33.023 -23.591 0.726 1.00 0.00 H

ATOM 2568 HD1 TRP A 163 35.010 -22.151 1.745 1.00 0.00 H

ATOM 2569 HE1 TRP A 163 36.910 -20.826 0.680 1.00 0.00 H

ATOM 2570 HE3 TRP A 163 33.099 -21.664 -2.973 1.00 0.00 H

ATOM 2571 HZ2 TRP A 163 37.529 -19.673 -1.929 1.00 0.00 H

ATOM 2572 HZ3 TRP A 163 34.356 -20.484 -4.736 1.00 0.00 H

ATOM 2573 HH2 TRP A 163 36.552 -19.472 -4.237 1.00 0.00 H

ATOM 2574 N THR A 164 29.590 -22.848 -0.514 1.00 7.74 N

ATOM 2575 CA THR A 164 28.439 -23.732 -0.708 1.00 7.67 C

ATOM 2576 C THR A 164 28.604 -24.426 -2.064 1.00 7.73 C

ATOM 2577 O THR A 164 28.853 -23.753 -3.065 1.00 7.73 O

ATOM 2578 CB THR A 164 27.098 -22.946 -0.728 1.00 7.98 C

ATOM 2579 OG1 THR A 164 26.782 -22.551 0.589 1.00 7.86 O

ATOM 2580 CG2 THR A 164 25.863 -23.700 -1.264 1.00 8.14 C

ATOM 2581 H THR A 164 29.840 -22.276 -1.310 1.00 0.00 H

ATOM 2582 HA THR A 164 28.401 -24.494 0.074 1.00 0.00 H

ATOM 2583 HB THR A 164 27.227 -22.033 -1.308 1.00 0.00 H

ATOM 2584 HG1 THR A 164 25.889 -22.199 0.587 1.00 0.00 H

ATOM 2585 HG21 THR A 164 24.950 -23.128 -1.096 1.00 0.00 H

ATOM 2586 HG22 THR A 164 25.926 -23.882 -2.337 1.00 0.00 H

ATOM 2587 HG23 THR A 164 25.742 -24.663 -0.768 1.00 0.00 H

ATOM 2588 N PHE A 165 28.448 -25.760 -2.059 1.00 8.48 N

ATOM 2589 CA PHE A 165 28.541 -26.602 -3.247 1.00 9.06 C

ATOM 2590 C PHE A 165 27.269 -26.487 -4.097 1.00 9.09 C

ATOM 2591 O PHE A 165 26.167 -26.642 -3.569 1.00 9.61 O

ATOM 2592 CB PHE A 165 28.799 -28.060 -2.817 1.00 9.17 C

ATOM 2593 CG PHE A 165 29.157 -29.007 -3.949 1.00 10.59 C

ATOM 2594 CD1 PHE A 165 30.390 -28.864 -4.619 1.00 10.54 C

ATOM 2595 CD2 PHE A 165 28.238 -29.979 -4.398 1.00 11.00 C

ATOM 2596 CE1 PHE A 165 30.711 -29.714 -5.668 1.00 11.99 C

ATOM 2597 CE2 PHE A 165 28.583 -30.824 -5.444 1.00 11.02 C

ATOM 2598 CZ PHE A 165 29.819 -30.700 -6.066 1.00 11.72 C

ATOM 2599 H PHE A 165 28.229 -26.236 -1.196 1.00 0.00 H

ATOM 2600 HA PHE A 165 29.397 -26.272 -3.834 1.00 0.00 H

ATOM 2601 HB3 PHE A 165 27.941 -28.455 -2.270 1.00 0.00 H

ATOM 2602 HB2 PHE A 165 29.627 -28.088 -2.111 1.00 0.00 H

ATOM 2603 HD1 PHE A 165 31.087 -28.097 -4.319 1.00 0.00 H

ATOM 2604 HD2 PHE A 165 27.273 -30.079 -3.923 1.00 0.00 H

ATOM 2605 HE1 PHE A 165 31.654 -29.606 -6.181 1.00 0.00 H

ATOM 2606 HE2 PHE A 165 27.887 -31.580 -5.777 1.00 0.00 H

ATOM 2607 HZ PHE A 165 30.081 -31.363 -6.878 1.00 0.00 H

ATOM 2608 N ASP A 166 27.464 -26.254 -5.401 1.00 9.11 N

ATOM 2609 CA ASP A 166 26.420 -26.307 -6.415 1.00 9.63 C

ATOM 2610 C ASP A 166 26.510 -27.685 -7.083 1.00 10.17 C

ATOM 2611 O ASP A 166 27.533 -28.000 -7.691 1.00 10.23 O

ATOM 2612 CB ASP A 166 26.604 -25.180 -7.450 1.00 10.09 C

ATOM 2613 CG ASP A 166 25.526 -25.118 -8.532 1.00 10.00 C

ATOM 2614 OD1 ASP A 166 25.924 -24.919 -9.698 1.00 11.18 O

ATOM 2615 OD2 ASP A 166 24.329 -25.206 -8.177 1.00 12.19 O1-

ATOM 2616 H ASP A 166 28.404 -26.127 -5.753 1.00 0.00 H

ATOM 2617 HA ASP A 166 25.439 -26.190 -5.949 1.00 0.00 H

ATOM 2618 HB3 ASP A 166 27.561 -25.335 -7.947 1.00 0.00 H

ATOM 2619 HB2 ASP A 166 26.657 -24.210 -6.958 1.00 0.00 H

ATOM 2620 N GLU A 167 25.424 -28.461 -6.977 1.00 10.53 N

ATOM 2621 CA GLU A 167 25.288 -29.780 -7.595 1.00 11.49 C

ATOM 2622 C GLU A 167 25.023 -29.721 -9.116 1.00 11.66 C

ATOM 2623 O GLU A 167 25.096 -30.766 -9.762 1.00 11.76 O

ATOM 2624 CB GLU A 167 24.190 -30.568 -6.850 1.00 11.49 C

ATOM 2625 CG GLU A 167 24.526 -30.806 -5.360 1.00 13.48 C

ATOM 2626 CD GLU A 167 23.469 -31.602 -4.588 1.00 15.12 C

ATOM 2627 OE1 GLU A 167 22.379 -31.847 -5.150 1.00 17.29 O

ATOM 2628 OE2 GLU A 167 23.777 -31.949 -3.427 1.00 16.37 O1-

ATOM 2629 H GLU A 167 24.625 -28.133 -6.452 1.00 0.00 H

ATOM 2630 HA GLU A 167 26.229 -30.318 -7.466 1.00 0.00 H

ATOM 2631 HB3 GLU A 167 24.035 -31.531 -7.338 1.00 0.00 H

ATOM 2632 HB2 GLU A 167 23.243 -30.032 -6.932 1.00 0.00 H

ATOM 2633 HG3 GLU A 167 24.662 -29.856 -4.841 1.00 0.00 H

ATOM 2634 HG2 GLU A 167 25.472 -31.340 -5.282 1.00 0.00 H

ATOM 2635 N THR A 168 24.739 -28.522 -9.661 1.00 11.84 N

ATOM 2636 CA THR A 168 24.482 -28.297 -11.085 1.00 12.02 C

ATOM 2637 C THR A 168 25.781 -28.239 -11.917 1.00 11.83 C

ATOM 2638 O THR A 168 25.886 -28.946 -12.918 1.00 12.17 O

ATOM 2639 CB THR A 168 23.682 -26.989 -11.355 1.00 0.00 C

ATOM 2640 OG1 THR A 168 22.626 -26.848 -10.424 1.00 0.00 O

ATOM 2641 CG2 THR A 168 23.081 -26.909 -12.768 1.00 0.00 C

ATOM 2642 HB THR A 168 24.324 -26.120 -11.224 1.00 0.00 H

ATOM 2643 HG1 THR A 168 23.001 -26.592 -9.576 1.00 0.00 H

ATOM 2644 HG21 THR A 168 22.498 -25.995 -12.892 1.00 0.00 H

ATOM 2645 HG22 THR A 168 23.856 -26.899 -13.535 1.00 0.00 H

ATOM 2646 HG23 THR A 168 22.422 -27.754 -12.969 1.00 0.00 H

ATOM 2647 H THR A 168 24.702 -27.706 -9.067 1.00 0.00 H

ATOM 2648 HA THR A 168 23.886 -29.134 -11.456 1.00 0.00 H

ATOM 2649 N THR A 169 26.746 -27.415 -11.469 1.00 11.10 N

ATOM 2650 CA THR A 169 28.059 -27.236 -12.101 1.00 10.64 C

ATOM 2651 C THR A 169 29.139 -28.169 -11.508 1.00 10.46 C

ATOM 2652 O THR A 169 30.156 -28.379 -12.166 1.00 11.04 O

ATOM 2653 CB THR A 169 28.567 -25.776 -11.919 1.00 10.84 C

ATOM 2654 OG1 THR A 169 28.690 -25.424 -10.551 1.00 10.23 O

ATOM 2655 CG2 THR A 169 27.671 -24.740 -12.612 1.00 10.83 C

ATOM 2656 H THR A 169 26.570 -26.844 -10.653 1.00 0.00 H

ATOM 2657 HA THR A 169 27.985 -27.445 -13.170 1.00 0.00 H

ATOM 2658 HB THR A 169 29.564 -25.684 -12.354 1.00 0.00 H

ATOM 2659 HG1 THR A 169 27.817 -25.177 -10.223 1.00 0.00 H

ATOM 2660 HG21 THR A 169 28.042 -23.725 -12.460 1.00 0.00 H

ATOM 2661 HG22 THR A 169 27.632 -24.916 -13.687 1.00 0.00 H

ATOM 2662 HG23 THR A 169 26.650 -24.779 -12.238 1.00 0.00 H

ATOM 2663 N ASN A 170 28.913 -28.701 -10.294 1.00 10.47 N

ATOM 2664 CA ASN A 170 29.869 -29.466 -9.479 1.00 10.73 C

ATOM 2665 C ASN A 170 31.076 -28.613 -9.036 1.00 10.64 C

ATOM 2666 O ASN A 170 32.209 -29.096 -9.036 1.00 11.43 O

ATOM 2667 CB ASN A 170 30.296 -30.811 -10.132 1.00 0.00 C

ATOM 2668 CG ASN A 170 29.139 -31.750 -10.488 1.00 0.00 C

ATOM 2669 OD1 ASN A 170 29.137 -32.345 -11.563 1.00 0.00 O

ATOM 2670 ND2 ASN A 170 28.163 -31.908 -9.590 1.00 0.00 N

ATOM 2671 HB2 ASN A 170 30.947 -31.361 -9.451 1.00 0.00 H

ATOM 2672 HB3 ASN A 170 30.887 -30.630 -11.031 1.00 0.00 H

ATOM 2673 HD22 ASN A 170 27.378 -32.508 -9.801 1.00 0.00 H

ATOM 2674 HD21 ASN A 170 28.186 -31.414 -8.711 1.00 0.00 H

ATOM 2675 H ASN A 170 28.041 -28.483 -9.832 1.00 0.00 H

ATOM 2676 HA ASN A 170 29.342 -29.699 -8.556 1.00 0.00 H

ATOM 2677 N GLU A 171 30.796 -27.358 -8.648 1.00 9.81 N

ATOM 2678 CA GLU A 171 31.766 -26.417 -8.096 1.00 9.24 C

ATOM 2679 C GLU A 171 31.159 -25.693 -6.894 1.00 8.69 C

ATOM 2680 O GLU A 171 29.947 -25.484 -6.835 1.00 8.49 O

ATOM 2681 CB GLU A 171 32.213 -25.403 -9.171 1.00 9.43 C

ATOM 2682 CG GLU A 171 33.032 -26.043 -10.305 1.00 9.98 C

ATOM 2683 CD GLU A 171 33.606 -25.032 -11.294 1.00 11.60 C

ATOM 2684 OE1 GLU A 171 32.936 -24.009 -11.555 1.00 10.83 O

ATOM 2685 OE2 GLU A 171 34.701 -25.326 -11.821 1.00 14.02 O1-

ATOM 2686 H GLU A 171 29.841 -27.030 -8.682 1.00 0.00 H

ATOM 2687 HA GLU A 171 32.642 -26.957 -7.732 1.00 0.00 H

ATOM 2688 HB3 GLU A 171 32.799 -24.606 -8.710 1.00 0.00 H

ATOM 2689 HB2 GLU A 171 31.332 -24.920 -9.596 1.00 0.00 H

ATOM 2690 HG3 GLU A 171 32.409 -26.744 -10.857 1.00 0.00 H

ATOM 2691 HG2 GLU A 171 33.852 -26.626 -9.892 1.00 0.00 H

ATOM 2692 N PHE A 172 32.042 -25.283 -5.977 1.00 7.96 N

ATOM 2693 CA PHE A 172 31.729 -24.387 -4.873 1.00 7.66 C

ATOM 2694 C PHE A 172 31.673 -22.929 -5.340 1.00 7.42 C

ATOM 2695 O PHE A 172 32.495 -22.538 -6.168 1.00 7.12 O

ATOM 2696 CB PHE A 172 32.810 -24.523 -3.791 1.00 7.31 C

ATOM 2697 CG PHE A 172 32.776 -25.794 -2.977 1.00 6.79 C

ATOM 2698 CD1 PHE A 172 33.504 -26.927 -3.389 1.00 8.23 C

ATOM 2699 CD2 PHE A 172 31.933 -25.881 -1.851 1.00 7.68 C

ATOM 2700 CE1 PHE A 172 33.440 -28.087 -2.633 1.00 8.13 C

ATOM 2701 CE2 PHE A 172 31.885 -27.049 -1.110 1.00 8.15 C

ATOM 2702 CZ PHE A 172 32.635 -28.146 -1.503 1.00 7.96 C

ATOM 2703 H PHE A 172 33.026 -25.464 -6.124 1.00 0.00 H

ATOM 2704 HA PHE A 172 30.765 -24.657 -4.450 1.00 0.00 H

ATOM 2705 HB3 PHE A 172 32.702 -23.715 -3.077 1.00 0.00 H

ATOM 2706 HB2 PHE A 172 33.804 -24.404 -4.226 1.00 0.00 H

ATOM 2707 HD1 PHE A 172 34.130 -26.887 -4.268 1.00 0.00 H

ATOM 2708 HD2 PHE A 172 31.336 -25.036 -1.549 1.00 0.00 H

ATOM 2709 HE1 PHE A 172 34.029 -28.943 -2.916 1.00 0.00 H

ATOM 2710 HE2 PHE A 172 31.254 -27.110 -0.235 1.00 0.00 H

ATOM 2711 HZ PHE A 172 32.583 -29.051 -0.924 1.00 0.00 H

ATOM 2712 N TYR A 173 30.771 -22.145 -4.728 1.00 6.95 N

ATOM 2713 CA TYR A 173 30.738 -20.688 -4.848 1.00 7.41 C

ATOM 2714 C TYR A 173 30.989 -20.042 -3.479 1.00 7.08 C

ATOM 2715 O TYR A 173 30.502 -20.553 -2.469 1.00 7.12 O

ATOM 2716 CB TYR A 173 29.421 -20.223 -5.497 1.00 7.41 C

ATOM 2717 CG TYR A 173 28.123 -20.559 -4.779 1.00 7.12 C

ATOM 2718 CD1 TYR A 173 27.683 -19.780 -3.689 1.00 8.40 C

ATOM 2719 CD2 TYR A 173 27.318 -21.621 -5.239 1.00 7.60 C

ATOM 2720 CE1 TYR A 173 26.431 -20.024 -3.101 1.00 7.95 C

ATOM 2721 CE2 TYR A 173 26.059 -21.862 -4.657 1.00 7.15 C

ATOM 2722 CZ TYR A 173 25.607 -21.050 -3.599 1.00 8.15 C

ATOM 2723 OH TYR A 173 24.377 -21.258 -3.048 1.00 8.64 O

ATOM 2724 H TYR A 173 30.122 -22.548 -4.064 1.00 0.00 H

ATOM 2725 HA TYR A 173 31.537 -20.376 -5.510 1.00 0.00 H

ATOM 2726 HB3 TYR A 173 29.367 -20.626 -6.508 1.00 0.00 H

ATOM 2727 HB2 TYR A 173 29.454 -19.142 -5.631 1.00 0.00 H

ATOM 2728 HD1 TYR A 173 28.296 -18.982 -3.301 1.00 0.00 H

ATOM 2729 HD2 TYR A 173 27.651 -22.238 -6.059 1.00 0.00 H

ATOM 2730 HE1 TYR A 173 26.103 -19.407 -2.280 1.00 0.00 H

ATOM 2731 HE2 TYR A 173 25.440 -22.663 -5.031 1.00 0.00 H

ATOM 2732 HH TYR A 173 24.204 -20.680 -2.299 1.00 0.00 H

ATOM 2733 N LEU A 174 31.760 -18.939 -3.474 1.00 6.85 N

ATOM 2734 CA LEU A 174 32.115 -18.194 -2.265 1.00 6.65 C

ATOM 2735 C LEU A 174 30.914 -17.398 -1.749 1.00 6.56 C

ATOM 2736 O LEU A 174 30.269 -16.695 -2.526 1.00 6.52 O

ATOM 2737 CB LEU A 174 33.303 -17.247 -2.556 1.00 6.53 C

ATOM 2738 CG LEU A 174 33.895 -16.477 -1.349 1.00 6.44 C

ATOM 2739 CD1 LEU A 174 34.576 -17.410 -0.330 1.00 6.57 C

ATOM 2740 CD2 LEU A 174 34.859 -15.376 -1.824 1.00 5.85 C

ATOM 2741 H LEU A 174 32.132 -18.587 -4.346 1.00 0.00 H

ATOM 2742 HA LEU A 174 32.418 -18.923 -1.512 1.00 0.00 H

ATOM 2743 HB3 LEU A 174 32.955 -16.504 -3.273 1.00 0.00 H

ATOM 2744 HB2 LEU A 174 34.103 -17.804 -3.044 1.00 0.00 H

ATOM 2745 HG LEU A 174 33.086 -15.964 -0.827 1.00 0.00 H

ATOM 2746 HD11 LEU A 174 34.189 -17.237 0.673 1.00 0.00 H

ATOM 2747 HD12 LEU A 174 34.418 -18.461 -0.562 1.00 0.00 H

ATOM 2748 HD13 LEU A 174 35.655 -17.262 -0.298 1.00 0.00 H

ATOM 2749 HD21 LEU A 174 35.559 -15.081 -1.043 1.00 0.00 H

ATOM 2750 HD22 LEU A 174 35.441 -15.692 -2.691 1.00 0.00 H

ATOM 2751 HD23 LEU A 174 34.307 -14.481 -2.106 1.00 0.00 H

ATOM 2752 N ARG A 175 30.689 -17.490 -0.437 1.00 5.80 N

ATOM 2753 CA ARG A 175 29.724 -16.692 0.298 1.00 6.31 C

ATOM 2754 C ARG A 175 30.289 -16.468 1.702 1.00 6.49 C

ATOM 2755 O ARG A 175 30.195 -17.353 2.548 1.00 6.36 O

ATOM 2756 CB ARG A 175 28.298 -17.304 0.273 1.00 5.96 C

ATOM 2757 CG ARG A 175 28.114 -18.825 0.113 1.00 7.10 C

ATOM 2758 CD ARG A 175 28.679 -19.697 1.232 1.00 7.13 C

ATOM 2759 NE ARG A 175 28.149 -19.365 2.563 1.00 6.89 N

ATOM 2760 CZ ARG A 175 27.852 -20.225 3.553 1.00 6.22 C

ATOM 2761 NH1 ARG A 175 27.859 -21.552 3.365 1.00 8.39 N

ATOM 2762 NH2 ARG A 175 27.542 -19.738 4.760 1.00 6.02 N1+

ATOM 2763 H ARG A 175 31.281 -18.090 0.126 1.00 0.00 H

ATOM 2764 HA ARG A 175 29.651 -15.716 -0.183 1.00 0.00 H

ATOM 2765 HB3 ARG A 175 27.794 -16.888 -0.594 1.00 0.00 H

ATOM 2766 HB2 ARG A 175 27.736 -16.932 1.125 1.00 0.00 H

ATOM 2767 HG3 ARG A 175 28.615 -19.117 -0.810 1.00 0.00 H

ATOM 2768 HG2 ARG A 175 27.067 -19.073 -0.053 1.00 0.00 H

ATOM 2769 HD3 ARG A 175 29.759 -19.738 1.208 1.00 0.00 H

ATOM 2770 HD2 ARG A 175 28.352 -20.704 1.008 1.00 0.00 H

ATOM 2771 HE ARG A 175 28.168 -18.379 2.792 1.00 0.00 H

ATOM 2772 HH12 ARG A 175 27.675 -22.182 4.132 1.00 0.00 H

ATOM 2773 HH11 ARG A 175 28.077 -21.936 2.455 1.00 0.00 H

ATOM 2774 HH22 ARG A 175 27.348 -20.358 5.533 1.00 0.00 H

ATOM 2775 HH21 ARG A 175 27.562 -18.737 4.917 1.00 0.00 H

ATOM 2776 N LEU A 176 30.868 -15.279 1.935 1.00 6.44 N

ATOM 2777 CA LEU A 176 31.368 -14.870 3.250 1.00 6.36 C

ATOM 2778 C LEU A 176 30.255 -14.719 4.301 1.00 6.60 C

ATOM 2779 O LEU A 176 30.551 -14.834 5.484 1.00 6.80 O

ATOM 2780 CB LEU A 176 32.168 -13.555 3.136 1.00 6.31 C

ATOM 2781 CG LEU A 176 33.529 -13.665 2.416 1.00 6.68 C

ATOM 2782 CD1 LEU A 176 34.188 -12.277 2.341 1.00 6.68 C

ATOM 2783 CD2 LEU A 176 34.482 -14.702 3.046 1.00 7.11 C

ATOM 2784 H LEU A 176 30.922 -14.590 1.191 1.00 0.00 H

ATOM 2785 HA LEU A 176 32.023 -15.662 3.616 1.00 0.00 H

ATOM 2786 HB3 LEU A 176 32.358 -13.157 4.133 1.00 0.00 H

ATOM 2787 HB2 LEU A 176 31.548 -12.808 2.635 1.00 0.00 H

ATOM 2788 HG LEU A 176 33.332 -13.985 1.392 1.00 0.00 H

ATOM 2789 HD11 LEU A 176 34.732 -12.158 1.405 1.00 0.00 H

ATOM 2790 HD12 LEU A 176 33.456 -11.470 2.390 1.00 0.00 H

ATOM 2791 HD13 LEU A 176 34.891 -12.118 3.159 1.00 0.00 H

ATOM 2792 HD21 LEU A 176 35.500 -14.323 3.137 1.00 0.00 H

ATOM 2793 HD22 LEU A 176 34.161 -15.005 4.043 1.00 0.00 H

ATOM 2794 HD23 LEU A 176 34.537 -15.599 2.430 1.00 0.00 H

ATOM 2795 N PHE A 177 29.004 -14.502 3.871 1.00 6.00 N

ATOM 2796 CA PHE A 177 27.832 -14.427 4.743 1.00 6.33 C

ATOM 2797 C PHE A 177 27.032 -15.735 4.614 1.00 6.82 C

ATOM 2798 O PHE A 177 27.657 -16.796 4.556 1.00 6.03 O

ATOM 2799 CB PHE A 177 27.084 -13.104 4.466 1.00 6.55 C

ATOM 2800 CG PHE A 177 27.935 -11.897 4.824 1.00 6.96 C

ATOM 2801 CD1 PHE A 177 28.006 -11.468 6.166 1.00 7.12 C

ATOM 2802 CD2 PHE A 177 28.854 -11.371 3.891 1.00 7.05 C

ATOM 2803 CE1 PHE A 177 28.954 -10.530 6.549 1.00 7.10 C

ATOM 2804 CE2 PHE A 177 29.805 -10.446 4.295 1.00 8.31 C

ATOM 2805 CZ PHE A 177 29.864 -10.042 5.622 1.00 8.91 C

ATOM 2806 H PHE A 177 28.829 -14.423 2.879 1.00 0.00 H

ATOM 2807 HA PHE A 177 28.136 -14.397 5.791 1.00 0.00 H

ATOM 2808 HB3 PHE A 177 26.174 -13.052 5.061 1.00 0.00 H

ATOM 2809 HB2 PHE A 177 26.788 -13.039 3.419 1.00 0.00 H

ATOM 2810 HD1 PHE A 177 27.353 -11.897 6.909 1.00 0.00 H

ATOM 2811 HD2 PHE A 177 28.853 -11.721 2.868 1.00 0.00 H

ATOM 2812 HE1 PHE A 177 29.017 -10.217 7.580 1.00 0.00 H

ATOM 2813 HE2 PHE A 177 30.521 -10.066 3.583 1.00 0.00 H

ATOM 2814 HZ PHE A 177 30.642 -9.368 5.939 1.00 0.00 H

ATOM 2815 N ALA A 178 25.689 -15.683 4.599 1.00 6.67 N

ATOM 2816 CA ALA A 178 24.827 -16.868 4.484 1.00 7.12 C

ATOM 2817 C ALA A 178 25.004 -17.605 3.143 1.00 6.83 C

ATOM 2818 O ALA A 178 25.540 -17.032 2.196 1.00 6.90 O

ATOM 2819 CB ALA A 178 23.362 -16.446 4.679 1.00 0.00 C

ATOM 2820 HB1 ALA A 178 22.998 -15.855 3.838 1.00 0.00 H

ATOM 2821 HB2 ALA A 178 22.709 -17.314 4.774 1.00 0.00 H

ATOM 2822 HB3 ALA A 178 23.237 -15.847 5.582 1.00 0.00 H

ATOM 2823 H ALA A 178 25.215 -14.789 4.630 1.00 0.00 H

ATOM 2824 HA ALA A 178 25.097 -17.553 5.290 1.00 0.00 H

ATOM 2825 N SER A 179 24.532 -18.862 3.079 1.00 7.40 N

ATOM 2826 CA SER A 179 24.601 -19.732 1.895 1.00 7.70 C

ATOM 2827 C SER A 179 23.955 -19.163 0.613 1.00 7.71 C

ATOM 2828 O SER A 179 24.314 -19.609 -0.477 1.00 7.79 O

ATOM 2829 CB SER A 179 24.032 -21.117 2.258 1.00 8.06 C

ATOM 2830 OG SER A 179 22.628 -21.090 2.428 1.00 10.70 O

ATOM 2831 H SER A 179 24.080 -19.265 3.887 1.00 0.00 H

ATOM 2832 HA SER A 179 25.658 -19.870 1.674 1.00 0.00 H

ATOM 2833 HB3 SER A 179 24.501 -21.502 3.165 1.00 0.00 H

ATOM 2834 HB2 SER A 179 24.254 -21.833 1.467 1.00 0.00 H

ATOM 2835 HG SER A 179 22.331 -21.975 2.657 1.00 0.00 H

ATOM 2836 N ARG A 180 23.041 -18.189 0.773 1.00 7.10 N

ATOM 2837 CA ARG A 180 22.352 -17.492 -0.307 1.00 7.00 C

ATOM 2838 C ARG A 180 22.839 -16.036 -0.476 1.00 7.17 C

ATOM 2839 O ARG A 180 22.068 -15.220 -0.971 1.00 6.98 O

ATOM 2840 CB ARG A 180 20.821 -17.608 -0.118 1.00 0.00 C

ATOM 2841 CG ARG A 180 20.310 -19.061 -0.158 1.00 0.00 C

ATOM 2842 CD ARG A 180 18.796 -19.165 0.069 1.00 0.00 C

ATOM 2843 NE ARG A 180 18.282 -20.520 -0.160 1.00 0.00 N

ATOM 2844 CZ ARG A 180 17.900 -21.033 -1.342 1.00 0.00 C

ATOM 2845 NH1 ARG A 180 17.967 -20.311 -2.470 1.00 0.00 N

ATOM 2846 NH2 ARG A 180 17.438 -22.289 -1.387 1.00 0.00 N1+

ATOM 2847 HB2 ARG A 180 20.524 -17.132 0.818 1.00 0.00 H

ATOM 2848 HB3 ARG A 180 20.313 -17.061 -0.914 1.00 0.00 H

ATOM 2849 HG2 ARG A 180 20.613 -19.589 -1.063 1.00 0.00 H

ATOM 2850 HG3 ARG A 180 20.791 -19.581 0.671 1.00 0.00 H

ATOM 2851 HD2 ARG A 180 18.652 -19.063 1.144 1.00 0.00 H

ATOM 2852 HD3 ARG A 180 18.212 -18.369 -0.394 1.00 0.00 H

ATOM 2853 HE ARG A 180 18.257 -21.119 0.652 1.00 0.00 H

ATOM 2854 HH12 ARG A 180 17.636 -20.677 -3.352 1.00 0.00 H

ATOM 2855 HH11 ARG A 180 18.319 -19.365 -2.442 1.00 0.00 H

ATOM 2856 HH22 ARG A 180 17.138 -22.690 -2.264 1.00 0.00 H

ATOM 2857 HH21 ARG A 180 17.375 -22.844 -0.546 1.00 0.00 H

ATOM 2858 H ARG A 180 22.815 -17.884 1.708 1.00 0.00 H

ATOM 2859 HA ARG A 180 22.580 -17.998 -1.236 1.00 0.00 H

ATOM 2860 N GLN A 181 24.094 -15.733 -0.095 1.00 6.85 N

ATOM 2861 CA GLN A 181 24.732 -14.417 -0.235 1.00 6.67 C

ATOM 2862 C GLN A 181 26.095 -14.554 -0.915 1.00 6.57 C

ATOM 2863 O GLN A 181 27.131 -14.412 -0.261 1.00 6.95 O

ATOM 2864 CB GLN A 181 24.861 -13.730 1.135 1.00 6.86 C

ATOM 2865 CG GLN A 181 23.539 -13.151 1.632 1.00 6.38 C

ATOM 2866 CD GLN A 181 23.704 -12.604 3.040 1.00 6.75 C

ATOM 2867 OE1 GLN A 181 23.873 -13.373 3.980 1.00 8.50 O

ATOM 2868 NE2 GLN A 181 23.656 -11.283 3.199 1.00 4.78 N

ATOM 2869 H GLN A 181 24.668 -16.450 0.329 1.00 0.00 H

ATOM 2870 HA GLN A 181 24.130 -13.772 -0.875 1.00 0.00 H

ATOM 2871 HB3 GLN A 181 25.573 -12.904 1.076 1.00 0.00 H

ATOM 2872 HB2 GLN A 181 25.273 -14.430 1.862 1.00 0.00 H

ATOM 2873 HG3 GLN A 181 22.776 -13.929 1.661 1.00 0.00 H

ATOM 2874 HG2 GLN A 181 23.189 -12.373 0.955 1.00 0.00 H

ATOM 2875 HE22 GLN A 181 23.717 -10.899 4.138 1.00 0.00 H

ATOM 2876 HE21 GLN A 181 23.506 -10.665 2.414 1.00 0.00 H

ATOM 2877 N VAL A 182 26.066 -14.848 -2.220 1.00 6.47 N

ATOM 2878 CA VAL A 182 27.262 -15.100 -3.019 1.00 6.09 C

ATOM 2879 C VAL A 182 28.048 -13.802 -3.252 1.00 5.90 C

ATOM 2880 O VAL A 182 27.467 -12.836 -3.751 1.00 6.28 O

ATOM 2881 CB VAL A 182 26.930 -15.689 -4.413 1.00 0.00 C

ATOM 2882 CG1 VAL A 182 28.176 -16.337 -5.048 1.00 0.00 C

ATOM 2883 CG2 VAL A 182 25.765 -16.681 -4.337 1.00 0.00 C

ATOM 2884 HB VAL A 182 26.607 -14.895 -5.088 1.00 0.00 H

ATOM 2885 HG11 VAL A 182 27.985 -16.650 -6.069 1.00 0.00 H

ATOM 2886 HG12 VAL A 182 29.028 -15.660 -5.080 1.00 0.00 H

ATOM 2887 HG13 VAL A 182 28.498 -17.210 -4.489 1.00 0.00 H

ATOM 2888 HG21 VAL A 182 25.676 -17.272 -5.240 1.00 0.00 H

ATOM 2889 HG22 VAL A 182 25.881 -17.369 -3.502 1.00 0.00 H

ATOM 2890 HG23 VAL A 182 24.820 -16.152 -4.210 1.00 0.00 H

ATOM 2891 H VAL A 182 25.182 -14.912 -2.703 1.00 0.00 H

ATOM 2892 HA VAL A 182 27.887 -15.810 -2.483 1.00 0.00 H

ATOM 2893 N ASP A 183 29.346 -13.820 -2.903 1.00 5.79 N

ATOM 2894 CA ASP A 183 30.267 -12.711 -3.146 1.00 5.98 C

ATOM 2895 C ASP A 183 30.440 -12.425 -4.640 1.00 6.08 C

ATOM 2896 O ASP A 183 30.850 -13.308 -5.394 1.00 6.75 O

ATOM 2897 CB ASP A 183 31.654 -12.836 -2.478 1.00 6.30 C

ATOM 2898 CG ASP A 183 31.646 -12.791 -0.949 1.00 7.28 C

ATOM 2899 OD1 ASP A 183 30.813 -13.487 -0.333 1.00 6.27 O

ATOM 2900 OD2 ASP A 183 32.543 -12.102 -0.416 1.00 6.25 O1-

ATOM 2901 H ASP A 183 29.747 -14.651 -2.490 1.00 0.00 H

ATOM 2902 HA ASP A 183 29.790 -11.842 -2.694 1.00 0.00 H

ATOM 2903 HB3 ASP A 183 32.285 -12.019 -2.830 1.00 0.00 H

ATOM 2904 HB2 ASP A 183 32.145 -13.758 -2.789 1.00 0.00 H

ATOM 2905 N LEU A 184 30.151 -11.168 -5.001 1.00 5.97 N

ATOM 2906 CA LEU A 184 30.404 -10.588 -6.312 1.00 5.70 C

ATOM 2907 C LEU A 184 31.918 -10.462 -6.533 1.00 6.35 C

ATOM 2908 O LEU A 184 32.631 -9.999 -5.641 1.00 6.47 O

ATOM 2909 CB LEU A 184 29.722 -9.204 -6.387 1.00 5.94 C

ATOM 2910 CG LEU A 184 28.177 -9.248 -6.477 1.00 5.77 C

ATOM 2911 CD1 LEU A 184 27.536 -7.920 -6.030 1.00 7.99 C

ATOM 2912 CD2 LEU A 184 27.700 -9.645 -7.888 1.00 7.77 C

ATOM 2913 H LEU A 184 29.849 -10.516 -4.289 1.00 0.00 H

ATOM 2914 HA LEU A 184 29.981 -11.253 -7.065 1.00 0.00 H

ATOM 2915 HB3 LEU A 184 30.108 -8.646 -7.240 1.00 0.00 H

ATOM 2916 HB2 LEU A 184 30.017 -8.628 -5.507 1.00 0.00 H

ATOM 2917 HG LEU A 184 27.821 -10.015 -5.788 1.00 0.00 H

ATOM 2918 HD11 LEU A 184 26.708 -7.620 -6.672 1.00 0.00 H

ATOM 2919 HD12 LEU A 184 27.139 -7.999 -5.019 1.00 0.00 H

ATOM 2920 HD13 LEU A 184 28.254 -7.103 -6.031 1.00 0.00 H

ATOM 2921 HD21 LEU A 184 26.790 -10.239 -7.831 1.00 0.00 H

ATOM 2922 HD22 LEU A 184 27.478 -8.771 -8.502 1.00 0.00 H

ATOM 2923 HD23 LEU A 184 28.442 -10.231 -8.430 1.00 0.00 H

ATOM 2924 N ASN A 185 32.370 -10.883 -7.721 1.00 6.13 N

ATOM 2925 CA ASN A 185 33.759 -10.797 -8.149 1.00 6.18 C

ATOM 2926 C ASN A 185 34.012 -9.407 -8.747 1.00 6.35 C

ATOM 2927 O ASN A 185 33.744 -9.189 -9.929 1.00 6.59 O

ATOM 2928 CB ASN A 185 34.063 -11.941 -9.145 1.00 6.82 C

ATOM 2929 CG ASN A 185 35.549 -12.089 -9.495 1.00 7.80 C

ATOM 2930 OD1 ASN A 185 36.422 -11.652 -8.751 1.00 8.40 O

ATOM 2931 ND2 ASN A 185 35.845 -12.715 -10.633 1.00 10.40 N

ATOM 2932 H ASN A 185 31.714 -11.256 -8.397 1.00 0.00 H

ATOM 2933 HA ASN A 185 34.417 -10.909 -7.286 1.00 0.00 H

ATOM 2934 HB3 ASN A 185 33.483 -11.814 -10.058 1.00 0.00 H

ATOM 2935 HB2 ASN A 185 33.751 -12.889 -8.707 1.00 0.00 H

ATOM 2936 HD22 ASN A 185 36.808 -12.851 -10.904 1.00 0.00 H

ATOM 2937 HD21 ASN A 185 35.105 -13.040 -11.244 1.00 0.00 H

ATOM 2938 N TRP A 186 34.545 -8.505 -7.909 1.00 6.05 N

ATOM 2939 CA TRP A 186 34.991 -7.170 -8.309 1.00 6.23 C

ATOM 2940 C TRP A 186 36.227 -7.170 -9.225 1.00 6.37 C

ATOM 2941 O TRP A 186 36.473 -6.147 -9.857 1.00 7.28 O

ATOM 2942 CB TRP A 186 35.212 -6.286 -7.065 1.00 6.11 C

ATOM 2943 CG TRP A 186 33.969 -5.746 -6.424 1.00 6.13 C

ATOM 2944 CD1 TRP A 186 33.001 -6.472 -5.820 1.00 6.19 C

ATOM 2945 CD2 TRP A 186 33.535 -4.354 -6.337 1.00 5.52 C

ATOM 2946 NE1 TRP A 186 31.990 -5.636 -5.399 1.00 6.17 N

ATOM 2947 CE2 TRP A 186 32.262 -4.319 -5.696 1.00 6.47 C

ATOM 2948 CE3 TRP A 186 34.082 -3.111 -6.734 1.00 6.12 C

ATOM 2949 CZ2 TRP A 186 31.562 -3.123 -5.483 1.00 6.91 C

ATOM 2950 CZ3 TRP A 186 33.391 -1.900 -6.521 1.00 6.05 C

ATOM 2951 CH2 TRP A 186 32.128 -1.905 -5.899 1.00 5.96 C

ATOM 2952 H TRP A 186 34.709 -8.759 -6.946 1.00 0.00 H

ATOM 2953 HA TRP A 186 34.197 -6.713 -8.903 1.00 0.00 H

ATOM 2954 HB3 TRP A 186 35.802 -5.413 -7.341 1.00 0.00 H

ATOM 2955 HB2 TRP A 186 35.802 -6.817 -6.318 1.00 0.00 H

ATOM 2956 HD1 TRP A 186 33.022 -7.543 -5.706 1.00 0.00 H

ATOM 2957 HE1 TRP A 186 31.153 -5.967 -4.932 1.00 0.00 H

ATOM 2958 HE3 TRP A 186 35.054 -3.089 -7.200 1.00 0.00 H

ATOM 2959 HZ2 TRP A 186 30.602 -3.154 -5.000 1.00 0.00 H

ATOM 2960 HZ3 TRP A 186 33.834 -0.966 -6.835 1.00 0.00 H

ATOM 2961 HH2 TRP A 186 31.600 -0.978 -5.732 1.00 0.00 H

ATOM 2962 N GLU A 187 36.958 -8.295 -9.336 1.00 6.34 N

ATOM 2963 CA GLU A 187 38.057 -8.460 -10.296 1.00 7.13 C

ATOM 2964 C GLU A 187 37.573 -8.414 -11.763 1.00 7.39 C

ATOM 2965 O GLU A 187 38.313 -7.929 -12.619 1.00 9.23 O

ATOM 2966 CB GLU A 187 38.834 -9.766 -10.012 1.00 6.80 C

ATOM 2967 CG GLU A 187 39.278 -9.994 -8.546 1.00 7.88 C

ATOM 2968 CD GLU A 187 40.196 -8.906 -7.984 1.00 9.04 C

ATOM 2969 OE1 GLU A 187 41.119 -8.488 -8.718 1.00 10.58 O

ATOM 2970 OE2 GLU A 187 39.940 -8.481 -6.834 1.00 8.71 O1-

ATOM 2971 H GLU A 187 36.716 -9.110 -8.790 1.00 0.00 H

ATOM 2972 HA GLU A 187 38.739 -7.620 -10.158 1.00 0.00 H

ATOM 2973 HB3 GLU A 187 38.236 -10.620 -10.329 1.00 0.00 H

ATOM 2974 HB2 GLU A 187 39.718 -9.794 -10.651 1.00 0.00 H

ATOM 2975 HG3 GLU A 187 38.407 -10.087 -7.897 1.00 0.00 H

ATOM 2976 HG2 GLU A 187 39.807 -10.945 -8.473 1.00 0.00 H

ATOM 2977 N ASN A 188 36.329 -8.866 -12.007 1.00 8.20 N

ATOM 2978 CA ASN A 188 35.613 -8.749 -13.277 1.00 9.07 C

ATOM 2979 C ASN A 188 35.084 -7.311 -13.429 1.00 9.39 C

ATOM 2980 O ASN A 188 34.253 -6.890 -12.624 1.00 9.11 O

ATOM 2981 CB ASN A 188 34.479 -9.804 -13.288 1.00 9.08 C

ATOM 2982 CG ASN A 188 33.799 -10.002 -14.648 1.00 9.73 C

ATOM 2983 OD1 ASN A 188 33.262 -9.062 -15.227 1.00 10.11 O

ATOM 2984 ND2 ASN A 188 33.792 -11.237 -15.153 1.00 11.37 N

ATOM 2985 H ASN A 188 35.783 -9.224 -11.236 1.00 0.00 H

ATOM 2986 HA ASN A 188 36.290 -8.953 -14.110 1.00 0.00 H

ATOM 2987 HB3 ASN A 188 33.718 -9.578 -12.543 1.00 0.00 H

ATOM 2988 HB2 ASN A 188 34.910 -10.760 -12.988 1.00 0.00 H

ATOM 2989 HD22 ASN A 188 33.359 -11.413 -16.048 1.00 0.00 H

ATOM 2990 HD21 ASN A 188 34.219 -11.999 -14.647 1.00 0.00 H

ATOM 2991 N GLU A 189 35.577 -6.587 -14.449 1.00 10.21 N

ATOM 2992 CA GLU A 189 35.211 -5.192 -14.697 1.00 10.97 C

ATOM 2993 C GLU A 189 33.751 -5.009 -15.148 1.00 10.57 C

ATOM 2994 O GLU A 189 33.116 -4.077 -14.663 1.00 10.12 O

ATOM 2995 CB GLU A 189 36.238 -4.518 -15.632 1.00 12.04 C

ATOM 2996 CG GLU A 189 35.958 -3.018 -15.889 1.00 15.49 C

ATOM 2997 CD GLU A 189 37.052 -2.287 -16.670 1.00 19.64 C

ATOM 2998 OE1 GLU A 189 36.884 -1.057 -16.829 1.00 22.90 O

ATOM 2999 OE2 GLU A 189 38.031 -2.945 -17.089 1.00 21.78 O1-

ATOM 3000 H GLU A 189 36.258 -6.993 -15.075 1.00 0.00 H

ATOM 3001 HA GLU A 189 35.295 -4.677 -13.738 1.00 0.00 H

ATOM 3002 HB3 GLU A 189 36.271 -5.052 -16.583 1.00 0.00 H

ATOM 3003 HB2 GLU A 189 37.229 -4.626 -15.189 1.00 0.00 H

ATOM 3004 HG3 GLU A 189 35.823 -2.502 -14.938 1.00 0.00 H

ATOM 3005 HG2 GLU A 189 35.028 -2.896 -16.445 1.00 0.00 H

ATOM 3006 N ASP A 190 33.224 -5.915 -15.993 1.00 10.20 N

ATOM 3007 CA ASP A 190 31.808 -5.950 -16.406 1.00 10.03 C

ATOM 3008 C ASP A 190 30.839 -6.094 -15.220 1.00 9.36 C

ATOM 3009 O ASP A 190 29.776 -5.474 -15.236 1.00 9.70 O

ATOM 3010 CB ASP A 190 31.490 -7.044 -17.454 1.00 10.86 C

ATOM 3011 CG ASP A 190 32.428 -7.067 -18.662 1.00 13.59 C

ATOM 3012 OD1 ASP A 190 32.740 -5.968 -19.170 1.00 16.16 O

ATOM 3013 OD2 ASP A 190 32.748 -8.190 -19.109 1.00 17.87 O1-

ATOM 3014 H ASP A 190 33.803 -6.665 -16.344 1.00 0.00 H

ATOM 3015 HA ASP A 190 31.597 -4.983 -16.866 1.00 0.00 H

ATOM 3016 HB3 ASP A 190 30.468 -6.940 -17.821 1.00 0.00 H

ATOM 3017 HB2 ASP A 190 31.543 -8.021 -16.971 1.00 0.00 H

ATOM 3018 N CYS A 191 31.254 -6.869 -14.201 1.00 8.94 N

ATOM 3019 CA CYS A 191 30.559 -7.019 -12.926 1.00 8.17 C

ATOM 3020 C CYS A 191 30.528 -5.697 -12.131 1.00 8.13 C

ATOM 3021 O CYS A 191 29.452 -5.342 -11.655 1.00 8.26 O

ATOM 3022 CB CYS A 191 31.130 -8.190 -12.101 1.00 7.86 C

ATOM 3023 SG CYS A 191 30.102 -8.591 -10.660 1.00 8.88 S

ATOM 3024 H CYS A 191 32.138 -7.353 -14.285 1.00 0.00 H

ATOM 3025 HA CYS A 191 29.526 -7.275 -13.170 1.00 0.00 H

ATOM 3026 HB3 CYS A 191 32.133 -7.962 -11.747 1.00 0.00 H

ATOM 3027 HB2 CYS A 191 31.209 -9.084 -12.718 1.00 0.00 H

ATOM 3028 HG CYS A 191 30.877 -9.578 -10.201 1.00 0.00 H

ATOM 3029 N ARG A 192 31.662 -4.966 -12.055 1.00 7.81 N

ATOM 3030 CA ARG A 192 31.732 -3.643 -11.413 1.00 7.72 C

ATOM 3031 C ARG A 192 30.844 -2.586 -12.088 1.00 7.92 C

ATOM 3032 O ARG A 192 30.153 -1.866 -11.373 1.00 7.78 O

ATOM 3033 CB ARG A 192 33.167 -3.084 -11.303 1.00 7.33 C

ATOM 3034 CG ARG A 192 34.184 -3.986 -10.587 1.00 7.38 C

ATOM 3035 CD ARG A 192 35.366 -3.195 -9.998 1.00 6.62 C

ATOM 3036 NE ARG A 192 36.182 -2.518 -11.011 1.00 6.94 N

ATOM 3037 CZ ARG A 192 37.103 -3.035 -11.837 1.00 8.66 C

ATOM 3038 NH1 ARG A 192 37.406 -4.339 -11.828 1.00 7.25 N

ATOM 3039 NH2 ARG A 192 37.729 -2.213 -12.688 1.00 10.42 N1+

ATOM 3040 H ARG A 192 32.510 -5.307 -12.486 1.00 0.00 H

ATOM 3041 HA ARG A 192 31.362 -3.771 -10.394 1.00 0.00 H

ATOM 3042 HB3 ARG A 192 33.094 -2.148 -10.745 1.00 0.00 H

ATOM 3043 HB2 ARG A 192 33.560 -2.808 -12.282 1.00 0.00 H

ATOM 3044 HG3 ARG A 192 34.542 -4.716 -11.309 1.00 0.00 H

ATOM 3045 HG2 ARG A 192 33.719 -4.553 -9.781 1.00 0.00 H

ATOM 3046 HD3 ARG A 192 35.961 -3.768 -9.290 1.00 0.00 H

ATOM 3047 HD2 ARG A 192 34.951 -2.371 -9.417 1.00 0.00 H

ATOM 3048 HE ARG A 192 35.984 -1.519 -11.097 1.00 0.00 H

ATOM 3049 HH12 ARG A 192 38.075 -4.721 -12.480 1.00 0.00 H

ATOM 3050 HH11 ARG A 192 36.960 -4.956 -11.156 1.00 0.00 H

ATOM 3051 HH22 ARG A 192 38.408 -2.561 -13.349 1.00 0.00 H

ATOM 3052 HH21 ARG A 192 37.533 -1.214 -12.661 1.00 0.00 H

ATOM 3053 N ARG A 193 30.856 -2.522 -13.433 1.00 8.24 N

ATOM 3054 CA ARG A 193 30.027 -1.600 -14.218 1.00 8.10 C

ATOM 3055 C ARG A 193 28.520 -1.845 -14.024 1.00 7.86 C

ATOM 3056 O ARG A 193 27.777 -0.876 -13.885 1.00 8.00 O

ATOM 3057 CB ARG A 193 30.384 -1.670 -15.720 1.00 0.00 C

ATOM 3058 CG ARG A 193 31.841 -1.333 -16.090 1.00 0.00 C

ATOM 3059 CD ARG A 193 32.230 0.144 -15.961 1.00 0.00 C

ATOM 3060 NE ARG A 193 33.665 0.325 -16.231 1.00 0.00 N

ATOM 3061 CZ ARG A 193 34.278 1.483 -16.520 1.00 0.00 C

ATOM 3062 NH1 ARG A 193 33.601 2.641 -16.530 1.00 0.00 N

ATOM 3063 NH2 ARG A 193 35.585 1.468 -16.813 1.00 0.00 N1+

ATOM 3064 HB2 ARG A 193 30.167 -2.677 -16.083 1.00 0.00 H

ATOM 3065 HB3 ARG A 193 29.725 -1.005 -16.281 1.00 0.00 H

ATOM 3066 HG2 ARG A 193 32.573 -1.960 -15.595 1.00 0.00 H

ATOM 3067 HG3 ARG A 193 31.920 -1.580 -17.150 1.00 0.00 H

ATOM 3068 HD2 ARG A 193 31.736 0.671 -16.778 1.00 0.00 H

ATOM 3069 HD3 ARG A 193 31.893 0.611 -15.036 1.00 0.00 H

ATOM 3070 HE ARG A 193 34.233 -0.511 -16.191 1.00 0.00 H

ATOM 3071 HH12 ARG A 193 34.036 3.496 -16.839 1.00 0.00 H

ATOM 3072 HH11 ARG A 193 32.618 2.641 -16.301 1.00 0.00 H

ATOM 3073 HH22 ARG A 193 36.080 2.306 -17.072 1.00 0.00 H

ATOM 3074 HH21 ARG A 193 36.091 0.581 -16.814 1.00 0.00 H

ATOM 3075 H ARG A 193 31.459 -3.146 -13.955 1.00 0.00 H

ATOM 3076 HA ARG A 193 30.239 -0.590 -13.861 1.00 0.00 H

ATOM 3077 N ALA A 194 28.108 -3.125 -13.980 1.00 7.27 N

ATOM 3078 CA ALA A 194 26.729 -3.546 -13.732 1.00 7.45 C

ATOM 3079 C ALA A 194 26.262 -3.290 -12.290 1.00 7.03 C

ATOM 3080 O ALA A 194 25.117 -2.880 -12.104 1.00 7.39 O

ATOM 3081 CB ALA A 194 26.589 -5.027 -14.089 1.00 7.51 C

ATOM 3082 H ALA A 194 28.782 -3.868 -14.112 1.00 0.00 H

ATOM 3083 HA ALA A 194 26.078 -2.977 -14.399 1.00 0.00 H

ATOM 3084 HB1 ALA A 194 25.569 -5.383 -13.937 1.00 0.00 H

ATOM 3085 HB2 ALA A 194 26.842 -5.187 -15.137 1.00 0.00 H

ATOM 3086 HB3 ALA A 194 27.253 -5.649 -13.487 1.00 0.00 H

ATOM 3087 N ILE A 195 27.167 -3.488 -11.313 1.00 6.78 N

ATOM 3088 CA ILE A 195 26.987 -3.123 -9.905 1.00 6.46 C

ATOM 3089 C ILE A 195 26.746 -1.617 -9.722 1.00 6.35 C

ATOM 3090 O ILE A 195 25.799 -1.247 -9.030 1.00 6.61 O

ATOM 3091 CB ILE A 195 28.185 -3.598 -9.024 1.00 6.03 C

ATOM 3092 CG1 ILE A 195 28.087 -5.114 -8.749 1.00 6.22 C

ATOM 3093 CG2 ILE A 195 28.386 -2.842 -7.692 1.00 6.26 C

ATOM 3094 CD1 ILE A 195 29.411 -5.742 -8.291 1.00 6.74 C

ATOM 3095 H ILE A 195 28.077 -3.859 -11.554 1.00 0.00 H

ATOM 3096 HA ILE A 195 26.091 -3.625 -9.546 1.00 0.00 H

ATOM 3097 HB ILE A 195 29.093 -3.441 -9.604 1.00 0.00 H

ATOM 3098 HG13 ILE A 195 27.751 -5.640 -9.644 1.00 0.00 H

ATOM 3099 HG12 ILE A 195 27.319 -5.301 -7.998 1.00 0.00 H

ATOM 3100 HG21 ILE A 195 29.192 -3.278 -7.105 1.00 0.00 H

ATOM 3101 HG22 ILE A 195 28.660 -1.798 -7.843 1.00 0.00 H

ATOM 3102 HG23 ILE A 195 27.480 -2.873 -7.090 1.00 0.00 H

ATOM 3103 HD11 ILE A 195 29.376 -6.822 -8.415 1.00 0.00 H

ATOM 3104 HD12 ILE A 195 30.259 -5.373 -8.868 1.00 0.00 H

ATOM 3105 HD13 ILE A 195 29.609 -5.541 -7.238 1.00 0.00 H

ATOM 3106 N PHE A 196 27.575 -0.788 -10.378 1.00 6.63 N

ATOM 3107 CA PHE A 196 27.447 0.666 -10.363 1.00 6.79 C

ATOM 3108 C PHE A 196 26.130 1.140 -10.986 1.00 7.14 C

ATOM 3109 O PHE A 196 25.491 2.011 -10.413 1.00 7.82 O

ATOM 3110 CB PHE A 196 28.630 1.329 -11.093 1.00 0.00 C

ATOM 3111 CG PHE A 196 30.031 1.094 -10.554 1.00 0.00 C

ATOM 3112 CD1 PHE A 196 30.276 0.788 -9.196 1.00 0.00 C

ATOM 3113 CD2 PHE A 196 31.128 1.333 -11.407 1.00 0.00 C

ATOM 3114 CE1 PHE A 196 31.581 0.638 -8.747 1.00 0.00 C

ATOM 3115 CE2 PHE A 196 32.423 1.195 -10.932 1.00 0.00 C

ATOM 3116 CZ PHE A 196 32.649 0.841 -9.608 1.00 0.00 C

ATOM 3117 HB2 PHE A 196 28.625 1.029 -12.141 1.00 0.00 H

ATOM 3118 HB3 PHE A 196 28.478 2.403 -11.093 1.00 0.00 H

ATOM 3119 HD1 PHE A 196 29.465 0.643 -8.499 1.00 0.00 H

ATOM 3120 HD2 PHE A 196 30.965 1.617 -12.436 1.00 0.00 H

ATOM 3121 HE1 PHE A 196 31.766 0.371 -7.719 1.00 0.00 H

ATOM 3122 HE2 PHE A 196 33.255 1.362 -11.598 1.00 0.00 H

ATOM 3123 HZ PHE A 196 33.661 0.733 -9.244 1.00 0.00 H

ATOM 3124 H PHE A 196 28.343 -1.167 -10.919 1.00 0.00 H

ATOM 3125 HA PHE A 196 27.442 0.996 -9.323 1.00 0.00 H

ATOM 3126 N GLU A 197 25.727 0.554 -12.122 1.00 7.71 N

ATOM 3127 CA GLU A 197 24.528 0.967 -12.846 1.00 8.08 C

ATOM 3128 C GLU A 197 23.226 0.640 -12.087 1.00 7.57 C

ATOM 3129 O GLU A 197 22.326 1.479 -12.047 1.00 7.67 O

ATOM 3130 CB GLU A 197 24.565 0.325 -14.252 1.00 9.47 C

ATOM 3131 CG GLU A 197 23.659 1.004 -15.296 1.00 13.19 C

ATOM 3132 CD GLU A 197 24.053 2.456 -15.586 1.00 13.62 C

ATOM 3133 OE1 GLU A 197 23.129 3.241 -15.884 1.00 19.61 O

ATOM 3134 OE2 GLU A 197 25.255 2.785 -15.470 1.00 18.08 O1-

ATOM 3135 H GLU A 197 26.296 -0.161 -12.555 1.00 0.00 H

ATOM 3136 HA GLU A 197 24.588 2.052 -12.942 1.00 0.00 H

ATOM 3137 HB3 GLU A 197 24.288 -0.728 -14.181 1.00 0.00 H

ATOM 3138 HB2 GLU A 197 25.587 0.320 -14.633 1.00 0.00 H

ATOM 3139 HG3 GLU A 197 22.619 0.971 -14.970 1.00 0.00 H

ATOM 3140 HG2 GLU A 197 23.706 0.451 -16.235 1.00 0.00 H

ATOM 3141 N SER A 198 23.174 -0.561 -11.489 1.00 6.87 N

ATOM 3142 CA SER A 198 21.993 -1.118 -10.834 1.00 6.63 C

ATOM 3143 C SER A 198 21.825 -0.644 -9.378 1.00 6.33 C

ATOM 3144 O SER A 198 20.718 -0.264 -8.998 1.00 7.36 O

ATOM 3145 CB SER A 198 22.078 -2.657 -10.941 1.00 6.51 C

ATOM 3146 OG SER A 198 20.908 -3.300 -10.489 1.00 7.21 O

ATOM 3147 H SER A 198 23.970 -1.181 -11.563 1.00 0.00 H

ATOM 3148 HA SER A 198 21.114 -0.789 -11.390 1.00 0.00 H

ATOM 3149 HB3 SER A 198 22.928 -3.039 -10.374 1.00 0.00 H

ATOM 3150 HB2 SER A 198 22.234 -2.952 -11.980 1.00 0.00 H

ATOM 3151 HG SER A 198 20.200 -3.083 -11.109 1.00 0.00 H

ATOM 3152 N ALA A 199 22.913 -0.692 -8.589 1.00 6.32 N

ATOM 3153 CA ALA A 199 22.880 -0.461 -7.144 1.00 6.19 C

ATOM 3154 C ALA A 199 23.178 0.986 -6.731 1.00 6.52 C

ATOM 3155 O ALA A 199 22.694 1.396 -5.676 1.00 6.83 O

ATOM 3156 CB ALA A 199 23.870 -1.418 -6.468 1.00 6.43 C

ATOM 3157 H ALA A 199 23.801 -0.991 -8.971 1.00 0.00 H

ATOM 3158 HA ALA A 199 21.888 -0.698 -6.765 1.00 0.00 H

ATOM 3159 HB1 ALA A 199 23.695 -1.461 -5.394 1.00 0.00 H

ATOM 3160 HB2 ALA A 199 23.773 -2.433 -6.854 1.00 0.00 H

ATOM 3161 HB3 ALA A 199 24.901 -1.099 -6.622 1.00 0.00 H

ATOM 3162 N VAL A 200 23.962 1.724 -7.535 1.00 6.61 N

ATOM 3163 CA VAL A 200 24.366 3.097 -7.224 1.00 6.42 C

ATOM 3164 C VAL A 200 23.618 4.089 -8.130 1.00 6.54 C

ATOM 3165 O VAL A 200 22.963 4.992 -7.617 1.00 6.65 O

ATOM 3166 CB VAL A 200 25.904 3.315 -7.379 1.00 6.36 C

ATOM 3167 CG1 VAL A 200 26.366 4.757 -7.091 1.00 6.85 C

ATOM 3168 CG2 VAL A 200 26.702 2.345 -6.499 1.00 7.65 C

ATOM 3169 H VAL A 200 24.326 1.325 -8.389 1.00 0.00 H

ATOM 3170 HA VAL A 200 24.109 3.346 -6.193 1.00 0.00 H

ATOM 3171 HB VAL A 200 26.204 3.107 -8.400 1.00 0.00 H

ATOM 3172 HG11 VAL A 200 27.453 4.839 -7.129 1.00 0.00 H

ATOM 3173 HG12 VAL A 200 25.973 5.460 -7.822 1.00 0.00 H

ATOM 3174 HG13 VAL A 200 26.048 5.084 -6.102 1.00 0.00 H

ATOM 3175 HG21 VAL A 200 27.776 2.504 -6.599 1.00 0.00 H

ATOM 3176 HG22 VAL A 200 26.435 2.496 -5.456 1.00 0.00 H

ATOM 3177 HG23 VAL A 200 26.505 1.304 -6.758 1.00 0.00 H

ATOM 3178 N GLY A 201 23.732 3.901 -9.455 1.00 6.45 N

ATOM 3179 CA GLY A 201 23.289 4.847 -10.472 1.00 6.56 C

ATOM 3180 C GLY A 201 21.770 4.976 -10.534 1.00 6.93 C

ATOM 3181 O GLY A 201 21.283 6.085 -10.731 1.00 7.35 O

ATOM 3182 H GLY A 201 24.279 3.115 -9.787 1.00 0.00 H

ATOM 3183 HA3 GLY A 201 23.661 4.531 -11.447 1.00 0.00 H

ATOM 3184 HA2 GLY A 201 23.723 5.820 -10.250 1.00 0.00 H

ATOM 3185 N PHE A 202 21.031 3.874 -10.315 1.00 6.54 N

ATOM 3186 CA PHE A 202 19.569 3.863 -10.276 1.00 6.88 C

ATOM 3187 C PHE A 202 19.032 4.722 -9.116 1.00 6.79 C

ATOM 3188 O PHE A 202 18.093 5.480 -9.329 1.00 7.52 O

ATOM 3189 CB PHE A 202 19.078 2.403 -10.194 1.00 0.00 C

ATOM 3190 CG PHE A 202 17.624 2.181 -10.574 1.00 0.00 C

ATOM 3191 CD1 PHE A 202 17.246 2.221 -11.933 1.00 0.00 C

ATOM 3192 CD2 PHE A 202 16.627 2.018 -9.589 1.00 0.00 C

ATOM 3193 CE1 PHE A 202 15.920 2.025 -12.293 1.00 0.00 C

ATOM 3194 CE2 PHE A 202 15.305 1.820 -9.970 1.00 0.00 C

ATOM 3195 CZ PHE A 202 14.956 1.813 -11.316 1.00 0.00 C

ATOM 3196 HB2 PHE A 202 19.673 1.788 -10.872 1.00 0.00 H

ATOM 3197 HB3 PHE A 202 19.257 1.997 -9.198 1.00 0.00 H

ATOM 3198 HD1 PHE A 202 17.992 2.375 -12.699 1.00 0.00 H

ATOM 3199 HD2 PHE A 202 16.890 2.021 -8.541 1.00 0.00 H

ATOM 3200 HE1 PHE A 202 15.640 2.028 -13.335 1.00 0.00 H

ATOM 3201 HE2 PHE A 202 14.547 1.669 -9.216 1.00 0.00 H

ATOM 3202 HZ PHE A 202 13.927 1.653 -11.602 1.00 0.00 H

ATOM 3203 H PHE A 202 21.492 2.986 -10.172 1.00 0.00 H

ATOM 3204 HA PHE A 202 19.221 4.296 -11.216 1.00 0.00 H

ATOM 3205 N TRP A 203 19.675 4.648 -7.939 1.00 6.76 N

ATOM 3206 CA TRP A 203 19.291 5.421 -6.756 1.00 6.46 C

ATOM 3207 C TRP A 203 19.780 6.874 -6.759 1.00 6.37 C

ATOM 3208 O TRP A 203 19.093 7.714 -6.177 1.00 6.08 O

ATOM 3209 CB TRP A 203 19.647 4.633 -5.485 1.00 6.70 C

ATOM 3210 CG TRP A 203 18.830 3.382 -5.407 1.00 6.89 C

ATOM 3211 CD1 TRP A 203 19.220 2.150 -5.804 1.00 7.18 C

ATOM 3212 CD2 TRP A 203 17.401 3.281 -5.123 1.00 6.79 C

ATOM 3213 NE1 TRP A 203 18.133 1.303 -5.794 1.00 7.11 N

ATOM 3214 CE2 TRP A 203 16.981 1.945 -5.391 1.00 5.93 C

ATOM 3215 CE3 TRP A 203 16.405 4.198 -4.714 1.00 7.64 C

ATOM 3216 CZ2 TRP A 203 15.644 1.532 -5.249 1.00 6.38 C

ATOM 3217 CZ3 TRP A 203 15.060 3.801 -4.577 1.00 6.53 C

ATOM 3218 CH2 TRP A 203 14.678 2.472 -4.844 1.00 7.14 C

ATOM 3219 H TRP A 203 20.467 4.030 -7.837 1.00 0.00 H

ATOM 3220 HA TRP A 203 18.202 5.509 -6.761 1.00 0.00 H

ATOM 3221 HB3 TRP A 203 19.437 5.230 -4.597 1.00 0.00 H

ATOM 3222 HB2 TRP A 203 20.710 4.388 -5.461 1.00 0.00 H

ATOM 3223 HD1 TRP A 203 20.215 1.898 -6.138 1.00 0.00 H

ATOM 3224 HE1 TRP A 203 18.187 0.329 -6.069 1.00 0.00 H

ATOM 3225 HE3 TRP A 203 16.678 5.225 -4.517 1.00 0.00 H

ATOM 3226 HZ2 TRP A 203 15.362 0.512 -5.462 1.00 0.00 H

ATOM 3227 HZ3 TRP A 203 14.317 4.523 -4.272 1.00 0.00 H

ATOM 3228 HH2 TRP A 203 13.645 2.174 -4.737 1.00 0.00 H

ATOM 3229 N LEU A 204 20.877 7.176 -7.479 1.00 6.29 N

ATOM 3230 CA LEU A 204 21.285 8.550 -7.792 1.00 5.88 C

ATOM 3231 C LEU A 204 20.287 9.252 -8.734 1.00 6.23 C

ATOM 3232 O LEU A 204 20.013 10.434 -8.527 1.00 6.48 O

ATOM 3233 CB LEU A 204 22.708 8.578 -8.392 1.00 5.86 C

ATOM 3234 CG LEU A 204 23.842 8.312 -7.379 1.00 6.55 C

ATOM 3235 CD1 LEU A 204 25.187 8.175 -8.107 1.00 7.81 C

ATOM 3236 CD2 LEU A 204 23.929 9.377 -6.269 1.00 7.01 C

ATOM 3237 H LEU A 204 21.407 6.436 -7.920 1.00 0.00 H

ATOM 3238 HA LEU A 204 21.288 9.114 -6.859 1.00 0.00 H

ATOM 3239 HB3 LEU A 204 22.895 9.549 -8.855 1.00 0.00 H

ATOM 3240 HB2 LEU A 204 22.762 7.854 -9.206 1.00 0.00 H

ATOM 3241 HG LEU A 204 23.647 7.356 -6.897 1.00 0.00 H

ATOM 3242 HD11 LEU A 204 25.946 7.769 -7.439 1.00 0.00 H

ATOM 3243 HD12 LEU A 204 25.113 7.508 -8.966 1.00 0.00 H

ATOM 3244 HD13 LEU A 204 25.544 9.140 -8.468 1.00 0.00 H

ATOM 3245 HD21 LEU A 204 24.956 9.704 -6.102 1.00 0.00 H

ATOM 3246 HD22 LEU A 204 23.346 10.266 -6.507 1.00 0.00 H

ATOM 3247 HD23 LEU A 204 23.560 8.986 -5.321 1.00 0.00 H

ATOM 3248 N ASP A 205 19.722 8.511 -9.705 1.00 6.14 N

ATOM 3249 CA ASP A 205 18.644 8.976 -10.590 1.00 7.00 C

ATOM 3250 C ASP A 205 17.304 9.205 -9.866 1.00 6.93 C

ATOM 3251 O ASP A 205 16.508 10.006 -10.354 1.00 8.32 O

ATOM 3252 CB ASP A 205 18.423 8.075 -11.831 1.00 6.52 C

ATOM 3253 CG ASP A 205 19.658 7.850 -12.708 1.00 7.24 C

ATOM 3254 OD1 ASP A 205 20.602 8.668 -12.634 1.00 7.64 O

ATOM 3255 OD2 ASP A 205 19.616 6.892 -13.509 1.00 9.22 O1-

ATOM 3256 H ASP A 205 20.018 7.552 -9.836 1.00 0.00 H

ATOM 3257 HA ASP A 205 18.951 9.957 -10.961 1.00 0.00 H

ATOM 3258 HB3 ASP A 205 17.640 8.500 -12.462 1.00 0.00 H

ATOM 3259 HB2 ASP A 205 18.075 7.099 -11.491 1.00 0.00 H

ATOM 3260 N HIS A 206 17.092 8.537 -8.718 1.00 7.20 N

ATOM 3261 CA HIS A 206 15.910 8.695 -7.864 1.00 7.54 C

ATOM 3262 C HIS A 206 16.179 9.551 -6.609 1.00 7.60 C

ATOM 3263 O HIS A 206 15.407 9.484 -5.652 1.00 8.75 O

ATOM 3264 CB HIS A 206 15.279 7.315 -7.584 1.00 7.77 C

ATOM 3265 CG HIS A 206 14.710 6.685 -8.835 1.00 8.14 C

ATOM 3266 ND1 HIS A 206 15.344 5.671 -9.528 1.00 10.52 N

ATOM 3267 CD2 HIS A 206 13.557 6.941 -9.544 1.00 9.30 C

ATOM 3268 CE1 HIS A 206 14.597 5.383 -10.594 1.00 10.31 C

ATOM 3269 NE2 HIS A 206 13.490 6.109 -10.665 1.00 11.29 N

ATOM 3270 H HIS A 206 17.794 7.889 -8.389 1.00 0.00 H

ATOM 3271 HA HIS A 206 15.151 9.266 -8.402 1.00 0.00 H

ATOM 3272 HB3 HIS A 206 14.463 7.400 -6.866 1.00 0.00 H

ATOM 3273 HB2 HIS A 206 16.012 6.642 -7.139 1.00 0.00 H

ATOM 3274 HD1 HIS A 206 16.256 5.281 -9.315 1.00 0.00 H

ATOM 3275 HD2 HIS A 206 12.778 7.661 -9.339 1.00 0.00 H

ATOM 3276 HE1 HIS A 206 14.872 4.644 -11.332 1.00 0.00 H

ATOM 3277 N GLY A 207 17.213 10.410 -6.677 1.00 7.60 N

ATOM 3278 CA GLY A 207 17.378 11.570 -5.802 1.00 7.41 C

ATOM 3279 C GLY A 207 18.021 11.272 -4.441 1.00 7.49 C

ATOM 3280 O GLY A 207 17.958 12.144 -3.575 1.00 7.55 O

ATOM 3281 H GLY A 207 17.810 10.378 -7.492 1.00 0.00 H

ATOM 3282 HA3 GLY A 207 18.003 12.297 -6.321 1.00 0.00 H

ATOM 3283 HA2 GLY A 207 16.413 12.054 -5.642 1.00 0.00 H

ATOM 3284 N VAL A 208 18.634 10.092 -4.231 1.00 6.50 N

ATOM 3285 CA VAL A 208 19.281 9.732 -2.962 1.00 6.06 C

ATOM 3286 C VAL A 208 20.488 10.646 -2.645 1.00 5.82 C

ATOM 3287 O VAL A 208 21.288 10.930 -3.536 1.00 5.67 O

ATOM 3288 CB VAL A 208 19.722 8.236 -2.947 1.00 6.04 C

ATOM 3289 CG1 VAL A 208 21.085 7.911 -3.596 1.00 6.52 C

ATOM 3290 CG2 VAL A 208 19.688 7.670 -1.525 1.00 6.16 C

ATOM 3291 H VAL A 208 18.659 9.396 -4.965 1.00 0.00 H

ATOM 3292 HA VAL A 208 18.532 9.877 -2.180 1.00 0.00 H

ATOM 3293 HB VAL A 208 18.974 7.678 -3.512 1.00 0.00 H

ATOM 3294 HG11 VAL A 208 21.201 6.835 -3.720 1.00 0.00 H

ATOM 3295 HG12 VAL A 208 21.179 8.365 -4.581 1.00 0.00 H

ATOM 3296 HG13 VAL A 208 21.924 8.253 -2.989 1.00 0.00 H

ATOM 3297 HG21 VAL A 208 20.001 6.630 -1.505 1.00 0.00 H

ATOM 3298 HG22 VAL A 208 20.338 8.230 -0.856 1.00 0.00 H

ATOM 3299 HG23 VAL A 208 18.676 7.709 -1.130 1.00 0.00 H

ATOM 3300 N ASP A 209 20.585 11.097 -1.387 1.00 5.24 N

ATOM 3301 CA ASP A 209 21.655 11.974 -0.893 1.00 5.33 C

ATOM 3302 C ASP A 209 22.899 11.215 -0.424 1.00 5.33 C

ATOM 3303 O ASP A 209 23.870 11.868 -0.043 1.00 5.55 O

ATOM 3304 CB ASP A 209 21.197 12.915 0.238 1.00 5.07 C

ATOM 3305 CG ASP A 209 19.912 13.661 -0.081 1.00 6.35 C

ATOM 3306 OD1 ASP A 209 19.966 14.554 -0.955 1.00 9.16 O

ATOM 3307 OD2 ASP A 209 18.908 13.367 0.597 1.00 5.85 O1-

ATOM 3308 H ASP A 209 19.899 10.816 -0.698 1.00 0.00 H

ATOM 3309 HA ASP A 209 21.980 12.612 -1.717 1.00 0.00 H

ATOM 3310 HB3 ASP A 209 21.056 12.357 1.166 1.00 0.00 H

ATOM 3311 HB2 ASP A 209 21.964 13.653 0.472 1.00 0.00 H

ATOM 3312 N GLY A 210 22.879 9.877 -0.434 1.00 5.64 N

ATOM 3313 CA GLY A 210 24.043 9.143 0.014 1.00 5.85 C

ATOM 3314 C GLY A 210 23.803 7.647 0.055 1.00 5.43 C

ATOM 3315 O GLY A 210 22.729 7.145 -0.283 1.00 5.15 O

ATOM 3316 H GLY A 210 22.068 9.360 -0.741 1.00 0.00 H

ATOM 3317 HA3 GLY A 210 24.338 9.480 1.009 1.00 0.00 H

ATOM 3318 HA2 GLY A 210 24.863 9.333 -0.669 1.00 0.00 H

ATOM 3319 N PHE A 211 24.858 6.952 0.497 1.00 5.02 N

ATOM 3320 CA PHE A 211 24.884 5.511 0.669 1.00 5.13 C

ATOM 3321 C PHE A 211 25.551 5.165 2.000 1.00 5.26 C

ATOM 3322 O PHE A 211 26.642 5.661 2.274 1.00 5.90 O

ATOM 3323 CB PHE A 211 25.644 4.844 -0.496 1.00 5.10 C

ATOM 3324 CG PHE A 211 25.017 5.042 -1.864 1.00 5.32 C

ATOM 3325 CD1 PHE A 211 23.870 4.308 -2.232 1.00 5.67 C

ATOM 3326 CD2 PHE A 211 25.465 6.082 -2.708 1.00 5.22 C

ATOM 3327 CE1 PHE A 211 23.242 4.560 -3.442 1.00 6.96 C

ATOM 3328 CE2 PHE A 211 24.813 6.329 -3.908 1.00 6.06 C

ATOM 3329 CZ PHE A 211 23.712 5.567 -4.275 1.00 6.35 C

ATOM 3330 H PHE A 211 25.702 7.444 0.757 1.00 0.00 H

ATOM 3331 HA PHE A 211 23.866 5.142 0.680 1.00 0.00 H

ATOM 3332 HB3 PHE A 211 25.703 3.772 -0.308 1.00 0.00 H

ATOM 3333 HB2 PHE A 211 26.677 5.192 -0.527 1.00 0.00 H

ATOM 3334 HD1 PHE A 211 23.469 3.556 -1.570 1.00 0.00 H

ATOM 3335 HD2 PHE A 211 26.307 6.693 -2.420 1.00 0.00 H

ATOM 3336 HE1 PHE A 211 22.373 3.985 -3.727 1.00 0.00 H

ATOM 3337 HE2 PHE A 211 25.159 7.122 -4.555 1.00 0.00 H

ATOM 3338 HZ PHE A 211 23.201 5.772 -5.202 1.00 0.00 H

ATOM 3339 N ARG A 212 24.935 4.250 2.762 1.00 4.17 N

ATOM 3340 CA ARG A 212 25.653 3.479 3.772 1.00 4.03 C

ATOM 3341 C ARG A 212 26.112 2.191 3.100 1.00 4.29 C

ATOM 3342 O ARG A 212 25.274 1.418 2.654 1.00 4.79 O

ATOM 3343 CB ARG A 212 24.783 3.246 5.019 1.00 4.61 C

ATOM 3344 CG ARG A 212 25.472 2.427 6.133 1.00 2.81 C

ATOM 3345 CD ARG A 212 25.395 0.906 5.947 1.00 4.05 C

ATOM 3346 NE ARG A 212 25.789 0.189 7.159 1.00 4.39 N

ATOM 3347 CZ ARG A 212 25.717 -1.139 7.323 1.00 4.25 C

ATOM 3348 NH1 ARG A 212 25.300 -1.947 6.339 1.00 3.62 N

ATOM 3349 NH2 ARG A 212 26.070 -1.652 8.504 1.00 4.67 N1+

ATOM 3350 H ARG A 212 24.035 3.886 2.477 1.00 0.00 H

ATOM 3351 HA ARG A 212 26.533 4.027 4.093 1.00 0.00 H

ATOM 3352 HB3 ARG A 212 23.825 2.797 4.756 1.00 0.00 H

ATOM 3353 HB2 ARG A 212 24.545 4.227 5.430 1.00 0.00 H

ATOM 3354 HG3 ARG A 212 25.065 2.698 7.105 1.00 0.00 H

ATOM 3355 HG2 ARG A 212 26.522 2.713 6.165 1.00 0.00 H

ATOM 3356 HD3 ARG A 212 26.130 0.570 5.219 1.00 0.00 H

ATOM 3357 HD2 ARG A 212 24.415 0.612 5.589 1.00 0.00 H

ATOM 3358 HE ARG A 212 26.118 0.754 7.927 1.00 0.00 H

ATOM 3359 HH12 ARG A 212 25.374 -2.956 6.426 1.00 0.00 H

ATOM 3360 HH11 ARG A 212 24.965 -1.557 5.465 1.00 0.00 H

ATOM 3361 HH22 ARG A 212 26.137 -2.657 8.625 1.00 0.00 H

ATOM 3362 HH21 ARG A 212 26.386 -1.059 9.255 1.00 0.00 H

ATOM 3363 N ILE A 213 27.429 1.992 3.023 1.00 4.05 N

ATOM 3364 CA ILE A 213 28.018 0.942 2.210 1.00 4.38 C

ATOM 3365 C ILE A 213 28.370 -0.262 3.103 1.00 4.76 C

ATOM 3366 O ILE A 213 29.358 -0.213 3.835 1.00 5.43 O

ATOM 3367 CB ILE A 213 29.263 1.469 1.445 1.00 4.66 C

ATOM 3368 CG1 ILE A 213 28.942 2.779 0.676 1.00 3.78 C

ATOM 3369 CG2 ILE A 213 29.799 0.397 0.489 1.00 4.46 C

ATOM 3370 CD1 ILE A 213 30.094 3.331 -0.174 1.00 5.31 C

ATOM 3371 H ILE A 213 28.069 2.649 3.452 1.00 0.00 H

ATOM 3372 HA ILE A 213 27.305 0.607 1.459 1.00 0.00 H

ATOM 3373 HB ILE A 213 30.052 1.699 2.156 1.00 0.00 H

ATOM 3374 HG13 ILE A 213 28.647 3.563 1.375 1.00 0.00 H

ATOM 3375 HG12 ILE A 213 28.077 2.620 0.033 1.00 0.00 H

ATOM 3376 HG21 ILE A 213 30.718 0.718 0.002 1.00 0.00 H

ATOM 3377 HG22 ILE A 213 30.033 -0.537 0.999 1.00 0.00 H

ATOM 3378 HG23 ILE A 213 29.066 0.171 -0.286 1.00 0.00 H

ATOM 3379 HD11 ILE A 213 29.870 4.340 -0.518 1.00 0.00 H

ATOM 3380 HD12 ILE A 213 31.026 3.368 0.391 1.00 0.00 H

ATOM 3381 HD13 ILE A 213 30.259 2.726 -1.065 1.00 0.00 H

ATOM 3382 N ASP A 214 27.526 -1.308 3.027 1.00 4.19 N

ATOM 3383 CA ASP A 214 27.647 -2.568 3.765 1.00 4.56 C

ATOM 3384 C ASP A 214 28.930 -3.316 3.382 1.00 5.11 C

ATOM 3385 O ASP A 214 29.148 -3.562 2.193 1.00 5.28 O

ATOM 3386 CB ASP A 214 26.416 -3.471 3.538 1.00 5.06 C

ATOM 3387 CG ASP A 214 26.420 -4.781 4.334 1.00 5.64 C

ATOM 3388 OD1 ASP A 214 26.018 -4.721 5.516 1.00 5.28 O

ATOM 3389 OD2 ASP A 214 26.790 -5.818 3.739 1.00 5.91 O1-

ATOM 3390 H ASP A 214 26.746 -1.258 2.385 1.00 0.00 H

ATOM 3391 HA ASP A 214 27.686 -2.306 4.823 1.00 0.00 H

ATOM 3392 HB3 ASP A 214 26.325 -3.721 2.487 1.00 0.00 H

ATOM 3393 HB2 ASP A 214 25.498 -2.933 3.763 1.00 0.00 H

ATOM 3394 N THR A 215 29.718 -3.667 4.415 1.00 4.59 N

ATOM 3395 CA THR A 215 30.976 -4.422 4.384 1.00 5.13 C

ATOM 3396 C THR A 215 31.903 -4.009 3.218 1.00 5.12 C

ATOM 3397 O THR A 215 32.387 -4.838 2.446 1.00 5.35 O

ATOM 3398 CB THR A 215 30.738 -5.946 4.484 1.00 0.00 C

ATOM 3399 OG1 THR A 215 29.996 -6.473 3.400 1.00 0.00 O

ATOM 3400 CG2 THR A 215 30.027 -6.306 5.797 1.00 0.00 C

ATOM 3401 HB THR A 215 31.706 -6.440 4.485 1.00 0.00 H

ATOM 3402 HG1 THR A 215 29.085 -6.172 3.482 1.00 0.00 H

ATOM 3403 HG21 THR A 215 29.900 -7.377 5.900 1.00 0.00 H

ATOM 3404 HG22 THR A 215 30.585 -5.955 6.665 1.00 0.00 H

ATOM 3405 HG23 THR A 215 29.028 -5.874 5.843 1.00 0.00 H

ATOM 3406 H THR A 215 29.377 -3.465 5.350 1.00 0.00 H

ATOM 3407 HA THR A 215 31.511 -4.170 5.293 1.00 0.00 H

ATOM 3408 N ALA A 216 32.089 -2.679 3.133 1.00 4.63 N

ATOM 3409 CA ALA A 216 32.806 -1.934 2.102 1.00 5.25 C

ATOM 3410 C ALA A 216 34.265 -2.366 1.899 1.00 5.08 C

ATOM 3411 O ALA A 216 34.761 -2.275 0.779 1.00 5.93 O

ATOM 3412 CB ALA A 216 32.747 -0.445 2.479 1.00 0.00 C

ATOM 3413 HB1 ALA A 216 33.243 0.182 1.741 1.00 0.00 H

ATOM 3414 HB2 ALA A 216 31.720 -0.097 2.564 1.00 0.00 H

ATOM 3415 HB3 ALA A 216 33.227 -0.260 3.441 1.00 0.00 H

ATOM 3416 H ALA A 216 31.641 -2.107 3.834 1.00 0.00 H

ATOM 3417 HA ALA A 216 32.274 -2.079 1.161 1.00 0.00 H

ATOM 3418 N GLY A 217 34.910 -2.837 2.979 1.00 4.93 N

ATOM 3419 CA GLY A 217 36.306 -3.262 2.993 1.00 5.66 C

ATOM 3420 C GLY A 217 36.525 -4.676 2.424 1.00 5.69 C

ATOM 3421 O GLY A 217 37.680 -5.086 2.341 1.00 5.55 O

ATOM 3422 HA3 GLY A 217 36.658 -3.245 4.025 1.00 0.00 H

ATOM 3423 H GLY A 217 34.410 -2.890 3.855 1.00 0.00 H

ATOM 3424 HA2 GLY A 217 36.919 -2.545 2.446 1.00 0.00 H

ATOM 3425 N LEU A 218 35.468 -5.421 2.046 1.00 5.70 N

ATOM 3426 CA LEU A 218 35.562 -6.828 1.636 1.00 5.60 C

ATOM 3427 C LEU A 218 35.647 -7.073 0.117 1.00 5.25 C

ATOM 3428 O LEU A 218 35.827 -8.232 -0.259 1.00 5.34 O

ATOM 3429 CB LEU A 218 34.381 -7.627 2.237 1.00 5.72 C

ATOM 3430 CG LEU A 218 34.352 -7.736 3.775 1.00 5.54 C

ATOM 3431 CD1 LEU A 218 33.185 -8.634 4.218 1.00 5.82 C

ATOM 3432 CD2 LEU A 218 35.675 -8.234 4.380 1.00 6.47 C

ATOM 3433 H LEU A 218 34.533 -5.041 2.122 1.00 0.00 H

ATOM 3434 HA LEU A 218 36.481 -7.248 2.033 1.00 0.00 H

ATOM 3435 HB3 LEU A 218 34.394 -8.645 1.843 1.00 0.00 H

ATOM 3436 HB2 LEU A 218 33.444 -7.189 1.894 1.00 0.00 H

ATOM 3437 HG LEU A 218 34.162 -6.738 4.172 1.00 0.00 H

ATOM 3438 HD11 LEU A 218 32.830 -8.357 5.211 1.00 0.00 H

ATOM 3439 HD12 LEU A 218 32.340 -8.560 3.534 1.00 0.00 H

ATOM 3440 HD13 LEU A 218 33.476 -9.684 4.252 1.00 0.00 H

ATOM 3441 HD21 LEU A 218 35.508 -8.936 5.196 1.00 0.00 H

ATOM 3442 HD22 LEU A 218 36.301 -8.746 3.652 1.00 0.00 H

ATOM 3443 HD23 LEU A 218 36.248 -7.398 4.778 1.00 0.00 H

ATOM 3444 N TYR A 219 35.506 -6.038 -0.733 1.00 5.33 N

ATOM 3445 CA TYR A 219 35.412 -6.183 -2.199 1.00 5.35 C

ATOM 3446 C TYR A 219 36.636 -6.833 -2.865 1.00 5.29 C

ATOM 3447 O TYR A 219 36.461 -7.608 -3.805 1.00 6.05 O

ATOM 3448 CB TYR A 219 35.180 -4.817 -2.872 1.00 5.18 C

ATOM 3449 CG TYR A 219 33.987 -3.989 -2.439 1.00 4.97 C

ATOM 3450 CD1 TYR A 219 32.886 -4.558 -1.761 1.00 5.73 C

ATOM 3451 CD2 TYR A 219 33.982 -2.615 -2.754 1.00 4.73 C

ATOM 3452 CE1 TYR A 219 31.798 -3.751 -1.388 1.00 6.12 C

ATOM 3453 CE2 TYR A 219 32.886 -1.812 -2.396 1.00 5.85 C

ATOM 3454 CZ TYR A 219 31.800 -2.384 -1.712 1.00 5.46 C

ATOM 3455 OH TYR A 219 30.739 -1.610 -1.369 1.00 6.02 O

ATOM 3456 H TYR A 219 35.360 -5.109 -0.364 1.00 0.00 H

ATOM 3457 HA TYR A 219 34.556 -6.826 -2.413 1.00 0.00 H

ATOM 3458 HB3 TYR A 219 35.094 -4.954 -3.950 1.00 0.00 H

ATOM 3459 HB2 TYR A 219 36.065 -4.198 -2.729 1.00 0.00 H

ATOM 3460 HD1 TYR A 219 32.863 -5.608 -1.514 1.00 0.00 H

ATOM 3461 HD2 TYR A 219 34.814 -2.173 -3.283 1.00 0.00 H

ATOM 3462 HE1 TYR A 219 30.961 -4.182 -0.859 1.00 0.00 H

ATOM 3463 HE2 TYR A 219 32.880 -0.762 -2.649 1.00 0.00 H

ATOM 3464 HH TYR A 219 30.069 -2.086 -0.874 1.00 0.00 H

ATOM 3465 N SER A 220 37.837 -6.495 -2.371 1.00 5.35 N

ATOM 3466 CA SER A 220 39.101 -6.993 -2.894 1.00 5.77 C

ATOM 3467 C SER A 220 39.525 -8.247 -2.126 1.00 5.99 C

ATOM 3468 O SER A 220 39.819 -8.171 -0.933 1.00 6.97 O

ATOM 3469 CB SER A 220 40.152 -5.870 -2.834 1.00 6.26 C

ATOM 3470 OG SER A 220 41.395 -6.315 -3.340 1.00 6.12 O

ATOM 3471 H SER A 220 37.893 -5.880 -1.572 1.00 0.00 H

ATOM 3472 HA SER A 220 38.981 -7.260 -3.946 1.00 0.00 H

ATOM 3473 HB3 SER A 220 40.288 -5.514 -1.813 1.00 0.00 H

ATOM 3474 HB2 SER A 220 39.823 -5.016 -3.425 1.00 0.00 H

ATOM 3475 HG SER A 220 41.273 -6.593 -4.252 1.00 0.00 H

ATOM 3476 N LYS A 221 39.566 -9.368 -2.856 1.00 5.87 N

ATOM 3477 CA LYS A 221 40.120 -10.632 -2.395 1.00 5.99 C

ATOM 3478 C LYS A 221 41.561 -10.749 -2.914 1.00 5.96 C

ATOM 3479 O LYS A 221 41.814 -10.399 -4.069 1.00 6.42 O

ATOM 3480 CB LYS A 221 39.253 -11.794 -2.926 1.00 6.46 C

ATOM 3481 CG LYS A 221 37.939 -12.061 -2.165 1.00 6.02 C

ATOM 3482 CD LYS A 221 36.879 -10.950 -2.259 1.00 6.26 C

ATOM 3483 CE LYS A 221 35.519 -11.406 -1.711 1.00 6.91 C

ATOM 3484 NZ LYS A 221 34.497 -10.353 -1.818 1.00 6.80 N1+

ATOM 3485 H LYS A 221 39.312 -9.333 -3.834 1.00 0.00 H

ATOM 3486 HA LYS A 221 40.121 -10.666 -1.305 1.00 0.00 H

ATOM 3487 HB3 LYS A 221 39.843 -12.704 -2.831 1.00 0.00 H

ATOM 3488 HB2 LYS A 221 39.062 -11.679 -3.995 1.00 0.00 H

ATOM 3489 HG3 LYS A 221 38.165 -12.253 -1.114 1.00 0.00 H

ATOM 3490 HG2 LYS A 221 37.511 -12.989 -2.547 1.00 0.00 H

ATOM 3491 HD3 LYS A 221 36.779 -10.609 -3.290 1.00 0.00 H

ATOM 3492 HD2 LYS A 221 37.212 -10.088 -1.682 1.00 0.00 H

ATOM 3493 HE3 LYS A 221 35.617 -11.692 -0.665 1.00 0.00 H

ATOM 3494 HE2 LYS A 221 35.165 -12.282 -2.252 1.00 0.00 H

ATOM 3495 HZ1 LYS A 221 34.795 -9.538 -1.295 1.00 0.00 H

ATOM 3496 HZ2 LYS A 221 34.350 -10.100 -2.784 1.00 0.00 H

ATOM 3497 HZ3 LYS A 221 33.632 -10.700 -1.419 1.00 0.00 H

ATOM 3498 N ARG A 222 42.472 -11.278 -2.076 1.00 6.31 N

ATOM 3499 CA ARG A 222 43.836 -11.631 -2.488 1.00 6.70 C

ATOM 3500 C ARG A 222 43.794 -12.793 -3.511 1.00 7.07 C

ATOM 3501 O ARG A 222 43.113 -13.784 -3.232 1.00 7.43 O

ATOM 3502 CB ARG A 222 44.676 -12.043 -1.256 1.00 0.00 C

ATOM 3503 CG ARG A 222 45.518 -10.888 -0.684 1.00 0.00 C

ATOM 3504 CD ARG A 222 46.497 -11.362 0.402 1.00 0.00 C

ATOM 3505 NE ARG A 222 47.527 -10.360 0.712 1.00 0.00 N

ATOM 3506 CZ ARG A 222 48.763 -10.318 0.184 1.00 0.00 C

ATOM 3507 NH1 ARG A 222 49.181 -11.245 -0.690 1.00 0.00 N

ATOM 3508 NH2 ARG A 222 49.597 -9.333 0.540 1.00 0.00 N1+

ATOM 3509 HB2 ARG A 222 44.059 -12.516 -0.491 1.00 0.00 H

ATOM 3510 HB3 ARG A 222 45.390 -12.810 -1.563 1.00 0.00 H

ATOM 3511 HG2 ARG A 222 46.059 -10.346 -1.462 1.00 0.00 H

ATOM 3512 HG3 ARG A 222 44.814 -10.179 -0.245 1.00 0.00 H

ATOM 3513 HD2 ARG A 222 45.935 -11.396 1.333 1.00 0.00 H

ATOM 3514 HD3 ARG A 222 46.874 -12.374 0.256 1.00 0.00 H

ATOM 3515 HE ARG A 222 47.248 -9.631 1.355 1.00 0.00 H

ATOM 3516 HH12 ARG A 222 50.113 -11.206 -1.078 1.00 0.00 H

ATOM 3517 HH11 ARG A 222 48.562 -11.994 -0.965 1.00 0.00 H

ATOM 3518 HH22 ARG A 222 50.530 -9.286 0.156 1.00 0.00 H

ATOM 3519 HH21 ARG A 222 49.303 -8.630 1.203 1.00 0.00 H

ATOM 3520 H ARG A 222 42.195 -11.547 -1.141 1.00 0.00 H

ATOM 3521 HA ARG A 222 44.261 -10.717 -2.896 1.00 0.00 H

ATOM 3522 N PRO A 223 44.507 -12.654 -4.659 1.00 7.25 N

ATOM 3523 CA PRO A 223 44.618 -13.700 -5.699 1.00 8.13 C

ATOM 3524 C PRO A 223 44.934 -15.122 -5.203 1.00 7.74 C

ATOM 3525 O PRO A 223 45.783 -15.298 -4.327 1.00 8.25 O

ATOM 3526 CD PRO A 223 45.257 -11.457 -5.059 1.00 0.00 C

ATOM 3527 CB PRO A 223 45.735 -13.182 -6.618 1.00 0.00 C

ATOM 3528 CG PRO A 223 45.631 -11.675 -6.517 1.00 0.00 C

ATOM 3529 HD3 PRO A 223 44.679 -10.535 -4.972 1.00 0.00 H

ATOM 3530 HD2 PRO A 223 46.151 -11.373 -4.438 1.00 0.00 H

ATOM 3531 HB2 PRO A 223 46.716 -13.499 -6.256 1.00 0.00 H

ATOM 3532 HB3 PRO A 223 45.634 -13.535 -7.645 1.00 0.00 H

ATOM 3533 HG3 PRO A 223 44.816 -11.329 -7.156 1.00 0.00 H

ATOM 3534 HG2 PRO A 223 46.539 -11.152 -6.817 1.00 0.00 H

ATOM 3535 HA PRO A 223 43.672 -13.698 -6.244 1.00 0.00 H

ATOM 3536 N GLY A 224 44.217 -16.096 -5.783 1.00 8.01 N

ATOM 3537 CA GLY A 224 44.349 -17.523 -5.497 1.00 8.02 C

ATOM 3538 C GLY A 224 43.452 -17.978 -4.335 1.00 8.40 C

ATOM 3539 O GLY A 224 43.284 -19.185 -4.166 1.00 8.97 O

ATOM 3540 H GLY A 224 43.540 -15.843 -6.489 1.00 0.00 H

ATOM 3541 HA3 GLY A 224 45.386 -17.780 -5.272 1.00 0.00 H

ATOM 3542 HA2 GLY A 224 44.073 -18.081 -6.392 1.00 0.00 H

ATOM 3543 N LEU A 225 42.883 -17.038 -3.552 1.00 8.10 N

ATOM 3544 CA LEU A 225 42.051 -17.255 -2.361 1.00 7.98 C

ATOM 3545 C LEU A 225 42.710 -18.242 -1.363 1.00 8.28 C

ATOM 3546 O LEU A 225 42.158 -19.324 -1.151 1.00 8.67 O

ATOM 3547 CB LEU A 225 40.619 -17.693 -2.776 1.00 8.03 C

ATOM 3548 CG LEU A 225 39.880 -16.792 -3.796 1.00 7.86 C

ATOM 3549 CD1 LEU A 225 38.548 -17.427 -4.234 1.00 8.30 C

ATOM 3550 CD2 LEU A 225 39.661 -15.355 -3.303 1.00 10.02 C

ATOM 3551 H LEU A 225 43.074 -16.068 -3.763 1.00 0.00 H

ATOM 3552 HA LEU A 225 41.981 -16.300 -1.840 1.00 0.00 H

ATOM 3553 HB3 LEU A 225 40.001 -17.782 -1.882 1.00 0.00 H

ATOM 3554 HB2 LEU A 225 40.675 -18.693 -3.204 1.00 0.00 H

ATOM 3555 HG LEU A 225 40.503 -16.731 -4.688 1.00 0.00 H

ATOM 3556 HD11 LEU A 225 38.519 -17.568 -5.314 1.00 0.00 H

ATOM 3557 HD12 LEU A 225 38.411 -18.403 -3.773 1.00 0.00 H

ATOM 3558 HD13 LEU A 225 37.685 -16.817 -3.964 1.00 0.00 H

ATOM 3559 HD21 LEU A 225 39.414 -14.702 -4.141 1.00 0.00 H

ATOM 3560 HD22 LEU A 225 38.841 -15.295 -2.587 1.00 0.00 H

ATOM 3561 HD23 LEU A 225 40.549 -14.942 -2.829 1.00 0.00 H

ATOM 3562 N PRO A 226 43.928 -17.925 -0.859 1.00 8.95 N

ATOM 3563 CA PRO A 226 44.747 -18.898 -0.108 1.00 9.18 C

ATOM 3564 C PRO A 226 44.123 -19.286 1.239 1.00 9.36 C

ATOM 3565 O PRO A 226 43.458 -18.443 1.841 1.00 9.09 O

ATOM 3566 CB PRO A 226 46.092 -18.168 0.104 1.00 9.09 C

ATOM 3567 CG PRO A 226 46.083 -16.995 -0.862 1.00 9.75 C

ATOM 3568 CD PRO A 226 44.611 -16.631 -0.937 1.00 9.85 C

ATOM 3569 HA PRO A 226 44.894 -19.782 -0.733 1.00 0.00 H

ATOM 3570 HB3 PRO A 226 46.950 -18.822 -0.057 1.00 0.00 H

ATOM 3571 HB2 PRO A 226 46.172 -17.781 1.122 1.00 0.00 H

ATOM 3572 HG3 PRO A 226 46.421 -17.328 -1.845 1.00 0.00 H

ATOM 3573 HG2 PRO A 226 46.721 -16.168 -0.548 1.00 0.00 H

ATOM 3574 HD2 PRO A 226 44.319 -16.037 -0.070 1.00 0.00 H

ATOM 3575 HD3 PRO A 226 44.411 -16.034 -1.824 1.00 0.00 H

ATOM 3576 N ASP A 227 44.395 -20.519 1.707 1.00 9.45 N

ATOM 3577 CA ASP A 227 44.123 -20.957 3.085 1.00 9.60 C

ATOM 3578 C ASP A 227 44.663 -19.962 4.119 1.00 9.64 C

ATOM 3579 O ASP A 227 45.860 -19.670 4.125 1.00 10.34 O

ATOM 3580 CB ASP A 227 44.665 -22.368 3.408 1.00 9.78 C

ATOM 3581 CG ASP A 227 43.982 -23.489 2.630 1.00 11.66 C

ATOM 3582 OD1 ASP A 227 44.699 -24.244 1.940 1.00 14.29 O

ATOM 3583 OD2 ASP A 227 42.754 -23.621 2.815 1.00 11.06 O1-

ATOM 3584 H ASP A 227 44.937 -21.158 1.143 1.00 0.00 H

ATOM 3585 HA ASP A 227 43.036 -20.983 3.182 1.00 0.00 H

ATOM 3586 HB3 ASP A 227 44.586 -22.599 4.471 1.00 0.00 H

ATOM 3587 HB2 ASP A 227 45.733 -22.399 3.185 1.00 0.00 H

ATOM 3588 N SER A 228 43.743 -19.444 4.944 1.00 9.92 N

ATOM 3589 CA SER A 228 44.030 -18.490 6.004 1.00 10.68 C

ATOM 3590 C SER A 228 44.859 -19.130 7.135 1.00 11.32 C

ATOM 3591 O SER A 228 44.721 -20.336 7.357 1.00 10.62 O

ATOM 3592 CB SER A 228 42.704 -17.915 6.544 1.00 0.00 C

ATOM 3593 OG SER A 228 42.491 -16.637 5.988 1.00 0.00 O

ATOM 3594 HB2 SER A 228 41.852 -18.561 6.328 1.00 0.00 H

ATOM 3595 HB3 SER A 228 42.727 -17.793 7.628 1.00 0.00 H

ATOM 3596 HG SER A 228 41.809 -16.199 6.506 1.00 0.00 H

ATOM 3597 H SER A 228 42.787 -19.769 4.885 1.00 0.00 H

ATOM 3598 HA SER A 228 44.601 -17.697 5.524 1.00 0.00 H

ATOM 3599 N PRO A 229 45.674 -18.319 7.852 1.00 12.11 N

ATOM 3600 CA PRO A 229 46.395 -18.777 9.057 1.00 13.01 C

ATOM 3601 C PRO A 229 45.483 -19.416 10.118 1.00 13.52 C

ATOM 3602 O PRO A 229 44.423 -18.865 10.421 1.00 13.67 O

ATOM 3603 CB PRO A 229 47.052 -17.497 9.596 1.00 13.09 C

ATOM 3604 CG PRO A 229 47.187 -16.582 8.396 1.00 12.95 C

ATOM 3605 CD PRO A 229 45.941 -16.901 7.586 1.00 12.53 C

ATOM 3606 HA PRO A 229 47.165 -19.480 8.733 1.00 0.00 H

ATOM 3607 HB3 PRO A 229 48.008 -17.686 10.086 1.00 0.00 H

ATOM 3608 HB2 PRO A 229 46.400 -17.019 10.323 1.00 0.00 H

ATOM 3609 HG3 PRO A 229 48.073 -16.864 7.827 1.00 0.00 H

ATOM 3610 HG2 PRO A 229 47.276 -15.528 8.659 1.00 0.00 H

ATOM 3611 HD2 PRO A 229 45.091 -16.310 7.928 1.00 0.00 H

ATOM 3612 HD3 PRO A 229 46.129 -16.669 6.538 1.00 0.00 H

ATOM 3613 N ILE A 230 45.914 -20.576 10.638 1.00 14.32 N

ATOM 3614 CA ILE A 230 45.225 -21.323 11.688 1.00 15.62 C

ATOM 3615 C ILE A 230 45.437 -20.690 13.085 1.00 16.12 C

ATOM 3616 O ILE A 230 46.203 -21.199 13.903 1.00 17.33 O

ATOM 3617 CB ILE A 230 45.550 -22.847 11.626 1.00 0.00 C

ATOM 3618 CG1 ILE A 230 44.796 -23.681 12.692 1.00 0.00 C

ATOM 3619 CG2 ILE A 230 47.059 -23.174 11.599 1.00 0.00 C

ATOM 3620 CD1 ILE A 230 44.723 -25.184 12.385 1.00 0.00 C

ATOM 3621 HB ILE A 230 45.155 -23.178 10.663 1.00 0.00 H

ATOM 3622 HG12 ILE A 230 45.269 -23.560 13.662 1.00 0.00 H

ATOM 3623 HG13 ILE A 230 43.781 -23.302 12.807 1.00 0.00 H

ATOM 3624 HG21 ILE A 230 47.229 -24.247 11.526 1.00 0.00 H

ATOM 3625 HG22 ILE A 230 47.546 -22.720 10.735 1.00 0.00 H

ATOM 3626 HG23 ILE A 230 47.580 -22.818 12.484 1.00 0.00 H

ATOM 3627 HD11 ILE A 230 43.817 -25.620 12.807 1.00 0.00 H

ATOM 3628 HD12 ILE A 230 44.711 -25.381 11.313 1.00 0.00 H

ATOM 3629 HD13 ILE A 230 45.573 -25.714 12.814 1.00 0.00 H

ATOM 3630 H ILE A 230 46.788 -20.967 10.319 1.00 0.00 H

ATOM 3631 HA ILE A 230 44.164 -21.243 11.486 1.00 0.00 H

ATOM 3632 N PHE A 231 44.757 -19.552 13.309 1.00 16.35 N

ATOM 3633 CA PHE A 231 44.774 -18.793 14.562 1.00 17.04 C

ATOM 3634 C PHE A 231 43.840 -19.349 15.646 1.00 17.69 C

ATOM 3635 O PHE A 231 44.133 -19.156 16.825 1.00 18.40 O

ATOM 3636 CB PHE A 231 44.465 -17.302 14.292 1.00 0.00 C

ATOM 3637 CG PHE A 231 45.645 -16.478 13.808 1.00 0.00 C

ATOM 3638 CD1 PHE A 231 46.799 -16.356 14.613 1.00 0.00 C

ATOM 3639 CD2 PHE A 231 45.597 -15.800 12.572 1.00 0.00 C

ATOM 3640 CE1 PHE A 231 47.874 -15.594 14.177 1.00 0.00 C

ATOM 3641 CE2 PHE A 231 46.681 -15.035 12.159 1.00 0.00 C

ATOM 3642 CZ PHE A 231 47.816 -14.939 12.954 1.00 0.00 C

ATOM 3643 HB2 PHE A 231 43.647 -17.222 13.573 1.00 0.00 H

ATOM 3644 HB3 PHE A 231 44.103 -16.816 15.200 1.00 0.00 H

ATOM 3645 HD1 PHE A 231 46.851 -16.851 15.571 1.00 0.00 H

ATOM 3646 HD2 PHE A 231 44.721 -15.871 11.943 1.00 0.00 H

ATOM 3647 HE1 PHE A 231 48.757 -15.507 14.793 1.00 0.00 H

ATOM 3648 HE2 PHE A 231 46.644 -14.520 11.210 1.00 0.00 H

ATOM 3649 HZ PHE A 231 48.656 -14.347 12.621 1.00 0.00 H

ATOM 3650 H PHE A 231 44.175 -19.179 12.571 1.00 0.00 H

ATOM 3651 HA PHE A 231 45.777 -18.864 14.985 1.00 0.00 H

ATOM 3652 N ASP A 232 42.751 -20.019 15.237 1.00 17.85 N

ATOM 3653 CA ASP A 232 41.759 -20.607 16.131 1.00 18.39 C

ATOM 3654 C ASP A 232 41.951 -22.131 16.083 1.00 18.73 C

ATOM 3655 O ASP A 232 41.520 -22.780 15.129 1.00 18.68 O

ATOM 3656 CB ASP A 232 40.333 -20.147 15.736 1.00 18.23 C

ATOM 3657 CG ASP A 232 39.230 -20.485 16.748 1.00 19.28 C

ATOM 3658 OD1 ASP A 232 39.498 -21.260 17.693 1.00 18.76 O

ATOM 3659 OD2 ASP A 232 38.110 -19.972 16.531 1.00 19.91 O1-

ATOM 3660 H ASP A 232 42.577 -20.137 14.247 1.00 0.00 H

ATOM 3661 HA ASP A 232 41.934 -20.273 17.156 1.00 0.00 H

ATOM 3662 HB3 ASP A 232 40.061 -20.569 14.767 1.00 0.00 H

ATOM 3663 HB2 ASP A 232 40.350 -19.061 15.627 1.00 0.00 H

ATOM 3664 N LYS A 233 42.613 -22.664 17.123 1.00 19.37 N

ATOM 3665 CA LYS A 233 42.931 -24.087 17.279 1.00 19.96 C

ATOM 3666 C LYS A 233 41.721 -24.965 17.656 1.00 19.89 C

ATOM 3667 O LYS A 233 41.848 -26.188 17.603 1.00 20.51 O

ATOM 3668 CB LYS A 233 44.058 -24.247 18.325 1.00 20.37 C

ATOM 3669 CG LYS A 233 45.356 -23.471 18.024 1.00 22.45 C

ATOM 3670 CD LYS A 233 45.948 -23.751 16.635 1.00 25.72 C

ATOM 3671 CE LYS A 233 47.332 -23.118 16.449 1.00 27.42 C

ATOM 3672 NZ LYS A 233 47.862 -23.382 15.103 1.00 28.05 N1+

ATOM 3673 H LYS A 233 42.929 -22.056 17.865 1.00 0.00 H

ATOM 3674 HA LYS A 233 43.289 -24.463 16.319 1.00 0.00 H

ATOM 3675 HB3 LYS A 233 44.309 -25.304 18.422 1.00 0.00 H

ATOM 3676 HB2 LYS A 233 43.691 -23.937 19.305 1.00 0.00 H

ATOM 3677 HG3 LYS A 233 46.093 -23.724 18.787 1.00 0.00 H

ATOM 3678 HG2 LYS A 233 45.177 -22.400 18.131 1.00 0.00 H

ATOM 3679 HD3 LYS A 233 45.276 -23.341 15.882 1.00 0.00 H

ATOM 3680 HD2 LYS A 233 45.998 -24.827 16.461 1.00 0.00 H

ATOM 3681 HE3 LYS A 233 48.031 -23.515 17.185 1.00 0.00 H

ATOM 3682 HE2 LYS A 233 47.280 -22.039 16.599 1.00 0.00 H

ATOM 3683 HZ1 LYS A 233 47.251 -22.950 14.421 1.00 0.00 H

ATOM 3684 HZ2 LYS A 233 48.785 -22.981 15.020 1.00 0.00 H

ATOM 3685 HZ3 LYS A 233 47.909 -24.377 14.940 1.00 0.00 H

ATOM 3686 N THR A 234 40.585 -24.341 18.012 1.00 19.41 N

ATOM 3687 CA THR A 234 39.327 -25.005 18.348 1.00 19.40 C

ATOM 3688 C THR A 234 38.228 -24.630 17.326 1.00 18.82 C

ATOM 3689 O THR A 234 37.072 -24.428 17.692 1.00 18.61 O

ATOM 3690 CB THR A 234 38.915 -24.704 19.823 1.00 0.00 C

ATOM 3691 OG1 THR A 234 37.741 -25.404 20.196 1.00 0.00 O

ATOM 3692 CG2 THR A 234 38.686 -23.215 20.146 1.00 0.00 C

ATOM 3693 HB THR A 234 39.708 -25.071 20.475 1.00 0.00 H

ATOM 3694 HG1 THR A 234 37.013 -25.071 19.663 1.00 0.00 H

ATOM 3695 HG21 THR A 234 38.390 -23.085 21.187 1.00 0.00 H

ATOM 3696 HG22 THR A 234 39.600 -22.639 20.001 1.00 0.00 H

ATOM 3697 HG23 THR A 234 37.910 -22.764 19.529 1.00 0.00 H

ATOM 3698 H THR A 234 40.553 -23.330 18.009 1.00 0.00 H

ATOM 3699 HA THR A 234 39.442 -26.087 18.263 1.00 0.00 H

ATOM 3700 N SER A 235 38.604 -24.559 16.039 1.00 18.05 N

ATOM 3701 CA SER A 235 37.681 -24.306 14.938 1.00 18.08 C

ATOM 3702 C SER A 235 38.240 -24.919 13.651 1.00 17.29 C

ATOM 3703 O SER A 235 39.420 -24.740 13.351 1.00 17.18 O

ATOM 3704 CB SER A 235 37.408 -22.791 14.816 1.00 18.35 C

ATOM 3705 OG SER A 235 36.486 -22.498 13.787 1.00 19.96 O

ATOM 3706 H SER A 235 39.570 -24.716 15.784 1.00 0.00 H

ATOM 3707 HA SER A 235 36.735 -24.807 15.160 1.00 0.00 H

ATOM 3708 HB3 SER A 235 38.335 -22.257 14.606 1.00 0.00 H

ATOM 3709 HB2 SER A 235 37.010 -22.394 15.750 1.00 0.00 H

ATOM 3710 HG SER A 235 36.983 -22.391 12.968 1.00 0.00 H

ATOM 3711 N LYS A 236 37.355 -25.601 12.903 1.00 16.66 N

ATOM 3712 CA LYS A 236 37.604 -26.146 11.566 1.00 16.22 C

ATOM 3713 C LYS A 236 37.980 -25.042 10.562 1.00 15.15 C

ATOM 3714 O LYS A 236 38.969 -25.190 9.844 1.00 15.58 O

ATOM 3715 CB LYS A 236 36.350 -26.945 11.124 1.00 0.00 C

ATOM 3716 CG LYS A 236 36.323 -27.429 9.655 1.00 0.00 C

ATOM 3717 CD LYS A 236 34.935 -27.932 9.218 1.00 0.00 C

ATOM 3718 CE LYS A 236 34.771 -27.939 7.690 1.00 0.00 C

ATOM 3719 NZ LYS A 236 33.408 -28.329 7.294 1.00 0.00 N1+

ATOM 3720 HB2 LYS A 236 36.211 -27.800 11.787 1.00 0.00 H

ATOM 3721 HB3 LYS A 236 35.479 -26.309 11.286 1.00 0.00 H

ATOM 3722 HG2 LYS A 236 36.627 -26.635 8.974 1.00 0.00 H

ATOM 3723 HG3 LYS A 236 37.059 -28.222 9.521 1.00 0.00 H

ATOM 3724 HD2 LYS A 236 34.793 -28.945 9.599 1.00 0.00 H

ATOM 3725 HD3 LYS A 236 34.147 -27.331 9.673 1.00 0.00 H

ATOM 3726 HE2 LYS A 236 34.974 -26.949 7.280 1.00 0.00 H

ATOM 3727 HE3 LYS A 236 35.485 -28.628 7.235 1.00 0.00 H

ATOM 3728 HZ1 LYS A 236 33.335 -28.323 6.287 1.00 0.00 H

ATOM 3729 HZ2 LYS A 236 33.214 -29.258 7.638 1.00 0.00 H

ATOM 3730 HZ3 LYS A 236 32.742 -27.678 7.685 1.00 0.00 H

ATOM 3731 H LYS A 236 36.411 -25.708 13.247 1.00 0.00 H

ATOM 3732 HA LYS A 236 38.445 -26.837 11.640 1.00 0.00 H

ATOM 3733 N LEU A 237 37.173 -23.969 10.541 1.00 13.84 N

ATOM 3734 CA LEU A 237 37.357 -22.835 9.645 1.00 12.50 C

ATOM 3735 C LEU A 237 38.073 -21.677 10.356 1.00 11.53 C

ATOM 3736 O LEU A 237 38.032 -21.581 11.583 1.00 11.37 O

ATOM 3737 CB LEU A 237 35.996 -22.350 9.119 1.00 0.00 C

ATOM 3738 CG LEU A 237 35.160 -23.314 8.257 1.00 0.00 C

ATOM 3739 CD1 LEU A 237 35.922 -24.002 7.115 1.00 0.00 C

ATOM 3740 CD2 LEU A 237 34.318 -24.293 9.080 1.00 0.00 C

ATOM 3741 HB2 LEU A 237 35.391 -21.980 9.945 1.00 0.00 H

ATOM 3742 HB3 LEU A 237 36.205 -21.494 8.486 1.00 0.00 H

ATOM 3743 HG LEU A 237 34.444 -22.657 7.777 1.00 0.00 H

ATOM 3744 HD11 LEU A 237 35.263 -24.659 6.549 1.00 0.00 H

ATOM 3745 HD12 LEU A 237 36.312 -23.269 6.412 1.00 0.00 H

ATOM 3746 HD13 LEU A 237 36.755 -24.604 7.479 1.00 0.00 H

ATOM 3747 HD21 LEU A 237 33.978 -23.851 10.017 1.00 0.00 H

ATOM 3748 HD22 LEU A 237 33.437 -24.618 8.527 1.00 0.00 H

ATOM 3749 HD23 LEU A 237 34.894 -25.176 9.325 1.00 0.00 H

ATOM 3750 H LEU A 237 36.387 -23.921 11.172 1.00 0.00 H

ATOM 3751 HA LEU A 237 37.977 -23.145 8.807 1.00 0.00 H

ATOM 3752 N GLN A 238 38.704 -20.810 9.551 1.00 10.26 N

ATOM 3753 CA GLN A 238 39.529 -19.688 9.995 1.00 9.76 C

ATOM 3754 C GLN A 238 39.055 -18.382 9.355 1.00 8.98 C

ATOM 3755 O GLN A 238 38.584 -18.400 8.220 1.00 8.38 O

ATOM 3756 CB GLN A 238 40.991 -19.968 9.579 1.00 10.33 C

ATOM 3757 CG GLN A 238 41.590 -21.259 10.171 1.00 10.80 C

ATOM 3758 CD GLN A 238 41.504 -21.323 11.700 1.00 11.14 C

ATOM 3759 OE1 GLN A 238 41.747 -20.332 12.386 1.00 11.33 O

ATOM 3760 NE2 GLN A 238 41.190 -22.500 12.238 1.00 14.09 N

ATOM 3761 H GLN A 238 38.671 -20.959 8.549 1.00 0.00 H

ATOM 3762 HA GLN A 238 39.459 -19.562 11.077 1.00 0.00 H

ATOM 3763 HB3 GLN A 238 41.619 -19.124 9.871 1.00 0.00 H

ATOM 3764 HB2 GLN A 238 41.062 -20.024 8.491 1.00 0.00 H

ATOM 3765 HG3 GLN A 238 42.633 -21.341 9.866 1.00 0.00 H

ATOM 3766 HG2 GLN A 238 41.088 -22.127 9.742 1.00 0.00 H

ATOM 3767 HE22 GLN A 238 41.143 -22.605 13.245 1.00 0.00 H

ATOM 3768 HE21 GLN A 238 40.983 -23.299 11.655 1.00 0.00 H

ATOM 3769 N HIS A 239 39.235 -17.264 10.079 1.00 8.67 N

ATOM 3770 CA HIS A 239 38.941 -15.900 9.623 1.00 9.07 C

ATOM 3771 C HIS A 239 39.642 -15.571 8.283 1.00 8.88 C

ATOM 3772 O HIS A 239 40.871 -15.635 8.238 1.00 9.60 O

ATOM 3773 CB HIS A 239 39.420 -14.901 10.700 1.00 0.00 C

ATOM 3774 CG HIS A 239 38.410 -14.605 11.778 1.00 0.00 C

ATOM 3775 ND1 HIS A 239 37.438 -13.609 11.631 1.00 0.00 N

ATOM 3776 CD2 HIS A 239 38.267 -15.189 13.019 1.00 0.00 C

ATOM 3777 CE1 HIS A 239 36.767 -13.617 12.774 1.00 0.00 C

ATOM 3778 NE2 HIS A 239 37.214 -14.534 13.633 1.00 0.00 N

ATOM 3779 HB2 HIS A 239 40.344 -15.248 11.167 1.00 0.00 H

ATOM 3780 HB3 HIS A 239 39.668 -13.939 10.247 1.00 0.00 H

ATOM 3781 HD2 HIS A 239 38.820 -15.978 13.507 1.00 0.00 H

ATOM 3782 HE1 HIS A 239 35.944 -12.949 12.988 1.00 0.00 H

ATOM 3783 HE2 HIS A 239 36.864 -14.709 14.566 1.00 0.00 H

ATOM 3784 H HIS A 239 39.653 -17.337 10.995 1.00 0.00 H

ATOM 3785 HA HIS A 239 37.859 -15.823 9.545 1.00 0.00 H

ATOM 3786 N PRO A 240 38.872 -15.227 7.221 1.00 8.55 N

ATOM 3787 CA PRO A 240 39.443 -14.859 5.912 1.00 8.58 C

ATOM 3788 C PRO A 240 40.082 -13.455 5.844 1.00 8.20 C

ATOM 3789 O PRO A 240 40.491 -13.068 4.752 1.00 7.87 O

ATOM 3790 CD PRO A 240 37.408 -15.182 7.185 1.00 0.00 C

ATOM 3791 CB PRO A 240 38.238 -14.978 4.966 1.00 0.00 C

ATOM 3792 CG PRO A 240 37.048 -14.600 5.824 1.00 0.00 C

ATOM 3793 HD3 PRO A 240 37.027 -16.200 7.276 1.00 0.00 H

ATOM 3794 HD2 PRO A 240 36.978 -14.573 7.981 1.00 0.00 H

ATOM 3795 HB2 PRO A 240 38.310 -14.378 4.058 1.00 0.00 H

ATOM 3796 HB3 PRO A 240 38.131 -16.014 4.659 1.00 0.00 H

ATOM 3797 HG3 PRO A 240 36.098 -14.973 5.443 1.00 0.00 H

ATOM 3798 HG2 PRO A 240 36.981 -13.513 5.893 1.00 0.00 H

ATOM 3799 HA PRO A 240 40.193 -15.584 5.599 1.00 0.00 H

ATOM 3800 N ASN A 241 40.143 -12.727 6.978 1.00 8.38 N

ATOM 3801 CA ASN A 241 40.531 -11.316 7.119 1.00 8.95 C

ATOM 3802 C ASN A 241 41.824 -10.915 6.389 1.00 8.71 C

ATOM 3803 O ASN A 241 41.825 -9.882 5.724 1.00 8.14 O

ATOM 3804 CB ASN A 241 40.535 -10.959 8.632 1.00 0.00 C

ATOM 3805 CG ASN A 241 40.949 -9.519 8.987 1.00 0.00 C

ATOM 3806 OD1 ASN A 241 42.126 -9.169 8.930 1.00 0.00 O

ATOM 3807 ND2 ASN A 241 39.991 -8.684 9.392 1.00 0.00 N

ATOM 3808 HB2 ASN A 241 39.547 -11.156 9.052 1.00 0.00 H

ATOM 3809 HB3 ASN A 241 41.217 -11.629 9.158 1.00 0.00 H

ATOM 3810 HD22 ASN A 241 40.226 -7.738 9.653 1.00 0.00 H

ATOM 3811 HD21 ASN A 241 39.029 -8.988 9.436 1.00 0.00 H

ATOM 3812 H ASN A 241 39.803 -13.153 7.827 1.00 0.00 H

ATOM 3813 HA ASN A 241 39.716 -10.782 6.638 1.00 0.00 H

ATOM 3814 N TRP A 242 42.877 -11.743 6.492 1.00 8.77 N

ATOM 3815 CA TRP A 242 44.179 -11.492 5.869 1.00 8.97 C

ATOM 3816 C TRP A 242 44.121 -11.402 4.327 1.00 8.73 C

ATOM 3817 O TRP A 242 44.932 -10.685 3.740 1.00 8.60 O

ATOM 3818 CB TRP A 242 45.162 -12.582 6.347 1.00 0.00 C

ATOM 3819 CG TRP A 242 46.618 -12.312 6.101 1.00 0.00 C

ATOM 3820 CD1 TRP A 242 47.438 -11.673 6.966 1.00 0.00 C

ATOM 3821 CD2 TRP A 242 47.427 -12.581 4.912 1.00 0.00 C

ATOM 3822 NE1 TRP A 242 48.693 -11.546 6.412 1.00 0.00 N

ATOM 3823 CE2 TRP A 242 48.745 -12.078 5.141 1.00 0.00 C

ATOM 3824 CE3 TRP A 242 47.186 -13.197 3.661 1.00 0.00 C

ATOM 3825 CZ2 TRP A 242 49.766 -12.174 4.179 1.00 0.00 C

ATOM 3826 CZ3 TRP A 242 48.206 -13.309 2.693 1.00 0.00 C

ATOM 3827 CH2 TRP A 242 49.491 -12.792 2.946 1.00 0.00 C

ATOM 3828 HB2 TRP A 242 45.050 -12.719 7.424 1.00 0.00 H

ATOM 3829 HB3 TRP A 242 44.905 -13.544 5.900 1.00 0.00 H

ATOM 3830 HD1 TRP A 242 47.138 -11.319 7.942 1.00 0.00 H

ATOM 3831 HE1 TRP A 242 49.459 -11.099 6.895 1.00 0.00 H

ATOM 3832 HE3 TRP A 242 46.205 -13.594 3.446 1.00 0.00 H

ATOM 3833 HZ2 TRP A 242 50.750 -11.779 4.385 1.00 0.00 H

ATOM 3834 HZ3 TRP A 242 48.000 -13.796 1.752 1.00 0.00 H

ATOM 3835 HH2 TRP A 242 50.266 -12.877 2.199 1.00 0.00 H

ATOM 3836 H TRP A 242 42.795 -12.587 7.039 1.00 0.00 H

ATOM 3837 HA TRP A 242 44.526 -10.529 6.251 1.00 0.00 H

ATOM 3838 N GLY A 243 43.157 -12.112 3.715 1.00 8.21 N

ATOM 3839 CA GLY A 243 42.971 -12.204 2.272 1.00 7.72 C

ATOM 3840 C GLY A 243 41.635 -11.618 1.792 1.00 7.17 C

ATOM 3841 O GLY A 243 41.317 -11.828 0.624 1.00 7.72 O

ATOM 3842 HA3 GLY A 243 43.016 -13.256 1.988 1.00 0.00 H

ATOM 3843 H GLY A 243 42.510 -12.643 4.284 1.00 0.00 H

ATOM 3844 HA2 GLY A 243 43.767 -11.698 1.730 1.00 0.00 H

ATOM 3845 N SER A 244 40.855 -10.919 2.641 1.00 6.69 N

ATOM 3846 CA SER A 244 39.536 -10.386 2.276 1.00 6.77 C

ATOM 3847 C SER A 244 39.301 -8.960 2.798 1.00 6.40 C

ATOM 3848 O SER A 244 38.807 -8.138 2.028 1.00 6.90 O

ATOM 3849 CB SER A 244 38.425 -11.366 2.709 1.00 0.00 C

ATOM 3850 OG SER A 244 37.156 -10.941 2.253 1.00 0.00 O

ATOM 3851 HB2 SER A 244 38.616 -12.361 2.303 1.00 0.00 H

ATOM 3852 HB3 SER A 244 38.395 -11.467 3.795 1.00 0.00 H

ATOM 3853 HG SER A 244 36.930 -10.117 2.691 1.00 0.00 H

ATOM 3854 H SER A 244 41.162 -10.766 3.592 1.00 0.00 H

ATOM 3855 HA SER A 244 39.478 -10.302 1.188 1.00 0.00 H

ATOM 3856 N HIS A 245 39.644 -8.671 4.068 1.00 6.14 N

ATOM 3857 CA HIS A 245 39.527 -7.324 4.637 1.00 6.75 C

ATOM 3858 C HIS A 245 40.671 -6.428 4.161 1.00 6.46 C

ATOM 3859 O HIS A 245 41.838 -6.782 4.336 1.00 6.99 O

ATOM 3860 CB HIS A 245 39.531 -7.347 6.174 1.00 0.00 C

ATOM 3861 CG HIS A 245 38.246 -7.767 6.829 1.00 0.00 C

ATOM 3862 ND1 HIS A 245 37.884 -9.106 6.993 1.00 0.00 N

ATOM 3863 CD2 HIS A 245 37.284 -6.971 7.416 1.00 0.00 C

ATOM 3864 CE1 HIS A 245 36.770 -9.073 7.705 1.00 0.00 C

ATOM 3865 NE2 HIS A 245 36.356 -7.834 7.970 1.00 0.00 N

ATOM 3866 HB2 HIS A 245 40.335 -7.980 6.544 1.00 0.00 H

ATOM 3867 HB3 HIS A 245 39.753 -6.349 6.559 1.00 0.00 H

ATOM 3868 HD2 HIS A 245 37.203 -5.897 7.507 1.00 0.00 H

ATOM 3869 HE1 HIS A 245 36.260 -9.965 8.037 1.00 0.00 H

ATOM 3870 HE2 HIS A 245 35.531 -7.574 8.492 1.00 0.00 H

ATOM 3871 H HIS A 245 40.074 -9.370 4.658 1.00 0.00 H

ATOM 3872 HA HIS A 245 38.583 -6.879 4.314 1.00 0.00 H

ATOM 3873 N ASN A 246 40.288 -5.264 3.611 1.00 6.60 N

ATOM 3874 CA ASN A 246 41.151 -4.176 3.147 1.00 6.65 C

ATOM 3875 C ASN A 246 42.204 -4.678 2.139 1.00 6.89 C

ATOM 3876 O ASN A 246 43.396 -4.422 2.313 1.00 7.56 O

ATOM 3877 CB ASN A 246 41.769 -3.415 4.353 1.00 7.17 C

ATOM 3878 CG ASN A 246 40.735 -2.784 5.297 1.00 7.47 C

ATOM 3879 OD1 ASN A 246 39.655 -2.378 4.873 1.00 7.47 O

ATOM 3880 ND2 ASN A 246 41.070 -2.676 6.583 1.00 9.04 N

ATOM 3881 H ASN A 246 39.297 -5.098 3.492 1.00 0.00 H

ATOM 3882 HA ASN A 246 40.488 -3.499 2.610 1.00 0.00 H

ATOM 3883 HB3 ASN A 246 42.404 -2.605 3.992 1.00 0.00 H

ATOM 3884 HB2 ASN A 246 42.417 -4.081 4.927 1.00 0.00 H

ATOM 3885 HD22 ASN A 246 40.447 -2.211 7.229 1.00 0.00 H

ATOM 3886 HD21 ASN A 246 41.972 -2.990 6.910 1.00 0.00 H

ATOM 3887 N GLY A 247 41.731 -5.428 1.126 1.00 6.38 N

ATOM 3888 CA GLY A 247 42.555 -6.113 0.129 1.00 6.56 C

ATOM 3889 C GLY A 247 43.334 -5.140 -0.779 1.00 6.72 C

ATOM 3890 O GLY A 247 43.120 -3.928 -0.720 1.00 7.31 O

ATOM 3891 H GLY A 247 40.736 -5.599 1.078 1.00 0.00 H

ATOM 3892 HA3 GLY A 247 41.912 -6.739 -0.484 1.00 0.00 H

ATOM 3893 HA2 GLY A 247 43.250 -6.776 0.645 1.00 0.00 H

ATOM 3894 N PRO A 248 44.247 -5.662 -1.627 1.00 6.96 N

ATOM 3895 CA PRO A 248 45.200 -4.842 -2.404 1.00 7.53 C

ATOM 3896 C PRO A 248 44.597 -3.866 -3.438 1.00 7.76 C

ATOM 3897 O PRO A 248 45.274 -2.899 -3.787 1.00 9.56 O

ATOM 3898 CB PRO A 248 46.128 -5.886 -3.053 1.00 7.69 C

ATOM 3899 CG PRO A 248 45.302 -7.162 -3.114 1.00 7.79 C

ATOM 3900 CD PRO A 248 44.473 -7.093 -1.839 1.00 6.62 C

ATOM 3901 HA PRO A 248 45.786 -4.239 -1.707 1.00 0.00 H

ATOM 3902 HB3 PRO A 248 46.995 -6.044 -2.410 1.00 0.00 H

ATOM 3903 HB2 PRO A 248 46.505 -5.592 -4.034 1.00 0.00 H

ATOM 3904 HG3 PRO A 248 45.902 -8.069 -3.192 1.00 0.00 H

ATOM 3905 HG2 PRO A 248 44.638 -7.123 -3.979 1.00 0.00 H

ATOM 3906 HD2 PRO A 248 43.550 -7.667 -1.941 1.00 0.00 H

ATOM 3907 HD3 PRO A 248 45.035 -7.492 -0.993 1.00 0.00 H

ATOM 3908 N ARG A 249 43.352 -4.108 -3.881 1.00 6.68 N

ATOM 3909 CA ARG A 249 42.619 -3.272 -4.837 1.00 6.53 C

ATOM 3910 C ARG A 249 41.437 -2.526 -4.193 1.00 6.67 C

ATOM 3911 O ARG A 249 40.627 -1.968 -4.932 1.00 6.72 O

ATOM 3912 CB ARG A 249 42.117 -4.144 -6.008 1.00 6.70 C

ATOM 3913 CG ARG A 249 43.228 -4.891 -6.764 1.00 7.33 C

ATOM 3914 CD ARG A 249 42.714 -5.580 -8.036 1.00 8.60 C

ATOM 3915 NE ARG A 249 42.337 -4.606 -9.072 1.00 7.54 N

ATOM 3916 CZ ARG A 249 41.633 -4.871 -10.185 1.00 9.45 C

ATOM 3917 NH1 ARG A 249 41.179 -6.104 -10.453 1.00 8.23 N

ATOM 3918 NH2 ARG A 249 41.387 -3.878 -11.048 1.00 9.85 N1+

ATOM 3919 H ARG A 249 42.855 -4.922 -3.543 1.00 0.00 H

ATOM 3920 HA ARG A 249 43.274 -2.503 -5.252 1.00 0.00 H

ATOM 3921 HB3 ARG A 249 41.589 -3.509 -6.721 1.00 0.00 H

ATOM 3922 HB2 ARG A 249 41.380 -4.864 -5.650 1.00 0.00 H

ATOM 3923 HG3 ARG A 249 43.777 -5.595 -6.138 1.00 0.00 H

ATOM 3924 HG2 ARG A 249 43.950 -4.133 -7.069 1.00 0.00 H

ATOM 3925 HD3 ARG A 249 41.780 -6.072 -7.771 1.00 0.00 H

ATOM 3926 HD2 ARG A 249 43.380 -6.365 -8.394 1.00 0.00 H

ATOM 3927 HE ARG A 249 42.670 -3.655 -8.931 1.00 0.00 H

ATOM 3928 HH12 ARG A 249 40.661 -6.294 -11.297 1.00 0.00 H

ATOM 3929 HH11 ARG A 249 41.315 -6.856 -9.782 1.00 0.00 H

ATOM 3930 HH22 ARG A 249 40.879 -4.048 -11.902 1.00 0.00 H

ATOM 3931 HH21 ARG A 249 41.723 -2.935 -10.854 1.00 0.00 H

ATOM 3932 N ILE A 250 41.314 -2.525 -2.853 1.00 6.57 N

ATOM 3933 CA ILE A 250 40.144 -1.968 -2.167 1.00 6.30 C

ATOM 3934 C ILE A 250 39.997 -0.440 -2.340 1.00 6.62 C

ATOM 3935 O ILE A 250 38.882 0.035 -2.543 1.00 7.25 O

ATOM 3936 CB ILE A 250 40.134 -2.340 -0.653 1.00 6.39 C

ATOM 3937 CG1 ILE A 250 38.729 -2.278 -0.018 1.00 6.36 C

ATOM 3938 CG2 ILE A 250 41.132 -1.531 0.209 1.00 6.70 C

ATOM 3939 CD1 ILE A 250 37.651 -3.061 -0.779 1.00 5.63 C

ATOM 3940 H ILE A 250 42.006 -2.987 -2.277 1.00 0.00 H

ATOM 3941 HA ILE A 250 39.281 -2.420 -2.657 1.00 0.00 H

ATOM 3942 HB ILE A 250 40.448 -3.382 -0.589 1.00 0.00 H

ATOM 3943 HG13 ILE A 250 38.414 -1.241 0.086 1.00 0.00 H

ATOM 3944 HG12 ILE A 250 38.786 -2.676 0.995 1.00 0.00 H

ATOM 3945 HG21 ILE A 250 41.231 -1.953 1.206 1.00 0.00 H

ATOM 3946 HG22 ILE A 250 42.128 -1.522 -0.233 1.00 0.00 H

ATOM 3947 HG23 ILE A 250 40.817 -0.496 0.340 1.00 0.00 H

ATOM 3948 HD11 ILE A 250 36.895 -3.451 -0.102 1.00 0.00 H

ATOM 3949 HD12 ILE A 250 37.143 -2.423 -1.502 1.00 0.00 H

ATOM 3950 HD13 ILE A 250 38.074 -3.912 -1.310 1.00 0.00 H

ATOM 3951 N HIS A 251 41.129 0.284 -2.327 1.00 6.55 N

ATOM 3952 CA HIS A 251 41.180 1.730 -2.538 1.00 6.40 C

ATOM 3953 C HIS A 251 40.985 2.112 -4.012 1.00 7.31 C

ATOM 3954 O HIS A 251 40.346 3.129 -4.266 1.00 6.79 O

ATOM 3955 CB HIS A 251 42.505 2.284 -1.987 1.00 6.48 C

ATOM 3956 CG HIS A 251 42.637 2.186 -0.486 1.00 7.10 C

ATOM 3957 ND1 HIS A 251 41.732 2.793 0.371 1.00 8.81 N1+

ATOM 3958 CD2 HIS A 251 43.458 1.396 0.298 1.00 4.66 C

ATOM 3959 CE1 HIS A 251 42.043 2.414 1.607 1.00 6.15 C

ATOM 3960 NE2 HIS A 251 43.079 1.579 1.617 1.00 9.51 N

ATOM 3961 H HIS A 251 42.012 -0.184 -2.184 1.00 0.00 H

ATOM 3962 HA HIS A 251 40.359 2.188 -1.985 1.00 0.00 H

ATOM 3963 HB3 HIS A 251 42.598 3.334 -2.259 1.00 0.00 H

ATOM 3964 HB2 HIS A 251 43.351 1.775 -2.450 1.00 0.00 H

ATOM 3965 HD1 HIS A 251 40.963 3.409 0.098 1.00 0.00 H

ATOM 3966 HD2 HIS A 251 44.260 0.719 0.040 1.00 0.00 H

ATOM 3967 HE1 HIS A 251 41.516 2.738 2.492 1.00 0.00 H

ATOM 3968 HE2 HIS A 251 43.487 1.139 2.431 1.00 0.00 H

ATOM 3969 N GLU A 252 41.467 1.272 -4.947 1.00 7.51 N

ATOM 3970 CA GLU A 252 41.205 1.380 -6.386 1.00 7.37 C

ATOM 3971 C GLU A 252 39.699 1.314 -6.703 1.00 7.43 C

ATOM 3972 O GLU A 252 39.208 2.154 -7.455 1.00 8.05 O

ATOM 3973 CB GLU A 252 42.010 0.291 -7.136 1.00 7.78 C

ATOM 3974 CG GLU A 252 41.730 0.166 -8.653 1.00 9.65 C

ATOM 3975 CD GLU A 252 42.412 -1.033 -9.320 1.00 11.25 C

ATOM 3976 OE1 GLU A 252 43.095 -1.811 -8.617 1.00 12.72 O

ATOM 3977 OE2 GLU A 252 42.198 -1.186 -10.542 1.00 14.14 O1-

ATOM 3978 H GLU A 252 41.966 0.444 -4.656 1.00 0.00 H

ATOM 3979 HA GLU A 252 41.566 2.356 -6.717 1.00 0.00 H

ATOM 3980 HB3 GLU A 252 41.832 -0.674 -6.666 1.00 0.00 H

ATOM 3981 HB2 GLU A 252 43.074 0.488 -6.996 1.00 0.00 H

ATOM 3982 HG3 GLU A 252 42.041 1.078 -9.163 1.00 0.00 H

ATOM 3983 HG2 GLU A 252 40.664 0.048 -8.846 1.00 0.00 H

ATOM 3984 N TYR A 253 39.005 0.337 -6.095 1.00 6.60 N

ATOM 3985 CA TYR A 253 37.567 0.117 -6.242 1.00 6.29 C

ATOM 3986 C TYR A 253 36.710 1.248 -5.662 1.00 5.83 C

ATOM 3987 O TYR A 253 35.739 1.626 -6.313 1.00 6.75 O

ATOM 3988 CB TYR A 253 37.177 -1.238 -5.618 1.00 0.00 C

ATOM 3989 CG TYR A 253 37.748 -2.491 -6.267 1.00 0.00 C

ATOM 3990 CD1 TYR A 253 38.188 -2.500 -7.612 1.00 0.00 C

ATOM 3991 CD2 TYR A 253 37.810 -3.683 -5.513 1.00 0.00 C

ATOM 3992 CE1 TYR A 253 38.677 -3.682 -8.191 1.00 0.00 C

ATOM 3993 CE2 TYR A 253 38.295 -4.868 -6.096 1.00 0.00 C

ATOM 3994 CZ TYR A 253 38.722 -4.864 -7.436 1.00 0.00 C

ATOM 3995 OH TYR A 253 39.173 -6.003 -8.021 1.00 0.00 O

ATOM 3996 HB2 TYR A 253 37.455 -1.243 -4.562 1.00 0.00 H

ATOM 3997 HB3 TYR A 253 36.093 -1.346 -5.636 1.00 0.00 H

ATOM 3998 HD1 TYR A 253 38.154 -1.616 -8.229 1.00 0.00 H

ATOM 3999 HD2 TYR A 253 37.478 -3.691 -4.487 1.00 0.00 H

ATOM 4000 HE1 TYR A 253 39.005 -3.682 -9.221 1.00 0.00 H

ATOM 4001 HE2 TYR A 253 38.323 -5.780 -5.520 1.00 0.00 H

ATOM 4002 HH TYR A 253 39.271 -6.756 -7.425 1.00 0.00 H

ATOM 4003 H TYR A 253 39.497 -0.325 -5.509 1.00 0.00 H

ATOM 4004 HA TYR A 253 37.338 0.101 -7.308 1.00 0.00 H

ATOM 4005 N HIS A 254 37.083 1.786 -4.488 1.00 6.39 N

ATOM 4006 CA HIS A 254 36.383 2.913 -3.865 1.00 6.23 C

ATOM 4007 C HIS A 254 36.630 4.259 -4.563 1.00 7.08 C

ATOM 4008 O HIS A 254 35.712 5.078 -4.583 1.00 7.17 O

ATOM 4009 CB HIS A 254 36.679 2.964 -2.355 1.00 6.40 C

ATOM 4010 CG HIS A 254 35.797 2.018 -1.585 1.00 6.05 C

ATOM 4011 ND1 HIS A 254 34.438 2.281 -1.384 1.00 8.07 N

ATOM 4012 CD2 HIS A 254 36.097 0.795 -1.024 1.00 7.10 C

ATOM 4013 CE1 HIS A 254 33.972 1.214 -0.754 1.00 6.93 C

ATOM 4014 NE2 HIS A 254 34.911 0.301 -0.517 1.00 6.42 N

ATOM 4015 H HIS A 254 37.890 1.424 -3.998 1.00 0.00 H

ATOM 4016 HA HIS A 254 35.310 2.742 -3.981 1.00 0.00 H

ATOM 4017 HB3 HIS A 254 36.500 3.966 -1.960 1.00 0.00 H

ATOM 4018 HB2 HIS A 254 37.727 2.740 -2.152 1.00 0.00 H

ATOM 4019 HD2 HIS A 254 37.017 0.240 -0.962 1.00 0.00 H

ATOM 4020 HE1 HIS A 254 32.938 1.097 -0.469 1.00 0.00 H

ATOM 4021 HE2 HIS A 254 34.782 -0.594 -0.059 1.00 0.00 H

ATOM 4022 N GLN A 255 37.813 4.443 -5.176 1.00 7.34 N

ATOM 4023 CA GLN A 255 38.103 5.575 -6.061 1.00 8.32 C

ATOM 4024 C GLN A 255 37.293 5.530 -7.364 1.00 7.85 C

ATOM 4025 O GLN A 255 36.821 6.575 -7.809 1.00 8.44 O

ATOM 4026 CB GLN A 255 39.616 5.683 -6.346 1.00 8.34 C

ATOM 4027 CG GLN A 255 40.397 6.248 -5.148 1.00 12.17 C

ATOM 4028 CD GLN A 255 41.911 6.309 -5.337 1.00 16.35 C

ATOM 4029 OE1 GLN A 255 42.433 6.105 -6.431 1.00 18.35 O

ATOM 4030 NE2 GLN A 255 42.626 6.600 -4.249 1.00 19.43 N

ATOM 4031 H GLN A 255 38.537 3.742 -5.093 1.00 0.00 H

ATOM 4032 HA GLN A 255 37.796 6.481 -5.539 1.00 0.00 H

ATOM 4033 HB3 GLN A 255 39.785 6.335 -7.205 1.00 0.00 H

ATOM 4034 HB2 GLN A 255 40.011 4.705 -6.626 1.00 0.00 H

ATOM 4035 HG3 GLN A 255 40.216 5.624 -4.282 1.00 0.00 H

ATOM 4036 HG2 GLN A 255 40.033 7.246 -4.906 1.00 0.00 H

ATOM 4037 HE22 GLN A 255 43.630 6.673 -4.306 1.00 0.00 H

ATOM 4038 HE21 GLN A 255 42.162 6.732 -3.360 1.00 0.00 H

ATOM 4039 N GLU A 256 37.124 4.320 -7.925 1.00 7.80 N

ATOM 4040 CA GLU A 256 36.352 4.065 -9.139 1.00 7.71 C

ATOM 4041 C GLU A 256 34.838 4.275 -8.926 1.00 7.74 C

ATOM 4042 O GLU A 256 34.198 4.864 -9.798 1.00 7.91 O

ATOM 4043 CB GLU A 256 36.669 2.647 -9.654 1.00 8.19 C

ATOM 4044 CG GLU A 256 36.200 2.401 -11.098 1.00 8.17 C

ATOM 4045 CD GLU A 256 36.354 0.943 -11.511 1.00 11.43 C

ATOM 4046 OE1 GLU A 256 37.272 0.650 -12.307 1.00 13.18 O

ATOM 4047 OE2 GLU A 256 35.534 0.134 -11.030 1.00 11.98 O1-

ATOM 4048 H GLU A 256 37.562 3.512 -7.501 1.00 0.00 H

ATOM 4049 HA GLU A 256 36.692 4.780 -9.890 1.00 0.00 H

ATOM 4050 HB3 GLU A 256 36.225 1.906 -8.987 1.00 0.00 H

ATOM 4051 HB2 GLU A 256 37.746 2.476 -9.613 1.00 0.00 H

ATOM 4052 HG3 GLU A 256 36.787 3.020 -11.774 1.00 0.00 H

ATOM 4053 HG2 GLU A 256 35.157 2.683 -11.230 1.00 0.00 H

ATOM 4054 N LEU A 257 34.308 3.828 -7.770 1.00 6.99 N

ATOM 4055 CA LEU A 257 32.915 4.034 -7.349 1.00 7.25 C

ATOM 4056 C LEU A 257 32.609 5.515 -7.070 1.00 7.24 C

ATOM 4057 O LEU A 257 31.545 5.977 -7.477 1.00 6.91 O

ATOM 4058 CB LEU A 257 32.530 3.045 -6.204 1.00 0.00 C

ATOM 4059 CG LEU A 257 32.007 3.586 -4.844 1.00 0.00 C

ATOM 4060 CD1 LEU A 257 30.630 4.282 -4.933 1.00 0.00 C

ATOM 4061 CD2 LEU A 257 31.967 2.457 -3.795 1.00 0.00 C

ATOM 4062 HB2 LEU A 257 31.771 2.365 -6.587 1.00 0.00 H

ATOM 4063 HB3 LEU A 257 33.385 2.404 -6.001 1.00 0.00 H

ATOM 4064 HG LEU A 257 32.735 4.310 -4.477 1.00 0.00 H

ATOM 4065 HD11 LEU A 257 29.919 3.899 -4.200 1.00 0.00 H

ATOM 4066 HD12 LEU A 257 30.723 5.353 -4.749 1.00 0.00 H

ATOM 4067 HD13 LEU A 257 30.172 4.156 -5.915 1.00 0.00 H

ATOM 4068 HD21 LEU A 257 32.137 2.859 -2.797 1.00 0.00 H

ATOM 4069 HD22 LEU A 257 31.004 1.943 -3.781 1.00 0.00 H

ATOM 4070 HD23 LEU A 257 32.730 1.698 -3.971 1.00 0.00 H

ATOM 4071 H LEU A 257 34.899 3.331 -7.116 1.00 0.00 H

ATOM 4072 HA LEU A 257 32.301 3.756 -8.208 1.00 0.00 H

ATOM 4073 N HIS A 258 33.542 6.236 -6.422 1.00 7.18 N

ATOM 4074 CA HIS A 258 33.429 7.675 -6.166 1.00 7.01 C

ATOM 4075 C HIS A 258 33.388 8.492 -7.469 1.00 7.16 C

ATOM 4076 O HIS A 258 32.538 9.371 -7.593 1.00 6.40 O

ATOM 4077 CB HIS A 258 34.574 8.136 -5.247 1.00 0.00 C

ATOM 4078 CG HIS A 258 34.542 9.601 -4.873 1.00 0.00 C

ATOM 4079 ND1 HIS A 258 34.315 10.040 -3.581 1.00 0.00 N

ATOM 4080 CD2 HIS A 258 34.741 10.744 -5.617 1.00 0.00 C

ATOM 4081 CE1 HIS A 258 34.372 11.374 -3.599 1.00 0.00 C

ATOM 4082 NE2 HIS A 258 34.616 11.871 -4.805 1.00 0.00 N

ATOM 4083 HB2 HIS A 258 34.562 7.549 -4.328 1.00 0.00 H

ATOM 4084 HB3 HIS A 258 35.532 7.928 -5.722 1.00 0.00 H

ATOM 4085 HD2 HIS A 258 34.961 10.846 -6.670 1.00 0.00 H

ATOM 4086 HE1 HIS A 258 34.231 11.986 -2.721 1.00 0.00 H

ATOM 4087 H HIS A 258 34.388 5.787 -6.096 1.00 0.00 H

ATOM 4088 HA HIS A 258 32.488 7.842 -5.637 1.00 0.00 H

ATOM 4089 HD1 HIS A 258 34.158 9.451 -2.772 1.00 0.00 H

ATOM 4090 N ARG A 259 34.278 8.154 -8.419 1.00 7.33 N

ATOM 4091 CA ARG A 259 34.308 8.679 -9.786 1.00 8.10 C

ATOM 4092 C ARG A 259 32.977 8.467 -10.528 1.00 7.42 C

ATOM 4093 O ARG A 259 32.503 9.403 -11.171 1.00 7.56 O

ATOM 4094 CB ARG A 259 35.525 8.067 -10.521 1.00 0.00 C

ATOM 4095 CG ARG A 259 35.441 7.998 -12.058 1.00 0.00 C

ATOM 4096 CD ARG A 259 36.728 7.453 -12.686 1.00 0.00 C

ATOM 4097 NE ARG A 259 36.497 6.986 -14.059 1.00 0.00 N

ATOM 4098 CZ ARG A 259 36.065 5.754 -14.379 1.00 0.00 C

ATOM 4099 NH1 ARG A 259 35.818 4.834 -13.436 1.00 0.00 N

ATOM 4100 NH2 ARG A 259 35.884 5.430 -15.664 1.00 0.00 N1+

ATOM 4101 HB2 ARG A 259 36.429 8.597 -10.216 1.00 0.00 H

ATOM 4102 HB3 ARG A 259 35.659 7.042 -10.180 1.00 0.00 H

ATOM 4103 HG2 ARG A 259 34.584 7.424 -12.413 1.00 0.00 H

ATOM 4104 HG3 ARG A 259 35.301 9.021 -12.409 1.00 0.00 H

ATOM 4105 HD2 ARG A 259 37.392 8.306 -12.825 1.00 0.00 H

ATOM 4106 HD3 ARG A 259 37.275 6.750 -12.057 1.00 0.00 H

ATOM 4107 HE ARG A 259 36.644 7.662 -14.794 1.00 0.00 H

ATOM 4108 HH12 ARG A 259 35.516 3.902 -13.683 1.00 0.00 H

ATOM 4109 HH11 ARG A 259 35.949 5.065 -12.462 1.00 0.00 H

ATOM 4110 HH22 ARG A 259 35.609 4.493 -15.910 1.00 0.00 H

ATOM 4111 HH21 ARG A 259 36.072 6.104 -16.393 1.00 0.00 H

ATOM 4112 H ARG A 259 34.959 7.435 -8.213 1.00 0.00 H

ATOM 4113 HA ARG A 259 34.472 9.755 -9.706 1.00 0.00 H

ATOM 4114 N PHE A 260 32.395 7.261 -10.404 1.00 7.11 N

ATOM 4115 CA PHE A 260 31.080 6.934 -10.950 1.00 6.95 C

ATOM 4116 C PHE A 260 29.960 7.811 -10.364 1.00 7.25 C

ATOM 4117 O PHE A 260 29.165 8.335 -11.139 1.00 6.83 O

ATOM 4118 CB PHE A 260 30.793 5.419 -10.833 1.00 7.19 C

ATOM 4119 CG PHE A 260 29.398 5.004 -11.274 1.00 6.89 C

ATOM 4120 CD1 PHE A 260 29.135 4.755 -12.639 1.00 7.99 C

ATOM 4121 CD2 PHE A 260 28.315 5.064 -10.369 1.00 6.87 C

ATOM 4122 CE1 PHE A 260 27.835 4.511 -13.063 1.00 7.67 C

ATOM 4123 CE2 PHE A 260 27.022 4.844 -10.819 1.00 7.71 C

ATOM 4124 CZ PHE A 260 26.785 4.555 -12.156 1.00 8.31 C

ATOM 4125 H PHE A 260 32.855 6.534 -9.871 1.00 0.00 H

ATOM 4126 HA PHE A 260 31.121 7.161 -12.018 1.00 0.00 H

ATOM 4127 HB3 PHE A 260 30.944 5.080 -9.810 1.00 0.00 H

ATOM 4128 HB2 PHE A 260 31.516 4.869 -11.437 1.00 0.00 H

ATOM 4129 HD1 PHE A 260 29.942 4.752 -13.356 1.00 0.00 H

ATOM 4130 HD2 PHE A 260 28.488 5.304 -9.330 1.00 0.00 H

ATOM 4131 HE1 PHE A 260 27.637 4.297 -14.103 1.00 0.00 H

ATOM 4132 HE2 PHE A 260 26.197 4.895 -10.126 1.00 0.00 H

ATOM 4133 HZ PHE A 260 25.778 4.364 -12.494 1.00 0.00 H

ATOM 4134 N MET A 261 29.940 7.971 -9.026 1.00 7.06 N

ATOM 4135 CA MET A 261 28.976 8.799 -8.290 1.00 7.40 C

ATOM 4136 C MET A 261 29.024 10.277 -8.725 1.00 7.78 C

ATOM 4137 O MET A 261 27.971 10.852 -8.989 1.00 8.28 O

ATOM 4138 CB MET A 261 29.128 8.566 -6.756 1.00 0.00 C

ATOM 4139 CG MET A 261 29.552 9.767 -5.884 1.00 0.00 C

ATOM 4140 SD MET A 261 29.864 9.375 -4.147 1.00 0.00 S

ATOM 4141 CE MET A 261 30.579 10.956 -3.623 1.00 0.00 C

ATOM 4142 HB2 MET A 261 28.171 8.208 -6.374 1.00 0.00 H

ATOM 4143 HB3 MET A 261 29.820 7.745 -6.565 1.00 0.00 H

ATOM 4144 HG2 MET A 261 30.471 10.206 -6.267 1.00 0.00 H

ATOM 4145 HG3 MET A 261 28.788 10.545 -5.915 1.00 0.00 H

ATOM 4146 HE1 MET A 261 30.791 10.937 -2.553 1.00 0.00 H

ATOM 4147 HE2 MET A 261 31.510 11.141 -4.157 1.00 0.00 H

ATOM 4148 HE3 MET A 261 29.900 11.782 -3.827 1.00 0.00 H

ATOM 4149 H MET A 261 30.633 7.494 -8.463 1.00 0.00 H

ATOM 4150 HA MET A 261 27.995 8.419 -8.577 1.00 0.00 H

ATOM 4151 N LYS A 262 30.246 10.824 -8.845 1.00 8.49 N

ATOM 4152 CA LYS A 262 30.563 12.166 -9.340 1.00 9.61 C

ATOM 4153 C LYS A 262 29.958 12.450 -10.725 1.00 9.11 C

ATOM 4154 O LYS A 262 29.282 13.464 -10.894 1.00 9.81 O

ATOM 4155 CB LYS A 262 32.106 12.356 -9.285 1.00 0.00 C

ATOM 4156 CG LYS A 262 32.715 13.387 -10.258 1.00 0.00 C

ATOM 4157 CD LYS A 262 34.245 13.461 -10.192 1.00 0.00 C

ATOM 4158 CE LYS A 262 34.821 14.245 -11.380 1.00 0.00 C

ATOM 4159 NZ LYS A 262 36.285 14.366 -11.290 1.00 0.00 N1+

ATOM 4160 HB2 LYS A 262 32.408 12.575 -8.260 1.00 0.00 H

ATOM 4161 HB3 LYS A 262 32.577 11.404 -9.523 1.00 0.00 H

ATOM 4162 HG2 LYS A 262 32.453 13.133 -11.285 1.00 0.00 H

ATOM 4163 HG3 LYS A 262 32.287 14.373 -10.067 1.00 0.00 H

ATOM 4164 HD2 LYS A 262 34.540 13.935 -9.255 1.00 0.00 H

ATOM 4165 HD3 LYS A 262 34.664 12.454 -10.175 1.00 0.00 H

ATOM 4166 HE2 LYS A 262 34.569 13.751 -12.319 1.00 0.00 H

ATOM 4167 HE3 LYS A 262 34.391 15.247 -11.418 1.00 0.00 H

ATOM 4168 HZ1 LYS A 262 36.531 14.860 -10.444 1.00 0.00 H

ATOM 4169 HZ2 LYS A 262 36.702 13.446 -11.275 1.00 0.00 H

ATOM 4170 HZ3 LYS A 262 36.631 14.878 -12.089 1.00 0.00 H

ATOM 4171 H LYS A 262 31.048 10.259 -8.595 1.00 0.00 H

ATOM 4172 HA LYS A 262 30.115 12.873 -8.640 1.00 0.00 H

ATOM 4173 N ASN A 263 30.225 11.542 -11.677 1.00 8.79 N

ATOM 4174 CA ASN A 263 29.818 11.662 -13.078 1.00 8.63 C

ATOM 4175 C ASN A 263 28.336 11.317 -13.314 1.00 8.52 C

ATOM 4176 O ASN A 263 27.817 11.695 -14.363 1.00 9.63 O

ATOM 4177 CB ASN A 263 30.741 10.771 -13.944 1.00 8.60 C

ATOM 4178 CG ASN A 263 32.197 11.255 -14.012 1.00 9.64 C

ATOM 4179 OD1 ASN A 263 32.471 12.453 -14.004 1.00 10.98 O

ATOM 4180 ND2 ASN A 263 33.144 10.319 -14.106 1.00 10.20 N

ATOM 4181 H ASN A 263 30.784 10.732 -11.443 1.00 0.00 H

ATOM 4182 HA ASN A 263 29.965 12.706 -13.364 1.00 0.00 H

ATOM 4183 HB3 ASN A 263 30.377 10.755 -14.973 1.00 0.00 H

ATOM 4184 HB2 ASN A 263 30.704 9.739 -13.590 1.00 0.00 H

ATOM 4185 HD22 ASN A 263 34.114 10.590 -14.172 1.00 0.00 H

ATOM 4186 HD21 ASN A 263 32.896 9.341 -14.108 1.00 0.00 H

ATOM 4187 N ARG A 264 27.684 10.621 -12.365 1.00 7.81 N

ATOM 4188 CA ARG A 264 26.290 10.191 -12.475 1.00 7.73 C

ATOM 4189 C ARG A 264 25.298 11.184 -11.846 1.00 7.58 C

ATOM 4190 O ARG A 264 24.174 11.264 -12.335 1.00 7.83 O

ATOM 4191 CB ARG A 264 26.156 8.764 -11.902 1.00 7.82 C

ATOM 4192 CG ARG A 264 24.763 8.114 -11.995 1.00 7.72 C

ATOM 4193 CD ARG A 264 24.131 8.187 -13.394 1.00 7.36 C

ATOM 4194 NE ARG A 264 22.986 7.286 -13.528 1.00 7.37 N

ATOM 4195 CZ ARG A 264 23.052 6.043 -14.026 1.00 6.86 C

ATOM 4196 NH1 ARG A 264 24.217 5.528 -14.440 1.00 8.40 N

ATOM 4197 NH2 ARG A 264 21.937 5.313 -14.126 1.00 10.18 N1+

ATOM 4198 H ARG A 264 28.180 10.328 -11.533 1.00 0.00 H

ATOM 4199 HA ARG A 264 26.038 10.134 -13.535 1.00 0.00 H

ATOM 4200 HB3 ARG A 264 26.473 8.758 -10.858 1.00 0.00 H

ATOM 4201 HB2 ARG A 264 26.855 8.116 -12.431 1.00 0.00 H

ATOM 4202 HG3 ARG A 264 24.113 8.660 -11.309 1.00 0.00 H

ATOM 4203 HG2 ARG A 264 24.790 7.090 -11.633 1.00 0.00 H

ATOM 4204 HD3 ARG A 264 24.842 8.153 -14.219 1.00 0.00 H

ATOM 4205 HD2 ARG A 264 23.633 9.150 -13.478 1.00 0.00 H

ATOM 4206 HE ARG A 264 22.092 7.637 -13.190 1.00 0.00 H

ATOM 4207 HH12 ARG A 264 24.238 4.608 -14.874 1.00 0.00 H

ATOM 4208 HH11 ARG A 264 25.067 6.065 -14.375 1.00 0.00 H

ATOM 4209 HH22 ARG A 264 21.976 4.398 -14.560 1.00 0.00 H

ATOM 4210 HH21 ARG A 264 21.041 5.712 -13.852 1.00 0.00 H

ATOM 4211 N VAL A 265 25.708 11.942 -10.814 1.00 7.61 N

ATOM 4212 CA VAL A 265 24.896 13.011 -10.220 1.00 8.34 C

ATOM 4213 C VAL A 265 24.632 14.136 -11.245 1.00 8.95 C

ATOM 4214 O VAL A 265 25.579 14.651 -11.841 1.00 9.50 O

ATOM 4215 CB VAL A 265 25.571 13.585 -8.941 1.00 8.34 C

ATOM 4216 CG1 VAL A 265 25.051 14.960 -8.474 1.00 8.61 C

ATOM 4217 CG2 VAL A 265 25.444 12.594 -7.774 1.00 8.79 C

ATOM 4218 H VAL A 265 26.644 11.833 -10.447 1.00 0.00 H

ATOM 4219 HA VAL A 265 23.939 12.572 -9.931 1.00 0.00 H

ATOM 4220 HB VAL A 265 26.636 13.697 -9.150 1.00 0.00 H

ATOM 4221 HG11 VAL A 265 25.490 15.237 -7.517 1.00 0.00 H

ATOM 4222 HG12 VAL A 265 25.314 15.751 -9.174 1.00 0.00 H

ATOM 4223 HG13 VAL A 265 23.969 14.953 -8.351 1.00 0.00 H

ATOM 4224 HG21 VAL A 265 26.090 12.903 -6.954 1.00 0.00 H

ATOM 4225 HG22 VAL A 265 24.421 12.548 -7.400 1.00 0.00 H

ATOM 4226 HG23 VAL A 265 25.731 11.583 -8.055 1.00 0.00 H

ATOM 4227 N LYS A 266 23.342 14.446 -11.452 1.00 8.98 N

ATOM 4228 CA LYS A 266 22.860 15.303 -12.539 1.00 10.25 C

ATOM 4229 C LYS A 266 22.627 16.764 -12.116 1.00 11.05 C

ATOM 4230 O LYS A 266 22.582 17.624 -12.994 1.00 12.12 O

ATOM 4231 CB LYS A 266 21.554 14.698 -13.103 1.00 10.08 C

ATOM 4232 CG LYS A 266 21.757 13.309 -13.732 1.00 9.97 C

ATOM 4233 CD LYS A 266 20.464 12.663 -14.244 1.00 13.40 C

ATOM 4234 CE LYS A 266 20.731 11.276 -14.847 1.00 13.71 C

ATOM 4235 NZ LYS A 266 19.479 10.562 -15.146 1.00 17.12 N1+

ATOM 4236 H LYS A 266 22.630 13.973 -10.916 1.00 0.00 H

ATOM 4237 HA LYS A 266 23.597 15.317 -13.344 1.00 0.00 H

ATOM 4238 HB3 LYS A 266 21.139 15.362 -13.863 1.00 0.00 H

ATOM 4239 HB2 LYS A 266 20.806 14.636 -12.311 1.00 0.00 H

ATOM 4240 HG3 LYS A 266 22.187 12.642 -12.989 1.00 0.00 H

ATOM 4241 HG2 LYS A 266 22.484 13.378 -14.542 1.00 0.00 H

ATOM 4242 HD3 LYS A 266 19.999 13.309 -14.990 1.00 0.00 H

ATOM 4243 HD2 LYS A 266 19.754 12.578 -13.420 1.00 0.00 H

ATOM 4244 HE3 LYS A 266 21.311 10.670 -14.150 1.00 0.00 H

ATOM 4245 HE2 LYS A 266 21.323 11.364 -15.758 1.00 0.00 H

ATOM 4246 HZ1 LYS A 266 18.951 10.446 -14.293 1.00 0.00 H

ATOM 4247 HZ2 LYS A 266 18.935 11.085 -15.817 1.00 0.00 H

ATOM 4248 HZ3 LYS A 266 19.696 9.649 -15.521 1.00 0.00 H

ATOM 4249 N ASP A 267 22.465 17.015 -10.806 1.00 11.03 N

ATOM 4250 CA ASP A 267 22.083 18.314 -10.230 1.00 11.36 C

ATOM 4251 C ASP A 267 23.232 18.986 -9.449 1.00 11.56 C

ATOM 4252 O ASP A 267 23.036 20.085 -8.932 1.00 11.78 O

ATOM 4253 CB ASP A 267 20.805 18.213 -9.351 1.00 11.34 C

ATOM 4254 CG ASP A 267 20.782 17.084 -8.309 1.00 11.65 C

ATOM 4255 OD1 ASP A 267 21.853 16.506 -8.022 1.00 10.25 O

ATOM 4256 OD2 ASP A 267 19.683 16.836 -7.770 1.00 12.73 O1-

ATOM 4257 H ASP A 267 22.523 16.255 -10.142 1.00 0.00 H

ATOM 4258 HA ASP A 267 21.846 19.007 -11.039 1.00 0.00 H

ATOM 4259 HB3 ASP A 267 19.964 18.046 -10.024 1.00 0.00 H

ATOM 4260 HB2 ASP A 267 20.618 19.157 -8.837 1.00 0.00 H

ATOM 4261 N GLY A 268 24.400 18.325 -9.364 1.00 11.70 N

ATOM 4262 CA GLY A 268 25.582 18.820 -8.658 1.00 12.70 C

ATOM 4263 C GLY A 268 25.458 18.735 -7.126 1.00 12.40 C

ATOM 4264 O GLY A 268 26.277 19.348 -6.441 1.00 13.16 O

ATOM 4265 H GLY A 268 24.482 17.428 -9.819 1.00 0.00 H

ATOM 4266 HA3 GLY A 268 25.786 19.851 -8.950 1.00 0.00 H

ATOM 4267 HA2 GLY A 268 26.442 18.228 -8.969 1.00 0.00 H

ATOM 4268 N ARG A 269 24.461 18.006 -6.582 1.00 11.94 N

ATOM 4269 CA ARG A 269 24.254 17.837 -5.140 1.00 11.50 C

ATOM 4270 C ARG A 269 25.392 17.035 -4.485 1.00 11.18 C

ATOM 4271 O ARG A 269 25.952 16.136 -5.116 1.00 11.47 O

ATOM 4272 CB ARG A 269 22.860 17.217 -4.872 1.00 11.26 C

ATOM 4273 CG ARG A 269 22.748 15.682 -4.990 1.00 10.99 C

ATOM 4274 CD ARG A 269 21.329 15.176 -4.701 1.00 8.84 C

ATOM 4275 NE ARG A 269 21.204 13.737 -4.954 1.00 8.19 N

ATOM 4276 CZ ARG A 269 20.990 13.160 -6.146 1.00 7.50 C

ATOM 4277 NH1 ARG A 269 20.827 13.873 -7.267 1.00 8.70 N

ATOM 4278 NH2 ARG A 269 20.937 11.829 -6.214 1.00 7.19 N1+

ATOM 4279 H ARG A 269 23.802 17.532 -7.186 1.00 0.00 H

ATOM 4280 HA ARG A 269 24.258 18.836 -4.698 1.00 0.00 H

ATOM 4281 HB3 ARG A 269 22.130 17.693 -5.528 1.00 0.00 H

ATOM 4282 HB2 ARG A 269 22.557 17.490 -3.860 1.00 0.00 H

ATOM 4283 HG3 ARG A 269 23.393 15.250 -4.224 1.00 0.00 H

ATOM 4284 HG2 ARG A 269 23.115 15.302 -5.944 1.00 0.00 H

ATOM 4285 HD3 ARG A 269 20.542 15.778 -5.157 1.00 0.00 H

ATOM 4286 HD2 ARG A 269 21.180 15.250 -3.623 1.00 0.00 H

ATOM 4287 HE ARG A 269 21.333 13.135 -4.151 1.00 0.00 H

ATOM 4288 HH12 ARG A 269 20.644 13.414 -8.146 1.00 0.00 H

ATOM 4289 HH11 ARG A 269 20.893 14.888 -7.250 1.00 0.00 H

ATOM 4290 HH22 ARG A 269 20.736 11.363 -7.092 1.00 0.00 H

ATOM 4291 HH21 ARG A 269 21.060 11.281 -5.370 1.00 0.00 H

ATOM 4292 N GLU A 270 25.691 17.362 -3.219 1.00 10.75 N

ATOM 4293 CA GLU A 270 26.717 16.669 -2.450 1.00 10.88 C

ATOM 4294 C GLU A 270 26.190 15.341 -1.887 1.00 10.33 C

ATOM 4295 O GLU A 270 25.209 15.333 -1.143 1.00 10.79 O

ATOM 4296 CB GLU A 270 27.308 17.609 -1.387 1.00 11.41 C

ATOM 4297 CG GLU A 270 28.399 16.930 -0.534 1.00 14.93 C

ATOM 4298 CD GLU A 270 29.310 17.907 0.204 1.00 18.60 C

ATOM 4299 OE1 GLU A 270 28.909 19.076 0.392 1.00 22.78 O

ATOM 4300 OE2 GLU A 270 30.414 17.452 0.576 1.00 17.84 O1-

ATOM 4301 H GLU A 270 25.195 18.106 -2.751 1.00 0.00 H

ATOM 4302 HA GLU A 270 27.542 16.441 -3.130 1.00 0.00 H

ATOM 4303 HB3 GLU A 270 26.519 17.991 -0.737 1.00 0.00 H

ATOM 4304 HB2 GLU A 270 27.727 18.478 -1.897 1.00 0.00 H

ATOM 4305 HG3 GLU A 270 29.034 16.317 -1.176 1.00 0.00 H

ATOM 4306 HG2 GLU A 270 27.951 16.254 0.194 1.00 0.00 H

ATOM 4307 N ILE A 271 26.880 14.257 -2.268 1.00 10.15 N

ATOM 4308 CA ILE A 271 26.583 12.886 -1.878 1.00 9.49 C

ATOM 4309 C ILE A 271 27.549 12.463 -0.759 1.00 9.58 C

ATOM 4310 O ILE A 271 28.764 12.518 -0.960 1.00 10.18 O

ATOM 4311 CB ILE A 271 26.744 11.934 -3.104 1.00 9.84 C

ATOM 4312 CG1 ILE A 271 25.725 12.249 -4.221 1.00 9.71 C

ATOM 4313 CG2 ILE A 271 26.739 10.421 -2.797 1.00 10.02 C

ATOM 4314 CD1 ILE A 271 24.246 12.095 -3.839 1.00 9.84 C

ATOM 4315 H ILE A 271 27.679 14.372 -2.873 1.00 0.00 H

ATOM 4316 HA ILE A 271 25.562 12.823 -1.513 1.00 0.00 H

ATOM 4317 HB ILE A 271 27.721 12.144 -3.538 1.00 0.00 H

ATOM 4318 HG13 ILE A 271 25.937 11.609 -5.077 1.00 0.00 H

ATOM 4319 HG12 ILE A 271 25.879 13.269 -4.575 1.00 0.00 H

ATOM 4320 HG21 ILE A 271 26.884 9.842 -3.709 1.00 0.00 H

ATOM 4321 HG22 ILE A 271 27.529 10.123 -2.109 1.00 0.00 H

ATOM 4322 HG23 ILE A 271 25.790 10.110 -2.376 1.00 0.00 H

ATOM 4323 HD11 ILE A 271 23.621 12.134 -4.731 1.00 0.00 H

ATOM 4324 HD12 ILE A 271 24.042 11.149 -3.340 1.00 0.00 H

ATOM 4325 HD13 ILE A 271 23.921 12.902 -3.183 1.00 0.00 H

ATOM 4326 N MET A 272 26.989 12.027 0.382 1.00 8.46 N

ATOM 4327 CA MET A 272 27.749 11.397 1.463 1.00 8.84 C

ATOM 4328 C MET A 272 27.852 9.891 1.201 1.00 7.74 C

ATOM 4329 O MET A 272 26.885 9.283 0.750 1.00 7.07 O

ATOM 4330 CB MET A 272 27.053 11.646 2.818 1.00 8.61 C

ATOM 4331 CG MET A 272 27.890 11.214 4.047 1.00 10.28 C

ATOM 4332 SD MET A 272 26.940 10.791 5.532 1.00 12.60 S

ATOM 4333 CE MET A 272 26.360 9.149 5.053 1.00 15.74 C

ATOM 4334 H MET A 272 25.982 12.007 0.469 1.00 0.00 H

ATOM 4335 HA MET A 272 28.747 11.837 1.507 1.00 0.00 H

ATOM 4336 HB3 MET A 272 26.076 11.160 2.835 1.00 0.00 H

ATOM 4337 HB2 MET A 272 26.844 12.711 2.903 1.00 0.00 H

ATOM 4338 HG3 MET A 272 28.595 12.003 4.300 1.00 0.00 H

ATOM 4339 HG2 MET A 272 28.513 10.348 3.841 1.00 0.00 H

ATOM 4340 HE1 MET A 272 25.854 8.674 5.892 1.00 0.00 H

ATOM 4341 HE2 MET A 272 25.671 9.210 4.212 1.00 0.00 H

ATOM 4342 HE3 MET A 272 27.201 8.525 4.758 1.00 0.00 H

ATOM 4343 N THR A 273 28.988 9.295 1.564 1.00 6.03 N

ATOM 4344 CA THR A 273 29.127 7.848 1.655 1.00 5.80 C

ATOM 4345 C THR A 273 29.752 7.513 3.019 1.00 5.70 C

ATOM 4346 O THR A 273 30.777 8.085 3.378 1.00 5.60 O

ATOM 4347 CB THR A 273 29.994 7.288 0.503 1.00 5.71 C

ATOM 4348 OG1 THR A 273 31.206 8.000 0.387 1.00 7.41 O

ATOM 4349 CG2 THR A 273 29.299 7.349 -0.865 1.00 6.56 C

ATOM 4350 H THR A 273 29.773 9.842 1.895 1.00 0.00 H

ATOM 4351 HA THR A 273 28.152 7.375 1.602 1.00 0.00 H

ATOM 4352 HB THR A 273 30.244 6.250 0.720 1.00 0.00 H

ATOM 4353 HG1 THR A 273 31.614 7.774 -0.453 1.00 0.00 H

ATOM 4354 HG21 THR A 273 29.914 6.895 -1.643 1.00 0.00 H

ATOM 4355 HG22 THR A 273 28.349 6.817 -0.844 1.00 0.00 H

ATOM 4356 HG23 THR A 273 29.093 8.377 -1.165 1.00 0.00 H

ATOM 4357 N VAL A 274 29.111 6.596 3.761 1.00 4.77 N

ATOM 4358 CA VAL A 274 29.617 6.062 5.025 1.00 5.24 C

ATOM 4359 C VAL A 274 29.903 4.563 4.849 1.00 4.78 C

ATOM 4360 O VAL A 274 28.988 3.792 4.575 1.00 5.86 O

ATOM 4361 CB VAL A 274 28.673 6.327 6.236 1.00 4.73 C

ATOM 4362 CG1 VAL A 274 27.223 5.845 6.053 1.00 5.57 C

ATOM 4363 CG2 VAL A 274 29.238 5.746 7.549 1.00 6.24 C

ATOM 4364 H VAL A 274 28.261 6.173 3.409 1.00 0.00 H

ATOM 4365 HA VAL A 274 30.553 6.554 5.270 1.00 0.00 H

ATOM 4366 HB VAL A 274 28.635 7.410 6.357 1.00 0.00 H

ATOM 4367 HG11 VAL A 274 26.574 6.209 6.846 1.00 0.00 H

ATOM 4368 HG12 VAL A 274 26.793 6.175 5.108 1.00 0.00 H

ATOM 4369 HG13 VAL A 274 27.171 4.764 6.097 1.00 0.00 H

ATOM 4370 HG21 VAL A 274 28.695 6.133 8.411 1.00 0.00 H

ATOM 4371 HG22 VAL A 274 29.163 4.659 7.578 1.00 0.00 H

ATOM 4372 HG23 VAL A 274 30.290 5.994 7.686 1.00 0.00 H

ATOM 4373 N GLY A 275 31.183 4.185 4.970 1.00 5.06 N

ATOM 4374 CA GLY A 275 31.628 2.816 4.745 1.00 4.41 C

ATOM 4375 C GLY A 275 31.564 2.035 6.055 1.00 4.21 C

ATOM 4376 O GLY A 275 32.174 2.437 7.043 1.00 3.68 O

ATOM 4377 H GLY A 275 31.888 4.873 5.202 1.00 0.00 H

ATOM 4378 HA3 GLY A 275 32.660 2.831 4.396 1.00 0.00 H

ATOM 4379 HA2 GLY A 275 31.043 2.323 3.972 1.00 0.00 H

ATOM 4380 N GLU A 276 30.910 0.865 6.032 1.00 4.24 N

ATOM 4381 CA GLU A 276 31.047 -0.160 7.062 1.00 5.09 C

ATOM 4382 C GLU A 276 32.300 -0.991 6.732 1.00 4.94 C

ATOM 4383 O GLU A 276 32.182 -2.076 6.176 1.00 5.77 O

ATOM 4384 CB GLU A 276 29.766 -1.021 7.120 1.00 5.00 C

ATOM 4385 CG GLU A 276 29.788 -2.067 8.261 1.00 5.08 C

ATOM 4386 CD GLU A 276 28.735 -3.164 8.121 1.00 5.61 C

ATOM 4387 OE1 GLU A 276 28.481 -3.577 6.973 1.00 5.26 O

ATOM 4388 OE2 GLU A 276 28.191 -3.575 9.167 1.00 6.39 O1-

ATOM 4389 H GLU A 276 30.412 0.581 5.196 1.00 0.00 H

ATOM 4390 HA GLU A 276 31.188 0.303 8.037 1.00 0.00 H

ATOM 4391 HB3 GLU A 276 29.623 -1.504 6.154 1.00 0.00 H

ATOM 4392 HB2 GLU A 276 28.896 -0.375 7.248 1.00 0.00 H

ATOM 4393 HG3 GLU A 276 29.654 -1.557 9.215 1.00 0.00 H

ATOM 4394 HG2 GLU A 276 30.749 -2.576 8.321 1.00 0.00 H

ATOM 4395 N VAL A 277 33.489 -0.455 7.034 1.00 5.92 N

ATOM 4396 CA VAL A 277 34.769 -1.123 6.769 1.00 6.88 C

ATOM 4397 C VAL A 277 35.199 -2.092 7.891 1.00 6.52 C

ATOM 4398 O VAL A 277 36.155 -2.838 7.686 1.00 6.76 O

ATOM 4399 CB VAL A 277 35.904 -0.091 6.537 1.00 0.00 C

ATOM 4400 CG1 VAL A 277 35.738 0.627 5.188 1.00 0.00 C

ATOM 4401 CG2 VAL A 277 36.047 0.910 7.693 1.00 0.00 C

ATOM 4402 HB VAL A 277 36.855 -0.624 6.465 1.00 0.00 H

ATOM 4403 HG11 VAL A 277 36.554 1.327 5.009 1.00 0.00 H

ATOM 4404 HG12 VAL A 277 35.733 -0.084 4.360 1.00 0.00 H

ATOM 4405 HG13 VAL A 277 34.806 1.192 5.147 1.00 0.00 H

ATOM 4406 HG21 VAL A 277 36.804 1.666 7.484 1.00 0.00 H

ATOM 4407 HG22 VAL A 277 35.101 1.405 7.874 1.00 0.00 H

ATOM 4408 HG23 VAL A 277 36.321 0.419 8.624 1.00 0.00 H

ATOM 4409 H VAL A 277 33.535 0.457 7.467 1.00 0.00 H

ATOM 4410 HA VAL A 277 34.678 -1.722 5.861 1.00 0.00 H

ATOM 4411 N ALA A 278 34.488 -2.071 9.036 1.00 6.64 N

ATOM 4412 CA ALA A 278 34.683 -2.908 10.227 1.00 6.86 C

ATOM 4413 C ALA A 278 35.949 -2.600 11.052 1.00 6.83 C

ATOM 4414 O ALA A 278 36.115 -3.180 12.123 1.00 6.55 O

ATOM 4415 CB ALA A 278 34.590 -4.408 9.873 1.00 0.00 C

ATOM 4416 HB1 ALA A 278 34.574 -5.024 10.773 1.00 0.00 H

ATOM 4417 HB2 ALA A 278 33.676 -4.622 9.318 1.00 0.00 H

ATOM 4418 HB3 ALA A 278 35.434 -4.745 9.272 1.00 0.00 H

ATOM 4419 H ALA A 278 33.725 -1.413 9.102 1.00 0.00 H

ATOM 4420 HA ALA A 278 33.842 -2.667 10.871 1.00 0.00 H

ATOM 4421 N HIS A 279 36.804 -1.694 10.555 1.00 31.02 N

ATOM 4422 CA HIS A 279 38.107 -1.325 11.097 1.00 32.28 C

ATOM 4423 C HIS A 279 38.693 -0.240 10.183 1.00 31.44 C

ATOM 4424 O HIS A 279 38.786 -0.461 8.975 1.00 32.07 O

ATOM 4425 CB HIS A 279 39.032 -2.569 11.172 1.00 0.00 C

ATOM 4426 CG HIS A 279 40.506 -2.313 11.390 1.00 0.00 C

ATOM 4427 ND1 HIS A 279 40.993 -1.418 12.330 1.00 0.00 N

ATOM 4428 CD2 HIS A 279 41.617 -2.826 10.758 1.00 0.00 C

ATOM 4429 CE1 HIS A 279 42.325 -1.424 12.224 1.00 0.00 C

ATOM 4430 NE2 HIS A 279 42.775 -2.261 11.298 1.00 0.00 N

ATOM 4431 HB2 HIS A 279 38.708 -3.231 11.975 1.00 0.00 H

ATOM 4432 HB3 HIS A 279 38.940 -3.146 10.251 1.00 0.00 H

ATOM 4433 HD2 HIS A 279 41.674 -3.547 9.956 1.00 0.00 H

ATOM 4434 HE1 HIS A 279 42.973 -0.816 12.838 1.00 0.00 H

ATOM 4435 H HIS A 279 36.589 -1.276 9.661 1.00 0.00 H

ATOM 4436 HA HIS A 279 37.969 -0.913 12.098 1.00 0.00 H

ATOM 4437 HD1 HIS A 279 40.449 -0.868 12.978 1.00 0.00 H

ATOM 4438 N GLY A 280 39.088 0.892 10.784 1.00 31.02 N

ATOM 4439 CA GLY A 280 39.737 1.994 10.090 1.00 30.54 C

ATOM 4440 C GLY A 280 40.949 2.441 10.915 1.00 30.29 C

ATOM 4441 O GLY A 280 40.795 2.936 12.030 1.00 30.82 O

ATOM 4442 HA3 GLY A 280 39.035 2.824 10.013 1.00 0.00 H

ATOM 4443 H GLY A 280 38.951 1.009 11.778 1.00 0.00 H

ATOM 4444 HA2 GLY A 280 40.041 1.737 9.075 1.00 0.00 H

ATOM 4445 N SER A 281 42.139 2.354 10.304 1.00 27.98 N

ATOM 4446 CA SER A 281 43.398 3.003 10.710 1.00 27.56 C

ATOM 4447 C SER A 281 44.134 3.474 9.441 1.00 27.51 C

ATOM 4448 O SER A 281 44.472 4.651 9.310 1.00 28.08 O

ATOM 4449 CB SER A 281 44.245 2.045 11.568 1.00 0.00 C

ATOM 4450 OG SER A 281 43.863 2.155 12.922 1.00 0.00 O

ATOM 4451 HB2 SER A 281 44.150 1.011 11.233 1.00 0.00 H

ATOM 4452 HB3 SER A 281 45.303 2.304 11.508 1.00 0.00 H

ATOM 4453 HG SER A 281 42.931 1.934 12.993 1.00 0.00 H

ATOM 4454 H SER A 281 42.176 1.875 9.414 1.00 0.00 H

ATOM 4455 HA SER A 281 43.177 3.907 11.285 1.00 0.00 H

ATOM 4456 N ASP A 282 44.119 2.563 8.455 1.00 9.78 N

ATOM 4457 CA ASP A 282 43.853 2.703 7.010 1.00 9.35 C

ATOM 4458 C ASP A 282 42.697 3.669 6.626 1.00 8.64 C

ATOM 4459 O ASP A 282 42.531 3.963 5.442 1.00 8.87 O

ATOM 4460 CB ASP A 282 43.508 1.304 6.425 1.00 0.00 C

ATOM 4461 CG ASP A 282 42.601 0.470 7.346 1.00 0.00 C

ATOM 4462 OD1 ASP A 282 41.371 0.526 7.139 1.00 0.00 O

ATOM 4463 OD2 ASP A 282 43.152 -0.142 8.288 1.00 0.00 O1-

ATOM 4464 HB2 ASP A 282 43.048 1.363 5.437 1.00 0.00 H

ATOM 4465 HB3 ASP A 282 44.421 0.734 6.247 1.00 0.00 H

ATOM 4466 HA ASP A 282 44.765 3.072 6.540 1.00 0.00 H

ATOM 4467 H ASP A 282 43.945 1.611 8.756 1.00 0.00 H

ATOM 4468 N ASN A 283 41.933 4.137 7.631 1.00 8.32 N

ATOM 4469 CA ASN A 283 40.898 5.170 7.620 1.00 8.08 C

ATOM 4470 C ASN A 283 41.298 6.435 6.847 1.00 8.15 C

ATOM 4471 O ASN A 283 40.431 7.015 6.209 1.00 8.27 O

ATOM 4472 CB ASN A 283 40.453 5.454 9.093 1.00 0.00 C

ATOM 4473 CG ASN A 283 40.624 6.876 9.646 1.00 0.00 C

ATOM 4474 OD1 ASN A 283 39.680 7.660 9.660 1.00 0.00 O

ATOM 4475 ND2 ASN A 283 41.838 7.212 10.084 1.00 0.00 N

ATOM 4476 HB2 ASN A 283 39.405 5.174 9.209 1.00 0.00 H

ATOM 4477 HB3 ASN A 283 40.999 4.818 9.779 1.00 0.00 H

ATOM 4478 HD22 ASN A 283 41.996 8.141 10.446 1.00 0.00 H

ATOM 4479 HD21 ASN A 283 42.600 6.551 10.064 1.00 0.00 H

ATOM 4480 H ASN A 283 42.147 3.790 8.554 1.00 0.00 H

ATOM 4481 HA ASN A 283 40.041 4.747 7.095 1.00 0.00 H

ATOM 4482 N ALA A 284 42.574 6.850 6.937 1.00 8.24 N

ATOM 4483 CA ALA A 284 43.090 8.041 6.269 1.00 8.54 C

ATOM 4484 C ALA A 284 43.065 7.910 4.740 1.00 7.70 C

ATOM 4485 O ALA A 284 42.691 8.870 4.073 1.00 7.67 O

ATOM 4486 CB ALA A 284 44.504 8.342 6.780 1.00 0.00 C

ATOM 4487 HB1 ALA A 284 44.912 9.238 6.310 1.00 0.00 H

ATOM 4488 HB2 ALA A 284 44.501 8.513 7.858 1.00 0.00 H

ATOM 4489 HB3 ALA A 284 45.188 7.517 6.576 1.00 0.00 H

ATOM 4490 H ALA A 284 43.233 6.323 7.492 1.00 0.00 H

ATOM 4491 HA ALA A 284 42.448 8.881 6.544 1.00 0.00 H

ATOM 4492 N LEU A 285 43.384 6.713 4.218 1.00 7.32 N

ATOM 4493 CA LEU A 285 43.275 6.387 2.795 1.00 7.46 C

ATOM 4494 C LEU A 285 41.814 6.331 2.310 1.00 7.27 C

ATOM 4495 O LEU A 285 41.569 6.678 1.158 1.00 7.04 O

ATOM 4496 CB LEU A 285 43.995 5.055 2.496 1.00 7.61 C

ATOM 4497 CG LEU A 285 45.515 5.049 2.764 1.00 8.56 C

ATOM 4498 CD1 LEU A 285 46.075 3.623 2.644 1.00 9.20 C

ATOM 4499 CD2 LEU A 285 46.290 6.029 1.861 1.00 9.56 C

ATOM 4500 H LEU A 285 43.644 5.954 4.831 1.00 0.00 H

ATOM 4501 HA LEU A 285 43.769 7.183 2.237 1.00 0.00 H

ATOM 4502 HB3 LEU A 285 43.829 4.778 1.453 1.00 0.00 H

ATOM 4503 HB2 LEU A 285 43.529 4.269 3.088 1.00 0.00 H

ATOM 4504 HG LEU A 285 45.673 5.354 3.799 1.00 0.00 H

ATOM 4505 HD11 LEU A 285 47.085 3.564 3.050 1.00 0.00 H

ATOM 4506 HD12 LEU A 285 45.462 2.907 3.194 1.00 0.00 H

ATOM 4507 HD13 LEU A 285 46.112 3.297 1.604 1.00 0.00 H

ATOM 4508 HD21 LEU A 285 47.118 5.542 1.344 1.00 0.00 H

ATOM 4509 HD22 LEU A 285 45.657 6.473 1.097 1.00 0.00 H

ATOM 4510 HD23 LEU A 285 46.712 6.844 2.449 1.00 0.00 H

ATOM 4511 N TYR A 286 40.877 5.930 3.187 1.00 6.64 N

ATOM 4512 CA TYR A 286 39.443 5.925 2.904 1.00 6.69 C

ATOM 4513 C TYR A 286 38.805 7.326 2.994 1.00 6.30 C

ATOM 4514 O TYR A 286 38.151 7.742 2.042 1.00 6.58 O

ATOM 4515 CB TYR A 286 38.720 4.931 3.844 1.00 6.49 C

ATOM 4516 CG TYR A 286 38.889 3.452 3.533 1.00 5.57 C

ATOM 4517 CD1 TYR A 286 38.505 2.948 2.271 1.00 6.25 C

ATOM 4518 CD2 TYR A 286 39.364 2.563 4.521 1.00 6.64 C

ATOM 4519 CE1 TYR A 286 38.598 1.570 1.997 1.00 6.28 C

ATOM 4520 CE2 TYR A 286 39.445 1.183 4.252 1.00 6.54 C

ATOM 4521 CZ TYR A 286 39.058 0.687 2.992 1.00 7.31 C

ATOM 4522 OH TYR A 286 39.116 -0.649 2.737 1.00 7.70 O

ATOM 4523 H TYR A 286 41.153 5.658 4.119 1.00 0.00 H

ATOM 4524 HA TYR A 286 39.304 5.584 1.877 1.00 0.00 H

ATOM 4525 HB3 TYR A 286 37.646 5.102 3.793 1.00 0.00 H

ATOM 4526 HB2 TYR A 286 38.992 5.125 4.881 1.00 0.00 H

ATOM 4527 HD1 TYR A 286 38.123 3.612 1.510 1.00 0.00 H

ATOM 4528 HD2 TYR A 286 39.652 2.929 5.496 1.00 0.00 H

ATOM 4529 HE1 TYR A 286 38.301 1.192 1.030 1.00 0.00 H

ATOM 4530 HE2 TYR A 286 39.795 0.508 5.019 1.00 0.00 H

ATOM 4531 HH TYR A 286 39.359 -1.172 3.511 1.00 0.00 H

ATOM 4532 N THR A 287 38.926 7.994 4.151 1.00 5.79 N

ATOM 4533 CA THR A 287 38.060 9.109 4.553 1.00 6.22 C

ATOM 4534 C THR A 287 38.613 10.515 4.260 1.00 6.21 C

ATOM 4535 O THR A 287 37.817 11.454 4.307 1.00 6.36 O

ATOM 4536 CB THR A 287 37.705 9.049 6.061 1.00 6.08 C

ATOM 4537 OG1 THR A 287 38.816 9.301 6.893 1.00 7.63 O

ATOM 4538 CG2 THR A 287 37.063 7.729 6.490 1.00 6.77 C

ATOM 4539 H THR A 287 39.510 7.610 4.885 1.00 0.00 H

ATOM 4540 HA THR A 287 37.117 9.026 4.018 1.00 0.00 H

ATOM 4541 HB THR A 287 36.976 9.834 6.276 1.00 0.00 H

ATOM 4542 HG1 THR A 287 39.406 8.542 6.858 1.00 0.00 H

ATOM 4543 HG21 THR A 287 36.767 7.779 7.537 1.00 0.00 H

ATOM 4544 HG22 THR A 287 36.166 7.541 5.906 1.00 0.00 H

ATOM 4545 HG23 THR A 287 37.730 6.876 6.368 1.00 0.00 H

ATOM 4546 N SER A 288 39.914 10.657 3.935 1.00 6.12 N

ATOM 4547 CA SER A 288 40.509 11.914 3.456 1.00 7.21 C

ATOM 4548 C SER A 288 39.780 12.416 2.198 1.00 7.69 C

ATOM 4549 O SER A 288 39.601 11.640 1.260 1.00 7.66 O

ATOM 4550 CB SER A 288 42.015 11.697 3.202 1.00 7.21 C

ATOM 4551 OG SER A 288 42.649 12.742 2.490 1.00 8.00 O

ATOM 4552 H SER A 288 40.517 9.847 3.924 1.00 0.00 H

ATOM 4553 HA SER A 288 40.403 12.666 4.239 1.00 0.00 H

ATOM 4554 HB3 SER A 288 42.148 10.794 2.616 1.00 0.00 H

ATOM 4555 HB2 SER A 288 42.540 11.551 4.147 1.00 0.00 H

ATOM 4556 HG SER A 288 42.657 13.537 3.042 1.00 0.00 H

ATOM 4557 N ALA A 289 39.361 13.692 2.212 1.00 8.36 N

ATOM 4558 CA ALA A 289 38.631 14.323 1.111 1.00 9.30 C

ATOM 4559 C ALA A 289 39.417 14.337 -0.214 1.00 9.53 C

ATOM 4560 O ALA A 289 38.815 14.127 -1.267 1.00 10.70 O

ATOM 4561 CB ALA A 289 38.218 15.743 1.523 1.00 9.15 C

ATOM 4562 H ALA A 289 39.526 14.266 3.028 1.00 0.00 H

ATOM 4563 HA ALA A 289 37.721 13.742 0.950 1.00 0.00 H

ATOM 4564 HB1 ALA A 289 37.593 16.208 0.759 1.00 0.00 H

ATOM 4565 HB2 ALA A 289 37.647 15.734 2.452 1.00 0.00 H

ATOM 4566 HB3 ALA A 289 39.087 16.384 1.671 1.00 0.00 H

ATOM 4567 N ALA A 290 40.746 14.525 -0.123 1.00 10.14 N

ATOM 4568 CA ALA A 290 41.691 14.487 -1.238 1.00 10.83 C

ATOM 4569 C ALA A 290 41.871 13.104 -1.893 1.00 10.29 C

ATOM 4570 O ALA A 290 42.272 13.060 -3.056 1.00 10.81 O

ATOM 4571 CB ALA A 290 43.044 15.024 -0.750 1.00 0.00 C

ATOM 4572 HB1 ALA A 290 43.789 15.017 -1.547 1.00 0.00 H

ATOM 4573 HB2 ALA A 290 42.952 16.054 -0.403 1.00 0.00 H

ATOM 4574 HB3 ALA A 290 43.434 14.430 0.078 1.00 0.00 H

ATOM 4575 H ALA A 290 41.152 14.718 0.785 1.00 0.00 H

ATOM 4576 HA ALA A 290 41.316 15.163 -2.010 1.00 0.00 H

ATOM 4577 N ARG A 291 41.571 12.010 -1.167 1.00 9.12 N

ATOM 4578 CA ARG A 291 41.626 10.641 -1.694 1.00 8.84 C

ATOM 4579 C ARG A 291 40.482 10.308 -2.660 1.00 8.80 C

ATOM 4580 O ARG A 291 40.683 9.444 -3.512 1.00 8.15 O

ATOM 4581 CB ARG A 291 41.631 9.623 -0.538 1.00 8.97 C

ATOM 4582 CG ARG A 291 42.929 9.618 0.286 1.00 9.40 C

ATOM 4583 CD ARG A 291 44.104 8.924 -0.415 1.00 10.10 C

ATOM 4584 NE ARG A 291 43.807 7.508 -0.663 1.00 9.57 N

ATOM 4585 CZ ARG A 291 44.600 6.635 -1.299 1.00 11.00 C

ATOM 4586 NH1 ARG A 291 45.812 6.989 -1.748 1.00 10.20 N

ATOM 4587 NH2 ARG A 291 44.157 5.390 -1.492 1.00 9.53 N1+

ATOM 4588 H ARG A 291 41.241 12.115 -0.218 1.00 0.00 H

ATOM 4589 HA ARG A 291 42.553 10.538 -2.259 1.00 0.00 H

ATOM 4590 HB3 ARG A 291 41.450 8.618 -0.921 1.00 0.00 H

ATOM 4591 HB2 ARG A 291 40.788 9.815 0.122 1.00 0.00 H

ATOM 4592 HG3 ARG A 291 42.723 9.056 1.195 1.00 0.00 H

ATOM 4593 HG2 ARG A 291 43.214 10.617 0.618 1.00 0.00 H

ATOM 4594 HD3 ARG A 291 45.026 9.049 0.152 1.00 0.00 H

ATOM 4595 HD2 ARG A 291 44.272 9.378 -1.390 1.00 0.00 H

ATOM 4596 HE ARG A 291 42.901 7.183 -0.339 1.00 0.00 H

ATOM 4597 HH12 ARG A 291 46.401 6.323 -2.227 1.00 0.00 H

ATOM 4598 HH11 ARG A 291 46.143 7.932 -1.605 1.00 0.00 H

ATOM 4599 HH22 ARG A 291 44.729 4.701 -1.958 1.00 0.00 H

ATOM 4600 HH21 ARG A 291 43.190 5.174 -1.267 1.00 0.00 H

ATOM 4601 N TYR A 292 39.324 10.978 -2.508 1.00 9.10 N

ATOM 4602 CA TYR A 292 38.102 10.774 -3.292 1.00 9.00 C

ATOM 4603 C TYR A 292 37.634 9.308 -3.246 1.00 8.41 C

ATOM 4604 O TYR A 292 37.582 8.645 -4.279 1.00 8.14 O

ATOM 4605 CB TYR A 292 38.289 11.290 -4.739 1.00 0.00 C

ATOM 4606 CG TYR A 292 38.559 12.778 -4.865 1.00 0.00 C

ATOM 4607 CD1 TYR A 292 37.600 13.715 -4.427 1.00 0.00 C

ATOM 4608 CD2 TYR A 292 39.764 13.230 -5.441 1.00 0.00 C

ATOM 4609 CE1 TYR A 292 37.839 15.095 -4.574 1.00 0.00 C

ATOM 4610 CE2 TYR A 292 40.003 14.610 -5.589 1.00 0.00 C

ATOM 4611 CZ TYR A 292 39.038 15.542 -5.160 1.00 0.00 C

ATOM 4612 OH TYR A 292 39.263 16.879 -5.311 1.00 0.00 O

ATOM 4613 HB2 TYR A 292 39.086 10.738 -5.238 1.00 0.00 H

ATOM 4614 HB3 TYR A 292 37.394 11.088 -5.324 1.00 0.00 H

ATOM 4615 HD1 TYR A 292 36.678 13.382 -3.978 1.00 0.00 H

ATOM 4616 HD2 TYR A 292 40.510 12.523 -5.773 1.00 0.00 H

ATOM 4617 HE1 TYR A 292 37.099 15.806 -4.238 1.00 0.00 H

ATOM 4618 HE2 TYR A 292 40.928 14.947 -6.032 1.00 0.00 H

ATOM 4619 HH TYR A 292 40.119 17.076 -5.699 1.00 0.00 H

ATOM 4620 H TYR A 292 39.265 11.686 -1.789 1.00 0.00 H

ATOM 4621 HA TYR A 292 37.320 11.364 -2.812 1.00 0.00 H

ATOM 4622 N GLU A 293 37.331 8.834 -2.030 1.00 7.55 N

ATOM 4623 CA GLU A 293 36.824 7.493 -1.767 1.00 7.07 C

ATOM 4624 C GLU A 293 35.518 7.638 -0.984 1.00 6.57 C

ATOM 4625 O GLU A 293 34.473 7.831 -1.606 1.00 7.46 O

ATOM 4626 CB GLU A 293 37.904 6.646 -1.056 1.00 7.27 C

ATOM 4627 CG GLU A 293 39.007 6.172 -2.007 1.00 7.14 C

ATOM 4628 CD GLU A 293 40.066 5.304 -1.332 1.00 7.78 C

ATOM 4629 OE1 GLU A 293 39.677 4.407 -0.552 1.00 7.38 O

ATOM 4630 OE2 GLU A 293 41.262 5.531 -1.619 1.00 8.32 O1-

ATOM 4631 H GLU A 293 37.419 9.438 -1.226 1.00 0.00 H

ATOM 4632 HA GLU A 293 36.555 6.986 -2.696 1.00 0.00 H

ATOM 4633 HB3 GLU A 293 37.450 5.788 -0.556 1.00 0.00 H

ATOM 4634 HB2 GLU A 293 38.384 7.245 -0.288 1.00 0.00 H

ATOM 4635 HG3 GLU A 293 39.493 7.032 -2.467 1.00 0.00 H

ATOM 4636 HG2 GLU A 293 38.556 5.587 -2.804 1.00 0.00 H

ATOM 4637 N VAL A 294 35.597 7.550 0.350 1.00 6.65 N

ATOM 4638 CA VAL A 294 34.445 7.596 1.242 1.00 5.67 C

ATOM 4639 C VAL A 294 34.467 8.914 2.049 1.00 5.80 C

ATOM 4640 O VAL A 294 35.530 9.520 2.191 1.00 5.88 O

ATOM 4641 CB VAL A 294 34.455 6.324 2.144 1.00 0.00 C

ATOM 4642 CG1 VAL A 294 35.200 6.452 3.470 1.00 0.00 C

ATOM 4643 CG2 VAL A 294 33.058 5.748 2.403 1.00 0.00 C

ATOM 4644 HB VAL A 294 34.981 5.548 1.583 1.00 0.00 H

ATOM 4645 HG11 VAL A 294 35.304 5.467 3.918 1.00 0.00 H

ATOM 4646 HG12 VAL A 294 36.193 6.855 3.333 1.00 0.00 H

ATOM 4647 HG13 VAL A 294 34.683 7.099 4.171 1.00 0.00 H

ATOM 4648 HG21 VAL A 294 33.126 4.769 2.877 1.00 0.00 H

ATOM 4649 HG22 VAL A 294 32.493 6.387 3.072 1.00 0.00 H

ATOM 4650 HG23 VAL A 294 32.495 5.622 1.479 1.00 0.00 H

ATOM 4651 H VAL A 294 36.499 7.448 0.798 1.00 0.00 H

ATOM 4652 HA VAL A 294 33.541 7.584 0.648 1.00 0.00 H

ATOM 4653 N SER A 295 33.300 9.359 2.543 1.00 5.56 N

ATOM 4654 CA SER A 295 33.189 10.579 3.355 1.00 5.14 C

ATOM 4655 C SER A 295 33.627 10.342 4.812 1.00 5.43 C

ATOM 4656 O SER A 295 34.191 11.246 5.423 1.00 4.90 O

ATOM 4657 CB SER A 295 31.742 11.118 3.328 1.00 5.65 C

ATOM 4658 OG SER A 295 31.314 11.380 2.008 1.00 6.20 O

ATOM 4659 H SER A 295 32.442 8.849 2.377 1.00 0.00 H

ATOM 4660 HA SER A 295 33.839 11.347 2.931 1.00 0.00 H

ATOM 4661 HB3 SER A 295 31.685 12.054 3.876 1.00 0.00 H

ATOM 4662 HB2 SER A 295 31.038 10.452 3.820 1.00 0.00 H

ATOM 4663 HG SER A 295 31.791 12.144 1.677 1.00 0.00 H

ATOM 4664 N GLU A 296 33.348 9.135 5.331 1.00 5.27 N

ATOM 4665 CA GLU A 296 33.481 8.734 6.733 1.00 5.19 C

ATOM 4666 C GLU A 296 33.323 7.205 6.834 1.00 5.43 C

ATOM 4667 O GLU A 296 32.895 6.577 5.865 1.00 5.79 O

ATOM 4668 CB GLU A 296 32.420 9.491 7.565 1.00 4.61 C

ATOM 4669 CG GLU A 296 30.968 9.162 7.156 1.00 4.85 C

ATOM 4670 CD GLU A 296 29.943 10.076 7.806 1.00 6.54 C

ATOM 4671 OE1 GLU A 296 29.006 9.533 8.426 1.00 6.45 O

ATOM 4672 OE2 GLU A 296 30.095 11.306 7.652 1.00 6.53 O1-

ATOM 4673 H GLU A 296 32.906 8.445 4.739 1.00 0.00 H

ATOM 4674 HA GLU A 296 34.479 8.999 7.079 1.00 0.00 H

ATOM 4675 HB3 GLU A 296 32.602 10.562 7.484 1.00 0.00 H

ATOM 4676 HB2 GLU A 296 32.557 9.263 8.623 1.00 0.00 H

ATOM 4677 HG3 GLU A 296 30.737 8.134 7.420 1.00 0.00 H

ATOM 4678 HG2 GLU A 296 30.831 9.245 6.078 1.00 0.00 H

ATOM 4679 N VAL A 297 33.664 6.620 7.995 1.00 5.18 N

ATOM 4680 CA VAL A 297 33.554 5.172 8.202 1.00 5.67 C

ATOM 4681 C VAL A 297 33.148 4.775 9.628 1.00 5.50 C

ATOM 4682 O VAL A 297 33.512 5.450 10.591 1.00 5.46 O

ATOM 4683 CB VAL A 297 34.874 4.410 7.890 1.00 0.00 C

ATOM 4684 CG1 VAL A 297 35.213 4.373 6.398 1.00 0.00 C

ATOM 4685 CG2 VAL A 297 36.099 4.824 8.730 1.00 0.00 C

ATOM 4686 HB VAL A 297 34.682 3.378 8.155 1.00 0.00 H

ATOM 4687 HG11 VAL A 297 36.039 3.691 6.193 1.00 0.00 H

ATOM 4688 HG12 VAL A 297 34.360 4.051 5.800 1.00 0.00 H

ATOM 4689 HG13 VAL A 297 35.518 5.359 6.064 1.00 0.00 H

ATOM 4690 HG21 VAL A 297 36.995 4.294 8.405 1.00 0.00 H

ATOM 4691 HG22 VAL A 297 36.302 5.890 8.651 1.00 0.00 H

ATOM 4692 HG23 VAL A 297 35.965 4.593 9.787 1.00 0.00 H

ATOM 4693 H VAL A 297 34.010 7.166 8.771 1.00 0.00 H

ATOM 4694 HA VAL A 297 32.763 4.797 7.552 1.00 0.00 H

ATOM 4695 N PHE A 298 32.460 3.623 9.708 1.00 5.09 N

ATOM 4696 CA PHE A 298 32.219 2.861 10.928 1.00 5.25 C

ATOM 4697 C PHE A 298 33.414 1.922 11.168 1.00 5.39 C

ATOM 4698 O PHE A 298 33.753 1.130 10.287 1.00 7.21 O

ATOM 4699 CB PHE A 298 30.932 2.019 10.772 1.00 5.20 C

ATOM 4700 CG PHE A 298 29.655 2.769 10.428 1.00 4.39 C

ATOM 4701 CD1 PHE A 298 29.291 3.940 11.125 1.00 4.12 C

ATOM 4702 CD2 PHE A 298 28.742 2.215 9.506 1.00 4.24 C

ATOM 4703 CE1 PHE A 298 28.081 4.563 10.860 1.00 3.35 C

ATOM 4704 CE2 PHE A 298 27.536 2.854 9.255 1.00 3.89 C

ATOM 4705 CZ PHE A 298 27.210 4.026 9.923 1.00 4.20 C

ATOM 4706 H PHE A 298 32.211 3.149 8.848 1.00 0.00 H

ATOM 4707 HA PHE A 298 32.111 3.542 11.773 1.00 0.00 H

ATOM 4708 HB3 PHE A 298 30.743 1.464 11.691 1.00 0.00 H

ATOM 4709 HB2 PHE A 298 31.092 1.261 10.007 1.00 0.00 H

ATOM 4710 HD1 PHE A 298 29.948 4.359 11.869 1.00 0.00 H

ATOM 4711 HD2 PHE A 298 28.976 1.295 8.991 1.00 0.00 H

ATOM 4712 HE1 PHE A 298 27.816 5.463 11.393 1.00 0.00 H

ATOM 4713 HE2 PHE A 298 26.846 2.434 8.545 1.00 0.00 H

ATOM 4714 HZ PHE A 298 26.269 4.515 9.719 1.00 0.00 H

ATOM 4715 N SER A 299 33.998 2.014 12.370 1.00 5.60 N

ATOM 4716 CA SER A 299 35.060 1.132 12.857 1.00 5.82 C

ATOM 4717 C SER A 299 34.507 0.398 14.084 1.00 5.76 C

ATOM 4718 O SER A 299 33.918 1.048 14.944 1.00 5.59 O

ATOM 4719 CB SER A 299 36.284 1.985 13.234 1.00 0.00 C

ATOM 4720 OG SER A 299 36.852 2.552 12.072 1.00 0.00 O

ATOM 4721 HB2 SER A 299 36.022 2.781 13.931 1.00 0.00 H

ATOM 4722 HB3 SER A 299 37.045 1.371 13.718 1.00 0.00 H

ATOM 4723 HG SER A 299 36.240 3.205 11.724 1.00 0.00 H

ATOM 4724 H SER A 299 33.662 2.693 13.039 1.00 0.00 H

ATOM 4725 HA SER A 299 35.349 0.396 12.104 1.00 0.00 H

ATOM 4726 N PHE A 300 34.658 -0.935 14.131 1.00 5.43 N

ATOM 4727 CA PHE A 300 33.944 -1.782 15.095 1.00 5.44 C

ATOM 4728 C PHE A 300 34.599 -1.938 16.469 1.00 5.61 C

ATOM 4729 O PHE A 300 34.034 -2.655 17.293 1.00 5.89 O

ATOM 4730 CB PHE A 300 33.690 -3.179 14.494 1.00 5.22 C

ATOM 4731 CG PHE A 300 32.710 -3.282 13.344 1.00 5.04 C

ATOM 4732 CD1 PHE A 300 31.959 -2.180 12.871 1.00 5.59 C

ATOM 4733 CD2 PHE A 300 32.516 -4.549 12.759 1.00 6.11 C

ATOM 4734 CE1 PHE A 300 31.068 -2.352 11.824 1.00 5.44 C

ATOM 4735 CE2 PHE A 300 31.619 -4.700 11.716 1.00 6.56 C

ATOM 4736 CZ PHE A 300 30.906 -3.606 11.253 1.00 6.13 C

ATOM 4737 H PHE A 300 35.168 -1.420 13.406 1.00 0.00 H

ATOM 4738 HA PHE A 300 32.975 -1.329 15.304 1.00 0.00 H

ATOM 4739 HB3 PHE A 300 33.308 -3.861 15.255 1.00 0.00 H

ATOM 4740 HB2 PHE A 300 34.636 -3.616 14.174 1.00 0.00 H

ATOM 4741 HD1 PHE A 300 32.065 -1.197 13.297 1.00 0.00 H

ATOM 4742 HD2 PHE A 300 33.072 -5.404 13.116 1.00 0.00 H

ATOM 4743 HE1 PHE A 300 30.499 -1.511 11.456 1.00 0.00 H

ATOM 4744 HE2 PHE A 300 31.475 -5.669 11.261 1.00 0.00 H

ATOM 4745 HZ PHE A 300 30.217 -3.749 10.443 1.00 0.00 H

ATOM 4746 N THR A 301 35.756 -1.305 16.711 1.00 5.99 N

ATOM 4747 CA THR A 301 36.548 -1.526 17.920 1.00 6.27 C

ATOM 4748 C THR A 301 35.767 -1.299 19.237 1.00 6.34 C

ATOM 4749 O THR A 301 35.879 -2.150 20.113 1.00 6.30 O

ATOM 4750 CB THR A 301 37.877 -0.719 17.917 1.00 0.00 C

ATOM 4751 OG1 THR A 301 37.737 0.614 18.346 1.00 0.00 O

ATOM 4752 CG2 THR A 301 38.625 -0.727 16.574 1.00 0.00 C

ATOM 4753 HB THR A 301 38.536 -1.191 18.648 1.00 0.00 H

ATOM 4754 HG1 THR A 301 38.574 1.069 18.224 1.00 0.00 H

ATOM 4755 HG21 THR A 301 39.599 -0.245 16.666 1.00 0.00 H

ATOM 4756 HG22 THR A 301 38.795 -1.747 16.229 1.00 0.00 H

ATOM 4757 HG23 THR A 301 38.070 -0.199 15.797 1.00 0.00 H

ATOM 4758 H THR A 301 36.164 -0.709 16.006 1.00 0.00 H

ATOM 4759 HA THR A 301 36.825 -2.583 17.899 1.00 0.00 H

ATOM 4760 N HIS A 302 34.944 -0.232 19.318 1.00 5.76 N

ATOM 4761 CA HIS A 302 34.119 0.127 20.484 1.00 6.05 C

ATOM 4762 C HIS A 302 32.948 -0.827 20.772 1.00 6.53 C

ATOM 4763 O HIS A 302 32.544 -0.946 21.928 1.00 7.62 O

ATOM 4764 CB HIS A 302 33.623 1.580 20.382 1.00 5.97 C

ATOM 4765 CG HIS A 302 32.712 1.906 19.229 1.00 5.80 C

ATOM 4766 ND1 HIS A 302 33.095 1.743 17.896 1.00 8.34 N

ATOM 4767 CD2 HIS A 302 31.435 2.424 19.264 1.00 2.47 C

ATOM 4768 CE1 HIS A 302 32.053 2.158 17.193 1.00 2.27 C

ATOM 4769 NE2 HIS A 302 31.037 2.568 17.949 1.00 6.63 N

ATOM 4770 H HIS A 302 34.891 0.406 18.535 1.00 0.00 H

ATOM 4771 HA HIS A 302 34.764 0.098 21.356 1.00 0.00 H

ATOM 4772 HB3 HIS A 302 34.474 2.252 20.324 1.00 0.00 H

ATOM 4773 HB2 HIS A 302 33.101 1.844 21.302 1.00 0.00 H

ATOM 4774 HD2 HIS A 302 30.802 2.705 20.090 1.00 0.00 H

ATOM 4775 HE1 HIS A 302 32.043 2.194 16.117 1.00 0.00 H

ATOM 4776 HE2 HIS A 302 30.157 2.948 17.627 1.00 0.00 H

ATOM 4777 N VAL A 303 32.440 -1.494 19.728 1.00 6.26 N

ATOM 4778 CA VAL A 303 31.373 -2.486 19.828 1.00 6.54 C

ATOM 4779 C VAL A 303 31.905 -3.940 19.874 1.00 6.43 C

ATOM 4780 O VAL A 303 31.101 -4.863 20.019 1.00 6.63 O

ATOM 4781 CB VAL A 303 30.349 -2.314 18.677 1.00 0.00 C

ATOM 4782 CG1 VAL A 303 29.574 -0.990 18.798 1.00 0.00 C

ATOM 4783 CG2 VAL A 303 30.923 -2.472 17.260 1.00 0.00 C

ATOM 4784 HB VAL A 303 29.618 -3.102 18.811 1.00 0.00 H

ATOM 4785 HG11 VAL A 303 28.814 -0.903 18.022 1.00 0.00 H

ATOM 4786 HG12 VAL A 303 29.068 -0.912 19.761 1.00 0.00 H

ATOM 4787 HG13 VAL A 303 30.237 -0.131 18.701 1.00 0.00 H

ATOM 4788 HG21 VAL A 303 30.139 -2.395 16.507 1.00 0.00 H

ATOM 4789 HG22 VAL A 303 31.632 -1.674 17.052 1.00 0.00 H

ATOM 4790 HG23 VAL A 303 31.420 -3.431 17.117 1.00 0.00 H

ATOM 4791 H VAL A 303 32.833 -1.353 18.807 1.00 0.00 H

ATOM 4792 HA VAL A 303 30.827 -2.342 20.762 1.00 0.00 H

ATOM 4793 N GLU A 304 33.239 -4.116 19.815 1.00 6.84 N

ATOM 4794 CA GLU A 304 33.943 -5.386 20.006 1.00 7.10 C

ATOM 4795 C GLU A 304 34.413 -5.626 21.451 1.00 7.44 C

ATOM 4796 O GLU A 304 34.820 -6.753 21.730 1.00 7.41 O

ATOM 4797 CB GLU A 304 35.146 -5.483 19.040 1.00 0.00 C

ATOM 4798 CG GLU A 304 34.770 -5.931 17.619 1.00 0.00 C

ATOM 4799 CD GLU A 304 36.011 -6.022 16.733 1.00 0.00 C

ATOM 4800 OE1 GLU A 304 36.647 -7.099 16.757 1.00 0.00 O

ATOM 4801 OE2 GLU A 304 36.310 -5.010 16.063 1.00 0.00 O1-

ATOM 4802 HB2 GLU A 304 35.650 -4.518 18.987 1.00 0.00 H

ATOM 4803 HB3 GLU A 304 35.896 -6.176 19.425 1.00 0.00 H

ATOM 4804 HG2 GLU A 304 34.290 -6.909 17.650 1.00 0.00 H

ATOM 4805 HG3 GLU A 304 34.049 -5.247 17.172 1.00 0.00 H

ATOM 4806 H GLU A 304 33.832 -3.310 19.670 1.00 0.00 H

ATOM 4807 HA GLU A 304 33.269 -6.213 19.782 1.00 0.00 H

ATOM 4808 N LEU A 305 34.367 -4.624 22.354 1.00 7.41 N

ATOM 4809 CA LEU A 305 34.799 -4.810 23.748 1.00 7.59 C

ATOM 4810 C LEU A 305 33.906 -5.799 24.493 1.00 7.98 C

ATOM 4811 O LEU A 305 32.686 -5.769 24.346 1.00 7.44 O

ATOM 4812 CB LEU A 305 34.855 -3.495 24.556 1.00 0.00 C

ATOM 4813 CG LEU A 305 36.033 -2.556 24.253 1.00 0.00 C

ATOM 4814 CD1 LEU A 305 37.383 -3.276 24.097 1.00 0.00 C

ATOM 4815 CD2 LEU A 305 35.743 -1.690 23.039 1.00 0.00 C

ATOM 4816 HB2 LEU A 305 33.908 -2.957 24.489 1.00 0.00 H

ATOM 4817 HB3 LEU A 305 34.958 -3.756 25.609 1.00 0.00 H

ATOM 4818 HG LEU A 305 36.126 -1.884 25.107 1.00 0.00 H

ATOM 4819 HD11 LEU A 305 38.205 -2.561 24.102 1.00 0.00 H

ATOM 4820 HD12 LEU A 305 37.548 -4.001 24.895 1.00 0.00 H

ATOM 4821 HD13 LEU A 305 37.447 -3.802 23.144 1.00 0.00 H

ATOM 4822 HD21 LEU A 305 35.427 -0.696 23.331 1.00 0.00 H

ATOM 4823 HD22 LEU A 305 36.623 -1.593 22.409 1.00 0.00 H

ATOM 4824 HD23 LEU A 305 34.936 -2.098 22.439 1.00 0.00 H

ATOM 4825 H LEU A 305 34.041 -3.707 22.087 1.00 0.00 H

ATOM 4826 HA LEU A 305 35.792 -5.258 23.717 1.00 0.00 H

ATOM 4827 N GLY A 306 34.560 -6.660 25.278 1.00 7.99 N

ATOM 4828 CA GLY A 306 33.910 -7.740 25.999 1.00 8.35 C

ATOM 4829 C GLY A 306 33.618 -8.947 25.096 1.00 8.41 C

ATOM 4830 O GLY A 306 32.898 -9.840 25.532 1.00 8.65 O

ATOM 4831 H GLY A 306 35.566 -6.605 25.348 1.00 0.00 H

ATOM 4832 HA3 GLY A 306 32.989 -7.402 26.475 1.00 0.00 H

ATOM 4833 HA2 GLY A 306 34.597 -8.032 26.786 1.00 0.00 H

ATOM 4834 N THR A 307 34.159 -8.981 23.866 1.00 8.43 N

ATOM 4835 CA THR A 307 34.055 -10.097 22.930 1.00 8.85 C

ATOM 4836 C THR A 307 35.467 -10.505 22.482 1.00 9.35 C

ATOM 4837 O THR A 307 36.331 -9.646 22.291 1.00 9.53 O

ATOM 4838 CB THR A 307 33.180 -9.748 21.687 1.00 8.87 C

ATOM 4839 OG1 THR A 307 33.841 -9.118 20.606 1.00 9.08 O

ATOM 4840 CG2 THR A 307 31.880 -8.999 22.014 1.00 9.66 C

ATOM 4841 H THR A 307 34.732 -8.209 23.553 1.00 0.00 H

ATOM 4842 HA THR A 307 33.604 -10.958 23.426 1.00 0.00 H

ATOM 4843 HB THR A 307 32.886 -10.702 21.265 1.00 0.00 H

ATOM 4844 HG1 THR A 307 34.255 -8.312 20.933 1.00 0.00 H

ATOM 4845 HG21 THR A 307 31.297 -8.837 21.110 1.00 0.00 H

ATOM 4846 HG22 THR A 307 31.265 -9.564 22.715 1.00 0.00 H

ATOM 4847 HG23 THR A 307 32.075 -8.019 22.448 1.00 0.00 H

ATOM 4848 N SER A 308 35.670 -11.822 22.342 1.00 9.97 N

ATOM 4849 CA SER A 308 36.909 -12.436 21.875 1.00 11.66 C

ATOM 4850 C SER A 308 37.163 -12.095 20.387 1.00 12.10 C

ATOM 4851 O SER A 308 36.224 -12.180 19.594 1.00 12.70 O

ATOM 4852 CB SER A 308 36.772 -13.955 22.116 1.00 11.30 C

ATOM 4853 OG SER A 308 37.783 -14.713 21.489 1.00 15.44 O

ATOM 4854 H SER A 308 34.912 -12.463 22.541 1.00 0.00 H

ATOM 4855 HA SER A 308 37.705 -12.056 22.515 1.00 0.00 H

ATOM 4856 HB3 SER A 308 35.815 -14.319 21.761 1.00 0.00 H

ATOM 4857 HB2 SER A 308 36.797 -14.170 23.184 1.00 0.00 H

ATOM 4858 HG SER A 308 37.646 -15.639 21.708 1.00 0.00 H

ATOM 4859 N PRO A 309 38.414 -11.725 20.019 1.00 13.35 N

ATOM 4860 CA PRO A 309 38.770 -11.469 18.610 1.00 13.85 C

ATOM 4861 C PRO A 309 38.733 -12.721 17.705 1.00 13.90 C

ATOM 4862 O PRO A 309 38.572 -12.573 16.494 1.00 14.46 O

ATOM 4863 CB PRO A 309 40.177 -10.858 18.700 1.00 13.89 C

ATOM 4864 CG PRO A 309 40.777 -11.466 19.958 1.00 15.62 C

ATOM 4865 CD PRO A 309 39.580 -11.579 20.896 1.00 13.96 C

ATOM 4866 HA PRO A 309 38.087 -10.732 18.182 1.00 0.00 H

ATOM 4867 HB3 PRO A 309 40.092 -9.777 18.822 1.00 0.00 H

ATOM 4868 HB2 PRO A 309 40.794 -11.036 17.817 1.00 0.00 H

ATOM 4869 HG3 PRO A 309 41.601 -10.885 20.374 1.00 0.00 H

ATOM 4870 HG2 PRO A 309 41.155 -12.464 19.729 1.00 0.00 H

ATOM 4871 HD2 PRO A 309 39.709 -12.420 21.579 1.00 0.00 H

ATOM 4872 HD3 PRO A 309 39.463 -10.667 21.483 1.00 0.00 H

ATOM 4873 N PHE A 310 38.852 -13.920 18.307 1.00 13.98 N

ATOM 4874 CA PHE A 310 38.789 -15.216 17.625 1.00 14.62 C

ATOM 4875 C PHE A 310 37.351 -15.600 17.232 1.00 14.47 C

ATOM 4876 O PHE A 310 37.164 -16.241 16.197 1.00 14.78 O

ATOM 4877 CB PHE A 310 39.380 -16.312 18.539 1.00 0.00 C

ATOM 4878 CG PHE A 310 40.773 -16.029 19.076 1.00 0.00 C

ATOM 4879 CD1 PHE A 310 40.941 -15.508 20.377 1.00 0.00 C

ATOM 4880 CD2 PHE A 310 41.901 -16.148 18.237 1.00 0.00 C

ATOM 4881 CE1 PHE A 310 42.204 -15.163 20.836 1.00 0.00 C

ATOM 4882 CE2 PHE A 310 43.158 -15.801 18.716 1.00 0.00 C

ATOM 4883 CZ PHE A 310 43.308 -15.313 20.009 1.00 0.00 C

ATOM 4884 HB2 PHE A 310 38.717 -16.495 19.387 1.00 0.00 H

ATOM 4885 HB3 PHE A 310 39.418 -17.253 17.987 1.00 0.00 H

ATOM 4886 HD1 PHE A 310 40.097 -15.381 21.031 1.00 0.00 H

ATOM 4887 HD2 PHE A 310 41.792 -16.512 17.226 1.00 0.00 H

ATOM 4888 HE1 PHE A 310 42.326 -14.774 21.836 1.00 0.00 H

ATOM 4889 HE2 PHE A 310 44.022 -15.906 18.076 1.00 0.00 H

ATOM 4890 HZ PHE A 310 44.290 -15.042 20.369 1.00 0.00 H

ATOM 4891 H PHE A 310 38.984 -13.957 19.308 1.00 0.00 H

ATOM 4892 HA PHE A 310 39.391 -15.158 16.716 1.00 0.00 H

ATOM 4893 N PHE A 311 36.368 -15.202 18.056 1.00 13.70 N

ATOM 4894 CA PHE A 311 34.954 -15.491 17.855 1.00 13.04 C

ATOM 4895 C PHE A 311 34.125 -14.519 18.693 1.00 12.78 C

ATOM 4896 O PHE A 311 34.273 -14.504 19.914 1.00 13.00 O

ATOM 4897 CB PHE A 311 34.645 -16.968 18.203 1.00 13.10 C

ATOM 4898 CG PHE A 311 33.187 -17.395 18.105 1.00 12.24 C

ATOM 4899 CD1 PHE A 311 32.670 -18.352 19.004 1.00 12.54 C

ATOM 4900 CD2 PHE A 311 32.385 -16.984 17.017 1.00 11.75 C

ATOM 4901 CE1 PHE A 311 31.374 -18.826 18.852 1.00 12.26 C

ATOM 4902 CE2 PHE A 311 31.091 -17.465 16.885 1.00 11.20 C

ATOM 4903 CZ PHE A 311 30.590 -18.385 17.794 1.00 12.58 C

ATOM 4904 H PHE A 311 36.593 -14.657 18.877 1.00 0.00 H

ATOM 4905 HA PHE A 311 34.728 -15.315 16.802 1.00 0.00 H

ATOM 4906 HB3 PHE A 311 34.994 -17.171 19.217 1.00 0.00 H

ATOM 4907 HB2 PHE A 311 35.217 -17.632 17.554 1.00 0.00 H

ATOM 4908 HD1 PHE A 311 33.284 -18.726 19.809 1.00 0.00 H

ATOM 4909 HD2 PHE A 311 32.764 -16.295 16.278 1.00 0.00 H

ATOM 4910 HE1 PHE A 311 30.980 -19.550 19.550 1.00 0.00 H

ATOM 4911 HE2 PHE A 311 30.479 -17.127 16.063 1.00 0.00 H

ATOM 4912 HZ PHE A 311 29.584 -18.758 17.671 1.00 0.00 H

ATOM 4913 N ARG A 312 33.257 -13.745 18.024 1.00 12.35 N

ATOM 4914 CA ARG A 312 32.414 -12.718 18.635 1.00 11.84 C

ATOM 4915 C ARG A 312 31.533 -13.250 19.786 1.00 11.51 C

ATOM 4916 O ARG A 312 31.514 -12.632 20.846 1.00 10.98 O

ATOM 4917 CB ARG A 312 31.658 -11.977 17.508 1.00 13.22 C

ATOM 4918 CG ARG A 312 30.711 -10.857 17.968 1.00 13.59 C

ATOM 4919 CD ARG A 312 30.317 -9.893 16.839 1.00 14.98 C

ATOM 4920 NE ARG A 312 29.544 -8.744 17.333 1.00 12.00 N

ATOM 4921 CZ ARG A 312 30.032 -7.700 18.026 1.00 12.44 C

ATOM 4922 NH1 ARG A 312 31.325 -7.615 18.366 1.00 13.27 N

ATOM 4923 NH2 ARG A 312 29.213 -6.709 18.386 1.00 11.50 N1+

ATOM 4924 H ARG A 312 33.194 -13.826 17.020 1.00 0.00 H

ATOM 4925 HA ARG A 312 33.102 -11.997 19.080 1.00 0.00 H

ATOM 4926 HB3 ARG A 312 31.101 -12.682 16.888 1.00 0.00 H

ATOM 4927 HB2 ARG A 312 32.415 -11.538 16.856 1.00 0.00 H

ATOM 4928 HG3 ARG A 312 31.120 -10.301 18.811 1.00 0.00 H

ATOM 4929 HG2 ARG A 312 29.806 -11.350 18.314 1.00 0.00 H

ATOM 4930 HD3 ARG A 312 29.611 -10.421 16.197 1.00 0.00 H

ATOM 4931 HD2 ARG A 312 31.153 -9.606 16.202 1.00 0.00 H

ATOM 4932 HE ARG A 312 28.553 -8.770 17.139 1.00 0.00 H

ATOM 4933 HH12 ARG A 312 31.663 -6.814 18.882 1.00 0.00 H

ATOM 4934 HH11 ARG A 312 31.970 -8.347 18.106 1.00 0.00 H

ATOM 4935 HH22 ARG A 312 29.567 -5.924 18.917 1.00 0.00 H

ATOM 4936 HH21 ARG A 312 28.230 -6.735 18.152 1.00 0.00 H

ATOM 4937 N TYR A 313 30.902 -14.424 19.615 1.00 10.11 N

ATOM 4938 CA TYR A 313 30.027 -15.038 20.624 1.00 10.25 C

ATOM 4939 C TYR A 313 30.751 -15.873 21.701 1.00 9.78 C

ATOM 4940 O TYR A 313 30.150 -16.788 22.259 1.00 9.68 O

ATOM 4941 CB TYR A 313 28.883 -15.788 19.919 1.00 10.21 C

ATOM 4942 CG TYR A 313 27.986 -14.858 19.131 1.00 10.73 C

ATOM 4943 CD1 TYR A 313 28.156 -14.706 17.740 1.00 10.07 C

ATOM 4944 CD2 TYR A 313 27.013 -14.096 19.808 1.00 11.70 C

ATOM 4945 CE1 TYR A 313 27.365 -13.782 17.034 1.00 11.00 C

ATOM 4946 CE2 TYR A 313 26.222 -13.174 19.100 1.00 12.34 C

ATOM 4947 CZ TYR A 313 26.398 -13.014 17.713 1.00 12.23 C

ATOM 4948 OH TYR A 313 25.633 -12.117 17.026 1.00 13.07 O

ATOM 4949 H TYR A 313 30.998 -14.921 18.742 1.00 0.00 H

ATOM 4950 HA TYR A 313 29.553 -14.234 21.190 1.00 0.00 H

ATOM 4951 HB3 TYR A 313 28.252 -16.305 20.645 1.00 0.00 H

ATOM 4952 HB2 TYR A 313 29.287 -16.552 19.259 1.00 0.00 H

ATOM 4953 HD1 TYR A 313 28.904 -15.281 17.215 1.00 0.00 H

ATOM 4954 HD2 TYR A 313 26.886 -14.201 20.875 1.00 0.00 H

ATOM 4955 HE1 TYR A 313 27.510 -13.664 15.970 1.00 0.00 H

ATOM 4956 HE2 TYR A 313 25.488 -12.587 19.629 1.00 0.00 H

ATOM 4957 HH TYR A 313 25.745 -12.191 16.067 1.00 0.00 H

ATOM 4958 N ASN A 314 31.996 -15.501 22.036 1.00 9.90 N

ATOM 4959 CA ASN A 314 32.670 -15.866 23.283 1.00 10.31 C

ATOM 4960 C ASN A 314 32.992 -14.548 23.992 1.00 9.72 C

ATOM 4961 O ASN A 314 33.737 -13.730 23.454 1.00 9.86 O

ATOM 4962 CB ASN A 314 33.955 -16.678 22.990 1.00 10.51 C

ATOM 4963 CG ASN A 314 33.768 -18.154 22.606 1.00 12.46 C

ATOM 4964 OD1 ASN A 314 34.755 -18.830 22.332 1.00 14.32 O

ATOM 4965 ND2 ASN A 314 32.540 -18.681 22.586 1.00 13.58 N

ATOM 4966 H ASN A 314 32.434 -14.750 21.518 1.00 0.00 H

ATOM 4967 HA ASN A 314 31.996 -16.457 23.907 1.00 0.00 H

ATOM 4968 HB3 ASN A 314 34.575 -16.690 23.888 1.00 0.00 H

ATOM 4969 HB2 ASN A 314 34.550 -16.190 22.219 1.00 0.00 H

ATOM 4970 HD22 ASN A 314 32.417 -19.656 22.355 1.00 0.00 H

ATOM 4971 HD21 ASN A 314 31.728 -18.113 22.786 1.00 0.00 H

ATOM 4972 N ILE A 315 32.404 -14.369 25.185 1.00 9.48 N

ATOM 4973 CA ILE A 315 32.575 -13.177 26.015 1.00 9.76 C

ATOM 4974 C ILE A 315 33.908 -13.238 26.781 1.00 10.68 C

ATOM 4975 O ILE A 315 34.286 -14.305 27.265 1.00 10.94 O

ATOM 4976 CB ILE A 315 31.405 -13.032 27.039 1.00 0.00 C

ATOM 4977 CG1 ILE A 315 30.030 -12.961 26.336 1.00 0.00 C

ATOM 4978 CG2 ILE A 315 31.548 -11.870 28.051 1.00 0.00 C

ATOM 4979 CD1 ILE A 315 29.863 -11.824 25.311 1.00 0.00 C

ATOM 4980 HB ILE A 315 31.386 -13.946 27.635 1.00 0.00 H

ATOM 4981 HG12 ILE A 315 29.865 -13.909 25.825 1.00 0.00 H

ATOM 4982 HG13 ILE A 315 29.235 -12.898 27.079 1.00 0.00 H

ATOM 4983 HG21 ILE A 315 30.670 -11.805 28.693 1.00 0.00 H

ATOM 4984 HG22 ILE A 315 32.404 -12.001 28.713 1.00 0.00 H

ATOM 4985 HG23 ILE A 315 31.659 -10.908 27.555 1.00 0.00 H

ATOM 4986 HD11 ILE A 315 28.809 -11.654 25.092 1.00 0.00 H

ATOM 4987 HD12 ILE A 315 30.275 -10.882 25.671 1.00 0.00 H

ATOM 4988 HD13 ILE A 315 30.356 -12.066 24.369 1.00 0.00 H

ATOM 4989 H ILE A 315 31.809 -15.091 25.563 1.00 0.00 H

ATOM 4990 HA ILE A 315 32.586 -12.309 25.358 1.00 0.00 H

ATOM 4991 N VAL A 316 34.568 -12.076 26.892 1.00 10.55 N

ATOM 4992 CA VAL A 316 35.765 -11.863 27.707 1.00 11.11 C

ATOM 4993 C VAL A 316 35.541 -10.635 28.626 1.00 11.50 C

ATOM 4994 O VAL A 316 34.639 -9.844 28.352 1.00 11.01 O

ATOM 4995 CB VAL A 316 37.026 -11.632 26.819 1.00 11.55 C

ATOM 4996 CG1 VAL A 316 37.344 -12.870 25.962 1.00 12.98 C

ATOM 4997 CG2 VAL A 316 36.984 -10.365 25.942 1.00 11.75 C

ATOM 4998 H VAL A 316 34.170 -11.244 26.477 1.00 0.00 H

ATOM 4999 HA VAL A 316 35.924 -12.744 28.325 1.00 0.00 H

ATOM 5000 HB VAL A 316 37.880 -11.511 27.484 1.00 0.00 H

ATOM 5001 HG11 VAL A 316 38.274 -12.739 25.408 1.00 0.00 H

ATOM 5002 HG12 VAL A 316 37.452 -13.761 26.581 1.00 0.00 H

ATOM 5003 HG13 VAL A 316 36.554 -13.062 25.236 1.00 0.00 H

ATOM 5004 HG21 VAL A 316 37.880 -10.284 25.326 1.00 0.00 H

ATOM 5005 HG22 VAL A 316 36.130 -10.390 25.271 1.00 0.00 H

ATOM 5006 HG23 VAL A 316 36.919 -9.452 26.533 1.00 0.00 H

ATOM 5007 N PRO A 317 36.355 -10.465 29.695 1.00 11.70 N

ATOM 5008 CA PRO A 317 36.347 -9.225 30.500 1.00 11.76 C

ATOM 5009 C PRO A 317 36.876 -7.993 29.740 1.00 11.33 C

ATOM 5010 O PRO A 317 37.751 -8.128 28.883 1.00 11.39 O

ATOM 5011 CB PRO A 317 37.255 -9.557 31.701 1.00 12.39 C

ATOM 5012 CG PRO A 317 37.323 -11.073 31.747 1.00 13.07 C

ATOM 5013 CD PRO A 317 37.258 -11.457 30.277 1.00 12.33 C

ATOM 5014 HA PRO A 317 35.328 -9.049 30.849 1.00 0.00 H

ATOM 5015 HB3 PRO A 317 36.883 -9.135 32.636 1.00 0.00 H

ATOM 5016 HB2 PRO A 317 38.264 -9.167 31.550 1.00 0.00 H

ATOM 5017 HG3 PRO A 317 36.444 -11.461 32.263 1.00 0.00 H

ATOM 5018 HG2 PRO A 317 38.209 -11.453 32.256 1.00 0.00 H

ATOM 5019 HD2 PRO A 317 38.240 -11.363 29.811 1.00 0.00 H

ATOM 5020 HD3 PRO A 317 36.926 -12.490 30.180 1.00 0.00 H

ATOM 5021 N PHE A 318 36.363 -6.813 30.115 1.00 10.75 N

ATOM 5022 CA PHE A 318 36.886 -5.512 29.695 1.00 10.11 C

ATOM 5023 C PHE A 318 36.617 -4.502 30.821 1.00 9.91 C

ATOM 5024 O PHE A 318 35.641 -4.649 31.560 1.00 10.31 O

ATOM 5025 CB PHE A 318 36.272 -5.096 28.332 1.00 10.32 C

ATOM 5026 CG PHE A 318 34.851 -4.545 28.332 1.00 10.10 C

ATOM 5027 CD1 PHE A 318 34.620 -3.152 28.285 1.00 10.58 C

ATOM 5028 CD2 PHE A 318 33.751 -5.414 28.483 1.00 9.13 C

ATOM 5029 CE1 PHE A 318 33.320 -2.662 28.314 1.00 10.91 C

ATOM 5030 CE2 PHE A 318 32.459 -4.905 28.504 1.00 9.67 C

ATOM 5031 CZ PHE A 318 32.245 -3.535 28.413 1.00 11.03 C

ATOM 5032 H PHE A 318 35.642 -6.778 30.822 1.00 0.00 H

ATOM 5033 HA PHE A 318 37.968 -5.600 29.577 1.00 0.00 H

ATOM 5034 HB3 PHE A 318 36.302 -5.943 27.645 1.00 0.00 H

ATOM 5035 HB2 PHE A 318 36.918 -4.345 27.875 1.00 0.00 H

ATOM 5036 HD1 PHE A 318 35.448 -2.464 28.204 1.00 0.00 H

ATOM 5037 HD2 PHE A 318 33.909 -6.478 28.572 1.00 0.00 H

ATOM 5038 HE1 PHE A 318 33.146 -1.597 28.258 1.00 0.00 H

ATOM 5039 HE2 PHE A 318 31.618 -5.576 28.600 1.00 0.00 H

ATOM 5040 HZ PHE A 318 31.237 -3.146 28.432 1.00 0.00 H

ATOM 5041 N THR A 319 37.484 -3.482 30.908 1.00 9.07 N

ATOM 5042 CA THR A 319 37.326 -2.335 31.805 1.00 9.45 C

ATOM 5043 C THR A 319 37.022 -1.069 30.973 1.00 8.78 C

ATOM 5044 O THR A 319 37.096 -1.118 29.744 1.00 8.53 O

ATOM 5045 CB THR A 319 38.598 -2.139 32.683 1.00 0.00 C

ATOM 5046 OG1 THR A 319 38.370 -1.183 33.702 1.00 0.00 O

ATOM 5047 CG2 THR A 319 39.870 -1.703 31.931 1.00 0.00 C

ATOM 5048 HB THR A 319 38.811 -3.082 33.189 1.00 0.00 H

ATOM 5049 HG1 THR A 319 39.188 -1.049 34.188 1.00 0.00 H

ATOM 5050 HG21 THR A 319 40.707 -1.579 32.618 1.00 0.00 H

ATOM 5051 HG22 THR A 319 40.167 -2.459 31.205 1.00 0.00 H

ATOM 5052 HG23 THR A 319 39.740 -0.760 31.402 1.00 0.00 H

ATOM 5053 H THR A 319 38.253 -3.427 30.258 1.00 0.00 H

ATOM 5054 HA THR A 319 36.475 -2.486 32.473 1.00 0.00 H

ATOM 5055 N LEU A 320 36.661 0.037 31.645 1.00 7.70 N

ATOM 5056 CA LEU A 320 36.235 1.287 31.011 1.00 7.68 C

ATOM 5057 C LEU A 320 37.273 1.904 30.053 1.00 7.37 C

ATOM 5058 O LEU A 320 36.860 2.398 29.007 1.00 7.15 O

ATOM 5059 CB LEU A 320 35.780 2.287 32.093 1.00 7.51 C

ATOM 5060 CG LEU A 320 35.025 3.530 31.568 1.00 7.51 C

ATOM 5061 CD1 LEU A 320 33.656 3.167 30.972 1.00 7.98 C

ATOM 5062 CD2 LEU A 320 34.861 4.563 32.685 1.00 8.58 C

ATOM 5063 H LEU A 320 36.641 0.015 32.655 1.00 0.00 H

ATOM 5064 HA LEU A 320 35.367 1.019 30.409 1.00 0.00 H

ATOM 5065 HB3 LEU A 320 36.654 2.607 32.662 1.00 0.00 H

ATOM 5066 HB2 LEU A 320 35.136 1.772 32.806 1.00 0.00 H

ATOM 5067 HG LEU A 320 35.608 4.009 30.784 1.00 0.00 H

ATOM 5068 HD11 LEU A 320 33.087 4.061 30.711 1.00 0.00 H

ATOM 5069 HD12 LEU A 320 33.764 2.576 30.065 1.00 0.00 H

ATOM 5070 HD13 LEU A 320 33.058 2.586 31.674 1.00 0.00 H

ATOM 5071 HD21 LEU A 320 34.297 5.433 32.352 1.00 0.00 H

ATOM 5072 HD22 LEU A 320 34.327 4.128 33.528 1.00 0.00 H

ATOM 5073 HD23 LEU A 320 35.826 4.916 33.049 1.00 0.00 H

ATOM 5074 N LYS A 321 38.580 1.815 30.381 1.00 6.98 N

ATOM 5075 CA LYS A 321 39.695 2.251 29.524 1.00 7.17 C

ATOM 5076 C LYS A 321 39.692 1.612 28.123 1.00 7.27 C

ATOM 5077 O LYS A 321 39.987 2.308 27.152 1.00 7.00 O

ATOM 5078 CB LYS A 321 41.053 2.021 30.232 1.00 7.32 C

ATOM 5079 CG LYS A 321 42.284 2.306 29.339 1.00 10.27 C

ATOM 5080 CD LYS A 321 43.626 2.191 30.060 1.00 10.51 C

ATOM 5081 CE LYS A 321 44.813 2.448 29.123 1.00 12.35 C

ATOM 5082 NZ LYS A 321 46.076 2.499 29.877 1.00 11.96 N1+

ATOM 5083 H LYS A 321 38.836 1.387 31.259 1.00 0.00 H

ATOM 5084 HA LYS A 321 39.577 3.328 29.390 1.00 0.00 H

ATOM 5085 HB3 LYS A 321 41.114 0.988 30.576 1.00 0.00 H

ATOM 5086 HB2 LYS A 321 41.097 2.640 31.129 1.00 0.00 H

ATOM 5087 HG3 LYS A 321 42.191 3.302 28.908 1.00 0.00 H

ATOM 5088 HG2 LYS A 321 42.316 1.611 28.500 1.00 0.00 H

ATOM 5089 HD3 LYS A 321 43.718 1.207 30.522 1.00 0.00 H

ATOM 5090 HD2 LYS A 321 43.657 2.918 30.863 1.00 0.00 H

ATOM 5091 HE3 LYS A 321 44.687 3.399 28.606 1.00 0.00 H

ATOM 5092 HE2 LYS A 321 44.876 1.673 28.359 1.00 0.00 H

ATOM 5093 HZ1 LYS A 321 46.007 3.223 30.587 1.00 0.00 H

ATOM 5094 HZ2 LYS A 321 46.242 1.611 30.328 1.00 0.00 H

ATOM 5095 HZ3 LYS A 321 46.840 2.713 29.252 1.00 0.00 H

ATOM 5096 N GLN A 322 39.357 0.311 28.051 1.00 7.34 N

ATOM 5097 CA GLN A 322 39.248 -0.444 26.802 1.00 7.26 C

ATOM 5098 C GLN A 322 38.186 0.155 25.864 1.00 6.90 C

ATOM 5099 O GLN A 322 38.432 0.241 24.664 1.00 7.60 O

ATOM 5100 CB GLN A 322 38.918 -1.925 27.105 1.00 0.00 C

ATOM 5101 CG GLN A 322 39.936 -2.683 27.980 1.00 0.00 C

ATOM 5102 CD GLN A 322 41.338 -2.798 27.373 1.00 0.00 C

ATOM 5103 OE1 GLN A 322 42.328 -2.600 28.071 1.00 0.00 O

ATOM 5104 NE2 GLN A 322 41.438 -3.131 26.083 1.00 0.00 N

ATOM 5105 HB2 GLN A 322 37.935 -1.999 27.566 1.00 0.00 H

ATOM 5106 HB3 GLN A 322 38.822 -2.465 26.171 1.00 0.00 H

ATOM 5107 HG2 GLN A 322 40.011 -2.199 28.952 1.00 0.00 H

ATOM 5108 HG3 GLN A 322 39.568 -3.692 28.171 1.00 0.00 H

ATOM 5109 HE22 GLN A 322 42.350 -3.216 25.657 1.00 0.00 H

ATOM 5110 HE21 GLN A 322 40.613 -3.295 25.526 1.00 0.00 H

ATOM 5111 H GLN A 322 39.111 -0.186 28.895 1.00 0.00 H

ATOM 5112 HA GLN A 322 40.210 -0.386 26.291 1.00 0.00 H

ATOM 5113 N TRP A 323 37.054 0.588 26.445 1.00 6.44 N

ATOM 5114 CA TRP A 323 35.948 1.224 25.737 1.00 6.38 C

ATOM 5115 C TRP A 323 36.189 2.704 25.395 1.00 6.18 C

ATOM 5116 O TRP A 323 35.724 3.137 24.342 1.00 6.80 O

ATOM 5117 CB TRP A 323 34.648 0.990 26.521 1.00 6.00 C

ATOM 5118 CG TRP A 323 33.391 1.476 25.866 1.00 6.60 C

ATOM 5119 CD1 TRP A 323 32.884 1.001 24.707 1.00 6.66 C

ATOM 5120 CD2 TRP A 323 32.480 2.529 26.300 1.00 5.64 C

ATOM 5121 NE1 TRP A 323 31.712 1.663 24.410 1.00 6.95 N

ATOM 5122 CE2 TRP A 323 31.397 2.600 25.372 1.00 6.34 C

ATOM 5123 CE3 TRP A 323 32.451 3.427 27.391 1.00 7.33 C

ATOM 5124 CZ2 TRP A 323 30.321 3.489 25.543 1.00 7.17 C

ATOM 5125 CZ3 TRP A 323 31.379 4.322 27.572 1.00 7.32 C

ATOM 5126 CH2 TRP A 323 30.313 4.348 26.654 1.00 7.64 C

ATOM 5127 H TRP A 323 36.947 0.499 27.446 1.00 0.00 H

ATOM 5128 HA TRP A 323 35.842 0.718 24.785 1.00 0.00 H

ATOM 5129 HB3 TRP A 323 34.721 1.443 27.511 1.00 0.00 H

ATOM 5130 HB2 TRP A 323 34.521 -0.081 26.691 1.00 0.00 H

ATOM 5131 HD1 TRP A 323 33.333 0.212 24.124 1.00 0.00 H

ATOM 5132 HE1 TRP A 323 31.157 1.442 23.595 1.00 0.00 H

ATOM 5133 HE3 TRP A 323 33.265 3.426 28.097 1.00 0.00 H

ATOM 5134 HZ2 TRP A 323 29.510 3.519 24.831 1.00 0.00 H

ATOM 5135 HZ3 TRP A 323 31.375 4.990 28.420 1.00 0.00 H

ATOM 5136 HH2 TRP A 323 29.495 5.036 26.800 1.00 0.00 H

ATOM 5137 N LYS A 324 36.936 3.433 26.245 1.00 5.77 N

ATOM 5138 CA LYS A 324 37.389 4.806 25.997 1.00 6.68 C

ATOM 5139 C LYS A 324 38.256 4.901 24.736 1.00 6.25 C

ATOM 5140 O LYS A 324 37.885 5.608 23.801 1.00 6.44 O

ATOM 5141 CB LYS A 324 38.203 5.338 27.194 1.00 6.34 C

ATOM 5142 CG LYS A 324 37.399 5.709 28.441 1.00 7.55 C

ATOM 5143 CD LYS A 324 38.322 6.257 29.540 1.00 7.28 C

ATOM 5144 CE LYS A 324 37.902 5.839 30.948 1.00 7.99 C

ATOM 5145 NZ LYS A 324 38.854 6.347 31.943 1.00 6.69 N1+

ATOM 5146 H LYS A 324 37.261 3.007 27.102 1.00 0.00 H

ATOM 5147 HA LYS A 324 36.507 5.433 25.858 1.00 0.00 H

ATOM 5148 HB3 LYS A 324 38.734 6.238 26.890 1.00 0.00 H

ATOM 5149 HB2 LYS A 324 38.982 4.626 27.461 1.00 0.00 H

ATOM 5150 HG3 LYS A 324 36.833 4.853 28.797 1.00 0.00 H

ATOM 5151 HG2 LYS A 324 36.662 6.465 28.182 1.00 0.00 H

ATOM 5152 HD3 LYS A 324 38.336 7.346 29.477 1.00 0.00 H

ATOM 5153 HD2 LYS A 324 39.351 5.938 29.368 1.00 0.00 H

ATOM 5154 HE3 LYS A 324 37.888 4.752 31.026 1.00 0.00 H

ATOM 5155 HE2 LYS A 324 36.901 6.197 31.183 1.00 0.00 H

ATOM 5156 HZ1 LYS A 324 38.600 5.983 32.853 1.00 0.00 H

ATOM 5157 HZ2 LYS A 324 39.788 6.029 31.713 1.00 0.00 H

ATOM 5158 HZ3 LYS A 324 38.832 7.357 31.975 1.00 0.00 H

ATOM 5159 N GLU A 325 39.385 4.172 24.751 1.00 6.35 N

ATOM 5160 CA GLU A 325 40.391 4.130 23.690 1.00 6.60 C

ATOM 5161 C GLU A 325 39.826 3.629 22.349 1.00 7.09 C

ATOM 5162 O GLU A 325 40.219 4.132 21.296 1.00 7.25 O

ATOM 5163 CB GLU A 325 41.578 3.283 24.197 1.00 0.00 C

ATOM 5164 CG GLU A 325 42.793 3.236 23.250 1.00 0.00 C

ATOM 5165 CD GLU A 325 43.947 2.421 23.837 1.00 0.00 C

ATOM 5166 OE1 GLU A 325 44.313 1.413 23.195 1.00 0.00 O

ATOM 5167 OE2 GLU A 325 44.443 2.818 24.915 1.00 0.00 O1-

ATOM 5168 HB2 GLU A 325 41.898 3.678 25.163 1.00 0.00 H

ATOM 5169 HB3 GLU A 325 41.239 2.265 24.394 1.00 0.00 H

ATOM 5170 HG2 GLU A 325 42.516 2.807 22.287 1.00 0.00 H

ATOM 5171 HG3 GLU A 325 43.151 4.247 23.051 1.00 0.00 H

ATOM 5172 H GLU A 325 39.591 3.607 25.565 1.00 0.00 H

ATOM 5173 HA GLU A 325 40.743 5.152 23.539 1.00 0.00 H

ATOM 5174 N ALA A 326 38.876 2.687 22.426 1.00 6.74 N

ATOM 5175 CA ALA A 326 38.148 2.150 21.287 1.00 6.93 C

ATOM 5176 C ALA A 326 37.113 3.124 20.695 1.00 7.51 C

ATOM 5177 O ALA A 326 36.973 3.164 19.475 1.00 7.18 O

ATOM 5178 CB ALA A 326 37.498 0.850 21.731 1.00 7.22 C

ATOM 5179 H ALA A 326 38.615 2.321 23.331 1.00 0.00 H

ATOM 5180 HA ALA A 326 38.871 1.914 20.505 1.00 0.00 H

ATOM 5181 HB1 ALA A 326 36.979 0.389 20.904 1.00 0.00 H

ATOM 5182 HB2 ALA A 326 38.248 0.135 22.070 1.00 0.00 H

ATOM 5183 HB3 ALA A 326 36.785 1.017 22.537 1.00 0.00 H

ATOM 5184 N ILE A 327 36.436 3.930 21.533 1.00 7.06 N

ATOM 5185 CA ILE A 327 35.601 5.048 21.072 1.00 7.86 C

ATOM 5186 C ILE A 327 36.430 6.137 20.366 1.00 7.87 C

ATOM 5187 O ILE A 327 36.006 6.627 19.321 1.00 9.11 O

ATOM 5188 CB ILE A 327 34.714 5.647 22.217 1.00 0.00 C

ATOM 5189 CG1 ILE A 327 33.404 4.830 22.303 1.00 0.00 C

ATOM 5190 CG2 ILE A 327 34.404 7.163 22.126 1.00 0.00 C

ATOM 5191 CD1 ILE A 327 32.431 5.244 23.412 1.00 0.00 C

ATOM 5192 HB ILE A 327 35.244 5.507 23.160 1.00 0.00 H

ATOM 5193 HG12 ILE A 327 32.875 4.887 21.351 1.00 0.00 H

ATOM 5194 HG13 ILE A 327 33.652 3.779 22.446 1.00 0.00 H

ATOM 5195 HG21 ILE A 327 33.829 7.506 22.983 1.00 0.00 H

ATOM 5196 HG22 ILE A 327 35.305 7.775 22.132 1.00 0.00 H

ATOM 5197 HG23 ILE A 327 33.832 7.396 21.227 1.00 0.00 H

ATOM 5198 HD11 ILE A 327 31.570 4.580 23.405 1.00 0.00 H

ATOM 5199 HD12 ILE A 327 32.898 5.184 24.394 1.00 0.00 H

ATOM 5200 HD13 ILE A 327 32.039 6.251 23.272 1.00 0.00 H

ATOM 5201 H ILE A 327 36.559 3.841 22.533 1.00 0.00 H

ATOM 5202 HA ILE A 327 34.933 4.646 20.307 1.00 0.00 H

ATOM 5203 N ALA A 328 37.609 6.458 20.927 1.00 8.43 N

ATOM 5204 CA ALA A 328 38.558 7.418 20.367 1.00 8.67 C

ATOM 5205 C ALA A 328 39.217 6.979 19.051 1.00 8.76 C

ATOM 5206 O ALA A 328 39.691 7.848 18.323 1.00 9.02 O

ATOM 5207 CB ALA A 328 39.603 7.790 21.425 1.00 9.51 C

ATOM 5208 H ALA A 328 37.881 6.012 21.794 1.00 0.00 H

ATOM 5209 HA ALA A 328 38.003 8.322 20.127 1.00 0.00 H

ATOM 5210 HB1 ALA A 328 40.401 8.406 21.007 1.00 0.00 H

ATOM 5211 HB2 ALA A 328 39.138 8.372 22.219 1.00 0.00 H

ATOM 5212 HB3 ALA A 328 40.062 6.906 21.870 1.00 0.00 H

ATOM 5213 N SER A 329 39.193 5.672 18.731 1.00 8.43 N

ATOM 5214 CA SER A 329 39.661 5.131 17.450 1.00 8.25 C

ATOM 5215 C SER A 329 38.810 5.601 16.252 1.00 7.62 C

ATOM 5216 O SER A 329 39.336 5.652 15.140 1.00 8.63 O

ATOM 5217 CB SER A 329 39.685 3.593 17.505 1.00 0.00 C

ATOM 5218 OG SER A 329 40.624 3.131 18.454 1.00 0.00 O

ATOM 5219 HB2 SER A 329 38.701 3.183 17.719 1.00 0.00 H

ATOM 5220 HB3 SER A 329 39.978 3.186 16.536 1.00 0.00 H

ATOM 5221 HG SER A 329 40.383 3.465 19.324 1.00 0.00 H

ATOM 5222 H SER A 329 38.779 5.011 19.374 1.00 0.00 H

ATOM 5223 HA SER A 329 40.680 5.487 17.283 1.00 0.00 H

ATOM 5224 N ASN A 330 37.536 5.963 16.501 1.00 7.08 N

ATOM 5225 CA ASN A 330 36.614 6.505 15.500 1.00 6.53 C

ATOM 5226 C ASN A 330 37.022 7.899 14.986 1.00 6.40 C

ATOM 5227 O ASN A 330 36.757 8.172 13.816 1.00 6.46 O

ATOM 5228 CB ASN A 330 35.174 6.589 16.066 1.00 0.00 C

ATOM 5229 CG ASN A 330 34.519 5.251 16.429 1.00 0.00 C

ATOM 5230 OD1 ASN A 330 34.806 4.219 15.826 1.00 0.00 O

ATOM 5231 ND2 ASN A 330 33.605 5.271 17.401 1.00 0.00 N

ATOM 5232 HB2 ASN A 330 35.144 7.259 16.925 1.00 0.00 H

ATOM 5233 HB3 ASN A 330 34.532 7.037 15.310 1.00 0.00 H

ATOM 5234 HD22 ASN A 330 33.143 4.414 17.673 1.00 0.00 H

ATOM 5235 HD21 ASN A 330 33.383 6.132 17.879 1.00 0.00 H

ATOM 5236 H ASN A 330 37.177 5.897 17.444 1.00 0.00 H

ATOM 5237 HA ASN A 330 36.618 5.817 14.651 1.00 0.00 H

ATOM 5238 N PHE A 331 37.616 8.752 15.848 1.00 6.58 N

ATOM 5239 CA PHE A 331 37.775 10.185 15.569 1.00 7.01 C

ATOM 5240 C PHE A 331 39.166 10.792 15.812 1.00 7.67 C

ATOM 5241 O PHE A 331 39.411 11.864 15.264 1.00 8.20 O

ATOM 5242 CB PHE A 331 36.660 10.997 16.276 1.00 7.76 C

ATOM 5243 CG PHE A 331 36.560 10.840 17.787 1.00 6.53 C

ATOM 5244 CD1 PHE A 331 37.546 11.387 18.637 1.00 7.91 C

ATOM 5245 CD2 PHE A 331 35.532 10.056 18.351 1.00 6.97 C

ATOM 5246 CE1 PHE A 331 37.486 11.159 20.002 1.00 8.69 C

ATOM 5247 CE2 PHE A 331 35.471 9.874 19.726 1.00 7.99 C

ATOM 5248 CZ PHE A 331 36.445 10.422 20.549 1.00 8.06 C

ATOM 5249 H PHE A 331 37.826 8.450 16.788 1.00 0.00 H

ATOM 5250 HA PHE A 331 37.636 10.346 14.501 1.00 0.00 H

ATOM 5251 HB3 PHE A 331 35.694 10.761 15.830 1.00 0.00 H

ATOM 5252 HB2 PHE A 331 36.809 12.060 16.079 1.00 0.00 H

ATOM 5253 HD1 PHE A 331 38.358 11.972 18.233 1.00 0.00 H

ATOM 5254 HD2 PHE A 331 34.779 9.607 17.719 1.00 0.00 H

ATOM 5255 HE1 PHE A 331 38.260 11.547 20.639 1.00 0.00 H

ATOM 5256 HE2 PHE A 331 34.669 9.291 20.152 1.00 0.00 H

ATOM 5257 HZ PHE A 331 36.406 10.255 21.616 1.00 0.00 H

ATOM 5258 N LEU A 332 40.056 10.179 16.613 1.00 8.01 N

ATOM 5259 CA LEU A 332 41.345 10.803 16.961 1.00 8.50 C

ATOM 5260 C LEU A 332 42.305 10.956 15.761 1.00 8.29 C

ATOM 5261 O LEU A 332 43.050 11.934 15.716 1.00 8.98 O

ATOM 5262 CB LEU A 332 41.990 10.075 18.163 1.00 0.00 C

ATOM 5263 CG LEU A 332 43.257 10.745 18.754 1.00 0.00 C

ATOM 5264 CD1 LEU A 332 43.000 12.194 19.223 1.00 0.00 C

ATOM 5265 CD2 LEU A 332 43.867 9.877 19.874 1.00 0.00 C

ATOM 5266 HB2 LEU A 332 41.248 9.990 18.959 1.00 0.00 H

ATOM 5267 HB3 LEU A 332 42.228 9.052 17.865 1.00 0.00 H

ATOM 5268 HG LEU A 332 44.013 10.787 17.969 1.00 0.00 H

ATOM 5269 HD11 LEU A 332 43.498 12.426 20.165 1.00 0.00 H

ATOM 5270 HD12 LEU A 332 43.368 12.909 18.487 1.00 0.00 H

ATOM 5271 HD13 LEU A 332 41.939 12.390 19.369 1.00 0.00 H

ATOM 5272 HD21 LEU A 332 44.926 9.696 19.692 1.00 0.00 H

ATOM 5273 HD22 LEU A 332 43.779 10.338 20.858 1.00 0.00 H

ATOM 5274 HD23 LEU A 332 43.383 8.901 19.943 1.00 0.00 H

ATOM 5275 H LEU A 332 39.833 9.302 17.067 1.00 0.00 H

ATOM 5276 HA LEU A 332 41.106 11.817 17.288 1.00 0.00 H

ATOM 5277 N PHE A 333 42.223 10.030 14.789 1.00 8.34 N

ATOM 5278 CA PHE A 333 42.960 10.079 13.525 1.00 8.41 C

ATOM 5279 C PHE A 333 42.634 11.272 12.605 1.00 8.13 C

ATOM 5280 O PHE A 333 43.493 11.622 11.795 1.00 8.36 O

ATOM 5281 CB PHE A 333 42.770 8.761 12.749 1.00 0.00 C

ATOM 5282 CG PHE A 333 43.722 7.635 13.105 1.00 0.00 C

ATOM 5283 CD1 PHE A 333 43.415 6.726 14.139 1.00 0.00 C

ATOM 5284 CD2 PHE A 333 44.987 7.566 12.482 1.00 0.00 C

ATOM 5285 CE1 PHE A 333 44.324 5.731 14.475 1.00 0.00 C

ATOM 5286 CE2 PHE A 333 45.881 6.564 12.833 1.00 0.00 C

ATOM 5287 CZ PHE A 333 45.547 5.646 13.820 1.00 0.00 C

ATOM 5288 HB2 PHE A 333 41.743 8.407 12.849 1.00 0.00 H

ATOM 5289 HB3 PHE A 333 42.915 8.949 11.685 1.00 0.00 H

ATOM 5290 HD1 PHE A 333 42.469 6.788 14.658 1.00 0.00 H

ATOM 5291 HD2 PHE A 333 45.257 8.282 11.720 1.00 0.00 H

ATOM 5292 HE1 PHE A 333 44.080 5.020 15.251 1.00 0.00 H

ATOM 5293 HE2 PHE A 333 46.839 6.500 12.339 1.00 0.00 H

ATOM 5294 HZ PHE A 333 46.245 4.866 14.088 1.00 0.00 H

ATOM 5295 H PHE A 333 41.578 9.261 14.897 1.00 0.00 H

ATOM 5296 HA PHE A 333 44.020 10.178 13.770 1.00 0.00 H

ATOM 5297 N ILE A 334 41.428 11.864 12.715 1.00 7.33 N

ATOM 5298 CA ILE A 334 41.007 12.965 11.837 1.00 7.40 C

ATOM 5299 C ILE A 334 41.610 14.332 12.222 1.00 7.65 C

ATOM 5300 O ILE A 334 41.478 15.268 11.435 1.00 7.86 O

ATOM 5301 CB ILE A 334 39.457 13.098 11.679 1.00 7.06 C

ATOM 5302 CG1 ILE A 334 38.673 13.930 12.735 1.00 7.18 C

ATOM 5303 CG2 ILE A 334 38.802 11.725 11.439 1.00 8.14 C

ATOM 5304 CD1 ILE A 334 37.287 14.378 12.256 1.00 7.56 C

ATOM 5305 H ILE A 334 40.762 11.553 13.409 1.00 0.00 H

ATOM 5306 HA ILE A 334 41.391 12.731 10.846 1.00 0.00 H

ATOM 5307 HB ILE A 334 39.331 13.640 10.741 1.00 0.00 H

ATOM 5308 HG13 ILE A 334 39.205 14.830 13.038 1.00 0.00 H

ATOM 5309 HG12 ILE A 334 38.538 13.353 13.641 1.00 0.00 H

ATOM 5310 HG21 ILE A 334 37.765 11.832 11.131 1.00 0.00 H

ATOM 5311 HG22 ILE A 334 39.317 11.170 10.655 1.00 0.00 H

ATOM 5312 HG23 ILE A 334 38.811 11.112 12.340 1.00 0.00 H

ATOM 5313 HD11 ILE A 334 36.923 15.216 12.850 1.00 0.00 H

ATOM 5314 HD12 ILE A 334 37.318 14.706 11.219 1.00 0.00 H

ATOM 5315 HD13 ILE A 334 36.556 13.573 12.337 1.00 0.00 H

ATOM 5316 N ASN A 335 42.243 14.421 13.406 1.00 8.05 N

ATOM 5317 CA ASN A 335 42.814 15.638 13.979 1.00 8.23 C

ATOM 5318 C ASN A 335 43.916 16.249 13.090 1.00 8.52 C

ATOM 5319 O ASN A 335 44.968 15.631 12.919 1.00 8.92 O

ATOM 5320 CB ASN A 335 43.353 15.326 15.397 1.00 8.29 C

ATOM 5321 CG ASN A 335 43.493 16.578 16.264 1.00 8.39 C

ATOM 5322 OD1 ASN A 335 42.552 17.355 16.370 1.00 9.16 O

ATOM 5323 ND2 ASN A 335 44.648 16.779 16.898 1.00 9.95 N

ATOM 5324 H ASN A 335 42.320 13.592 13.979 1.00 0.00 H

ATOM 5325 HA ASN A 335 42.019 16.382 14.050 1.00 0.00 H

ATOM 5326 HB3 ASN A 335 44.283 14.755 15.362 1.00 0.00 H

ATOM 5327 HB2 ASN A 335 42.634 14.694 15.920 1.00 0.00 H

ATOM 5328 HD22 ASN A 335 44.765 17.621 17.454 1.00 0.00 H

ATOM 5329 HD21 ASN A 335 45.416 16.132 16.801 1.00 0.00 H

ATOM 5330 N GLY A 336 43.644 17.448 12.550 1.00 9.00 N

ATOM 5331 CA GLY A 336 44.554 18.194 11.680 1.00 9.56 C

ATOM 5332 C GLY A 336 44.632 17.609 10.258 1.00 10.18 C

ATOM 5333 O GLY A 336 45.668 17.761 9.612 1.00 11.68 O

ATOM 5334 H GLY A 336 42.738 17.871 12.717 1.00 0.00 H

ATOM 5335 HA3 GLY A 336 45.551 18.228 12.123 1.00 0.00 H

ATOM 5336 HA2 GLY A 336 44.201 19.223 11.614 1.00 0.00 H

ATOM 5337 N THR A 337 43.575 16.927 9.783 1.00 9.69 N

ATOM 5338 CA THR A 337 43.505 16.285 8.463 1.00 9.43 C

ATOM 5339 C THR A 337 42.131 16.542 7.816 1.00 9.03 C

ATOM 5340 O THR A 337 41.183 16.873 8.522 1.00 9.57 O

ATOM 5341 CB THR A 337 43.715 14.743 8.557 1.00 9.13 C

ATOM 5342 OG1 THR A 337 42.556 14.043 8.952 1.00 9.97 O

ATOM 5343 CG2 THR A 337 44.882 14.289 9.444 1.00 10.14 C

ATOM 5344 H THR A 337 42.737 16.859 10.348 1.00 0.00 H

ATOM 5345 HA THR A 337 44.259 16.711 7.798 1.00 0.00 H

ATOM 5346 HB THR A 337 43.938 14.391 7.549 1.00 0.00 H

ATOM 5347 HG1 THR A 337 42.293 14.371 9.818 1.00 0.00 H

ATOM 5348 HG21 THR A 337 45.039 13.213 9.363 1.00 0.00 H

ATOM 5349 HG22 THR A 337 45.811 14.781 9.153 1.00 0.00 H

ATOM 5350 HG23 THR A 337 44.699 14.510 10.495 1.00 0.00 H

ATOM 5351 N ASP A 338 42.029 16.349 6.491 1.00 7.99 N

ATOM 5352 CA ASP A 338 40.783 16.482 5.719 1.00 7.97 C

ATOM 5353 C ASP A 338 39.842 15.255 5.835 1.00 7.80 C

ATOM 5354 O ASP A 338 38.875 15.168 5.077 1.00 8.09 O

ATOM 5355 CB ASP A 338 41.032 16.866 4.237 1.00 8.10 C

ATOM 5356 CG ASP A 338 41.916 15.907 3.429 1.00 8.89 C

ATOM 5357 OD1 ASP A 338 42.544 15.010 4.034 1.00 10.37 O

ATOM 5358 OD2 ASP A 338 41.951 16.092 2.195 1.00 10.39 O1-

ATOM 5359 H ASP A 338 42.832 16.045 5.957 1.00 0.00 H

ATOM 5360 HA ASP A 338 40.220 17.308 6.150 1.00 0.00 H

ATOM 5361 HB3 ASP A 338 41.511 17.846 4.217 1.00 0.00 H

ATOM 5362 HB2 ASP A 338 40.075 16.977 3.726 1.00 0.00 H

ATOM 5363 N SER A 339 40.129 14.332 6.769 1.00 7.52 N

ATOM 5364 CA SER A 339 39.304 13.164 7.076 1.00 7.24 C

ATOM 5365 C SER A 339 38.058 13.538 7.893 1.00 6.99 C

ATOM 5366 O SER A 339 37.980 14.644 8.434 1.00 7.06 O

ATOM 5367 CB SER A 339 40.156 12.142 7.849 1.00 0.00 C

ATOM 5368 OG SER A 339 41.189 11.625 7.033 1.00 0.00 O

ATOM 5369 HB2 SER A 339 40.571 12.582 8.749 1.00 0.00 H

ATOM 5370 HB3 SER A 339 39.549 11.308 8.195 1.00 0.00 H

ATOM 5371 HG SER A 339 41.849 12.313 6.914 1.00 0.00 H

ATOM 5372 H SER A 339 40.933 14.472 7.364 1.00 0.00 H

ATOM 5373 HA SER A 339 38.967 12.715 6.145 1.00 0.00 H

ATOM 5374 N TRP A 340 37.117 12.584 7.998 1.00 6.90 N

ATOM 5375 CA TRP A 340 35.956 12.719 8.869 1.00 6.58 C

ATOM 5376 C TRP A 340 35.540 11.372 9.472 1.00 6.86 C

ATOM 5377 O TRP A 340 35.827 10.314 8.912 1.00 7.62 O

ATOM 5378 CB TRP A 340 34.814 13.441 8.139 1.00 7.05 C

ATOM 5379 CG TRP A 340 33.724 13.916 9.046 1.00 5.67 C

ATOM 5380 CD1 TRP A 340 32.513 13.342 9.209 1.00 5.75 C

ATOM 5381 CD2 TRP A 340 33.785 15.005 10.012 1.00 6.26 C

ATOM 5382 NE1 TRP A 340 31.809 14.018 10.178 1.00 6.79 N

ATOM 5383 CE2 TRP A 340 32.541 15.058 10.709 1.00 6.27 C

ATOM 5384 CE3 TRP A 340 34.768 15.955 10.373 1.00 6.61 C

ATOM 5385 CZ2 TRP A 340 32.276 16.019 11.695 1.00 6.71 C

ATOM 5386 CZ3 TRP A 340 34.518 16.918 11.370 1.00 6.34 C

ATOM 5387 CH2 TRP A 340 33.268 16.959 12.015 1.00 6.01 C

ATOM 5388 H TRP A 340 37.233 11.691 7.539 1.00 0.00 H

ATOM 5389 HA TRP A 340 36.255 13.343 9.713 1.00 0.00 H

ATOM 5390 HB3 TRP A 340 34.396 12.805 7.363 1.00 0.00 H

ATOM 5391 HB2 TRP A 340 35.206 14.323 7.631 1.00 0.00 H

ATOM 5392 HD1 TRP A 340 32.164 12.478 8.665 1.00 0.00 H

ATOM 5393 HE1 TRP A 340 30.863 13.755 10.432 1.00 0.00 H

ATOM 5394 HE3 TRP A 340 35.722 15.945 9.868 1.00 0.00 H

ATOM 5395 HZ2 TRP A 340 31.320 16.036 12.193 1.00 0.00 H

ATOM 5396 HZ3 TRP A 340 35.280 17.637 11.631 1.00 0.00 H

ATOM 5397 HH2 TRP A 340 33.069 17.719 12.749 1.00 0.00 H

ATOM 5398 N ALA A 341 34.895 11.474 10.642 1.00 7.12 N

ATOM 5399 CA ALA A 341 34.564 10.380 11.542 1.00 7.08 C

ATOM 5400 C ALA A 341 33.052 10.146 11.633 1.00 7.13 C

ATOM 5401 O ALA A 341 32.258 10.999 11.238 1.00 6.59 O

ATOM 5402 CB ALA A 341 35.097 10.777 12.926 1.00 0.00 C

ATOM 5403 HB1 ALA A 341 34.661 11.715 13.275 1.00 0.00 H

ATOM 5404 HB2 ALA A 341 34.879 10.017 13.676 1.00 0.00 H

ATOM 5405 HB3 ALA A 341 36.177 10.913 12.894 1.00 0.00 H

ATOM 5406 H ALA A 341 34.666 12.395 10.988 1.00 0.00 H

ATOM 5407 HA ALA A 341 35.046 9.454 11.222 1.00 0.00 H

ATOM 5408 N THR A 342 32.697 9.002 12.228 1.00 5.93 N

ATOM 5409 CA THR A 342 31.347 8.695 12.692 1.00 6.21 C

ATOM 5410 C THR A 342 31.364 8.573 14.227 1.00 6.04 C

ATOM 5411 O THR A 342 32.403 8.289 14.825 1.00 6.15 O

ATOM 5412 CB THR A 342 30.833 7.352 12.105 1.00 6.24 C

ATOM 5413 OG1 THR A 342 31.566 6.231 12.568 1.00 6.01 O

ATOM 5414 CG2 THR A 342 30.809 7.347 10.571 1.00 7.22 C

ATOM 5415 H THR A 342 33.413 8.360 12.536 1.00 0.00 H

ATOM 5416 HA THR A 342 30.651 9.489 12.416 1.00 0.00 H

ATOM 5417 HB THR A 342 29.805 7.202 12.441 1.00 0.00 H

ATOM 5418 HG1 THR A 342 32.364 6.146 12.035 1.00 0.00 H

ATOM 5419 HG21 THR A 342 30.427 6.403 10.185 1.00 0.00 H

ATOM 5420 HG22 THR A 342 30.163 8.140 10.202 1.00 0.00 H

ATOM 5421 HG23 THR A 342 31.797 7.504 10.143 1.00 0.00 H

ATOM 5422 N THR A 343 30.186 8.775 14.826 1.00 5.97 N

ATOM 5423 CA THR A 343 29.904 8.561 16.241 1.00 6.84 C

ATOM 5424 C THR A 343 28.569 7.813 16.342 1.00 5.74 C

ATOM 5425 O THR A 343 27.569 8.256 15.782 1.00 5.69 O

ATOM 5426 CB THR A 343 29.948 9.868 17.109 1.00 0.00 C

ATOM 5427 OG1 THR A 343 28.929 9.984 18.086 1.00 0.00 O

ATOM 5428 CG2 THR A 343 29.958 11.191 16.330 1.00 0.00 C

ATOM 5429 HB THR A 343 30.879 9.835 17.676 1.00 0.00 H

ATOM 5430 HG1 THR A 343 29.003 9.271 18.733 1.00 0.00 H

ATOM 5431 HG21 THR A 343 30.023 12.044 17.004 1.00 0.00 H

ATOM 5432 HG22 THR A 343 30.815 11.246 15.662 1.00 0.00 H

ATOM 5433 HG23 THR A 343 29.055 11.313 15.737 1.00 0.00 H

ATOM 5434 H THR A 343 29.386 9.042 14.266 1.00 0.00 H

ATOM 5435 HA THR A 343 30.668 7.891 16.641 1.00 0.00 H

ATOM 5436 N TYR A 344 28.601 6.660 17.019 1.00 5.05 N

ATOM 5437 CA TYR A 344 27.471 5.752 17.172 1.00 5.39 C

ATOM 5438 C TYR A 344 27.801 4.758 18.289 1.00 5.39 C

ATOM 5439 O TYR A 344 28.967 4.597 18.654 1.00 5.79 O

ATOM 5440 CB TYR A 344 27.156 5.038 15.828 1.00 5.21 C

ATOM 5441 CG TYR A 344 28.063 3.889 15.424 1.00 5.09 C

ATOM 5442 CD1 TYR A 344 29.272 4.155 14.750 1.00 6.52 C

ATOM 5443 CD2 TYR A 344 27.704 2.556 15.719 1.00 4.99 C

ATOM 5444 CE1 TYR A 344 30.120 3.095 14.377 1.00 5.82 C

ATOM 5445 CE2 TYR A 344 28.558 1.498 15.356 1.00 5.35 C

ATOM 5446 CZ TYR A 344 29.762 1.766 14.678 1.00 5.55 C

ATOM 5447 OH TYR A 344 30.592 0.740 14.337 1.00 5.91 O

ATOM 5448 H TYR A 344 29.456 6.360 17.467 1.00 0.00 H

ATOM 5449 HA TYR A 344 26.601 6.334 17.485 1.00 0.00 H

ATOM 5450 HB3 TYR A 344 27.161 5.753 15.009 1.00 0.00 H

ATOM 5451 HB2 TYR A 344 26.138 4.648 15.873 1.00 0.00 H

ATOM 5452 HD1 TYR A 344 29.556 5.172 14.520 1.00 0.00 H

ATOM 5453 HD2 TYR A 344 26.782 2.342 16.239 1.00 0.00 H

ATOM 5454 HE1 TYR A 344 31.050 3.309 13.872 1.00 0.00 H

ATOM 5455 HE2 TYR A 344 28.293 0.482 15.606 1.00 0.00 H

ATOM 5456 HH TYR A 344 31.435 1.035 13.986 1.00 0.00 H

ATOM 5457 N ILE A 345 26.761 4.066 18.765 1.00 5.35 N

ATOM 5458 CA ILE A 345 26.899 2.996 19.748 1.00 5.71 C

ATOM 5459 C ILE A 345 25.880 1.857 19.509 1.00 5.44 C

ATOM 5460 O ILE A 345 26.086 0.762 20.032 1.00 5.71 O

ATOM 5461 CB ILE A 345 26.817 3.575 21.194 1.00 0.00 C

ATOM 5462 CG1 ILE A 345 27.363 2.629 22.284 1.00 0.00 C

ATOM 5463 CG2 ILE A 345 25.428 4.128 21.555 1.00 0.00 C

ATOM 5464 CD1 ILE A 345 28.861 2.341 22.119 1.00 0.00 C

ATOM 5465 HB ILE A 345 27.476 4.445 21.218 1.00 0.00 H

ATOM 5466 HG12 ILE A 345 27.220 3.088 23.263 1.00 0.00 H

ATOM 5467 HG13 ILE A 345 26.806 1.695 22.309 1.00 0.00 H

ATOM 5468 HG21 ILE A 345 25.413 4.538 22.565 1.00 0.00 H

ATOM 5469 HG22 ILE A 345 25.169 4.938 20.877 1.00 0.00 H

ATOM 5470 HG23 ILE A 345 24.639 3.384 21.488 1.00 0.00 H

ATOM 5471 HD11 ILE A 345 29.301 2.029 23.063 1.00 0.00 H

ATOM 5472 HD12 ILE A 345 29.036 1.544 21.396 1.00 0.00 H

ATOM 5473 HD13 ILE A 345 29.402 3.229 21.794 1.00 0.00 H

ATOM 5474 H ILE A 345 25.830 4.265 18.432 1.00 0.00 H

ATOM 5475 HA ILE A 345 27.874 2.528 19.604 1.00 0.00 H

ATOM 5476 N GLU A 346 24.847 2.104 18.681 1.00 5.52 N

ATOM 5477 CA GLU A 346 23.889 1.099 18.224 1.00 5.53 C

ATOM 5478 C GLU A 346 23.724 1.161 16.701 1.00 5.32 C

ATOM 5479 O GLU A 346 23.897 2.214 16.084 1.00 5.62 O

ATOM 5480 CB GLU A 346 22.519 1.300 18.904 1.00 5.15 C

ATOM 5481 CG GLU A 346 22.528 1.037 20.420 1.00 5.28 C

ATOM 5482 CD GLU A 346 21.164 1.206 21.091 1.00 5.91 C

ATOM 5483 OE1 GLU A 346 20.134 1.149 20.385 1.00 5.91 O

ATOM 5484 OE2 GLU A 346 21.173 1.368 22.331 1.00 6.43 O1-

ATOM 5485 H GLU A 346 24.732 3.018 18.270 1.00 0.00 H

ATOM 5486 HA GLU A 346 24.251 0.097 18.467 1.00 0.00 H

ATOM 5487 HB3 GLU A 346 21.785 0.640 18.437 1.00 0.00 H

ATOM 5488 HB2 GLU A 346 22.171 2.316 18.722 1.00 0.00 H

ATOM 5489 HG3 GLU A 346 23.220 1.724 20.899 1.00 0.00 H

ATOM 5490 HG2 GLU A 346 22.890 0.029 20.624 1.00 0.00 H

ATOM 5491 N ASN A 347 23.321 0.007 16.160 1.00 5.31 N

ATOM 5492 CA ASN A 347 22.832 -0.217 14.804 1.00 5.18 C

ATOM 5493 C ASN A 347 22.051 -1.546 14.797 1.00 5.30 C

ATOM 5494 O ASN A 347 21.866 -2.160 15.851 1.00 5.85 O

ATOM 5495 CB ASN A 347 23.961 -0.095 13.739 1.00 5.12 C

ATOM 5496 CG ASN A 347 25.141 -1.057 13.895 1.00 5.09 C

ATOM 5497 OD1 ASN A 347 24.953 -2.265 13.978 1.00 5.23 O

ATOM 5498 ND2 ASN A 347 26.366 -0.533 13.893 1.00 6.22 N

ATOM 5499 H ASN A 347 23.198 -0.790 16.769 1.00 0.00 H

ATOM 5500 HA ASN A 347 22.108 0.573 14.610 1.00 0.00 H

ATOM 5501 HB3 ASN A 347 24.336 0.929 13.729 1.00 0.00 H

ATOM 5502 HB2 ASN A 347 23.553 -0.259 12.742 1.00 0.00 H

ATOM 5503 HD22 ASN A 347 27.173 -1.136 13.962 1.00 0.00 H

ATOM 5504 HD21 ASN A 347 26.499 0.464 13.807 1.00 0.00 H

ATOM 5505 N HIS A 348 21.607 -1.975 13.607 1.00 5.26 N

ATOM 5506 CA HIS A 348 20.861 -3.220 13.398 1.00 4.66 C

ATOM 5507 C HIS A 348 21.670 -4.525 13.636 1.00 4.72 C

ATOM 5508 O HIS A 348 21.067 -5.598 13.657 1.00 4.69 O

ATOM 5509 CB HIS A 348 20.246 -3.209 11.992 1.00 5.22 C

ATOM 5510 CG HIS A 348 21.252 -3.435 10.899 1.00 4.26 C

ATOM 5511 ND1 HIS A 348 22.257 -2.507 10.611 1.00 5.00 N

ATOM 5512 CD2 HIS A 348 21.419 -4.548 10.106 1.00 5.59 C

ATOM 5513 CE1 HIS A 348 22.992 -3.093 9.681 1.00 5.00 C

ATOM 5514 NE2 HIS A 348 22.554 -4.308 9.358 1.00 5.00 N

ATOM 5515 H HIS A 348 21.799 -1.432 12.778 1.00 0.00 H

ATOM 5516 HA HIS A 348 20.029 -3.182 14.093 1.00 0.00 H

ATOM 5517 HB3 HIS A 348 19.714 -2.276 11.808 1.00 0.00 H

ATOM 5518 HB2 HIS A 348 19.501 -4.001 11.933 1.00 0.00 H

ATOM 5519 HD2 HIS A 348 20.874 -5.477 10.048 1.00 0.00 H

ATOM 5520 HE1 HIS A 348 23.860 -2.640 9.236 1.00 0.00 H

ATOM 5521 HE2 HIS A 348 22.982 -4.937 8.694 1.00 0.00 H

ATOM 5522 N ASP A 349 23.002 -4.415 13.773 1.00 4.40 N

ATOM 5523 CA ASP A 349 23.943 -5.510 14.035 1.00 4.63 C

ATOM 5524 C ASP A 349 24.459 -5.532 15.483 1.00 4.98 C

ATOM 5525 O ASP A 349 25.339 -6.341 15.778 1.00 5.13 O

ATOM 5526 CB ASP A 349 25.137 -5.538 13.045 1.00 5.02 C

ATOM 5527 CG ASP A 349 24.736 -5.583 11.572 1.00 5.11 C

ATOM 5528 OD1 ASP A 349 23.810 -6.363 11.256 1.00 6.26 O

ATOM 5529 OD2 ASP A 349 25.436 -4.927 10.771 1.00 6.06 O1-

ATOM 5530 H ASP A 349 23.424 -3.497 13.715 1.00 0.00 H

ATOM 5531 HA ASP A 349 23.412 -6.447 13.915 1.00 0.00 H

ATOM 5532 HB3 ASP A 349 25.787 -6.394 13.230 1.00 0.00 H

ATOM 5533 HB2 ASP A 349 25.747 -4.648 13.204 1.00 0.00 H

ATOM 5534 N GLN A 350 23.932 -4.658 16.357 1.00 5.29 N

ATOM 5535 CA GLN A 350 24.447 -4.467 17.711 1.00 5.64 C

ATOM 5536 C GLN A 350 23.376 -4.620 18.779 1.00 5.71 C

ATOM 5537 O GLN A 350 22.260 -4.143 18.607 1.00 5.53 O

ATOM 5538 CB GLN A 350 25.078 -3.058 17.818 1.00 6.21 C

ATOM 5539 CG GLN A 350 26.394 -2.883 17.041 1.00 6.16 C

ATOM 5540 CD GLN A 350 27.437 -3.940 17.391 1.00 6.25 C

ATOM 5541 OE1 GLN A 350 27.541 -4.349 18.547 1.00 7.96 O

ATOM 5542 NE2 GLN A 350 28.210 -4.382 16.398 1.00 3.69 N

ATOM 5543 H GLN A 350 23.191 -4.034 16.067 1.00 0.00 H

ATOM 5544 HA GLN A 350 25.185 -5.235 17.941 1.00 0.00 H

ATOM 5545 HB3 GLN A 350 25.271 -2.816 18.865 1.00 0.00 H

ATOM 5546 HB2 GLN A 350 24.363 -2.309 17.476 1.00 0.00 H

ATOM 5547 HG3 GLN A 350 26.808 -1.892 17.225 1.00 0.00 H

ATOM 5548 HG2 GLN A 350 26.199 -2.945 15.975 1.00 0.00 H

ATOM 5549 HE22 GLN A 350 28.921 -5.077 16.573 1.00 0.00 H

ATOM 5550 HE21 GLN A 350 28.084 -4.032 15.459 1.00 0.00 H

ATOM 5551 N ALA A 351 23.793 -5.178 19.925 1.00 5.19 N

ATOM 5552 CA ALA A 351 23.051 -5.108 21.175 1.00 5.10 C

ATOM 5553 C ALA A 351 22.932 -3.672 21.704 1.00 5.23 C

ATOM 5554 O ALA A 351 23.717 -2.799 21.326 1.00 5.09 O

ATOM 5555 CB ALA A 351 23.728 -6.024 22.195 1.00 0.00 C

ATOM 5556 HB1 ALA A 351 24.783 -5.780 22.320 1.00 0.00 H

ATOM 5557 HB2 ALA A 351 23.254 -5.950 23.173 1.00 0.00 H

ATOM 5558 HB3 ALA A 351 23.660 -7.064 21.875 1.00 0.00 H

ATOM 5559 H ALA A 351 24.733 -5.540 19.986 1.00 0.00 H

ATOM 5560 HA ALA A 351 22.043 -5.465 20.986 1.00 0.00 H

ATOM 5561 N ARG A 352 21.926 -3.460 22.563 1.00 4.92 N

ATOM 5562 CA ARG A 352 21.593 -2.156 23.117 1.00 5.36 C

ATOM 5563 C ARG A 352 22.683 -1.624 24.052 1.00 5.68 C

ATOM 5564 O ARG A 352 23.194 -2.360 24.897 1.00 5.88 O

ATOM 5565 CB ARG A 352 20.224 -2.220 23.809 1.00 5.54 C

ATOM 5566 CG ARG A 352 19.067 -2.606 22.866 1.00 5.52 C

ATOM 5567 CD ARG A 352 18.851 -1.595 21.733 1.00 6.62 C

ATOM 5568 NE ARG A 352 17.681 -1.935 20.918 1.00 5.65 N

ATOM 5569 CZ ARG A 352 17.083 -1.096 20.056 1.00 5.09 C

ATOM 5570 NH1 ARG A 352 17.518 0.158 19.895 1.00 4.41 N

ATOM 5571 NH2 ARG A 352 16.035 -1.513 19.336 1.00 5.99 N1+

ATOM 5572 H ARG A 352 21.350 -4.234 22.866 1.00 0.00 H

ATOM 5573 HA ARG A 352 21.526 -1.464 22.278 1.00 0.00 H

ATOM 5574 HB3 ARG A 352 20.018 -1.249 24.251 1.00 0.00 H

ATOM 5575 HB2 ARG A 352 20.264 -2.924 24.640 1.00 0.00 H

ATOM 5576 HG3 ARG A 352 18.143 -2.699 23.429 1.00 0.00 H

ATOM 5577 HG2 ARG A 352 19.257 -3.594 22.446 1.00 0.00 H

ATOM 5578 HD3 ARG A 352 19.700 -1.575 21.051 1.00 0.00 H

ATOM 5579 HD2 ARG A 352 18.743 -0.596 22.158 1.00 0.00 H

ATOM 5580 HE ARG A 352 17.377 -2.901 20.958 1.00 0.00 H

ATOM 5581 HH12 ARG A 352 17.055 0.771 19.233 1.00 0.00 H

ATOM 5582 HH11 ARG A 352 18.371 0.480 20.348 1.00 0.00 H

ATOM 5583 HH22 ARG A 352 15.598 -0.883 18.677 1.00 0.00 H

ATOM 5584 HH21 ARG A 352 15.694 -2.460 19.420 1.00 0.00 H

ATOM 5585 N SER A 353 23.013 -0.342 23.846 1.00 5.37 N

ATOM 5586 CA SER A 353 24.095 0.376 24.508 1.00 5.43 C

ATOM 5587 C SER A 353 23.941 0.505 26.028 1.00 5.82 C

ATOM 5588 O SER A 353 24.959 0.490 26.715 1.00 5.88 O

ATOM 5589 CB SER A 353 24.289 1.735 23.819 1.00 6.00 C

ATOM 5590 OG SER A 353 23.176 2.596 23.969 1.00 6.01 O

ATOM 5591 H SER A 353 22.496 0.190 23.156 1.00 0.00 H

ATOM 5592 HA SER A 353 25.002 -0.205 24.329 1.00 0.00 H

ATOM 5593 HB3 SER A 353 24.489 1.574 22.762 1.00 0.00 H

ATOM 5594 HB2 SER A 353 25.162 2.242 24.231 1.00 0.00 H

ATOM 5595 HG SER A 353 22.448 2.255 23.433 1.00 0.00 H

ATOM 5596 N ILE A 354 22.696 0.591 26.528 1.00 5.53 N

ATOM 5597 CA ILE A 354 22.409 0.643 27.963 1.00 5.63 C

ATOM 5598 C ILE A 354 22.743 -0.672 28.686 1.00 5.92 C

ATOM 5599 O ILE A 354 23.279 -0.622 29.790 1.00 6.16 O

ATOM 5600 CB ILE A 354 20.947 1.080 28.259 1.00 6.10 C

ATOM 5601 CG1 ILE A 354 20.785 2.564 27.892 1.00 5.42 C

ATOM 5602 CG2 ILE A 354 20.475 0.872 29.718 1.00 7.18 C

ATOM 5603 CD1 ILE A 354 19.331 3.023 27.801 1.00 7.59 C

ATOM 5604 H ILE A 354 21.903 0.612 25.903 1.00 0.00 H

ATOM 5605 HA ILE A 354 23.065 1.405 28.377 1.00 0.00 H

ATOM 5606 HB ILE A 354 20.289 0.498 27.612 1.00 0.00 H

ATOM 5607 HG13 ILE A 354 21.268 2.785 26.940 1.00 0.00 H

ATOM 5608 HG12 ILE A 354 21.312 3.151 28.642 1.00 0.00 H

ATOM 5609 HG21 ILE A 354 19.456 1.222 29.871 1.00 0.00 H

ATOM 5610 HG22 ILE A 354 20.475 -0.179 29.987 1.00 0.00 H

ATOM 5611 HG23 ILE A 354 21.115 1.399 30.426 1.00 0.00 H

ATOM 5612 HD11 ILE A 354 19.269 3.974 27.273 1.00 0.00 H

ATOM 5613 HD12 ILE A 354 18.736 2.292 27.258 1.00 0.00 H

ATOM 5614 HD13 ILE A 354 18.882 3.164 28.782 1.00 0.00 H

ATOM 5615 N THR A 355 22.475 -1.818 28.042 1.00 5.86 N

ATOM 5616 CA THR A 355 22.861 -3.130 28.560 1.00 5.90 C

ATOM 5617 C THR A 355 24.386 -3.353 28.520 1.00 6.10 C

ATOM 5618 O THR A 355 24.941 -3.895 29.474 1.00 6.90 O

ATOM 5619 CB THR A 355 22.193 -4.278 27.761 1.00 6.16 C

ATOM 5620 OG1 THR A 355 20.791 -4.107 27.793 1.00 6.42 O

ATOM 5621 CG2 THR A 355 22.537 -5.684 28.295 1.00 6.19 C

ATOM 5622 H THR A 355 22.038 -1.803 27.132 1.00 0.00 H

ATOM 5623 HA THR A 355 22.540 -3.197 29.601 1.00 0.00 H

ATOM 5624 HB THR A 355 22.487 -4.226 26.712 1.00 0.00 H

ATOM 5625 HG1 THR A 355 20.484 -4.348 28.678 1.00 0.00 H

ATOM 5626 HG21 THR A 355 21.889 -6.454 27.884 1.00 0.00 H

ATOM 5627 HG22 THR A 355 23.558 -5.965 28.041 1.00 0.00 H

ATOM 5628 HG23 THR A 355 22.448 -5.732 29.380 1.00 0.00 H

ATOM 5629 N ARG A 356 25.019 -2.933 27.414 1.00 6.13 N

ATOM 5630 CA ARG A 356 26.435 -3.147 27.128 1.00 5.92 C

ATOM 5631 C ARG A 356 27.380 -2.241 27.935 1.00 6.27 C

ATOM 5632 O ARG A 356 28.398 -2.738 28.415 1.00 6.66 O

ATOM 5633 CB ARG A 356 26.658 -2.946 25.615 1.00 5.92 C

ATOM 5634 CG ARG A 356 26.047 -4.056 24.739 1.00 6.62 C

ATOM 5635 CD ARG A 356 26.965 -5.279 24.583 1.00 5.95 C

ATOM 5636 NE ARG A 356 28.110 -4.982 23.704 1.00 6.82 N

ATOM 5637 CZ ARG A 356 29.415 -5.191 23.949 1.00 7.29 C

ATOM 5638 NH1 ARG A 356 29.857 -5.674 25.119 1.00 6.34 N

ATOM 5639 NH2 ARG A 356 30.307 -4.907 22.994 1.00 6.07 N1+

ATOM 5640 H ARG A 356 24.482 -2.489 26.681 1.00 0.00 H

ATOM 5641 HA ARG A 356 26.673 -4.182 27.382 1.00 0.00 H

ATOM 5642 HB3 ARG A 356 27.723 -2.865 25.395 1.00 0.00 H

ATOM 5643 HB2 ARG A 356 26.224 -1.988 25.328 1.00 0.00 H

ATOM 5644 HG3 ARG A 356 25.956 -3.615 23.745 1.00 0.00 H

ATOM 5645 HG2 ARG A 356 25.031 -4.333 25.020 1.00 0.00 H

ATOM 5646 HD3 ARG A 356 26.428 -6.184 24.303 1.00 0.00 H

ATOM 5647 HD2 ARG A 356 27.384 -5.483 25.567 1.00 0.00 H

ATOM 5648 HE ARG A 356 27.861 -4.611 22.798 1.00 0.00 H

ATOM 5649 HH12 ARG A 356 30.850 -5.816 25.257 1.00 0.00 H

ATOM 5650 HH11 ARG A 356 29.212 -5.884 25.865 1.00 0.00 H

ATOM 5651 HH22 ARG A 356 31.291 -5.069 23.174 1.00 0.00 H

ATOM 5652 HH21 ARG A 356 30.018 -4.568 22.087 1.00 0.00 H

ATOM 5653 N PHE A 357 27.062 -0.938 28.017 1.00 6.27 N

ATOM 5654 CA PHE A 357 28.001 0.111 28.437 1.00 6.75 C

ATOM 5655 C PHE A 357 27.444 1.004 29.555 1.00 6.82 C

ATOM 5656 O PHE A 357 28.106 1.975 29.918 1.00 6.98 O

ATOM 5657 CB PHE A 357 28.378 0.968 27.208 1.00 6.78 C

ATOM 5658 CG PHE A 357 28.859 0.174 26.007 1.00 7.38 C

ATOM 5659 CD1 PHE A 357 30.015 -0.631 26.099 1.00 7.56 C

ATOM 5660 CD2 PHE A 357 28.114 0.177 24.810 1.00 7.52 C

ATOM 5661 CE1 PHE A 357 30.412 -1.396 25.011 1.00 7.87 C

ATOM 5662 CE2 PHE A 357 28.533 -0.591 23.732 1.00 8.84 C

ATOM 5663 CZ PHE A 357 29.679 -1.369 23.831 1.00 7.36 C

ATOM 5664 H PHE A 357 26.199 -0.610 27.603 1.00 0.00 H

ATOM 5665 HA PHE A 357 28.914 -0.335 28.837 1.00 0.00 H

ATOM 5666 HB3 PHE A 357 29.174 1.661 27.483 1.00 0.00 H

ATOM 5667 HB2 PHE A 357 27.532 1.586 26.905 1.00 0.00 H

ATOM 5668 HD1 PHE A 357 30.590 -0.657 27.012 1.00 0.00 H

ATOM 5669 HD2 PHE A 357 27.219 0.775 24.727 1.00 0.00 H

ATOM 5670 HE1 PHE A 357 31.300 -2.008 25.080 1.00 0.00 H

ATOM 5671 HE2 PHE A 357 27.965 -0.583 22.813 1.00 0.00 H

ATOM 5672 HZ PHE A 357 30.001 -1.960 22.988 1.00 0.00 H

ATOM 5673 N ALA A 358 26.258 0.672 30.081 1.00 6.93 N

ATOM 5674 CA ALA A 358 25.586 1.416 31.140 1.00 6.85 C

ATOM 5675 C ALA A 358 25.079 0.400 32.181 1.00 7.92 C

ATOM 5676 O ALA A 358 25.850 -0.490 32.543 1.00 8.03 O

ATOM 5677 CB ALA A 358 24.549 2.350 30.486 1.00 0.00 C

ATOM 5678 HB1 ALA A 358 24.777 2.494 29.433 1.00 0.00 H

ATOM 5679 HB2 ALA A 358 23.525 1.987 30.545 1.00 0.00 H

ATOM 5680 HB3 ALA A 358 24.592 3.344 30.920 1.00 0.00 H

ATOM 5681 H ALA A 358 25.778 -0.154 29.751 1.00 0.00 H

ATOM 5682 HA ALA A 358 26.313 2.037 31.661 1.00 0.00 H

ATOM 5683 N ASP A 359 23.826 0.519 32.649 1.00 7.53 N

ATOM 5684 CA ASP A 359 23.197 -0.482 33.511 1.00 8.60 C

ATOM 5685 C ASP A 359 21.695 -0.491 33.195 1.00 7.89 C

ATOM 5686 O ASP A 359 21.020 0.503 33.455 1.00 7.79 O

ATOM 5687 CB ASP A 359 23.532 -0.246 35.007 1.00 9.28 C

ATOM 5688 CG ASP A 359 23.079 -1.347 35.978 1.00 11.46 C

ATOM 5689 OD1 ASP A 359 22.139 -2.102 35.648 1.00 11.77 O

ATOM 5690 OD2 ASP A 359 23.648 -1.376 37.089 1.00 15.51 O1-

ATOM 5691 H ASP A 359 23.236 1.282 32.344 1.00 0.00 H

ATOM 5692 HA ASP A 359 23.586 -1.466 33.236 1.00 0.00 H

ATOM 5693 HB3 ASP A 359 23.120 0.708 35.335 1.00 0.00 H

ATOM 5694 HB2 ASP A 359 24.616 -0.176 35.095 1.00 0.00 H

ATOM 5695 N ASP A 360 21.207 -1.624 32.655 1.00 7.80 N

ATOM 5696 CA ASP A 360 19.820 -1.816 32.216 1.00 8.24 C

ATOM 5697 C ASP A 360 18.836 -2.178 33.345 1.00 8.22 C

ATOM 5698 O ASP A 360 17.665 -2.404 33.035 1.00 8.13 O

ATOM 5699 CB ASP A 360 19.681 -2.816 31.039 1.00 8.34 C

ATOM 5700 CG ASP A 360 20.154 -4.253 31.276 1.00 9.01 C

ATOM 5701 OD1 ASP A 360 20.526 -4.603 32.416 1.00 10.62 O

ATOM 5702 OD2 ASP A 360 20.082 -5.010 30.287 1.00 10.39 O1-

ATOM 5703 H ASP A 360 21.820 -2.416 32.525 1.00 0.00 H

ATOM 5704 HA ASP A 360 19.462 -0.856 31.845 1.00 0.00 H

ATOM 5705 HB3 ASP A 360 20.242 -2.426 30.191 1.00 0.00 H

ATOM 5706 HB2 ASP A 360 18.639 -2.877 30.735 1.00 0.00 H

ATOM 5707 N SER A 361 19.280 -2.211 34.615 1.00 8.45 N

ATOM 5708 CA SER A 361 18.411 -2.403 35.783 1.00 9.13 C

ATOM 5709 C SER A 361 17.289 -1.339 35.846 1.00 9.15 C

ATOM 5710 O SER A 361 17.530 -0.213 35.410 1.00 9.17 O

ATOM 5711 CB SER A 361 19.267 -2.397 37.070 1.00 9.22 C

ATOM 5712 OG SER A 361 19.806 -1.123 37.372 1.00 9.26 O

ATOM 5713 H SER A 361 20.263 -2.053 34.807 1.00 0.00 H

ATOM 5714 HA SER A 361 17.995 -3.401 35.663 1.00 0.00 H

ATOM 5715 HB3 SER A 361 20.079 -3.121 36.993 1.00 0.00 H

ATOM 5716 HB2 SER A 361 18.659 -2.711 37.920 1.00 0.00 H

ATOM 5717 HG SER A 361 20.644 -1.047 36.901 1.00 0.00 H

ATOM 5718 N PRO A 362 16.096 -1.679 36.391 1.00 9.67 N

ATOM 5719 CA PRO A 362 15.012 -0.698 36.618 1.00 9.81 C

ATOM 5720 C PRO A 362 15.412 0.595 37.359 1.00 9.69 C

ATOM 5721 O PRO A 362 14.844 1.647 37.066 1.00 10.16 O

ATOM 5722 CB PRO A 362 13.956 -1.492 37.403 1.00 9.72 C

ATOM 5723 CG PRO A 362 14.175 -2.933 36.978 1.00 9.98 C

ATOM 5724 CD PRO A 362 15.687 -3.020 36.812 1.00 10.10 C

ATOM 5725 HA PRO A 362 14.608 -0.434 35.639 1.00 0.00 H

ATOM 5726 HB3 PRO A 362 12.939 -1.152 37.202 1.00 0.00 H

ATOM 5727 HB2 PRO A 362 14.125 -1.406 38.478 1.00 0.00 H

ATOM 5728 HG3 PRO A 362 13.693 -3.098 36.014 1.00 0.00 H

ATOM 5729 HG2 PRO A 362 13.773 -3.661 37.684 1.00 0.00 H

ATOM 5730 HD2 PRO A 362 16.167 -3.255 37.763 1.00 0.00 H

ATOM 5731 HD3 PRO A 362 15.933 -3.801 36.093 1.00 0.00 H

ATOM 5732 N LYS A 363 16.407 0.493 38.258 1.00 9.78 N

ATOM 5733 CA LYS A 363 16.995 1.606 38.997 1.00 10.07 C

ATOM 5734 C LYS A 363 17.783 2.583 38.102 1.00 9.58 C

ATOM 5735 O LYS A 363 17.601 3.791 38.253 1.00 9.83 O

ATOM 5736 CB LYS A 363 17.884 1.038 40.126 1.00 10.29 C

ATOM 5737 CG LYS A 363 18.399 2.097 41.120 1.00 11.94 C

ATOM 5738 CD LYS A 363 19.363 1.516 42.163 1.00 14.66 C

ATOM 5739 CE LYS A 363 19.776 2.557 43.214 1.00 17.69 C

ATOM 5740 NZ LYS A 363 20.794 2.019 44.132 1.00 22.40 N1+

ATOM 5741 H LYS A 363 16.824 -0.411 38.427 1.00 0.00 H

ATOM 5742 HA LYS A 363 16.176 2.162 39.459 1.00 0.00 H

ATOM 5743 HB3 LYS A 363 18.729 0.500 39.691 1.00 0.00 H

ATOM 5744 HB2 LYS A 363 17.315 0.297 40.690 1.00 0.00 H

ATOM 5745 HG3 LYS A 363 17.547 2.554 41.624 1.00 0.00 H

ATOM 5746 HG2 LYS A 363 18.904 2.905 40.593 1.00 0.00 H

ATOM 5747 HD3 LYS A 363 20.249 1.133 41.655 1.00 0.00 H

ATOM 5748 HD2 LYS A 363 18.898 0.661 42.655 1.00 0.00 H

ATOM 5749 HE3 LYS A 363 18.908 2.873 43.794 1.00 0.00 H

ATOM 5750 HE2 LYS A 363 20.182 3.447 42.735 1.00 0.00 H

ATOM 5751 HZ1 LYS A 363 21.621 1.778 43.604 1.00 0.00 H

ATOM 5752 HZ2 LYS A 363 21.032 2.720 44.819 1.00 0.00 H

ATOM 5753 HZ3 LYS A 363 20.436 1.197 44.598 1.00 0.00 H

ATOM 5754 N TYR A 364 18.657 2.056 37.225 1.00 9.53 N

ATOM 5755 CA TYR A 364 19.668 2.853 36.524 1.00 9.35 C

ATOM 5756 C TYR A 364 19.455 3.017 35.011 1.00 8.79 C

ATOM 5757 O TYR A 364 20.163 3.843 34.442 1.00 7.87 O

ATOM 5758 CB TYR A 364 21.076 2.288 36.824 1.00 0.00 C

ATOM 5759 CG TYR A 364 21.595 2.410 38.254 1.00 0.00 C

ATOM 5760 CD1 TYR A 364 21.265 3.510 39.078 1.00 0.00 C

ATOM 5761 CD2 TYR A 364 22.492 1.439 38.744 1.00 0.00 C

ATOM 5762 CE1 TYR A 364 21.824 3.630 40.365 1.00 0.00 C

ATOM 5763 CE2 TYR A 364 23.062 1.562 40.025 1.00 0.00 C

ATOM 5764 CZ TYR A 364 22.725 2.658 40.840 1.00 0.00 C

ATOM 5765 OH TYR A 364 23.266 2.780 42.087 1.00 0.00 O

ATOM 5766 HB2 TYR A 364 21.112 1.241 36.519 1.00 0.00 H

ATOM 5767 HB3 TYR A 364 21.818 2.799 36.207 1.00 0.00 H

ATOM 5768 HD1 TYR A 364 20.601 4.288 38.738 1.00 0.00 H

ATOM 5769 HD2 TYR A 364 22.764 0.604 38.124 1.00 0.00 H

ATOM 5770 HE1 TYR A 364 21.572 4.480 40.981 1.00 0.00 H

ATOM 5771 HE2 TYR A 364 23.754 0.810 40.376 1.00 0.00 H

ATOM 5772 HH TYR A 364 23.965 2.144 42.259 1.00 0.00 H

ATOM 5773 H TYR A 364 18.739 1.051 37.138 1.00 0.00 H

ATOM 5774 HA TYR A 364 19.641 3.876 36.891 1.00 0.00 H

ATOM 5775 N ARG A 365 18.516 2.295 34.372 1.00 7.85 N

ATOM 5776 CA ARG A 365 18.308 2.324 32.911 1.00 7.78 C

ATOM 5777 C ARG A 365 18.006 3.714 32.324 1.00 7.41 C

ATOM 5778 O ARG A 365 18.469 3.998 31.220 1.00 7.01 O

ATOM 5779 CB ARG A 365 17.245 1.287 32.490 1.00 7.94 C

ATOM 5780 CG ARG A 365 15.814 1.546 33.007 1.00 7.40 C

ATOM 5781 CD ARG A 365 14.779 0.534 32.495 1.00 8.72 C

ATOM 5782 NE ARG A 365 15.142 -0.851 32.823 1.00 8.70 N

ATOM 5783 CZ ARG A 365 14.312 -1.907 32.845 1.00 10.30 C

ATOM 5784 NH1 ARG A 365 13.010 -1.785 32.550 1.00 10.11 N

ATOM 5785 NH2 ARG A 365 14.803 -3.109 33.170 1.00 9.06 N1+

ATOM 5786 H ARG A 365 17.974 1.613 34.887 1.00 0.00 H

ATOM 5787 HA ARG A 365 19.247 2.011 32.456 1.00 0.00 H

ATOM 5788 HB3 ARG A 365 17.579 0.312 32.833 1.00 0.00 H

ATOM 5789 HB2 ARG A 365 17.223 1.220 31.401 1.00 0.00 H

ATOM 5790 HG3 ARG A 365 15.505 2.511 32.609 1.00 0.00 H

ATOM 5791 HG2 ARG A 365 15.763 1.641 34.093 1.00 0.00 H

ATOM 5792 HD3 ARG A 365 14.518 0.683 31.448 1.00 0.00 H

ATOM 5793 HD2 ARG A 365 13.870 0.718 33.068 1.00 0.00 H

ATOM 5794 HE ARG A 365 16.126 -1.025 32.989 1.00 0.00 H

ATOM 5795 HH12 ARG A 365 12.397 -2.586 32.578 1.00 0.00 H

ATOM 5796 HH11 ARG A 365 12.632 -0.881 32.306 1.00 0.00 H

ATOM 5797 HH22 ARG A 365 14.208 -3.924 33.198 1.00 0.00 H

ATOM 5798 HH21 ARG A 365 15.796 -3.209 33.346 1.00 0.00 H

ATOM 5799 N LYS A 366 17.278 4.558 33.076 1.00 7.41 N

ATOM 5800 CA LYS A 366 16.977 5.931 32.680 1.00 7.03 C

ATOM 5801 C LYS A 366 18.216 6.833 32.748 1.00 6.82 C

ATOM 5802 O LYS A 366 18.615 7.350 31.709 1.00 7.07 O

ATOM 5803 CB LYS A 366 15.789 6.494 33.484 1.00 0.00 C

ATOM 5804 CG LYS A 366 14.441 5.860 33.084 1.00 0.00 C

ATOM 5805 CD LYS A 366 13.208 6.486 33.764 1.00 0.00 C

ATOM 5806 CE LYS A 366 13.002 7.971 33.420 1.00 0.00 C

ATOM 5807 NZ LYS A 366 11.715 8.487 33.919 1.00 0.00 N1+

ATOM 5808 HB2 LYS A 366 15.960 6.383 34.556 1.00 0.00 H

ATOM 5809 HB3 LYS A 366 15.735 7.566 33.292 1.00 0.00 H

ATOM 5810 HG2 LYS A 366 14.315 5.922 32.002 1.00 0.00 H

ATOM 5811 HG3 LYS A 366 14.464 4.795 33.318 1.00 0.00 H

ATOM 5812 HD2 LYS A 366 12.323 5.920 33.470 1.00 0.00 H

ATOM 5813 HD3 LYS A 366 13.295 6.370 34.845 1.00 0.00 H

ATOM 5814 HE2 LYS A 366 13.797 8.577 33.853 1.00 0.00 H

ATOM 5815 HE3 LYS A 366 13.037 8.113 32.341 1.00 0.00 H

ATOM 5816 HZ1 LYS A 366 10.945 8.023 33.452 1.00 0.00 H

ATOM 5817 HZ2 LYS A 366 11.642 8.348 34.916 1.00 0.00 H

ATOM 5818 HZ3 LYS A 366 11.646 9.474 33.713 1.00 0.00 H

ATOM 5819 H LYS A 366 16.942 4.261 33.980 1.00 0.00 H

ATOM 5820 HA LYS A 366 16.668 5.900 31.634 1.00 0.00 H

ATOM 5821 N ILE A 367 18.817 6.990 33.941 1.00 6.61 N

ATOM 5822 CA ILE A 367 19.971 7.872 34.163 1.00 6.65 C

ATOM 5823 C ILE A 367 21.249 7.456 33.399 1.00 6.41 C

ATOM 5824 O ILE A 367 21.960 8.333 32.913 1.00 6.85 O

ATOM 5825 CB ILE A 367 20.293 8.050 35.676 1.00 6.51 C

ATOM 5826 CG1 ILE A 367 21.370 9.137 35.924 1.00 7.67 C

ATOM 5827 CG2 ILE A 367 20.631 6.722 36.390 1.00 7.20 C

ATOM 5828 CD1 ILE A 367 21.415 9.675 37.360 1.00 8.42 C

ATOM 5829 H ILE A 367 18.456 6.512 34.754 1.00 0.00 H

ATOM 5830 HA ILE A 367 19.680 8.851 33.776 1.00 0.00 H

ATOM 5831 HB ILE A 367 19.377 8.424 36.135 1.00 0.00 H

ATOM 5832 HG13 ILE A 367 21.180 9.985 35.265 1.00 0.00 H

ATOM 5833 HG12 ILE A 367 22.358 8.773 35.646 1.00 0.00 H

ATOM 5834 HG21 ILE A 367 20.777 6.861 37.460 1.00 0.00 H

ATOM 5835 HG22 ILE A 367 19.812 6.014 36.278 1.00 0.00 H

ATOM 5836 HG23 ILE A 367 21.529 6.250 35.997 1.00 0.00 H

ATOM 5837 HD11 ILE A 367 22.232 10.387 37.476 1.00 0.00 H

ATOM 5838 HD12 ILE A 367 20.489 10.190 37.615 1.00 0.00 H

ATOM 5839 HD13 ILE A 367 21.569 8.881 38.090 1.00 0.00 H

ATOM 5840 N SER A 368 21.494 6.142 33.264 1.00 6.25 N

ATOM 5841 CA SER A 368 22.617 5.588 32.507 1.00 6.32 C

ATOM 5842 C SER A 368 22.388 5.655 30.980 1.00 6.48 C

ATOM 5843 O SER A 368 23.362 5.771 30.239 1.00 6.60 O

ATOM 5844 CB SER A 368 22.932 4.175 33.045 1.00 6.78 C

ATOM 5845 OG SER A 368 22.116 3.169 32.479 1.00 7.06 O

ATOM 5846 H SER A 368 20.870 5.468 33.688 1.00 0.00 H

ATOM 5847 HA SER A 368 23.489 6.205 32.731 1.00 0.00 H

ATOM 5848 HB3 SER A 368 22.827 4.145 34.130 1.00 0.00 H

ATOM 5849 HB2 SER A 368 23.973 3.925 32.860 1.00 0.00 H

ATOM 5850 HG SER A 368 21.279 3.159 32.954 1.00 0.00 H

ATOM 5851 N GLY A 369 21.113 5.657 30.544 1.00 6.20 N

ATOM 5852 CA GLY A 369 20.712 5.910 29.160 1.00 6.44 C

ATOM 5853 C GLY A 369 20.932 7.377 28.791 1.00 6.23 C

ATOM 5854 O GLY A 369 21.448 7.649 27.709 1.00 6.49 O

ATOM 5855 H GLY A 369 20.366 5.545 31.215 1.00 0.00 H

ATOM 5856 HA3 GLY A 369 19.658 5.662 29.039 1.00 0.00 H

ATOM 5857 HA2 GLY A 369 21.288 5.293 28.472 1.00 0.00 H

ATOM 5858 N LYS A 370 20.615 8.303 29.712 1.00 5.71 N

ATOM 5859 CA LYS A 370 20.915 9.727 29.585 1.00 6.03 C

ATOM 5860 C LYS A 370 22.424 10.033 29.641 1.00 5.87 C

ATOM 5861 O LYS A 370 22.833 11.024 29.048 1.00 6.42 O

ATOM 5862 CB LYS A 370 20.200 10.529 30.683 1.00 5.76 C

ATOM 5863 CG LYS A 370 18.668 10.512 30.641 1.00 6.42 C

ATOM 5864 CD LYS A 370 18.084 11.316 31.811 1.00 6.05 C

ATOM 5865 CE LYS A 370 16.560 11.220 31.905 1.00 7.93 C

ATOM 5866 NZ LYS A 370 16.046 12.044 33.012 1.00 9.99 N1+

ATOM 5867 H LYS A 370 20.178 8.013 30.577 1.00 0.00 H

ATOM 5868 HA LYS A 370 20.539 10.066 28.619 1.00 0.00 H

ATOM 5869 HB3 LYS A 370 20.502 11.571 30.581 1.00 0.00 H

ATOM 5870 HB2 LYS A 370 20.541 10.193 31.663 1.00 0.00 H

ATOM 5871 HG3 LYS A 370 18.287 9.496 30.660 1.00 0.00 H

ATOM 5872 HG2 LYS A 370 18.327 10.925 29.694 1.00 0.00 H

ATOM 5873 HD3 LYS A 370 18.378 12.362 31.707 1.00 0.00 H

ATOM 5874 HD2 LYS A 370 18.527 10.970 32.747 1.00 0.00 H

ATOM 5875 HE3 LYS A 370 16.254 10.185 32.061 1.00 0.00 H

ATOM 5876 HE2 LYS A 370 16.098 11.555 30.977 1.00 0.00 H

ATOM 5877 HZ1 LYS A 370 15.040 11.938 33.053 1.00 0.00 H

ATOM 5878 HZ2 LYS A 370 16.452 11.741 33.885 1.00 0.00 H

ATOM 5879 HZ3 LYS A 370 16.275 13.015 32.852 1.00 0.00 H

ATOM 5880 N LEU A 371 23.232 9.203 30.323 1.00 5.41 N

ATOM 5881 CA LEU A 371 24.688 9.360 30.372 1.00 4.92 C

ATOM 5882 C LEU A 371 25.358 9.067 29.021 1.00 5.19 C

ATOM 5883 O LEU A 371 26.264 9.803 28.630 1.00 5.41 O

ATOM 5884 CB LEU A 371 25.283 8.475 31.480 1.00 5.58 C

ATOM 5885 CG LEU A 371 26.806 8.645 31.667 1.00 4.57 C

ATOM 5886 CD1 LEU A 371 27.183 10.045 32.173 1.00 6.08 C

ATOM 5887 CD2 LEU A 371 27.340 7.556 32.582 1.00 5.95 C

ATOM 5888 H LEU A 371 22.843 8.409 30.814 1.00 0.00 H

ATOM 5889 HA LEU A 371 24.893 10.401 30.620 1.00 0.00 H

ATOM 5890 HB3 LEU A 371 25.076 7.431 31.247 1.00 0.00 H

ATOM 5891 HB2 LEU A 371 24.775 8.671 32.423 1.00 0.00 H

ATOM 5892 HG LEU A 371 27.318 8.488 30.719 1.00 0.00 H

ATOM 5893 HD11 LEU A 371 27.865 10.006 33.022 1.00 0.00 H

ATOM 5894 HD12 LEU A 371 27.668 10.623 31.386 1.00 0.00 H

ATOM 5895 HD13 LEU A 371 26.304 10.603 32.495 1.00 0.00 H

ATOM 5896 HD21 LEU A 371 28.422 7.633 32.677 1.00 0.00 H

ATOM 5897 HD22 LEU A 371 26.882 7.640 33.566 1.00 0.00 H

ATOM 5898 HD23 LEU A 371 27.119 6.564 32.189 1.00 0.00 H

ATOM 5899 N LEU A 372 24.888 8.017 28.328 1.00 5.40 N

ATOM 5900 CA LEU A 372 25.311 7.684 26.966 1.00 4.86 C

ATOM 5901 C LEU A 372 24.917 8.783 25.970 1.00 5.27 C

ATOM 5902 O LEU A 372 25.705 9.089 25.080 1.00 5.68 O

ATOM 5903 CB LEU A 372 24.693 6.341 26.540 1.00 5.30 C

ATOM 5904 CG LEU A 372 25.192 5.122 27.337 1.00 5.77 C

ATOM 5905 CD1 LEU A 372 24.250 3.931 27.127 1.00 8.24 C

ATOM 5906 CD2 LEU A 372 26.641 4.751 26.979 1.00 6.08 C

ATOM 5907 H LEU A 372 24.156 7.443 28.723 1.00 0.00 H

ATOM 5908 HA LEU A 372 26.398 7.608 26.954 1.00 0.00 H

ATOM 5909 HB3 LEU A 372 24.875 6.162 25.479 1.00 0.00 H

ATOM 5910 HB2 LEU A 372 23.609 6.415 26.644 1.00 0.00 H

ATOM 5911 HG LEU A 372 25.177 5.359 28.400 1.00 0.00 H

ATOM 5912 HD11 LEU A 372 24.809 3.012 26.979 1.00 0.00 H

ATOM 5913 HD12 LEU A 372 23.601 3.795 27.991 1.00 0.00 H

ATOM 5914 HD13 LEU A 372 23.603 4.062 26.259 1.00 0.00 H

ATOM 5915 HD21 LEU A 372 26.993 3.918 27.589 1.00 0.00 H

ATOM 5916 HD22 LEU A 372 26.729 4.457 25.933 1.00 0.00 H

ATOM 5917 HD23 LEU A 372 27.320 5.584 27.149 1.00 0.00 H

ATOM 5918 N THR A 373 23.743 9.397 26.183 1.00 5.40 N

ATOM 5919 CA THR A 373 23.264 10.550 25.432 1.00 6.23 C

ATOM 5920 C THR A 373 24.185 11.785 25.558 1.00 6.41 C

ATOM 5921 O THR A 373 24.454 12.415 24.538 1.00 6.61 O

ATOM 5922 CB THR A 373 21.842 10.962 25.879 1.00 0.00 C

ATOM 5923 OG1 THR A 373 20.965 9.863 25.870 1.00 0.00 O

ATOM 5924 CG2 THR A 373 21.227 12.077 25.047 1.00 0.00 C

ATOM 5925 HB THR A 373 21.859 11.321 26.901 1.00 0.00 H

ATOM 5926 HG1 THR A 373 20.700 9.680 24.963 1.00 0.00 H

ATOM 5927 HG21 THR A 373 20.175 12.226 25.287 1.00 0.00 H

ATOM 5928 HG22 THR A 373 21.751 12.995 25.283 1.00 0.00 H

ATOM 5929 HG23 THR A 373 21.324 11.896 23.979 1.00 0.00 H

ATOM 5930 H THR A 373 23.135 9.072 26.923 1.00 0.00 H

ATOM 5931 HA THR A 373 23.231 10.263 24.379 1.00 0.00 H

ATOM 5932 N LEU A 374 24.680 12.077 26.778 1.00 6.03 N

ATOM 5933 CA LEU A 374 25.648 13.147 27.053 1.00 6.28 C

ATOM 5934 C LEU A 374 27.001 12.928 26.370 1.00 6.45 C

ATOM 5935 O LEU A 374 27.574 13.892 25.862 1.00 6.74 O

ATOM 5936 CB LEU A 374 25.923 13.261 28.562 1.00 0.00 C

ATOM 5937 CG LEU A 374 24.754 13.759 29.412 1.00 0.00 C

ATOM 5938 CD1 LEU A 374 25.168 13.662 30.878 1.00 0.00 C

ATOM 5939 CD2 LEU A 374 24.357 15.184 29.032 1.00 0.00 C

ATOM 5940 HB2 LEU A 374 26.254 12.297 28.947 1.00 0.00 H

ATOM 5941 HB3 LEU A 374 26.768 13.936 28.724 1.00 0.00 H

ATOM 5942 HG LEU A 374 23.880 13.138 29.266 1.00 0.00 H

ATOM 5943 HD11 LEU A 374 24.583 14.307 31.522 1.00 0.00 H

ATOM 5944 HD12 LEU A 374 25.070 12.644 31.252 1.00 0.00 H

ATOM 5945 HD13 LEU A 374 26.207 13.962 30.985 1.00 0.00 H

ATOM 5946 HD21 LEU A 374 23.837 15.688 29.839 1.00 0.00 H

ATOM 5947 HD22 LEU A 374 25.240 15.776 28.792 1.00 0.00 H

ATOM 5948 HD23 LEU A 374 23.694 15.193 28.168 1.00 0.00 H

ATOM 5949 H LEU A 374 24.407 11.510 27.570 1.00 0.00 H

ATOM 5950 HA LEU A 374 25.237 14.086 26.680 1.00 0.00 H

ATOM 5951 N LEU A 375 27.478 11.671 26.386 1.00 5.55 N

ATOM 5952 CA LEU A 375 28.721 11.250 25.748 1.00 6.16 C

ATOM 5953 C LEU A 375 28.675 11.487 24.234 1.00 6.51 C

ATOM 5954 O LEU A 375 29.470 12.284 23.743 1.00 6.52 O

ATOM 5955 CB LEU A 375 29.048 9.787 26.140 1.00 5.66 C

ATOM 5956 CG LEU A 375 30.243 9.120 25.412 1.00 5.76 C

ATOM 5957 CD1 LEU A 375 31.585 9.840 25.662 1.00 6.51 C

ATOM 5958 CD2 LEU A 375 30.333 7.621 25.741 1.00 7.30 C

ATOM 5959 H LEU A 375 26.945 10.945 26.845 1.00 0.00 H

ATOM 5960 HA LEU A 375 29.503 11.897 26.145 1.00 0.00 H

ATOM 5961 HB3 LEU A 375 28.167 9.175 25.951 1.00 0.00 H

ATOM 5962 HB2 LEU A 375 29.206 9.734 27.218 1.00 0.00 H

ATOM 5963 HG LEU A 375 30.052 9.168 24.341 1.00 0.00 H

ATOM 5964 HD11 LEU A 375 32.180 9.884 24.750 1.00 0.00 H

ATOM 5965 HD12 LEU A 375 31.454 10.866 26.001 1.00 0.00 H

ATOM 5966 HD13 LEU A 375 32.183 9.335 26.420 1.00 0.00 H

ATOM 5967 HD21 LEU A 375 31.361 7.282 25.864 1.00 0.00 H

ATOM 5968 HD22 LEU A 375 29.797 7.378 26.656 1.00 0.00 H

ATOM 5969 HD23 LEU A 375 29.896 7.024 24.939 1.00 0.00 H

ATOM 5970 N GLU A 376 27.718 10.842 23.545 1.00 6.60 N

ATOM 5971 CA GLU A 376 27.533 10.932 22.092 1.00 7.24 C

ATOM 5972 C GLU A 376 27.195 12.349 21.585 1.00 7.60 C

ATOM 5973 O GLU A 376 27.619 12.699 20.483 1.00 8.07 O

ATOM 5974 CB GLU A 376 26.465 9.914 21.634 1.00 0.00 C

ATOM 5975 CG GLU A 376 26.747 8.428 21.964 1.00 0.00 C

ATOM 5976 CD GLU A 376 28.044 7.855 21.387 1.00 0.00 C

ATOM 5977 OE1 GLU A 376 28.455 8.304 20.295 1.00 0.00 O

ATOM 5978 OE2 GLU A 376 28.602 6.957 22.053 1.00 0.00 O1-

ATOM 5979 HB2 GLU A 376 25.503 10.184 22.074 1.00 0.00 H

ATOM 5980 HB3 GLU A 376 26.330 10.004 20.558 1.00 0.00 H

ATOM 5981 HG2 GLU A 376 26.776 8.277 23.039 1.00 0.00 H

ATOM 5982 HG3 GLU A 376 25.922 7.816 21.597 1.00 0.00 H

ATOM 5983 H GLU A 376 27.087 10.218 24.033 1.00 0.00 H

ATOM 5984 HA GLU A 376 28.486 10.664 21.633 1.00 0.00 H

ATOM 5985 N CYS A 377 26.504 13.156 22.414 1.00 7.54 N

ATOM 5986 CA CYS A 377 26.232 14.584 22.202 1.00 8.13 C

ATOM 5987 C CYS A 377 27.509 15.435 22.055 1.00 7.57 C

ATOM 5988 O CYS A 377 27.474 16.435 21.339 1.00 8.06 O

ATOM 5989 CB CYS A 377 25.307 15.131 23.317 1.00 0.00 C

ATOM 5990 SG CYS A 377 25.080 16.936 23.338 1.00 0.00 S

ATOM 5991 HB2 CYS A 377 24.324 14.674 23.220 1.00 0.00 H

ATOM 5992 HB3 CYS A 377 25.689 14.847 24.296 1.00 0.00 H

ATOM 5993 HG CYS A 377 24.229 16.988 22.309 1.00 0.00 H

ATOM 5994 H CYS A 377 26.180 12.779 23.295 1.00 0.00 H

ATOM 5995 HA CYS A 377 25.690 14.665 21.259 1.00 0.00 H

ATOM 5996 N SER A 378 28.596 15.021 22.724 1.00 7.30 N

ATOM 5997 CA SER A 378 29.825 15.791 22.865 1.00 6.98 C

ATOM 5998 C SER A 378 30.975 15.330 21.946 1.00 7.33 C

ATOM 5999 O SER A 378 31.989 16.024 21.909 1.00 7.35 O

ATOM 6000 CB SER A 378 30.200 15.770 24.362 1.00 0.00 C

ATOM 6001 OG SER A 378 31.235 16.677 24.663 1.00 0.00 O

ATOM 6002 HB2 SER A 378 29.337 16.050 24.968 1.00 0.00 H

ATOM 6003 HB3 SER A 378 30.496 14.770 24.679 1.00 0.00 H

ATOM 6004 HG SER A 378 32.009 16.436 24.148 1.00 0.00 H

ATOM 6005 H SER A 378 28.549 14.165 23.262 1.00 0.00 H

ATOM 6006 HA SER A 378 29.635 16.821 22.584 1.00 0.00 H

ATOM 6007 N LEU A 379 30.827 14.202 21.226 1.00 6.89 N

ATOM 6008 CA LEU A 379 31.894 13.621 20.399 1.00 7.07 C

ATOM 6009 C LEU A 379 31.852 14.117 18.948 1.00 6.95 C

ATOM 6010 O LEU A 379 30.773 14.301 18.385 1.00 6.73 O

ATOM 6011 CB LEU A 379 31.795 12.083 20.422 1.00 6.66 C

ATOM 6012 CG LEU A 379 32.129 11.449 21.788 1.00 6.65 C

ATOM 6013 CD1 LEU A 379 31.752 9.955 21.789 1.00 7.10 C

ATOM 6014 CD2 LEU A 379 33.586 11.703 22.231 1.00 5.90 C

ATOM 6015 H LEU A 379 29.964 13.679 21.282 1.00 0.00 H

ATOM 6016 HA LEU A 379 32.861 13.911 20.812 1.00 0.00 H

ATOM 6017 HB3 LEU A 379 32.463 11.654 19.673 1.00 0.00 H

ATOM 6018 HB2 LEU A 379 30.786 11.789 20.122 1.00 0.00 H

ATOM 6019 HG LEU A 379 31.510 11.927 22.539 1.00 0.00 H

ATOM 6020 HD11 LEU A 379 32.471 9.336 22.321 1.00 0.00 H

ATOM 6021 HD12 LEU A 379 30.786 9.806 22.270 1.00 0.00 H

ATOM 6022 HD13 LEU A 379 31.665 9.553 20.779 1.00 0.00 H

ATOM 6023 HD21 LEU A 379 34.097 10.792 22.543 1.00 0.00 H

ATOM 6024 HD22 LEU A 379 34.188 12.143 21.436 1.00 0.00 H

ATOM 6025 HD23 LEU A 379 33.617 12.389 23.078 1.00 0.00 H

ATOM 6026 N THR A 380 33.051 14.287 18.367 1.00 6.56 N

ATOM 6027 CA THR A 380 33.265 14.722 16.987 1.00 7.06 C

ATOM 6028 C THR A 380 32.944 13.610 15.979 1.00 7.62 C

ATOM 6029 O THR A 380 33.252 12.444 16.231 1.00 8.87 O

ATOM 6030 CB THR A 380 34.744 15.152 16.787 1.00 7.14 C

ATOM 6031 OG1 THR A 380 34.913 16.357 17.493 1.00 8.16 O

ATOM 6032 CG2 THR A 380 35.267 15.365 15.350 1.00 7.45 C

ATOM 6033 H THR A 380 33.888 14.073 18.889 1.00 0.00 H

ATOM 6034 HA THR A 380 32.602 15.567 16.807 1.00 0.00 H

ATOM 6035 HB THR A 380 35.392 14.414 17.264 1.00 0.00 H

ATOM 6036 HG1 THR A 380 35.693 16.809 17.149 1.00 0.00 H

ATOM 6037 HG21 THR A 380 36.287 15.752 15.362 1.00 0.00 H

ATOM 6038 HG22 THR A 380 35.298 14.432 14.786 1.00 0.00 H

ATOM 6039 HG23 THR A 380 34.659 16.072 14.788 1.00 0.00 H

ATOM 6040 N GLY A 381 32.367 14.023 14.842 1.00 6.57 N

ATOM 6041 CA GLY A 381 31.976 13.141 13.751 1.00 5.84 C

ATOM 6042 C GLY A 381 30.480 13.308 13.470 1.00 5.36 C

ATOM 6043 O GLY A 381 29.789 14.123 14.084 1.00 6.20 O

ATOM 6044 H GLY A 381 32.160 15.006 14.723 1.00 0.00 H

ATOM 6045 HA3 GLY A 381 32.183 12.093 13.969 1.00 0.00 H

ATOM 6046 HA2 GLY A 381 32.558 13.392 12.866 1.00 0.00 H

ATOM 6047 N THR A 382 29.990 12.506 12.519 1.00 5.31 N

ATOM 6048 CA THR A 382 28.581 12.407 12.149 1.00 4.96 C

ATOM 6049 C THR A 382 27.867 11.470 13.132 1.00 5.01 C

ATOM 6050 O THR A 382 28.238 10.299 13.220 1.00 5.19 O

ATOM 6051 CB THR A 382 28.451 11.811 10.729 1.00 5.05 C

ATOM 6052 OG1 THR A 382 29.135 12.674 9.850 1.00 4.73 O

ATOM 6053 CG2 THR A 382 27.011 11.630 10.211 1.00 6.59 C

ATOM 6054 H THR A 382 30.616 11.882 12.026 1.00 0.00 H

ATOM 6055 HA THR A 382 28.115 13.394 12.169 1.00 0.00 H

ATOM 6056 HB THR A 382 28.964 10.849 10.693 1.00 0.00 H

ATOM 6057 HG1 THR A 382 29.246 12.222 9.002 1.00 0.00 H

ATOM 6058 HG21 THR A 382 27.009 11.257 9.186 1.00 0.00 H

ATOM 6059 HG22 THR A 382 26.438 10.924 10.811 1.00 0.00 H

ATOM 6060 HG23 THR A 382 26.475 12.577 10.213 1.00 0.00 H

ATOM 6061 N LEU A 383 26.889 12.009 13.877 1.00 4.50 N

ATOM 6062 CA LEU A 383 26.142 11.258 14.885 1.00 5.41 C

ATOM 6063 C LEU A 383 25.076 10.367 14.228 1.00 5.28 C

ATOM 6064 O LEU A 383 24.363 10.831 13.342 1.00 5.84 O

ATOM 6065 CB LEU A 383 25.539 12.242 15.912 1.00 5.42 C

ATOM 6066 CG LEU A 383 24.737 11.595 17.067 1.00 5.57 C

ATOM 6067 CD1 LEU A 383 25.555 10.592 17.893 1.00 6.65 C

ATOM 6068 CD2 LEU A 383 24.129 12.662 17.986 1.00 6.25 C

ATOM 6069 H LEU A 383 26.621 12.972 13.738 1.00 0.00 H

ATOM 6070 HA LEU A 383 26.849 10.627 15.419 1.00 0.00 H

ATOM 6071 HB3 LEU A 383 24.896 12.950 15.387 1.00 0.00 H

ATOM 6072 HB2 LEU A 383 26.347 12.832 16.344 1.00 0.00 H

ATOM 6073 HG LEU A 383 23.896 11.053 16.633 1.00 0.00 H

ATOM 6074 HD11 LEU A 383 24.996 10.283 18.776 1.00 0.00 H

ATOM 6075 HD12 LEU A 383 25.782 9.686 17.333 1.00 0.00 H

ATOM 6076 HD13 LEU A 383 26.495 11.032 18.226 1.00 0.00 H

ATOM 6077 HD21 LEU A 383 23.155 12.331 18.340 1.00 0.00 H

ATOM 6078 HD22 LEU A 383 24.750 12.849 18.863 1.00 0.00 H

ATOM 6079 HD23 LEU A 383 23.983 13.610 17.470 1.00 0.00 H

ATOM 6080 N TYR A 384 24.978 9.118 14.707 1.00 5.27 N

ATOM 6081 CA TYR A 384 23.945 8.154 14.342 1.00 5.25 C

ATOM 6082 C TYR A 384 23.212 7.726 15.616 1.00 5.75 C

ATOM 6083 O TYR A 384 23.857 7.356 16.600 1.00 5.55 O

ATOM 6084 CB TYR A 384 24.560 6.918 13.655 1.00 5.62 C

ATOM 6085 CG TYR A 384 25.181 7.170 12.295 1.00 5.21 C

ATOM 6086 CD1 TYR A 384 26.432 7.810 12.184 1.00 5.88 C

ATOM 6087 CD2 TYR A 384 24.503 6.766 11.129 1.00 5.79 C

ATOM 6088 CE1 TYR A 384 26.966 8.111 10.918 1.00 6.42 C

ATOM 6089 CE2 TYR A 384 25.048 7.041 9.861 1.00 6.65 C

ATOM 6090 CZ TYR A 384 26.264 7.741 9.756 1.00 5.80 C

ATOM 6091 OH TYR A 384 26.742 8.076 8.526 1.00 6.33 O

ATOM 6092 H TYR A 384 25.630 8.811 15.417 1.00 0.00 H

ATOM 6093 HA TYR A 384 23.234 8.604 13.650 1.00 0.00 H

ATOM 6094 HB3 TYR A 384 23.788 6.156 13.531 1.00 0.00 H

ATOM 6095 HB2 TYR A 384 25.304 6.468 14.303 1.00 0.00 H

ATOM 6096 HD1 TYR A 384 26.972 8.094 13.074 1.00 0.00 H

ATOM 6097 HD2 TYR A 384 23.552 6.264 11.208 1.00 0.00 H

ATOM 6098 HE1 TYR A 384 27.907 8.635 10.843 1.00 0.00 H

ATOM 6099 HE2 TYR A 384 24.519 6.741 8.969 1.00 0.00 H

ATOM 6100 HH TYR A 384 27.555 8.604 8.551 1.00 0.00 H

ATOM 6101 N VAL A 385 21.876 7.754 15.542 1.00 5.21 N

ATOM 6102 CA VAL A 385 20.963 7.263 16.565 1.00 5.59 C

ATOM 6103 C VAL A 385 20.140 6.139 15.916 1.00 5.17 C

ATOM 6104 O VAL A 385 19.443 6.392 14.937 1.00 5.82 O

ATOM 6105 CB VAL A 385 19.998 8.390 17.045 1.00 5.83 C

ATOM 6106 CG1 VAL A 385 18.931 7.913 18.053 1.00 7.11 C

ATOM 6107 CG2 VAL A 385 20.777 9.573 17.654 1.00 5.90 C

ATOM 6108 H VAL A 385 21.436 8.097 14.696 1.00 0.00 H

ATOM 6109 HA VAL A 385 21.507 6.870 17.424 1.00 0.00 H

ATOM 6110 HB VAL A 385 19.464 8.784 16.178 1.00 0.00 H

ATOM 6111 HG11 VAL A 385 18.316 8.745 18.394 1.00 0.00 H

ATOM 6112 HG12 VAL A 385 18.250 7.177 17.624 1.00 0.00 H

ATOM 6113 HG13 VAL A 385 19.396 7.473 18.934 1.00 0.00 H

ATOM 6114 HG21 VAL A 385 20.102 10.348 18.020 1.00 0.00 H

ATOM 6115 HG22 VAL A 385 21.395 9.246 18.491 1.00 0.00 H

ATOM 6116 HG23 VAL A 385 21.433 10.043 16.921 1.00 0.00 H

ATOM 6117 N TYR A 386 20.231 4.911 16.445 1.00 4.92 N

ATOM 6118 CA TYR A 386 19.430 3.784 15.959 1.00 4.81 C

ATOM 6119 C TYR A 386 17.996 3.839 16.519 1.00 4.88 C

ATOM 6120 O TYR A 386 17.788 4.441 17.571 1.00 6.00 O

ATOM 6121 CB TYR A 386 20.155 2.473 16.311 1.00 5.74 C

ATOM 6122 CG TYR A 386 19.454 1.219 15.825 1.00 4.94 C

ATOM 6123 CD1 TYR A 386 19.317 0.974 14.445 1.00 5.36 C

ATOM 6124 CD2 TYR A 386 18.880 0.326 16.745 1.00 5.66 C

ATOM 6125 CE1 TYR A 386 18.616 -0.153 13.980 1.00 5.13 C

ATOM 6126 CE2 TYR A 386 18.140 -0.777 16.285 1.00 5.19 C

ATOM 6127 CZ TYR A 386 18.023 -1.031 14.904 1.00 5.42 C

ATOM 6128 OH TYR A 386 17.334 -2.121 14.461 1.00 6.43 O

ATOM 6129 H TYR A 386 20.817 4.745 17.253 1.00 0.00 H

ATOM 6130 HA TYR A 386 19.358 3.847 14.873 1.00 0.00 H

ATOM 6131 HB3 TYR A 386 20.283 2.411 17.392 1.00 0.00 H

ATOM 6132 HB2 TYR A 386 21.157 2.485 15.882 1.00 0.00 H

ATOM 6133 HD1 TYR A 386 19.747 1.662 13.744 1.00 0.00 H

ATOM 6134 HD2 TYR A 386 18.981 0.509 17.803 1.00 0.00 H

ATOM 6135 HE1 TYR A 386 18.524 -0.332 12.919 1.00 0.00 H

ATOM 6136 HE2 TYR A 386 17.675 -1.431 17.005 1.00 0.00 H

ATOM 6137 HH TYR A 386 16.983 -2.660 15.177 1.00 0.00 H

ATOM 6138 N GLN A 387 17.035 3.205 15.823 1.00 4.95 N

ATOM 6139 CA GLN A 387 15.642 3.112 16.265 1.00 5.58 C

ATOM 6140 C GLN A 387 15.497 2.421 17.632 1.00 5.34 C

ATOM 6141 O GLN A 387 15.903 1.271 17.797 1.00 6.05 O

ATOM 6142 CB GLN A 387 14.743 2.476 15.182 1.00 5.87 C

ATOM 6143 CG GLN A 387 15.126 1.047 14.734 1.00 6.76 C

ATOM 6144 CD GLN A 387 13.972 0.363 14.008 1.00 7.55 C

ATOM 6145 OE1 GLN A 387 13.588 0.788 12.925 1.00 8.24 O

ATOM 6146 NE2 GLN A 387 13.418 -0.702 14.591 1.00 8.11 N

ATOM 6147 H GLN A 387 17.260 2.746 14.951 1.00 0.00 H

ATOM 6148 HA GLN A 387 15.310 4.141 16.389 1.00 0.00 H

ATOM 6149 HB3 GLN A 387 14.710 3.131 14.309 1.00 0.00 H

ATOM 6150 HB2 GLN A 387 13.721 2.468 15.568 1.00 0.00 H

ATOM 6151 HG3 GLN A 387 15.416 0.415 15.570 1.00 0.00 H

ATOM 6152 HG2 GLN A 387 15.988 1.088 14.069 1.00 0.00 H

ATOM 6153 HE22 GLN A 387 12.643 -1.173 14.148 1.00 0.00 H

ATOM 6154 HE21 GLN A 387 13.759 -1.034 15.481 1.00 0.00 H

ATOM 6155 N GLY A 388 14.951 3.165 18.598 1.00 5.68 N

ATOM 6156 CA GLY A 388 14.784 2.722 19.973 1.00 5.61 C

ATOM 6157 C GLY A 388 16.014 3.037 20.847 1.00 5.83 C

ATOM 6158 O GLY A 388 15.970 2.781 22.046 1.00 5.79 O

ATOM 6159 H GLY A 388 14.695 4.125 18.400 1.00 0.00 H

ATOM 6160 HA3 GLY A 388 14.550 1.658 20.029 1.00 0.00 H

ATOM 6161 HA2 GLY A 388 13.917 3.258 20.355 1.00 0.00 H

ATOM 6162 N GLN A 389 17.103 3.616 20.313 1.00 5.70 N

ATOM 6163 CA GLN A 389 18.195 4.145 21.140 1.00 5.29 C

ATOM 6164 C GLN A 389 17.728 5.375 21.941 1.00 5.73 C

ATOM 6165 O GLN A 389 18.119 5.529 23.099 1.00 6.35 O

ATOM 6166 CB GLN A 389 19.410 4.482 20.257 1.00 4.90 C

ATOM 6167 CG GLN A 389 20.657 4.922 21.052 1.00 5.42 C

ATOM 6168 CD GLN A 389 21.844 5.274 20.157 1.00 5.67 C

ATOM 6169 OE1 GLN A 389 21.919 4.868 19.000 1.00 5.73 O

ATOM 6170 NE2 GLN A 389 22.792 6.035 20.702 1.00 4.00 N

ATOM 6171 H GLN A 389 17.135 3.835 19.325 1.00 0.00 H

ATOM 6172 HA GLN A 389 18.501 3.370 21.846 1.00 0.00 H

ATOM 6173 HB3 GLN A 389 19.140 5.273 19.559 1.00 0.00 H

ATOM 6174 HB2 GLN A 389 19.657 3.615 19.644 1.00 0.00 H

ATOM 6175 HG3 GLN A 389 20.964 4.133 21.738 1.00 0.00 H

ATOM 6176 HG2 GLN A 389 20.432 5.798 21.661 1.00 0.00 H

ATOM 6177 HE22 GLN A 389 23.595 6.311 20.155 1.00 0.00 H

ATOM 6178 HE21 GLN A 389 22.716 6.341 21.660 1.00 0.00 H

ATOM 6179 N GLU A 390 16.871 6.195 21.309 1.00 5.51 N

ATOM 6180 CA GLU A 390 16.273 7.391 21.882 1.00 5.69 C

ATOM 6181 C GLU A 390 15.289 7.117 23.025 1.00 6.14 C

ATOM 6182 O GLU A 390 15.205 7.947 23.920 1.00 6.34 O

ATOM 6183 CB GLU A 390 15.653 8.297 20.801 1.00 6.01 C

ATOM 6184 CG GLU A 390 14.297 7.881 20.180 1.00 6.07 C

ATOM 6185 CD GLU A 390 14.327 6.736 19.166 1.00 6.81 C

ATOM 6186 OE1 GLU A 390 15.389 6.110 18.971 1.00 6.28 O

ATOM 6187 OE2 GLU A 390 13.261 6.519 18.554 1.00 6.99 O1-

ATOM 6188 H GLU A 390 16.573 5.979 20.363 1.00 0.00 H

ATOM 6189 HA GLU A 390 17.106 7.944 22.297 1.00 0.00 H

ATOM 6190 HB3 GLU A 390 16.381 8.479 20.009 1.00 0.00 H

ATOM 6191 HB2 GLU A 390 15.503 9.265 21.278 1.00 0.00 H

ATOM 6192 HG3 GLU A 390 13.876 8.747 19.671 1.00 0.00 H

ATOM 6193 HG2 GLU A 390 13.571 7.632 20.952 1.00 0.00 H

ATOM 6194 N ILE A 391 14.582 5.975 22.999 1.00 5.98 N

ATOM 6195 CA ILE A 391 13.669 5.576 24.076 1.00 5.97 C

ATOM 6196 C ILE A 391 14.422 4.914 25.256 1.00 6.26 C

ATOM 6197 O ILE A 391 13.814 4.624 26.284 1.00 5.71 O

ATOM 6198 CB ILE A 391 12.548 4.625 23.555 1.00 0.00 C

ATOM 6199 CG1 ILE A 391 12.994 3.176 23.277 1.00 0.00 C

ATOM 6200 CG2 ILE A 391 11.825 5.242 22.339 1.00 0.00 C

ATOM 6201 CD1 ILE A 391 11.871 2.245 22.845 1.00 0.00 C

ATOM 6202 HB ILE A 391 11.797 4.562 24.337 1.00 0.00 H

ATOM 6203 HG12 ILE A 391 13.713 3.213 22.481 1.00 0.00 H

ATOM 6204 HG13 ILE A 391 13.499 2.725 24.127 1.00 0.00 H

ATOM 6205 HG21 ILE A 391 10.909 4.703 22.096 1.00 0.00 H

ATOM 6206 HG22 ILE A 391 11.539 6.276 22.539 1.00 0.00 H

ATOM 6207 HG23 ILE A 391 12.454 5.240 21.448 1.00 0.00 H

ATOM 6208 HD11 ILE A 391 12.160 1.201 22.964 1.00 0.00 H

ATOM 6209 HD12 ILE A 391 10.981 2.427 23.447 1.00 0.00 H

ATOM 6210 HD13 ILE A 391 11.615 2.399 21.796 1.00 0.00 H

ATOM 6211 H ILE A 391 14.693 5.328 22.232 1.00 0.00 H

ATOM 6212 HA ILE A 391 13.181 6.468 24.472 1.00 0.00 H

ATOM 6213 N GLY A 392 15.732 4.670 25.097 1.00 5.62 N

ATOM 6214 CA GLY A 392 16.546 3.968 26.078 1.00 5.92 C

ATOM 6215 C GLY A 392 16.177 2.482 26.114 1.00 6.54 C

ATOM 6216 O GLY A 392 16.038 1.920 27.201 1.00 6.65 O

ATOM 6217 H GLY A 392 16.187 4.950 24.239 1.00 0.00 H

ATOM 6218 HA3 GLY A 392 16.436 4.413 27.069 1.00 0.00 H

ATOM 6219 HA2 GLY A 392 17.587 4.072 25.779 1.00 0.00 H

ATOM 6220 N GLN A 393 16.008 1.861 24.930 1.00 6.31 N

ATOM 6221 CA GLN A 393 15.771 0.428 24.778 1.00 6.70 C

ATOM 6222 C GLN A 393 16.964 -0.372 25.312 1.00 6.89 C

ATOM 6223 O GLN A 393 18.114 0.025 25.128 1.00 7.21 O

ATOM 6224 CB GLN A 393 15.457 0.089 23.299 1.00 6.84 C

ATOM 6225 CG GLN A 393 15.100 -1.373 22.946 1.00 7.32 C

ATOM 6226 CD GLN A 393 13.869 -1.921 23.670 1.00 7.37 C

ATOM 6227 OE1 GLN A 393 13.867 -2.042 24.891 1.00 9.30 O

ATOM 6228 NE2 GLN A 393 12.829 -2.291 22.919 1.00 4.67 N

ATOM 6229 H GLN A 393 16.116 2.385 24.072 1.00 0.00 H

ATOM 6230 HA GLN A 393 14.900 0.199 25.387 1.00 0.00 H

ATOM 6231 HB3 GLN A 393 16.319 0.363 22.690 1.00 0.00 H

ATOM 6232 HB2 GLN A 393 14.635 0.721 22.964 1.00 0.00 H

ATOM 6233 HG3 GLN A 393 15.937 -2.032 23.169 1.00 0.00 H

ATOM 6234 HG2 GLN A 393 14.941 -1.447 21.871 1.00 0.00 H

ATOM 6235 HE22 GLN A 393 11.996 -2.659 23.357 1.00 0.00 H

ATOM 6236 HE21 GLN A 393 12.858 -2.190 21.915 1.00 0.00 H

ATOM 6237 N ILE A 394 16.630 -1.475 25.980 1.00 6.46 N

ATOM 6238 CA ILE A 394 17.553 -2.416 26.593 1.00 6.88 C

ATOM 6239 C ILE A 394 17.413 -3.767 25.873 1.00 6.30 C

ATOM 6240 O ILE A 394 16.445 -3.970 25.138 1.00 6.82 O

ATOM 6241 CB ILE A 394 17.199 -2.576 28.101 1.00 6.41 C

ATOM 6242 CG1 ILE A 394 15.784 -3.150 28.384 1.00 7.48 C

ATOM 6243 CG2 ILE A 394 17.406 -1.231 28.832 1.00 6.23 C

ATOM 6244 CD1 ILE A 394 15.528 -3.454 29.865 1.00 8.34 C

ATOM 6245 H ILE A 394 15.653 -1.724 26.045 1.00 0.00 H

ATOM 6246 HA ILE A 394 18.587 -2.080 26.493 1.00 0.00 H

ATOM 6247 HB ILE A 394 17.921 -3.278 28.521 1.00 0.00 H

ATOM 6248 HG13 ILE A 394 15.609 -4.069 27.826 1.00 0.00 H

ATOM 6249 HG12 ILE A 394 15.028 -2.446 28.034 1.00 0.00 H

ATOM 6250 HG21 ILE A 394 17.271 -1.317 29.909 1.00 0.00 H

ATOM 6251 HG22 ILE A 394 18.412 -0.849 28.661 1.00 0.00 H

ATOM 6252 HG23 ILE A 394 16.703 -0.472 28.488 1.00 0.00 H

ATOM 6253 HD11 ILE A 394 14.712 -4.169 29.977 1.00 0.00 H

ATOM 6254 HD12 ILE A 394 16.408 -3.878 30.349 1.00 0.00 H

ATOM 6255 HD13 ILE A 394 15.247 -2.549 30.403 1.00 0.00 H

ATOM 6256 N ASN A 395 18.376 -4.676 26.083 1.00 6.13 N

ATOM 6257 CA ASN A 395 18.303 -6.059 25.598 1.00 6.43 C

ATOM 6258 C ASN A 395 17.089 -6.785 26.189 1.00 7.09 C

ATOM 6259 O ASN A 395 16.741 -6.551 27.349 1.00 7.29 O

ATOM 6260 CB ASN A 395 19.581 -6.820 26.014 1.00 6.21 C

ATOM 6261 CG ASN A 395 20.824 -6.509 25.178 1.00 7.37 C

ATOM 6262 OD1 ASN A 395 20.891 -5.515 24.461 1.00 7.66 O

ATOM 6263 ND2 ASN A 395 21.824 -7.385 25.271 1.00 8.27 N

ATOM 6264 H ASN A 395 19.168 -4.447 26.672 1.00 0.00 H

ATOM 6265 HA ASN A 395 18.237 -5.998 24.509 1.00 0.00 H

ATOM 6266 HB3 ASN A 395 19.412 -7.894 25.913 1.00 0.00 H

ATOM 6267 HB2 ASN A 395 19.789 -6.657 27.072 1.00 0.00 H

ATOM 6268 HD22 ASN A 395 22.678 -7.249 24.745 1.00 0.00 H

ATOM 6269 HD21 ASN A 395 21.729 -8.208 25.848 1.00 0.00 H

ATOM 6270 N PHE A 396 16.511 -7.702 25.398 1.00 7.34 N

ATOM 6271 CA PHE A 396 15.573 -8.691 25.919 1.00 8.55 C

ATOM 6272 C PHE A 396 16.297 -9.706 26.818 1.00 9.14 C

ATOM 6273 O PHE A 396 17.502 -9.921 26.674 1.00 9.15 O

ATOM 6274 CB PHE A 396 14.767 -9.348 24.779 1.00 8.27 C

ATOM 6275 CG PHE A 396 15.534 -10.077 23.689 1.00 8.15 C

ATOM 6276 CD1 PHE A 396 16.183 -11.304 23.945 1.00 9.51 C

ATOM 6277 CD2 PHE A 396 15.630 -9.509 22.404 1.00 7.66 C

ATOM 6278 CE1 PHE A 396 16.869 -11.952 22.928 1.00 8.85 C

ATOM 6279 CE2 PHE A 396 16.293 -10.192 21.395 1.00 8.18 C

ATOM 6280 CZ PHE A 396 16.917 -11.403 21.654 1.00 8.39 C

ATOM 6281 H PHE A 396 16.865 -7.865 24.465 1.00 0.00 H

ATOM 6282 HA PHE A 396 14.850 -8.164 26.545 1.00 0.00 H

ATOM 6283 HB3 PHE A 396 14.127 -8.597 24.315 1.00 0.00 H

ATOM 6284 HB2 PHE A 396 14.076 -10.069 25.217 1.00 0.00 H

ATOM 6285 HD1 PHE A 396 16.129 -11.761 24.921 1.00 0.00 H

ATOM 6286 HD2 PHE A 396 15.166 -8.558 22.192 1.00 0.00 H

ATOM 6287 HE1 PHE A 396 17.345 -12.897 23.129 1.00 0.00 H

ATOM 6288 HE2 PHE A 396 16.314 -9.783 20.402 1.00 0.00 H

ATOM 6289 HZ PHE A 396 17.433 -11.922 20.859 1.00 0.00 H

ATOM 6290 N LYS A 397 15.535 -10.289 27.745 1.00 9.96 N

ATOM 6291 CA LYS A 397 16.042 -11.209 28.753 1.00 11.27 C

ATOM 6292 C LYS A 397 15.366 -12.565 28.581 1.00 11.12 C

ATOM 6293 O LYS A 397 14.215 -12.616 28.148 1.00 11.24 O

ATOM 6294 CB LYS A 397 15.732 -10.627 30.152 1.00 11.73 C

ATOM 6295 CG LYS A 397 16.338 -9.235 30.417 1.00 14.28 C

ATOM 6296 CD LYS A 397 17.873 -9.202 30.344 1.00 18.54 C

ATOM 6297 CE LYS A 397 18.437 -7.784 30.452 1.00 21.07 C

ATOM 6298 NZ LYS A 397 19.903 -7.789 30.343 1.00 20.61 N1+

ATOM 6299 H LYS A 397 14.547 -10.082 27.795 1.00 0.00 H

ATOM 6300 HA LYS A 397 17.115 -11.372 28.648 1.00 0.00 H

ATOM 6301 HB3 LYS A 397 16.087 -11.316 30.920 1.00 0.00 H

ATOM 6302 HB2 LYS A 397 14.651 -10.562 30.288 1.00 0.00 H

ATOM 6303 HG3 LYS A 397 16.023 -8.897 31.405 1.00 0.00 H

ATOM 6304 HG2 LYS A 397 15.922 -8.511 29.716 1.00 0.00 H

ATOM 6305 HD3 LYS A 397 18.222 -9.635 29.407 1.00 0.00 H

ATOM 6306 HD2 LYS A 397 18.283 -9.823 31.141 1.00 0.00 H

ATOM 6307 HE3 LYS A 397 18.154 -7.328 31.402 1.00 0.00 H

ATOM 6308 HE2 LYS A 397 18.035 -7.152 29.660 1.00 0.00 H

ATOM 6309 HZ1 LYS A 397 20.219 -6.824 30.390 1.00 0.00 H

ATOM 6310 HZ2 LYS A 397 20.305 -8.311 31.108 1.00 0.00 H

ATOM 6311 HZ3 LYS A 397 20.183 -8.194 29.462 1.00 0.00 H

ATOM 6312 N GLU A 398 16.074 -13.620 29.020 1.00 11.83 N

ATOM 6313 CA GLU A 398 15.576 -14.980 29.263 1.00 12.44 C

ATOM 6314 C GLU A 398 14.871 -15.673 28.071 1.00 12.06 C

ATOM 6315 O GLU A 398 14.000 -16.515 28.289 1.00 12.94 O

ATOM 6316 CB GLU A 398 14.815 -15.043 30.621 1.00 0.00 C

ATOM 6317 CG GLU A 398 13.580 -14.123 30.775 1.00 0.00 C

ATOM 6318 CD GLU A 398 12.920 -14.218 32.147 1.00 0.00 C

ATOM 6319 OE1 GLU A 398 13.635 -13.977 33.145 1.00 0.00 O

ATOM 6320 OE2 GLU A 398 11.704 -14.503 32.171 1.00 0.00 O1-

ATOM 6321 HB2 GLU A 398 14.512 -16.073 30.817 1.00 0.00 H

ATOM 6322 HB3 GLU A 398 15.526 -14.809 31.414 1.00 0.00 H

ATOM 6323 HG2 GLU A 398 13.854 -13.076 30.682 1.00 0.00 H

ATOM 6324 HG3 GLU A 398 12.847 -14.336 29.996 1.00 0.00 H

ATOM 6325 H GLU A 398 17.013 -13.454 29.351 1.00 0.00 H

ATOM 6326 HA GLU A 398 16.478 -15.577 29.403 1.00 0.00 H

ATOM 6327 N TRP A 399 15.288 -15.343 26.835 1.00 11.59 N

ATOM 6328 CA TRP A 399 14.837 -16.008 25.611 1.00 11.77 C

ATOM 6329 C TRP A 399 15.633 -17.310 25.372 1.00 11.90 C

ATOM 6330 O TRP A 399 16.863 -17.246 25.327 1.00 11.96 O

ATOM 6331 CB TRP A 399 15.055 -15.080 24.400 1.00 11.75 C

ATOM 6332 CG TRP A 399 14.061 -13.981 24.165 1.00 12.11 C

ATOM 6333 CD1 TRP A 399 13.318 -13.330 25.087 1.00 12.38 C

ATOM 6334 CD2 TRP A 399 13.742 -13.354 22.892 1.00 12.49 C

ATOM 6335 NE1 TRP A 399 12.593 -12.324 24.479 1.00 13.04 N

ATOM 6336 CE2 TRP A 399 12.828 -12.280 23.120 1.00 12.85 C

ATOM 6337 CE3 TRP A 399 14.146 -13.586 21.561 1.00 12.40 C

ATOM 6338 CZ2 TRP A 399 12.370 -11.451 22.081 1.00 12.78 C

ATOM 6339 CZ3 TRP A 399 13.671 -12.784 20.512 1.00 13.95 C

ATOM 6340 CH2 TRP A 399 12.806 -11.702 20.767 1.00 12.73 C

ATOM 6341 H TRP A 399 16.008 -14.645 26.725 1.00 0.00 H

ATOM 6342 HA TRP A 399 13.767 -16.194 25.677 1.00 0.00 H

ATOM 6343 HB3 TRP A 399 15.029 -15.679 23.487 1.00 0.00 H

ATOM 6344 HB2 TRP A 399 16.054 -14.644 24.430 1.00 0.00 H

ATOM 6345 HD1 TRP A 399 13.301 -13.571 26.136 1.00 0.00 H

ATOM 6346 HE1 TRP A 399 11.997 -11.695 24.997 1.00 0.00 H

ATOM 6347 HE3 TRP A 399 14.830 -14.394 21.343 1.00 0.00 H

ATOM 6348 HZ2 TRP A 399 11.693 -10.634 22.284 1.00 0.00 H

ATOM 6349 HZ3 TRP A 399 13.990 -13.016 19.513 1.00 0.00 H

ATOM 6350 HH2 TRP A 399 12.465 -11.082 19.951 1.00 0.00 H

ATOM 6351 N PRO A 400 14.932 -18.448 25.146 1.00 12.23 N

ATOM 6352 CA PRO A 400 15.519 -19.642 24.497 1.00 12.21 C

ATOM 6353 C PRO A 400 16.106 -19.347 23.102 1.00 11.74 C

ATOM 6354 O PRO A 400 15.610 -18.448 22.422 1.00 11.63 O

ATOM 6355 CB PRO A 400 14.332 -20.616 24.391 1.00 12.73 C

ATOM 6356 CG PRO A 400 13.332 -20.156 25.433 1.00 13.47 C

ATOM 6357 CD PRO A 400 13.507 -18.649 25.412 1.00 12.99 C

ATOM 6358 HA PRO A 400 16.285 -20.048 25.159 1.00 0.00 H

ATOM 6359 HB3 PRO A 400 14.631 -21.653 24.542 1.00 0.00 H

ATOM 6360 HB2 PRO A 400 13.861 -20.548 23.408 1.00 0.00 H

ATOM 6361 HG3 PRO A 400 13.623 -20.544 26.410 1.00 0.00 H

ATOM 6362 HG2 PRO A 400 12.312 -20.481 25.229 1.00 0.00 H

ATOM 6363 HD2 PRO A 400 12.929 -18.211 24.600 1.00 0.00 H

ATOM 6364 HD3 PRO A 400 13.175 -18.217 26.356 1.00 0.00 H

ATOM 6365 N ILE A 401 17.141 -20.107 22.703 1.00 11.85 N

ATOM 6366 CA ILE A 401 17.871 -19.920 21.438 1.00 11.96 C

ATOM 6367 C ILE A 401 16.978 -20.051 20.181 1.00 12.32 C

ATOM 6368 O ILE A 401 17.214 -19.339 19.206 1.00 11.92 O

ATOM 6369 CB ILE A 401 19.096 -20.878 21.301 1.00 0.00 C

ATOM 6370 CG1 ILE A 401 20.024 -20.740 22.532 1.00 0.00 C

ATOM 6371 CG2 ILE A 401 19.894 -20.653 19.989 1.00 0.00 C

ATOM 6372 CD1 ILE A 401 21.282 -21.618 22.502 1.00 0.00 C

ATOM 6373 HB ILE A 401 18.726 -21.904 21.290 1.00 0.00 H

ATOM 6374 HG12 ILE A 401 20.326 -19.702 22.640 1.00 0.00 H

ATOM 6375 HG13 ILE A 401 19.474 -20.980 23.442 1.00 0.00 H

ATOM 6376 HG21 ILE A 401 20.794 -21.264 19.943 1.00 0.00 H

ATOM 6377 HG22 ILE A 401 19.320 -20.915 19.101 1.00 0.00 H

ATOM 6378 HG23 ILE A 401 20.205 -19.613 19.892 1.00 0.00 H

ATOM 6379 HD11 ILE A 401 21.568 -21.929 23.506 1.00 0.00 H

ATOM 6380 HD12 ILE A 401 21.105 -22.525 21.933 1.00 0.00 H

ATOM 6381 HD13 ILE A 401 22.128 -21.092 22.059 1.00 0.00 H

ATOM 6382 H ILE A 401 17.473 -20.848 23.302 1.00 0.00 H

ATOM 6383 HA ILE A 401 18.261 -18.902 21.454 1.00 0.00 H

ATOM 6384 N GLU A 402 15.943 -20.905 20.244 1.00 12.91 N

ATOM 6385 CA GLU A 402 14.954 -21.101 19.184 1.00 13.58 C

ATOM 6386 C GLU A 402 14.026 -19.893 18.918 1.00 13.40 C

ATOM 6387 O GLU A 402 13.438 -19.854 17.838 1.00 13.87 O

ATOM 6388 CB GLU A 402 14.162 -22.414 19.410 1.00 14.57 C

ATOM 6389 CG GLU A 402 13.293 -22.521 20.689 1.00 16.31 C

ATOM 6390 CD GLU A 402 14.001 -23.048 21.945 1.00 18.72 C

ATOM 6391 OE1 GLU A 402 13.262 -23.490 22.850 1.00 20.60 O

ATOM 6392 OE2 GLU A 402 15.250 -22.994 22.003 1.00 17.16 O1-

ATOM 6393 H GLU A 402 15.813 -21.480 21.070 1.00 0.00 H

ATOM 6394 HA GLU A 402 15.521 -21.241 18.262 1.00 0.00 H

ATOM 6395 HB3 GLU A 402 14.853 -23.257 19.365 1.00 0.00 H

ATOM 6396 HB2 GLU A 402 13.504 -22.563 18.552 1.00 0.00 H

ATOM 6397 HG3 GLU A 402 12.470 -23.207 20.484 1.00 0.00 H

ATOM 6398 HG2 GLU A 402 12.828 -21.563 20.924 1.00 0.00 H

ATOM 6399 N LYS A 403 13.920 -18.924 19.853 1.00 12.48 N

ATOM 6400 CA LYS A 403 13.182 -17.670 19.636 1.00 12.74 C

ATOM 6401 C LYS A 403 13.883 -16.684 18.688 1.00 11.52 C

ATOM 6402 O LYS A 403 13.180 -15.919 18.030 1.00 11.49 O

ATOM 6403 CB LYS A 403 12.886 -16.942 20.963 1.00 13.16 C

ATOM 6404 CG LYS A 403 11.724 -17.528 21.777 1.00 15.79 C

ATOM 6405 CD LYS A 403 11.277 -16.599 22.922 1.00 14.83 C

ATOM 6406 CE LYS A 403 10.510 -15.348 22.454 1.00 15.38 C

ATOM 6407 NZ LYS A 403 10.143 -14.488 23.591 1.00 17.71 N1+

ATOM 6408 H LYS A 403 14.431 -19.005 20.722 1.00 0.00 H

ATOM 6409 HA LYS A 403 12.226 -17.925 19.174 1.00 0.00 H

ATOM 6410 HB3 LYS A 403 12.610 -15.917 20.720 1.00 0.00 H

ATOM 6411 HB2 LYS A 403 13.785 -16.856 21.572 1.00 0.00 H

ATOM 6412 HG3 LYS A 403 12.033 -18.490 22.190 1.00 0.00 H

ATOM 6413 HG2 LYS A 403 10.874 -17.736 21.126 1.00 0.00 H

ATOM 6414 HD3 LYS A 403 12.156 -16.284 23.486 1.00 0.00 H

ATOM 6415 HD2 LYS A 403 10.660 -17.167 23.618 1.00 0.00 H

ATOM 6416 HE3 LYS A 403 9.602 -15.641 21.928 1.00 0.00 H

ATOM 6417 HE2 LYS A 403 11.102 -14.745 21.766 1.00 0.00 H

ATOM 6418 HZ1 LYS A 403 10.983 -14.165 24.052 1.00 0.00 H

ATOM 6419 HZ2 LYS A 403 9.621 -13.691 23.256 1.00 0.00 H

ATOM 6420 HZ3 LYS A 403 9.578 -15.011 24.245 1.00 0.00 H

ATOM 6421 N TYR A 404 15.227 -16.696 18.655 1.00 10.08 N

ATOM 6422 CA TYR A 404 16.060 -15.820 17.826 1.00 9.65 C

ATOM 6423 C TYR A 404 15.841 -16.112 16.331 1.00 9.13 C

ATOM 6424 O TYR A 404 15.913 -17.274 15.929 1.00 11.05 O

ATOM 6425 CB TYR A 404 17.542 -16.034 18.187 1.00 9.49 C

ATOM 6426 CG TYR A 404 18.003 -15.565 19.561 1.00 9.61 C

ATOM 6427 CD1 TYR A 404 18.855 -14.448 19.677 1.00 8.33 C

ATOM 6428 CD2 TYR A 404 17.634 -16.270 20.726 1.00 9.85 C

ATOM 6429 CE1 TYR A 404 19.360 -14.067 20.935 1.00 8.49 C

ATOM 6430 CE2 TYR A 404 18.134 -15.888 21.986 1.00 9.56 C

ATOM 6431 CZ TYR A 404 19.013 -14.794 22.087 1.00 9.06 C

ATOM 6432 OH TYR A 404 19.528 -14.434 23.298 1.00 10.31 O

ATOM 6433 H TYR A 404 15.726 -17.379 19.207 1.00 0.00 H

ATOM 6434 HA TYR A 404 15.790 -14.784 18.040 1.00 0.00 H

ATOM 6435 HB3 TYR A 404 18.165 -15.535 17.444 1.00 0.00 H

ATOM 6436 HB2 TYR A 404 17.776 -17.093 18.092 1.00 0.00 H

ATOM 6437 HD1 TYR A 404 19.138 -13.889 18.798 1.00 0.00 H

ATOM 6438 HD2 TYR A 404 16.976 -17.119 20.657 1.00 0.00 H

ATOM 6439 HE1 TYR A 404 20.017 -13.219 21.019 1.00 0.00 H

ATOM 6440 HE2 TYR A 404 17.855 -16.446 22.867 1.00 0.00 H

ATOM 6441 HH TYR A 404 20.154 -13.708 23.233 1.00 0.00 H

ATOM 6442 N GLU A 405 15.581 -15.054 15.545 1.00 7.94 N

ATOM 6443 CA GLU A 405 15.326 -15.149 14.105 1.00 7.20 C

ATOM 6444 C GLU A 405 16.619 -15.245 13.285 1.00 7.11 C

ATOM 6445 O GLU A 405 16.605 -15.884 12.232 1.00 7.78 O

ATOM 6446 CB GLU A 405 14.431 -13.963 13.654 1.00 6.74 C

ATOM 6447 CG GLU A 405 15.046 -12.535 13.663 1.00 6.82 C

ATOM 6448 CD GLU A 405 15.917 -12.143 12.452 1.00 8.46 C

ATOM 6449 OE1 GLU A 405 16.722 -11.196 12.595 1.00 7.32 O

ATOM 6450 OE2 GLU A 405 15.755 -12.774 11.385 1.00 7.37 O1-

ATOM 6451 H GLU A 405 15.590 -14.122 15.937 1.00 0.00 H

ATOM 6452 HA GLU A 405 14.758 -16.064 13.923 1.00 0.00 H

ATOM 6453 HB3 GLU A 405 13.545 -13.956 14.288 1.00 0.00 H

ATOM 6454 HB2 GLU A 405 14.036 -14.181 12.661 1.00 0.00 H

ATOM 6455 HG3 GLU A 405 15.613 -12.394 14.580 1.00 0.00 H

ATOM 6456 HG2 GLU A 405 14.236 -11.810 13.715 1.00 0.00 H

ATOM 6457 N ASP A 406 17.674 -14.554 13.750 1.00 6.31 N

ATOM 6458 CA ASP A 406 18.840 -14.189 12.953 1.00 6.75 C

ATOM 6459 C ASP A 406 19.642 -15.414 12.479 1.00 7.09 C

ATOM 6460 O ASP A 406 19.946 -16.309 13.268 1.00 7.43 O

ATOM 6461 CB ASP A 406 19.655 -13.092 13.681 1.00 6.81 C

ATOM 6462 CG ASP A 406 20.966 -12.624 13.056 1.00 7.03 C

ATOM 6463 OD1 ASP A 406 21.148 -12.797 11.833 1.00 7.47 O

ATOM 6464 OD2 ASP A 406 21.747 -12.003 13.806 1.00 7.84 O1-

ATOM 6465 H ASP A 406 17.590 -14.072 14.636 1.00 0.00 H

ATOM 6466 HA ASP A 406 18.437 -13.711 12.057 1.00 0.00 H

ATOM 6467 HB3 ASP A 406 19.839 -13.386 14.713 1.00 0.00 H

ATOM 6468 HB2 ASP A 406 19.038 -12.199 13.704 1.00 0.00 H

ATOM 6469 N VAL A 407 19.967 -15.393 11.176 1.00 6.69 N

ATOM 6470 CA VAL A 407 20.824 -16.342 10.473 1.00 7.70 C

ATOM 6471 C VAL A 407 22.249 -16.454 11.058 1.00 7.44 C

ATOM 6472 O VAL A 407 22.828 -17.536 10.985 1.00 8.30 O

ATOM 6473 CB VAL A 407 20.904 -15.976 8.964 1.00 7.44 C

ATOM 6474 CG1 VAL A 407 21.496 -14.577 8.686 1.00 8.13 C

ATOM 6475 CG2 VAL A 407 21.635 -17.028 8.111 1.00 8.75 C

ATOM 6476 H VAL A 407 19.666 -14.600 10.628 1.00 0.00 H

ATOM 6477 HA VAL A 407 20.350 -17.319 10.568 1.00 0.00 H

ATOM 6478 HB VAL A 407 19.873 -15.965 8.608 1.00 0.00 H

ATOM 6479 HG11 VAL A 407 21.537 -14.372 7.616 1.00 0.00 H

ATOM 6480 HG12 VAL A 407 20.894 -13.789 9.139 1.00 0.00 H

ATOM 6481 HG13 VAL A 407 22.511 -14.474 9.066 1.00 0.00 H

ATOM 6482 HG21 VAL A 407 21.464 -16.851 7.049 1.00 0.00 H

ATOM 6483 HG22 VAL A 407 22.713 -17.003 8.275 1.00 0.00 H

ATOM 6484 HG23 VAL A 407 21.285 -18.033 8.337 1.00 0.00 H

ATOM 6485 N ASP A 408 22.752 -15.368 11.675 1.00 7.42 N

ATOM 6486 CA ASP A 408 24.001 -15.322 12.437 1.00 7.48 C

ATOM 6487 C ASP A 408 23.974 -16.328 13.602 1.00 7.84 C

ATOM 6488 O ASP A 408 24.903 -17.123 13.718 1.00 7.99 O

ATOM 6489 CB ASP A 408 24.301 -13.887 12.935 1.00 0.00 C

ATOM 6490 CG ASP A 408 25.677 -13.653 13.568 1.00 0.00 C

ATOM 6491 OD1 ASP A 408 26.600 -14.455 13.311 1.00 0.00 O

ATOM 6492 OD2 ASP A 408 25.796 -12.626 14.271 1.00 0.00 O1-

ATOM 6493 HB2 ASP A 408 24.176 -13.178 12.116 1.00 0.00 H

ATOM 6494 HB3 ASP A 408 23.573 -13.650 13.706 1.00 0.00 H

ATOM 6495 H ASP A 408 22.201 -14.518 11.687 1.00 0.00 H

ATOM 6496 HA ASP A 408 24.798 -15.620 11.758 1.00 0.00 H

ATOM 6497 N VAL A 409 22.890 -16.315 14.401 1.00 7.79 N

ATOM 6498 CA VAL A 409 22.709 -17.247 15.516 1.00 8.08 C

ATOM 6499 C VAL A 409 22.419 -18.681 15.034 1.00 8.40 C

ATOM 6500 O VAL A 409 22.987 -19.602 15.616 1.00 8.85 O

ATOM 6501 CB VAL A 409 21.677 -16.788 16.610 1.00 0.00 C

ATOM 6502 CG1 VAL A 409 21.176 -15.345 16.435 1.00 0.00 C

ATOM 6503 CG2 VAL A 409 20.482 -17.720 16.917 1.00 0.00 C

ATOM 6504 HB VAL A 409 22.250 -16.770 17.538 1.00 0.00 H

ATOM 6505 HG11 VAL A 409 20.595 -15.023 17.298 1.00 0.00 H

ATOM 6506 HG12 VAL A 409 21.999 -14.638 16.321 1.00 0.00 H

ATOM 6507 HG13 VAL A 409 20.532 -15.267 15.564 1.00 0.00 H

ATOM 6508 HG21 VAL A 409 19.937 -17.345 17.779 1.00 0.00 H

ATOM 6509 HG22 VAL A 409 19.788 -17.784 16.079 1.00 0.00 H

ATOM 6510 HG23 VAL A 409 20.796 -18.729 17.188 1.00 0.00 H

ATOM 6511 H VAL A 409 22.143 -15.656 14.233 1.00 0.00 H

ATOM 6512 HA VAL A 409 23.677 -17.287 16.018 1.00 0.00 H

ATOM 6513 N LYS A 410 21.574 -18.850 13.996 1.00 8.49 N

ATOM 6514 CA LYS A 410 21.173 -20.163 13.471 1.00 8.47 C

ATOM 6515 C LYS A 410 22.371 -20.962 12.919 1.00 8.27 C

ATOM 6516 O LYS A 410 22.495 -22.147 13.228 1.00 8.85 O

ATOM 6517 CB LYS A 410 19.987 -20.022 12.465 1.00 0.00 C

ATOM 6518 CG LYS A 410 20.289 -20.308 10.975 1.00 0.00 C

ATOM 6519 CD LYS A 410 19.083 -20.193 10.037 1.00 0.00 C

ATOM 6520 CE LYS A 410 19.444 -20.644 8.611 1.00 0.00 C

ATOM 6521 NZ LYS A 410 18.320 -20.455 7.680 1.00 0.00 N1+

ATOM 6522 HB2 LYS A 410 19.215 -20.729 12.772 1.00 0.00 H

ATOM 6523 HB3 LYS A 410 19.521 -19.041 12.566 1.00 0.00 H

ATOM 6524 HG2 LYS A 410 21.060 -19.621 10.629 1.00 0.00 H

ATOM 6525 HG3 LYS A 410 20.693 -21.315 10.867 1.00 0.00 H

ATOM 6526 HD2 LYS A 410 18.260 -20.797 10.421 1.00 0.00 H

ATOM 6527 HD3 LYS A 410 18.732 -19.161 10.024 1.00 0.00 H

ATOM 6528 HE2 LYS A 410 20.297 -20.080 8.235 1.00 0.00 H

ATOM 6529 HE3 LYS A 410 19.732 -21.696 8.608 1.00 0.00 H

ATOM 6530 HZ1 LYS A 410 18.066 -19.477 7.653 1.00 0.00 H

ATOM 6531 HZ2 LYS A 410 18.592 -20.755 6.755 1.00 0.00 H

ATOM 6532 HZ3 LYS A 410 17.529 -20.998 7.992 1.00 0.00 H

ATOM 6533 H LYS A 410 21.141 -18.041 13.570 1.00 0.00 H

ATOM 6534 HA LYS A 410 20.795 -20.727 14.327 1.00 0.00 H

ATOM 6535 N ASN A 411 23.223 -20.292 12.122 1.00 7.68 N

ATOM 6536 CA ASN A 411 24.383 -20.895 11.470 1.00 8.00 C

ATOM 6537 C ASN A 411 25.518 -21.150 12.474 1.00 8.36 C

ATOM 6538 O ASN A 411 26.107 -22.223 12.398 1.00 9.23 O

ATOM 6539 CB ASN A 411 24.851 -20.024 10.281 1.00 7.51 C

ATOM 6540 CG ASN A 411 23.948 -20.037 9.040 1.00 8.52 C

ATOM 6541 OD1 ASN A 411 22.882 -20.646 9.017 1.00 9.67 O

ATOM 6542 ND2 ASN A 411 24.388 -19.351 7.983 1.00 8.86 N

ATOM 6543 H ASN A 411 23.052 -19.316 11.916 1.00 0.00 H

ATOM 6544 HA ASN A 411 24.040 -21.854 11.074 1.00 0.00 H

ATOM 6545 HB3 ASN A 411 25.833 -20.367 9.949 1.00 0.00 H

ATOM 6546 HB2 ASN A 411 24.973 -18.995 10.609 1.00 0.00 H

ATOM 6547 HD22 ASN A 411 23.835 -19.318 7.140 1.00 0.00 H

ATOM 6548 HD21 ASN A 411 25.267 -18.857 8.027 1.00 0.00 H

ATOM 6549 N ASN A 412 25.786 -20.226 13.421 1.00 8.61 N

ATOM 6550 CA ASN A 412 26.784 -20.444 14.483 1.00 8.76 C

ATOM 6551 C ASN A 412 26.374 -21.577 15.434 1.00 8.87 C

ATOM 6552 O ASN A 412 27.244 -22.357 15.813 1.00 9.75 O

ATOM 6553 CB ASN A 412 27.057 -19.142 15.273 1.00 8.64 C

ATOM 6554 CG ASN A 412 28.096 -18.224 14.625 1.00 9.23 C

ATOM 6555 OD1 ASN A 412 29.213 -18.652 14.343 1.00 9.08 O

ATOM 6556 ND2 ASN A 412 27.761 -16.951 14.424 1.00 8.89 N

ATOM 6557 H ASN A 412 25.285 -19.348 13.436 1.00 0.00 H

ATOM 6558 HA ASN A 412 27.691 -20.732 13.946 1.00 0.00 H

ATOM 6559 HB3 ASN A 412 27.500 -19.401 16.235 1.00 0.00 H

ATOM 6560 HB2 ASN A 412 26.131 -18.616 15.506 1.00 0.00 H

ATOM 6561 HD22 ASN A 412 28.412 -16.308 13.998 1.00 0.00 H

ATOM 6562 HD21 ASN A 412 26.822 -16.627 14.616 1.00 0.00 H

ATOM 6563 N TYR A 413 25.072 -21.686 15.752 1.00 9.52 N

ATOM 6564 CA TYR A 413 24.497 -22.776 16.540 1.00 9.88 C

ATOM 6565 C TYR A 413 24.672 -24.145 15.859 1.00 10.49 C

ATOM 6566 O TYR A 413 25.155 -25.072 16.505 1.00 10.28 O

ATOM 6567 CB TYR A 413 23.018 -22.464 16.848 1.00 10.44 C

ATOM 6568 CG TYR A 413 22.296 -23.495 17.694 1.00 11.49 C

ATOM 6569 CD1 TYR A 413 22.371 -23.417 19.097 1.00 12.56 C

ATOM 6570 CD2 TYR A 413 21.553 -24.532 17.089 1.00 13.48 C

ATOM 6571 CE1 TYR A 413 21.710 -24.369 19.893 1.00 13.27 C

ATOM 6572 CE2 TYR A 413 20.885 -25.480 17.888 1.00 13.31 C

ATOM 6573 CZ TYR A 413 20.957 -25.391 19.291 1.00 13.40 C

ATOM 6574 OH TYR A 413 20.292 -26.286 20.073 1.00 14.72 O

ATOM 6575 H TYR A 413 24.418 -20.994 15.409 1.00 0.00 H

ATOM 6576 HA TYR A 413 25.036 -22.805 17.489 1.00 0.00 H

ATOM 6577 HB3 TYR A 413 22.461 -22.329 15.920 1.00 0.00 H

ATOM 6578 HB2 TYR A 413 22.954 -21.511 17.373 1.00 0.00 H

ATOM 6579 HD1 TYR A 413 22.947 -22.633 19.568 1.00 0.00 H

ATOM 6580 HD2 TYR A 413 21.493 -24.599 16.012 1.00 0.00 H

ATOM 6581 HE1 TYR A 413 21.792 -24.324 20.968 1.00 0.00 H

ATOM 6582 HE2 TYR A 413 20.309 -26.266 17.423 1.00 0.00 H

ATOM 6583 HH TYR A 413 20.362 -26.061 21.004 1.00 0.00 H

ATOM 6584 N GLU A 414 24.319 -24.234 14.564 1.00 10.97 N

ATOM 6585 CA GLU A 414 24.475 -25.431 13.731 1.00 12.34 C

ATOM 6586 C GLU A 414 25.925 -25.935 13.646 1.00 12.30 C

ATOM 6587 O GLU A 414 26.155 -27.139 13.733 1.00 12.49 O

ATOM 6588 CB GLU A 414 23.875 -25.166 12.331 1.00 0.00 C

ATOM 6589 CG GLU A 414 24.155 -26.267 11.282 1.00 0.00 C

ATOM 6590 CD GLU A 414 23.465 -25.995 9.950 1.00 0.00 C

ATOM 6591 OE1 GLU A 414 23.844 -24.996 9.300 1.00 0.00 O

ATOM 6592 OE2 GLU A 414 22.578 -26.804 9.600 1.00 0.00 O1-

ATOM 6593 HB2 GLU A 414 22.796 -25.038 12.432 1.00 0.00 H

ATOM 6594 HB3 GLU A 414 24.248 -24.215 11.951 1.00 0.00 H

ATOM 6595 HG2 GLU A 414 25.221 -26.352 11.073 1.00 0.00 H

ATOM 6596 HG3 GLU A 414 23.831 -27.235 11.666 1.00 0.00 H

ATOM 6597 H GLU A 414 23.931 -23.423 14.100 1.00 0.00 H

ATOM 6598 HA GLU A 414 23.891 -26.227 14.195 1.00 0.00 H

ATOM 6599 N ILE A 415 26.862 -24.997 13.466 1.00 12.08 N

ATOM 6600 CA ILE A 415 28.278 -25.284 13.272 1.00 11.88 C

ATOM 6601 C ILE A 415 28.992 -25.673 14.572 1.00 12.25 C

ATOM 6602 O ILE A 415 29.854 -26.549 14.527 1.00 11.97 O

ATOM 6603 CB ILE A 415 28.974 -24.089 12.573 1.00 0.00 C

ATOM 6604 CG1 ILE A 415 28.410 -23.948 11.137 1.00 0.00 C

ATOM 6605 CG2 ILE A 415 30.520 -24.094 12.607 1.00 0.00 C

ATOM 6606 CD1 ILE A 415 28.785 -25.065 10.151 1.00 0.00 C

ATOM 6607 HB ILE A 415 28.670 -23.185 13.106 1.00 0.00 H

ATOM 6608 HG12 ILE A 415 27.323 -23.934 11.159 1.00 0.00 H

ATOM 6609 HG13 ILE A 415 28.669 -22.978 10.733 1.00 0.00 H

ATOM 6610 HG21 ILE A 415 30.932 -23.254 12.055 1.00 0.00 H

ATOM 6611 HG22 ILE A 415 30.906 -24.001 13.622 1.00 0.00 H

ATOM 6612 HG23 ILE A 415 30.933 -25.008 12.181 1.00 0.00 H

ATOM 6613 HD11 ILE A 415 28.522 -24.782 9.132 1.00 0.00 H

ATOM 6614 HD12 ILE A 415 29.849 -25.297 10.167 1.00 0.00 H

ATOM 6615 HD13 ILE A 415 28.239 -25.984 10.368 1.00 0.00 H

ATOM 6616 H ILE A 415 26.588 -24.026 13.387 1.00 0.00 H

ATOM 6617 HA ILE A 415 28.355 -26.155 12.621 1.00 0.00 H

ATOM 6618 N ILE A 416 28.576 -25.080 15.703 1.00 11.80 N

ATOM 6619 CA ILE A 416 29.009 -25.481 17.040 1.00 12.58 C

ATOM 6620 C ILE A 416 28.470 -26.869 17.433 1.00 12.94 C

ATOM 6621 O ILE A 416 29.217 -27.658 18.008 1.00 12.83 O

ATOM 6622 CB ILE A 416 28.625 -24.414 18.106 1.00 12.34 C

ATOM 6623 CG1 ILE A 416 29.523 -23.170 17.930 1.00 12.84 C

ATOM 6624 CG2 ILE A 416 28.651 -24.861 19.586 1.00 13.44 C

ATOM 6625 CD1 ILE A 416 29.022 -21.934 18.678 1.00 12.32 C

ATOM 6626 H ILE A 416 27.875 -24.352 15.660 1.00 0.00 H

ATOM 6627 HA ILE A 416 30.095 -25.552 16.996 1.00 0.00 H

ATOM 6628 HB ILE A 416 27.597 -24.114 17.895 1.00 0.00 H

ATOM 6629 HG13 ILE A 416 29.625 -22.916 16.875 1.00 0.00 H

ATOM 6630 HG12 ILE A 416 30.533 -23.405 18.269 1.00 0.00 H

ATOM 6631 HG21 ILE A 416 28.296 -24.055 20.223 1.00 0.00 H

ATOM 6632 HG22 ILE A 416 28.009 -25.715 19.790 1.00 0.00 H

ATOM 6633 HG23 ILE A 416 29.652 -25.112 19.930 1.00 0.00 H

ATOM 6634 HD11 ILE A 416 29.194 -21.031 18.094 1.00 0.00 H

ATOM 6635 HD12 ILE A 416 27.953 -21.989 18.885 1.00 0.00 H

ATOM 6636 HD13 ILE A 416 29.551 -21.829 19.624 1.00 0.00 H

ATOM 6637 N LYS A 417 27.213 -27.160 17.057 1.00 13.83 N

ATOM 6638 CA LYS A 417 26.583 -28.469 17.213 1.00 14.99 C

ATOM 6639 C LYS A 417 27.283 -29.576 16.401 1.00 15.20 C

ATOM 6640 O LYS A 417 27.414 -30.688 16.908 1.00 15.46 O

ATOM 6641 CB LYS A 417 25.076 -28.349 16.905 1.00 14.49 C

ATOM 6642 CG LYS A 417 24.288 -29.671 16.981 1.00 16.23 C

ATOM 6643 CD LYS A 417 22.764 -29.489 16.898 1.00 17.32 C

ATOM 6644 CE LYS A 417 22.291 -28.891 15.563 1.00 22.39 C

ATOM 6645 NZ LYS A 417 20.821 -28.855 15.480 1.00 25.16 N1+

ATOM 6646 H LYS A 417 26.656 -26.451 16.597 1.00 0.00 H

ATOM 6647 HA LYS A 417 26.671 -28.731 18.265 1.00 0.00 H

ATOM 6648 HB3 LYS A 417 24.943 -27.911 15.917 1.00 0.00 H

ATOM 6649 HB2 LYS A 417 24.638 -27.640 17.610 1.00 0.00 H

ATOM 6650 HG3 LYS A 417 24.536 -30.185 17.910 1.00 0.00 H

ATOM 6651 HG2 LYS A 417 24.602 -30.339 16.178 1.00 0.00 H

ATOM 6652 HD3 LYS A 417 22.432 -28.853 17.720 1.00 0.00 H

ATOM 6653 HD2 LYS A 417 22.287 -30.457 17.055 1.00 0.00 H

ATOM 6654 HE3 LYS A 417 22.671 -29.479 14.727 1.00 0.00 H

ATOM 6655 HE2 LYS A 417 22.664 -27.875 15.442 1.00 0.00 H

ATOM 6656 HZ1 LYS A 417 20.452 -28.288 16.230 1.00 0.00 H

ATOM 6657 HZ2 LYS A 417 20.546 -28.457 14.593 1.00 0.00 H

ATOM 6658 HZ3 LYS A 417 20.455 -29.793 15.553 1.00 0.00 H

ATOM 6659 N LYS A 418 27.735 -29.253 15.178 1.00 15.51 N

ATOM 6660 CA LYS A 418 28.417 -30.195 14.292 1.00 16.53 C

ATOM 6661 C LYS A 418 29.888 -30.427 14.690 1.00 16.26 C

ATOM 6662 O LYS A 418 30.347 -31.566 14.607 1.00 16.63 O
[truncated: 186,332 more chars]
